# Clinical and cost-effectiveness of the digital intervention, MyWay Diabetes, in people with type 2 diabetes living in Greater Manchester during the COVID-19 pandemic

**DOI:** 10.1371/journal.pone.0349232

**Published:** 2026-06-25

**Authors:** Rathi Ravindrarajah, Matthew Gittins, Luke Paterson, Gabriel Rogers, George Tilson, Deborah J. Wake, Doogie Brodie, Scott G. Cunningham, Naresh Kanumilli, Ewan Jones, Peter Elton, Nicola Milne, Michael Morgan-Curran, Evangelos Kontopantelis, Thomas Allen, Martin K. Rutter

**Affiliations:** 1 Division of Nursing, Midwifery & Social Work, Faculty of Biology, Medicine and Health, University of Manchester, Manchester, United Kingdom; 2 Division of Informatics, Imaging and Data Science, School of Health Sciences, Faculty of Biology, Medicine, and Health, The University of Manchester, Manchester, United Kingdom; 3 Manchester Centre for Health Economics, Division of Population Health, Health Services Research & Primary Care, University of Manchester, Manchester, United Kingdom; 4 My Way Digital Health, Dundee, United Kingdom; 5 Centre for Medical Informatics, Usher Institute, University of Edinburgh, Edinburgh, United Kingdom; 6 Population Health & Genomics, School of Medicine, University of Dundee, Dundee, United Kingdom; 7 Northenden Group Practice, Manchester, United Kingdom; 8 Diabetes, Endocrinology & Metabolism Centre, Manchester Royal Infirmary, Manchester, United Kingdom; 9 Greater Manchester & Eastern Cheshire Strategic Clinical Networks, Greater Manchester Health & Social Care Partnership, Manchester, United Kingdom; 10 MyCognition Limited, Sheffield, United Kingdom; 11 Asclepius MedTech Limited, Leeds, United Kingdom; 12 Division of Family Medicine, Yong Loo Lin School of Medicine, National University of Singapore, Singapore; 13 Danish Centre for Health Economics, University of Southern Denmark, Odsense, Denmark; 14 Division of Diabetes, Endocrinology & Gastroenterology, School of Medical Sciences, University of Manchester, Manchester, United Kingdom; Pescara General Hospital, ITALY

## Abstract

**Aims:**

We aimed to assess a) whether using the digital intervention, *MyWay Diabetes* (MWD), during the COVID-19 pandemic in Greater Manchester was associated with improvements in glycaemia and cardiovascular risk factors; and b) the cost-effectiveness of MWD.

**Methods:**

From 01^st^ July 2019–30^th^ April 2022, we assessed risk factor changes in MWD users (n = 507) with T2D, adjusting for changes in a matched control group of non-users (n = 10,135). Economic evaluation was conducted using United Kingdom Prospective Diabetes Study Outcomes Model version 2.1 simulating 1,000 patients over 40-years based on observed risk factor changes.

**Results:**

Over 2-years follow-up, MWD use was associated with a clinically significant reductions in HbA1c of 3.1 (95%CI: 1.5 to 4.6) mmol/mol and decreases in systolic blood pressure (1.4 (0.2 to 2.6) mmHg) and cholesterol (0.1 (0.02 to 0.2) mmol/L) when compared to non-users. Including medication costs, MWD generated more health (0·015 QALYs) and lower costs (−£67 per person) compared to usual care. Excluding medication costs, MWD generated QALYs at a cost of £389 each, below the NICE threshold of £20,000–30,000/QALY.

**Interpretation:**

At population level, the risk factor changes associated with MWD are likely to reduce long-term diabetes-related complications risks and the intervention appears cost-effective.

## Introduction

Diabetes is a serious long-term condition causing premature death and complications such as cardiovascular disease (CVD). Diabetes affects more than 371 million people worldwide, of whom 90% have type 2 diabetes (T2D). Effectively managing T2D requires individuals to make daily lifestyle choices and self-management decisions, guided by the knowledge, support, and advice provided by healthcare services.

Current strategies for T2D management are often inadequate, with data from the UK’s National Audit Office survey showing only 8.2% of patients in 2015 with T2D attended a self-management programme. [1] This highlights the urgent need for innovative interventions. Practical, clinical, and financial considerations make digital solutions an appealing option for supporting T2D self-management. Digital solutions offer clear benefits over usual care when face-to-face care is limited, such as during the COVID-19 pandemic, and in people with limited mobility.

Our study aimed to assess whether using the *MyWay Diabetes* (MWD) digital intervention during the pandemic was associated with improvements in glycaemic control and CVD risk factors in people with T2D, compared to a matched non-user group with T2D. Additionally, we evaluated the cost-effectiveness of MWD based on risk factor changes associated with its use.

## Methods

The development of the MWD intervention, along with the aims and methodology of this study have been published.[2] Briefly, we aimed to assess the clinical and cost-effectiveness of MWD in people with T2D in Greater Manchester (GM) during the COVID-19 pandemic. While we initially planned to evaluate three other digital interventions offered to MWD users (*Changing Health, Oviva, and MyCognition)* [2] uptake was very low, so findings are presented for MWD only.

MWD supports diabetes self-management by providing multimedia resources including traditional information, interactive educational tools and videos on complications.[2] Unlike other digital interventions, the MWD platform directly integrates with NHS primary-care data, enabling seamless access to patient records and more personalised data-driven support. It also allows users to access their primary care clinical records, offering personalised information and explanations about their diagnosis, clinical test results and medication. Content related to key diabetes indicators, such as HbA1c, blood pressure and body weight, is recorded to empower users and improve self-management. The four interventions were initially offered to consenting participants between 01^st^ July 2019 and 31^st^ March 2021, [2] but only MWD was offered from 31^st^ March 2021–30^th^ April 2021. Since then, MWD has been provided as a clinical service across GM.

### Design, setting and study duration

To evaluate effectiveness, we compared outcome changes in MWD users with a matched control group, adjusting for risk factors assessed at the time of intervention. The MWD start date was used as the ‘intervention date’ for matched controls. Primary care data from 1^st^ April 2016 enabled risk factor assessment prior to the study starting in July 2019. Post-intervention data was used up to 31^st^ March 2022.

MyWay Digital Health (MWDH) securely supplied NHS numbers of MWD users to GMCR, allowing their identification. The GM Care Record (GMCR) [3] provided University of Manchester researchers access to pseudonymised data on users and non-users within a secure data environment (SDE).[4] Data was accessed for analysis through the SDE from 01^st^ January 2022 till 30^th^ June 2024.

*Inclusion criteria* for MWD participants were provision of informed consent; age ≥ 18 years; T2D determined from primary care diagnostic codes (Supplementary Material e1.1 in S2 File); registration with a GP in GM consenting to data linkage with MWD, self-certified understanding of English or access to support from family or friends; and access to a digital device [mobile phone and email].

*Inclusion criteria* for controls for non-users of MWD; age ≥ 18 years; T2D determined from primary care records, registration with a GP in GM; and have not opted out for use of Primary Care data in research.

### Sample size

We estimated that with a ratio of 1:10 for participants to controls and assuming a standard deviation[SD] of 15 mmol/mol for HbA1c, [5] 86 participants and 430 controls would provide 80% power to detect a significant (p < 0.05) 5 mmol/mol between-group difference in HbA1c.

*Matching* for birth year (within 1 year) and sex was performed, with each MWD user matched to up to 20 controls not using MWD to increase statistical power. Age and sex were defined at the MWD start date for each user, which served as the index date for the corresponding matched control non-users.

The primary endpoint was the mean post-intervention HbA1c difference between MWD users and controls, adjusted for pre-intervention levels.[6] Secondary outcomes included differences in body weight, blood pressure, lipid levels and urinary albumin creatinine ratio [UACR]. We used multiple imputations with chained equations to handle missing data, generating 10 complete datasets, combined using Rubin’s rules.[7] The imputation model was closely aligned to the analytical models and included outcomes and relevant covariates regardless of missingness, as per best practice; [8] [9] We also assessed population uptake of MWD and medication use (Supplementary Material e1.2 in S2 File).

### Covariates

Models were adjusted for: age, sex, ethnicity, deprivation, smoking and time between pre- and post-intervention outcome assessments, since T2D is a progressive condition with worsening outcomes over time. MWD users had shorter time intervals between outcomes measured pre- and post-intervention compared to controls (Supplementary Table e2.01 in S2 File). Models were also adjusted for baseline risk factors, including: HbA1c, systolic blood pressure, diastolic blood pressure, cholesterol (total, HDL and LDL), creatinine (UACR), triglycerides and body weight. Ethnicity was self-reported. Area-level deprivation was measured using the Index of Multiple Deprivation 2019 (IMD), based on GP postcode.

### Statistical methods assessing clinical effectiveness

Descriptive statistics compared characteristics of MWD users and controls, with data reported at baseline (up to 2 years pre-intervention) and follow-up (up to two years post-intervention). For outcomes with multiple values, we selected the pre-intervention value closest to the intervention date and selected the post-intervention value with the longest follow-up. We present means (SD) for continuous and frequencies (%) for categorical variables. After multiple imputation, we used multivariable linear regression models to estimate adjusted differences in post-intervention outcomes between MWD users and controls. Additional models included interaction terms to examine the influence of ethnicity and deprivation on study results.

*Sensitivity analyses* assessed the impact of a) excluding participants who also used *Changing Health, Oviva and MyCognition* interventions; b) limiting the analysis to individuals with complete pre- and post-intervention outcome data.

### Health economic analysis

To project lifetime costs and effects associated with short-term observed risk factor changes, we used the United Kingdom Prospective Diabetes Study (UKPDS) Outcomes Model version 2.1 (UKPDS-OM2.1) to conduct an economic evaluation from the English NHS perspective.[10, 11] Supplementary Material e3 in S2 File provides comprehensive methods, with Tables e3.01–4.05 highlighting key model parameters and patient characteristics. Briefly, UKPDS-OM2.1 is a validated, patient-level simulation model that predicts relevant events (e.g., myocardial infarction) and long-term outcomes (life expectancy, quality-adjusted life-years [QALYs], and costs) for groups with different baseline characteristics and longitudinal risk profiles.[10] We followed NICE’s reference case, discounting costs (pounds sterling [£] for 2021/22) and QALYs at 3·5% per year over a 40-year time horizon.[12]

Our simulated cohort was based on that used for national policymaking.[12] We derived 1-year treatment effects from the significant differences in pre-and-post-intervention risk factors when comparing treatment groups (HbA1c, SBP, and LDL-cholesterol), and applied these effects to simulated people in the intervention group, with no treatment effect applied to usual care. We also modelled best- and worst-case scenarios based, respectively, on the upper and lower 95% confidence bounds of the relevant effects. In the base-case analysis, we assumed 2-year cost and 1-year effect durations, with natural disease progression thereafter predicted using UKPDS progression equations.[13] We explored alternative durations of cost and effect in sensitivity analysis. Supplementary Material e3.11, Figures e4.01–4.05 in S2 File illustrate the underlying risk-factor progression trajectories for each scenario.

MWDH advised that the full annual licence fee for GM (182,075 people with T2D in February 2024) was £313,557. This fee is currently subject to a negotiated discount; however, following NICE’s stipulation that analyses should only reflect commercial arrangements when prices are nationally guaranteed for a technology’s lifetime, we used the full fee in our base case (see Supplementary Material e3.2 in S2 File). We apportioned the fee among users (n = 16,194 in December 2023) to derive a cost-per-user of £38 over a 2-year cost period. This cost was only applied to people in the model who remained alive – equivalent to assuming that uptake of new user uptake over time offsets attrition among existing users.

Since UKPDS-OM2.1 does not incorporate medication costs, we undertook an additional analysis (described in Supplementary Material e1.3 in S2 File), using baseline and post-intervention participant data from GMCR, mapped to NHS Prescription Service Drug Tariffs (June 2023).[14] Briefly, we estimated post-intervention mean total medication costs for each group using a generalised linear model (log-link and gamma family) adjusting for age, sex, ethnicity, deprivation, smoking status, and medication costs in the 2 years before intervention.

*Sensitivity analyses* explored a range of assumptions including duration and magnitude of effects and costs, uptake levels, intervention costs, and parameter inputs. We also examined different starting populations: people newly diagnosed with T2D (NICE’s ‘initial therapy’ cohort) and the GM population from our study.

*Ethical approval* was granted by the Northwest GM South Research Ethics Committee (reference 261268).

## Results

A total of 507 people with T2D registered to use MWD, matched for age and sex (1:20) with 10,135 controls not using MWD. Among these MWD registrants, 15 (3%) also used *MyCognition*, 7 (1.4%) also used *Changing Health*, 0 (0%) also used *Oviva* and 485 (95.6%) accessed the MWD intervention.

Baseline characteristics of MWD users (cases) and controls are presented in Table 1. Mean age and sex distributions were similar in both groups (54 years; 47% female). 69% of MWD users were White, 14% were Asian, and 7% were of Black ethnicity. In controls, there was a higher proportion of Asian ethnicity (24%) but a lower proportion of Black ethnicity (5%) individuals compared to cases. Approximately half of the participants in both groups were from the most deprived IMD. Diabetes duration was ~ 9 years in both groups.

**Table 1:** Baseline characteristics of MyWay Diabetes users and matched controls not using MyWay Diabetes.

	MWD users		Controls not using MWD		P-value	SMD
	Number	Data	Number	Data		
*N*, %	507	4·8	10,135	95		
Age, years	507	54 ± 12	10,135	54 ± 12	0·99	0.000
Sex, % female	240	47	4,796	47	0·99	0.000
Ethnicity						
White, %	348	69	6,309	62	<0.0001	0.148
Asian, %	69	14	2,404	24		−0.257
Black, %	36	7·1	462	4·6		0.107
Mixed, %	10	2	138	1·4		0.046
Other, %	28	5·5	455	4·5		0.046
Unknown, %	16	3·2	367	3·6		−0.022
IMD						
1, Least Deprived, %	27	5·3	730	7·2	0·14	−0.079
2, %	45	8·9	1102	11		−0.070
3, %	55	11	1077	11		0.000
4, %	117	23	2000	20		0.073
5, Most Deprived, %	263	52	5,226	52		0.000
Diabetes duration, years	341	9·4 ± 6·5	10,133	9·3 ± 6·3	0·91	0.016
HbA1c, mmol/mol	487	61·0 ± 17·9	8,808	60·3 ± 19·6	0·44	0.036
HbA1c, DCCT %	487	7·7 ± 1·7	8,808	7·7 ± 1·8	0·44	0.000
HbA1c > 53 mmol/mol, %	287	57	4,712	47	<0·0001	0.201
Systolic blood pressure, mmHg	463	132·6 ± 15·0	8,508	130·3 ± 15·0	0·001	0.153
Diastolic blood pressure, mmHg	463	79·6 ± 9·6	8,508	78·1 ± 9·7	0·001	0.155
Total cholesterol, mmol/L	466	4·5 ± 1·1	8,361	4·4 ± 1·2	0·02	0.084
LDL cholesterol, mmol/L	407	2·3 ± 1·0	7,205	2·3 ± 1·0	0·1	0.000
HDL cholesterol, mmol/L	464	1·2 ± 0·4	8,062	1·2 ± 0·3	0·08	0.000
Triglycerides, mmol/L	448	2·2 ± 1·6	8,235	2·2 ± 1·6	0·83	0.000
Creatinine, umol/L	481	76 ± 25	8,718	76 ± 39	0·84	0.000
CKD_EPI eGFR, ml/min	481	94 ± 20	8,718	95 ± 20	0·59	−0.050
Urine albumin creatinine ratio, mg/mmol	237	5·3 ± 16·0	3,852	7·3 ± 32·9	0·34	−0.062
Smoking status						
Current, %	53	10.5	1,861	18.4	<0·0001	−0.226
Ex-smoker, %	229	45.2	4,155	41		0.085
Non-smoker, %	215	42.4	3,995	39.4		0.061
Body weight, Kg	452	92·5 ± 21·4	8,023	90·2 ± 22·2	0·0362	0.104
Height, cm	467	168·2 ± 10·1	8,597	167·3 ± 10·8	0·08	0.084
BMI, kg/m2	472	32·9 ± 7·4	9,350	32·9 ± 9·5	0·92	0.000
Admission before intervention, %	98	20	1,965	19	0·97	0.025
Admission after intervention, %	99	20	2,016	20	0·84	0.000

Data are N, %, mean±SD unless stated

All participants had T2D as defined by primary care codes in appendix 1. MWD users with T2D were individually matched for age and sex with up to 20 controls with T2D not using MWD. 169 individuals did not have a T2D diagnosis date.

BMI, body mass index; CKD EPI eGFR, chronic kidney disease epidemiology collaboration estimated glomerular filtration rate; HbA1c, glycosylated haemoglobin; HDL, high density lipoprotein; IMD, index of material deprivation; LDL, low density lipoprotein; SD, standard deviation.

SMD = Standardized Mean Difference. Continuous variables: Cohen’s d with pooled SD. Binary/proportion variables: (p₁ − p₂) / √[(p₁(1 − p₁) + p₂(1 − p₂)) / 2]. |SMD| ≥ 0.2 indicates a small effect

Baseline HbA1c, blood pressure, cholesterol levels were similar between MWD users and controls. However, MWD users had a higher mean body weight (~2 kg) than controls (92.5 kg vs. 90.2 kg) and a lower smoking prevalence (10.5% vs. 18.4%).

The primary analyses included 10,642 individuals (507 MWD users matched with 10,135 controls), following multiple imputation. The extent of missing data is presented in Supplementary Table e2.02 in S2 File. Across outcomes, the proportion of missing data was consistently higher at the post-intervention timepoint than at baseline for both DMW users and controls. For the primary outcome (HbA1c), missingness increased from 3.9% to 21.9% among DMW users and from 13.1% to 31.2% among controls. Similar patterns were observed for secondary outcomes: systolic and diastolic blood pressure missingness rose from 8.7% to 21.5% in users and from 16.1% to 33.4% in controls; total cholesterol missingness increased from 8.1% to 28.2% in users and from 17.5% to 36.8% in controls; and body weight missingness rose from 10.8% to 30.0% in users and from 20.8% to 41.5% in controls. Exploratory outcomes also showed substantial missingness, with post-intervention missing data ranging from 22.1% to 65.3% among users and 31.7% to 74.9% among controls. Smoking status had the lowest missingness overall (1.4% in users and 0.5% in controls). After up to 2 years of follow-up, the multivariable-adjusted post-intervention mean HbA1c level was significantly lower in MWD users than in controls (−3.1 mmol/mol, 95%CI: −4.7 to −1.6) (Table 2; Table e2.01). The corresponding adjusted systolic blood pressure difference was −1.7 mmHg (95%CI: −3.0 to −0.4); total cholesterol was −0.1 mmol/L (95%CI: −0.2 to −0.001); and LDL-cholesterol was −0.1 mmol/L (95%CI: −0.2 to −0.001). No significant difference was observed for the remaining secondary outcome, body weight (Table 2; Table e2.01).

**Table 2:** Multivariable-adjusted mean differences in post-intervention primary, secondary and exploratory outcomes between MWD users and matched controls not using MWD.

	Mean difference	95% CI	p-value
Primary outcome			
HbA1c, mmol/mol	−3·14	−4·66 to −1·62	<0·001
Secondary outcomes			
Systolic blood pressure, mmHg	−1·69	−3·01 to −0·37	0·013
Diastolic blood pressure, mmHg	−1·02	−1·98 to −0·07	0·036
Total cholesterol, mmol/L	−0·11	−0·22 to −0·001	0·049
Body weight, kg	−0·21	−1·06 to 0·65	0·629
Exploratory outcomes			
LDL-cholesterol	−0·09	−0·19 to −0·001	0·048
HDL-cholesterol	0·002	−0·02 to 0·02	0·807
Triglycerides	−0·005	−0·16 to 0·15	0·951
Urinary albumin creatinine ratio	−2·34	−7·25 to 2·58	0·320

MWD users were individually matched for age and sex with controls.

The analysis was performed after multiple imputation of missing data on outcomes and covariates.

Data are multivariable-adjusted differences in post-intervention outcome measures comparing MWD users and controls not using MWD. Negative values indicate that levels are lower in MWD users than in controls.

All models are adjusted for age, sex, ethnicity, deprivation, baseline risk factors (HbA1c, systolic blood pressure, diastolic blood pressure, total, HDL and LDL cholesterol, triglycerides, UACR, smoking and body weight) and time from pre-intervention outcome assessment to post intervention outcome assessment.

When an ethnicity interaction term was added to the model, compared to White MWD users, there were no statistically significant HbA1c changes from baseline for Asian MWD users (−3.3 mmol/mol, 95% CI (−7.1, 0.4)) or other (Black/Mixed/other) ethnic groups (−4.5 mmol/mol, 95%CI (−9.3, 0.24)). Similarly, deprivation (measured by IMD) showed no significant interaction with study outcomes.

### Sensitivity analyses

**Fig 1:**
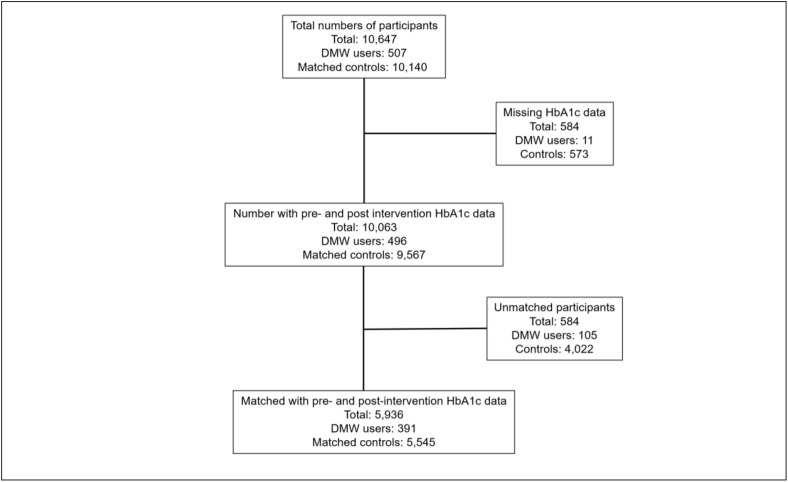
Flow diagram showing the creation of the cohort of matched DMW users and controls not using DMW, who have both pre- and post-intervention HbA1c data.

**Fig 2:**
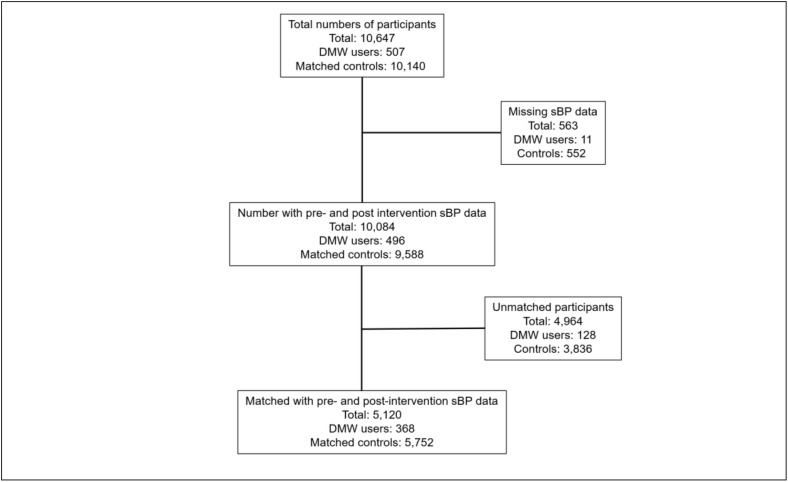
Flow diagram showing the creation of the cohort of matched DMW users and controls not using DMW, who have both pre- and post-intervention systolic blood pressure data.

**Fig 3:**
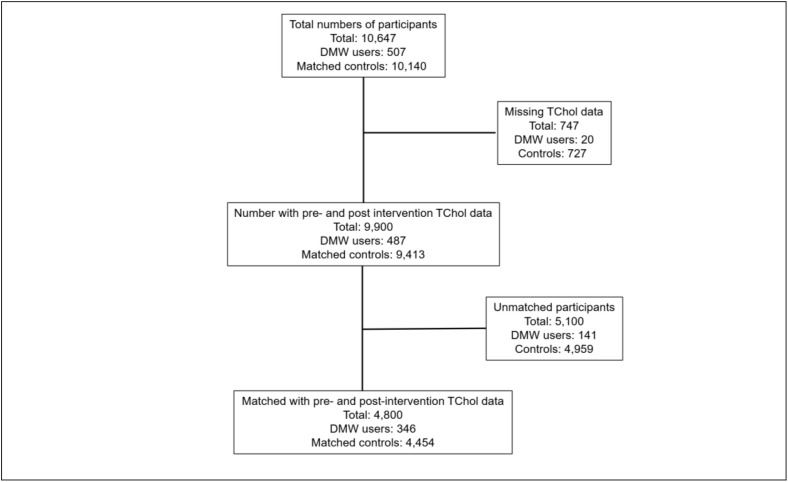
Flow diagram showing the creation of the cohort of matched DMW users and controls not using DMW, who have both pre- and post-intervention total cholesterol data.

**Fig 4:**
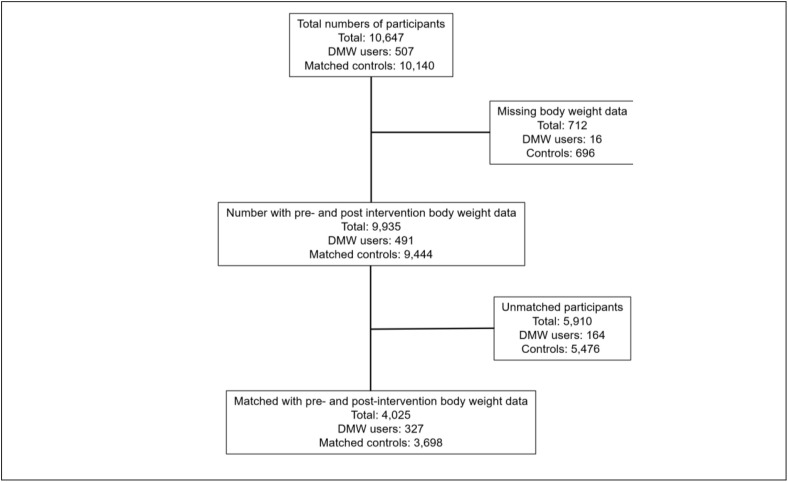
Flow diagram showing the creation of the cohort of matched DMW users and controls not using DMW, who have both pre- and post-intervention body-weight data.

Excluding participants who also used *Changing Health and MyCognition* interventions had no effect on main study outcomes (Supplementary Table e2.03 in S2 File)*.* Restricting the analysis to matched individuals with complete pre- and post-intervention outcome data provided similar results (Figs 1–4; Supplementary Table e2.05 in S2 File)*.*

### Health economics analysis

The UKPDS-OM2.1 model suggests that short-term benefits observed with MWD, compared with usual care, would translate into small reductions in all simulated long-term diabetes-related complications: e.g., 17 fewer myocardial infarctions per 100,000 people over 10 years, equivalent to a number needed to treat of 520 to prevent one event (Supplementary Material e4 in S2 File).

There was a 17% lower change in medication usage in MWD users compared with controls, driven by a post-intervention increase in prescribing in controls that we did not observe in MWD users. This resulted in a 32% lower change in cost of medicines when compared with controls (Supplementary Material e2.1, Tables e2.08–e2.09 in S2 File).

In the base-case cost–utility analysis (Table 3), MWD generates more health (0·015 QALYs) and lower costs (−£67 per person) than usual care, meaning it is ‘dominant’ – both better and, in the long run, cheaper compared with usual care. Excluding medication costs, MWD is no longer net-cost-saving, but generates QALYs at a cost of £389 each, far below the NICE threshold of £20,000–30,000/QALY. 4.1 Table e4.07.

**Table 3:** Base-case economic analyses and sensitivity analyses (patient-level; discounted; 40-year time horizon).

Outcome	Usual care	MWD (WCS / BCS)	Difference (WCS / BCS)
Base case (1-year effects; 2-year costs)			
Costs:			
MWD intervention	··	£38 (£38 / £38)	£38 (£38 / £38)
Diabetes medicines	£1,548	£1,475 (£1,474 / £1,476)	−£73 (−£74 / − £72)
Long-term:			
Complications:			
Amputation	£1,427	£1,418 (£1,421 / £1,413)	−£8 (−£5 / − £14)
Blindness	£1,295	£1,281 (£1,288 / £1,274)	−£14 (−£7 / − £21)
Heart failure	£2,205	£2,207 (£2,206 / £2,208)	£2 (£0 / £2)
Ischaemic heart disease	£3,994	£3,988 (£3,997 / £3,979)	−£7 (£2 / − £15)
Myocardial infarction	£3,227	£3,201 (£3,216 / £3,184)	−£26 (−£10 / − £42)
Renal failure	£2,538	£2,524 (£2,535 / £2,515)	−£14 (−£3 / − £22)
Stroke	£2,259	£2,237 (£2,252 / £2,223)	−£22 (−£7 / − £36)
Ulcer	£375	£371 (£373 / £369)	−£3 (−£2 / − £5)
All complications	£17,319	£17,227 (£17,288 / £17,166)	−£92 (−£31 / − £153)
Other healthcare costs	£17,207	£17,268 (£17,230 / £17,308)	£60 (£23 / £101)
Total long-term	£34,527	£34,495 (£34,519 / £34,475)	−£32 (−£8 / − £52)
Total costs	£36,075	£36,007 (£36,030 / £35,988)	−£67 (−£45 / − £86)
Effects:			
LYs	12·392	12·409 (12·399 / 12·421)	0·017 (0·006 / 0·028)
QALYs	9·875	9·890 (9·881 / 9·901)	0·015 (0·006 / 0·026)
Cost per QALY:			
Including medicines	··	··	Dominant (Dominant / Dominant)
Excluding medicines	··	··	£389 (£4,898 / Dominant)
Sensitivity analyses			
*Permanent costs and effects*			
Total costs	£36,075	£35,722 (£35,803 / £35,636)	−£353 (−£271 / − £438)
QALYs	9·875	9·981 (9·909 / 10·054)	0·106 (0·034 / 0·179)
Cost per QALY:			
Including medicines	··	··	Dominant (Dominant / Dominant)
Excluding medicines	··	··	£1,196 (£6,339 / £196)
*5-year costs and effects*			
Total costs	£36,075	£35,918 (£35,971 / £35,870)	−£157 (−£104 / − £205)
QALYs	9·875	9·913 (9·888 / 9·936)	0·038 (0·013 / 0·061)
Cost per QALY:			
Including medicines	··	··	Dominant (Dominant / Dominant)
Excluding medicines	··	··	£287 (£5,231 / Dominant)
*1-year effect with immediate rebound*			
Total costs	£36,075	£36,035 (£36,044 / £36,022)	−£39 (−£31 / − £52)
QALYs	9·875	9·879 (9·876 / 9·883)	0·004 (0·001 / 0·008)
Cost per QALY:			
Including medicines	··	··	Dominant (Dominant / Dominant)
Excluding medicines	··	··	£8,899 (£36,516 / £2,930)

**Abbreviations**: BCS; Best Case Scenario, MWD; Diabetes My Way, LYs; Life-Years, QALYs; Quality-Adjusted Life-Years, WCS; Worse Case Scenario.

**Note**: WCS and BCS do not constitute confidence intervals. They represent the worst case and best case magnitude of the treatment effects for the examined clinical risk factors.

**Fig 5:**
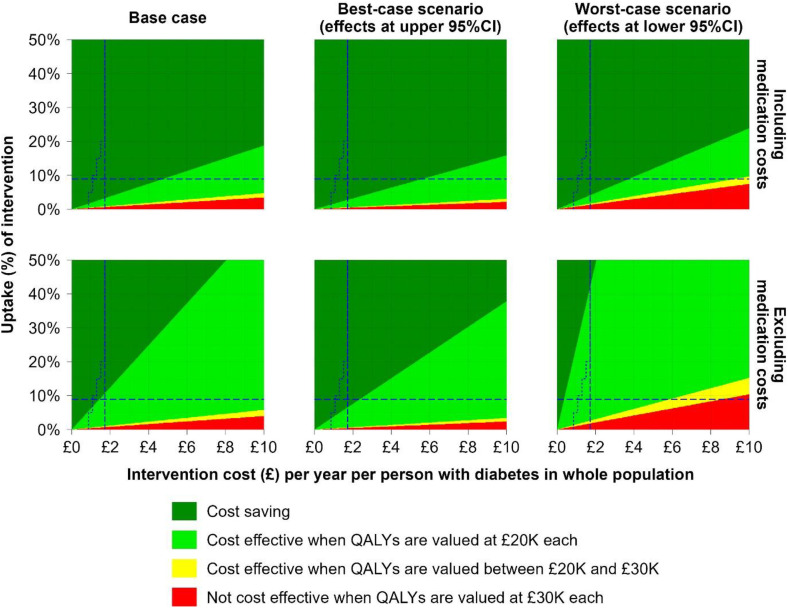
Cost-effectiveness of MyWay Diabetes as a function of cost and uptake.

Fig 5 depicts the impact of intervention cost and uptake on cost-effectiveness. We can see that an intervention with MWD’s observed effects is likely to represent good value for money as long as uptake exceeds 5% and cost-per-patient-per-year is less than £10. Under most circumstances, it will also be net-cost-saving, unless we adopt pessimistic effect estimates and exclude estimated medication costs.

Across a series of sensitivity analyses, all results indicated the intervention was cost-saving or cost-effective by NICE standards (Supplementary Material e4.2, Tables e4.04-4.06 and section 4.5 in S2 File) MWD remains dominant if we assume costs and effects are longer-lasting or permanent but, in these scenarios, has marginally worse cost effectiveness if we exclude medication costs; this is because the diminishing returns of the intervention are very slightly outweighed by its diminishing costs.

Supplementary Material e4.3–e4.5 4.3 and 4.4 in S2 File show scenario analyses with different starting populations. In both cases, health gains are slightly lower but cost reductions slightly greater, with the net result that cost–utility results are similar to the base case.

## Discussion

### Main findings

Over a period of up to 2-years, including the COVID-19 pandemic, the use of MWD was associated with a clinically significant 3.1 mmol/mol reduction in HbA1c, alongside reductions in systolic blood pressure and LDL-cholesterol compared to non-users. Our analysis also demonstrated that MWD is net cost-saving from an NHS perspective.

### Strengths and limitations

Our study has several strengths: a) the cohort from this pragmatic real-world observational study is likely to be more representative of the target population than one recruited to a more costly and time-consuming RCT, and is less prone to be affected by control group contamination through MWD’s Massive Open Online Courses and other online content; b) the inclusion of a carefully selected, prospectively studied matched control group, along with extensive covariate adjustment, allowed for stronger causal inferences regarding risk factor changes attributed to MWD use; c) sensitivity analyses demonstrated the robustness of our main findings; d) our economic analysis adhered to national decision-making standards based on NICE guidance, [12] exploring effects across different patient populations and estimating time-path trajectories using published equations rather than assuming linear relationships.

Our study limitations are: a) the observational design, rather than an RCT, limits causal inference; b) the follow-up of up to 2 years restricts assessment of long-term MWD effects; c) the COVID-19 pandemic may have influenced health behaviours, and limited GP practice engagement (now mitigated by a streamlined NHS login); d) early adopters may be more motivated and health-conscious than non-users; e) the study was limited to people with T2D in Greater Manchester, requiring evaluations of other geographical areas and those living with type 1 diabetes; f) limited statistical power reduced the robustness of interaction and sub-group analyses; g) data on MWD usage/participant engagement was not collected, limiting our ability to assess the impact of motivation and sustained use on effectiveness; h) the health economics analysis was limited by: 1) the absence of a probabilistic sensitivity analysis (this was not computationally feasible), which limits assessment of parameter uncertainty; 2) having a base-case assumption that as people die new people join – an open cohort would be ideal but cannot be achieved with the UKPDS outcomes model; [15] 3) using the UKPDS outcomes model [10, 11] based on an older cohort that did not use newer drug classes; [16] and, 4) reliance on short-term data without knowing long-term treatment effects and/or costs.

### Comparison with prior literature

No other diabetes digital intervention uses primary care data to provide population-level personalised automated online support, making direct comparison with other interventions challenging. The combination of online structured education, NHS data provision, remote monitoring support through a data app and open access content is unique.

However, the HbA1c reductions associated with MWD use are consistent with the clinical impacts of a wide range of T2D digital interventions as highlighted in several systematic reviews and meta-analyses.[17–19] These interventions included strategies such as self-monitoring glucose and CVD risk factors, reminders to initiate self-care activities, automated feedback on self-reported data, real-time support through text messages and/or phone consultation, personalised feedback from clinicians on data uploads and AI-driven systems providing personalised self-care training. Whilst many of these systems required considerable clinical input and resources, the HbA1c improvements reported (3.3–4.3 mmol/mol), [17–20] were comparable to those associated with MWD use: 3.1 (95% CI: 1.5 to 4.6) mmol/mol.

In the meta-analysis by Cui *et al.,* four studies assessed the impact of digital interventions on blood pressure.[17] These interventions included providing blood pressure monitors, mobile apps, prompting monitoring and recording, and sending real-time educational messages. Although the results were not statistically significant (e.g., systolic blood pressure reduction 2.6 (5.6 to −0.4) mmHg; p = 0.08, compared to usual care), the magnitude of the reduction was similar to that associated with MWD use (1.6 (0.3 to 2.8) mmHg).

Three studies from the same meta-analysis evaluated the effect of digital interventions on lipids.[17] While no statistically significant results were found, e.g., total cholesterol lowering of 0.15 (0.6 to −0.3) mmol/L, the magnitude of the reduction was similar to that associated with MWD use 0.10 (0.00 to 0.20) mmol/L.

As observed in our observational study, Cui et al showed no significant impact of digital interventions on body weight across the four studies that evaluated this outcome.[17] Likewise, a recent review of systematic reviews found that mobile applications were not associated with significant improvements in body weight, blood pressure, or lipids.[21]

Other studies of the cost-effectiveness of digital interventions for people with T2D have shown reductions in medication use, but no clear evidence of beneficial impacts on health or social care utilisation or costs.[22]

Our finding that MWD is net cost-saving aligns with the overall conclusion of the MWD evaluation in Scotland, although our study employed more conservative assumptions (including assuming a full fee in base case; see Supplementary Material, e3.2 in S2 File), resulting in a lower estimate of cost-effectiveness.[23] This is also consistent with general trends in the literature showing that digital interventions supporting T2D self-management are good value for money for healthcare systems, [24] primarily due to lower labour costs compared with delivering traditional lifestyle interventions. For example, a cost-effective UK in-person diabetes education and self-management intervention reported slightly higher QALYs gained (0·0392) but with higher relative costs (+£82 per person) largely driven by the intervention costs (+£209 per person).[25]) Digital interventions have the added potential to become more cost-effective/saving as uptake increases, spreading the cost across more users.[26]

### Mechanistic insights

Although our study was not designed to investigate mechanisms behind MWD’s impact on risk factors, improvements in diet or physical activity, driven by increased awareness of key lifestyle factors, could have played a role. Notably, MWD users did not increase their diabetes medication prescriptions (while controls did), and there were no intervention-related changes in body weight, suggesting that these factors were not mediating factors.

Our finding that MWD use was associated with a smaller change in prescribing costs requires careful consideration. The difference-in-differences design of our analysis makes residual confounding alone an unlikely explanation. While it’s possible that individuals who signed up for MWD had varying propensities to receive diabetes medications (which we may not have fully accounted for), it is harder to argue that participants self-selected as those who would not alter their medication use over time. In fact, the intervention cohort had higher baseline exposure to diabetes medications than controls, yet the groups converged during the intervention period. This suggests that the intervention may have helped prevent the escalation of prescribing in MWD users, which was observed in the control group. Given the uncertainty surrounding these dynamics, we provide full cost-effectiveness results both with and without estimated medication costs. Reassuringly, MWD remains good value in both scenarios. While the smaller change in medication costs observed among MWD users is encouraging, it may reflect differences in prescribing behaviour or medication adherence rather than a direct effect of the intervention, and this potential influence should be considered when interpreting the findings.

### Clinical implications

We demonstrated that MWD can be effectively delivered to people with T2D during the COVID-19 pandemic, showing clinically meaningful improvements in glycaemic control and improvements in blood pressure and cholesterol that would be expected to reduce risks for long-term diabetes-related complications. Since MWD does not directly involve clinical staff input, it could lessen clinical primary care workload. Given the ‘real-world’ design (not an RCT), the findings are likely to be highly generalisable. We also showed that MWD was cost-effective and potentially cost saving when the cost of medication was included, making it appealing to financially constrained health services.

### Future research opportunities

Future studies in the post-COVID-19 period should target larger diverse ideally nationwide cohorts, including people with type 1 diabetes, allowing greater statistical power for subgroup analyses by ethnicity [27] and deprivation for example. It would be useful to compare effects of MWD and other online interventions including the NHS *Healthy Living* programme.[28] Large cohorts would be needed to assess if combining these interventions offers added benefits. Further studies should assess medication adherence [29] and effects in subgroups defined by age and digital literacy. A scoping review concluded that T2D digital interventions can be cost-saving or cost-effective but poor reporting quality limits comparisons between interventions and decision making when commissioning services. Therefore, general improvements to study reporting could be valuable.[30]

### Conclusions

Using MWD for up to 2-years, including during the COVID-19 pandemic, was associated with a clinically relevant improvement in HbA1c and improvements in blood pressure and cholesterol when compared to non-users. At a population level, these changes would likely reduce risks for long-term diabetes complications. We found MWD to be net cost-saving from an NHS perspective, which could be attractive to financially constrained health services.

## Supporting information

S1 File STROBE-checklist-v4-combined. (DOCX)

S2 File Supplementary Material. (DOCX)
