# Supplementary material for: Clinical and cost-effectiveness of the digital intervention, MyWay Diabetes, in people with type 2 diabetes living in Greater Manchester during the COVID-19 pandemic
Source: PLoS One. 2026 Jun 25;21(6):e0349232. doi: 10.1371/journal.pone.0349232 (PMC13298753; doi:10.1371/journal.pone.0349232)
Supplement: S2 File — (DOCX) [file pone.0349232.s002.docx]

**Clinical and cost-effectiveness of the digital intervention, MyWay Diabetes, in people with type 2 diabetes living in Greater Manchester during the covid-19 pandemic**

Rathi Ravindrarajah, Matthew Gittins, Luke Paterson, Gabriel Rogers, Deborah J Wake, Doogie Brodie, Scott G Cunningham, Naresh Kanumilli, Ewan Jones, Nicola Milne, Michael Morgan-Curran, Evangelos Kontopantelis, Thomas Allen, Martin K Rutter

**ELECTRONIC SUPPLEMENTARY MATERIAL**

Contents

[e1: Quantitative Analysis Methods 2](#_Toc195182158)

[e1.1 Type 2 diabetes diagnostic codes 2](#_Toc195182159)

[e1.2 General classification of medication 2](#_Toc195182160)

[e1.3 Prescription/medication analysis 2](#_Toc195182161)

[e2: Additional quantitative analysis results 3](#_Toc195182162)

[e2.1 Analysis of effectiveness 3](#_Toc195182163)

[e2.2 Prescription/medication analysis 8](#_Toc195182164)

[e3: Heath Economics Modelling Methods 19](#_Toc195182165)

[e3.1 Overview 19](#_Toc195182166)

[e3.2 DMW usual Pricing Structure 19](#_Toc195182167)

[e3.3 Intervention and Comparator 19](#_Toc195182168)

[e3.4 Microsimulation Model and Simulated Cohort 19](#_Toc195182169)

[e3.5 Treatment Effects 22](#_Toc195182170)

[e3.6 Cost Inputs 22](#_Toc195182171)

[e3.7 Health Utility Inputs 26](#_Toc195182172)

[e3.8 Presenting Results 26](#_Toc195182173)

[e3.9 Planned Sensitivity Analysis 27](#_Toc195182174)

[e3.10 Planned Scenario Analysis 30](#_Toc195182175)

[e4: Health Economics Modelling Results 33](#_Toc195182176)

[e4.1 Time path trajectories 33](#_Toc195182177)

[e4.2 Base-case population (weighted average of NICE cohorts) 38](#_Toc195182178)

[e4.3 Greater Manchester Diabetes My Way population 45](#_Toc195182179)

[e4.4 NICE initial population 53](#_Toc195182180)

[e4.5 UKPDS-OM default inputs 61](#_Toc195182181)

[e5: Consolidated Health Economic Evaluation Reporting Standards 2022 (CHEERS 2022) Checklist 68](#_Toc195182182)

[References 70](#_Toc195182183)

# Quantitative Analysis Methods

## Type 2 diabetes diagnostic codes

Type 2 diabetes was determined based on the following primary care diagnosis codes: <https://github.com/rw251/gm-idcr/tree/master/shared/clinical-code-sets/conditions/diabetes-type-ii/1>

## General classification of medication

1. The anti-diabetic list contains: alpha glucosidase inhibitors (Agis), dipeptidyl peptidase 4 inhibitors (DPP4i), Glucagon-like peptide−1 (GLP−1) receptor agonists, insulin, meglitinide, metformin, Sodium-glucose cotransporter−2 (SGLT2) inhibitors, sulphonylureas, and thiazolidinediones.

2. The antihypertensive list contains: angiotensin-converting enzyme (ACE) inhibitors, alpha blockers, angiotensin II receptor blockers, beta blockers, calcium channel blockers, central-acting drugs, combined, diuretics, peripheral adrenergic inhibitors, and vasodilators.

3. The antiplatelet list contains: aspirin, aspirin/dipyridamole, clopidogrel, and other antiplatelets.

4. The lipid-lowering list contains: statin, fibrate, ezetimibe, and other lipid-lowering drugs.

## Prescription/medication analysis

Prescription issued date, name, dosage (if recorded), and quantity were obtained from the GMCR for any Endocrine, Cardiovascular, and Central Nervous System related medications received by study participants. Each medication was firstly re-classified using their common nomenclature: metformin, sulphonylurea, pioglitazone, DPP4i, SGLT2i, GLP1-RA, insulin, statin, fibrate, ezemtimbe, and other lipid lowering drug. These were then further classified into anti-diabetic drugs, antihypertensive, anti-platelet, and lipid-lowering (see supplementary for details). In addition, prescriptions were also sub-categorised if obtained in combination with another medication (e.g. metformin & sulphonylurea), or in isolation (e.g. metformin only), and any presence of the medication (e.g. metformin & another medication or metformin only).

Summaries were then produced for the number prescriptions obtained, average dose, and total cost for anti-diabetic drugs only (based on individual costs, by quantity) during the two years prior and post intervention. The change two years pre and post intervention were then compared for intervention vs control groups using a Generalised Linear Model assuming a gamma distribution. The adjustment covariate set from the main analysis was applied.

# Additional quantitative analysis results

## Analysis of effectiveness

Table e2.01: Average time intervals between the assessment of outcomes pre-intervention and post-intervention in DMW users compared to matched controls not using DMW, along with between-group time interval differences

| Outcome | DMW users | | Controls not using DMW | | Mean (95% CI) between‑group difference, days | p-value for between-group difference |
| --- | --- | --- | --- | --- | --- | --- |
|  | N | Mean (SD), days | N | Mean (SD), days |  |  |
| **Primary outcome** |  |  |  |  |  |  |
| HbA1c | 391 | 437 (223) | 5, 545 | 531 (225) | 94 (71, 117) | <0·001 |
| **Secondary outcomes** |  |  |  |  |  |  |
| Systolic blood pressure | 368 | 487 (263) | 4, 752 | 573 (244) | 85 (59, 111) | <0·001 |
| Diastolic blood pressure | 368 | 487 (264) | 4, 752 | 573 (245) | 86 (59, 111) | <0·002 |
| Total cholesterol | 346 | 489 (246) | 4, 453 | 583 (239) | 94 (68, 120) | <0·001 |
| Body weight | 327 | 502 (252) | 3, 698 | 588 (238) | 87 (60, 114) | <0·001 |
| **Exploratory outcomes** |  |  |  |  |  |  |
| LDL cholesterol | 284 | 490 (240) | 2, 941 | 580 (602) | 90 (60, 120) | <0·001 |
| HDL cholesterol | 339 | 500 (251) | 4, 126 | 579 (243) | 80 (53, 107) | <0·001 |
| Triglycerides | 325 | 485 (244) | 4, 112 | 574 (237) | 88 (62, 116) | <0·001 |
| Urine albumin creatinine ratio | 114 | 539 (250) | 455 | 617 (250) | 78 (27, 130) | 0·003 |

Numbers are less than 507 due to the availability of paired data, both pre- and post-intervention
HbA1c, glycosylated haemoglobin; HDL, high-density lipoprotein; LDL, low-density lipoprotein

Table e2.02: Proportion of missing data for DMW users and matched controls not using DMW at pre-and post-intervention assessments

| Outcomes | DMW users | | Controls | |
| --- | --- | --- | --- | --- |
|  | Pre-intervention | Post-intervention | Pre-intervention | Post-intervention |
| **Primary outcome** |  |  |  |  |
| HbA1c, mmol/mol | 3·9 | 21·9 | 13·1 | 31·2 |
| **Secondary outcomes** |  |  |  |  |
| Systolic blood pressure, mmHg | 8·7 | 21·5 | 16·1 | 33·4 |
| Diastolic blood pressure, mmHg | 8·7 | 21·5 | 16·1 | 33·4 |
| Total cholesterol, mmol/L | 8·1 | 28·2 | 17·5 | 36·8 |
| Body weight, kg | 10·8 | 30·0 | 20·8 | 41·5 |
| **Exploratory outcomes** |  |  |  |  |
| LDL-cholesterol | 19·7 | 38·7 | 28·9 | 47·3 |
| HDL-cholesterol | 30·0 | 8·5 | 37·6 | 20·5 |
| Triglycerides | 11·6 | 29·8 | 18·7 | 37·3 |
| Creatinine | 5·1 | 22·1 | 14·0 | 31·7 |
| Urinary albumin creatinine ratio | 53·3 | 65·3 | 62·0 | 74·9 |
| Smoking, % | 1·4 |  | 0·5 |  |

MDW users were individually matched for age and sex with controls.
Data are % missing data in DMW users and controls not using DMW at pre-and post-intervention assessments.

Table e2.03: Multivariable-adjusted mean differences in post intervention primary and secondary outcomes between DMW users and matched controls not using DMW, after excluding participants who also used Changing Health and MyCognition interventions

| Outcomes | Mean difference | 95% CI | p-value |
| --- | --- | --- | --- |
| **Primary outcome** |  |  |  |
| HbA1c, mmol/mol | −3·30 | −4·82 to −1·80 | <0·001 |
| **Secondary outcomes** |  |  |  |
| Systolic blood pressure, mmHg | −1·40 | −2·65 to −0·15 | 0·029 |
| Diastolic blood pressure, mmHg | −1·09 | −1·99 to −0·18 | 0·019 |
| Total cholesterol, mmol/L | −0·12 | −0·21 to −0·02 | 0·019 |
| Body weight, kg | 0·01 | −0·86 to 0·88 | 0·98 |
| **Exploratory outcomes** |  |  |  |
| LDL-cholesterol | −0·10 | −0·18 to −0·03 | 0·007 |
| HDL-cholesterol | 0·002 | −0·02 to 0·02 | 0·837 |
| Triglycerides | 0·010 | −0·15 to 0·17 | 0·902 |
| Urinary albumin creatinine ratio | −2·63 | −6·54 to 1·27 | 0·172 |

MDW users were individually matched for age and sex with controls.
The analysis was performed after multiple imputation of missing data on outcomes and covariates.
Data are multivariable-adjusted differences in post-intervention outcome measures comparing DMW users and controls not using DMW.
Negative values indicate that levels are lower in DMW users than in controls.
All models are adjusted for age, sex, ethnicity, deprivation, baseline risk factors (HbA1c, systolic blood pressure, diastolic blood pressure, total, HDL and LDL cholesterol, triglycerides, UACR, smoking and body weight) and time from pre-intervention outcome assessment to post intervention outcome assessment.

Table e2.04: Multivariable-adjusted mean differences in primary and secondary outcomes between DMW users and matched controls not using DMW, using paired available outcome data

| Outcomes | N: DMW users; controls | Mean difference | 95% CI | p-value |
| --- | --- | --- | --- | --- |
| **Primary outcome** |  |  |  |  |
| HbA1c, mmol/mol | 391; 5545 | −2·70 | −4·96 to −0·44 | 0·019 |
| **Secondary outcomes** |  |  |  |  |
| Systolic blood pressure, mmHg | 368; 4752 | −2·45 | −4·35 to −0·55 | 0·012 |
| Total cholesterol, mmol/L | 346; 4453 | −0·11 | −0·24 to 0·02 | 0·104 |
| Body weight, kg | 327; 3698 | 0·36 | −0·61 to 1·32 | 0·468 |

In each of the four analyses, MDW users with paired data (pre- and post-intervention) were individually matched for age and sex with controls not using MDW who also had paired data.
Data are multivariable-adjusted differences in post-intervention outcome measures comparing DMW users and controls not using DMW.
Negative values indicate that levels are lower in DMW users than in controls.
All models are adjusted for age, sex, ethnicity, deprivation, levels of baseline risk factors (HbA1c, systolic blood pressure, diastolic blood pressure, total, HDL and LDL cholesterol, triglycerides, UACR, smoking and body weight, imputed if necessary) and time from pre-intervention outcome assessment to post intervention outcome assessment.

Table e2.05: Within-group changes in outcomes for test bed participants and matched controls who did not take up the intervention

|  | DMW users | | | | | | Controls not using DMW | | | | | |
| --- | --- | --- | --- | --- | --- | --- | --- | --- | --- | --- | --- | --- |
|  | Pre-intervention | | Post-intervention | | Within-person change (95% CI) (paired data) | p‑value | Pre- intervention | | Post- intervention | | Within-person change (95% CI) (paired data) | p‑value |
|  | N | Mean (SD) | N | Mean (SD) |  |  | N | Mean (SD) | N | Mean (SD) |  |  |
| ***Primary outcome*** |  |  |  |  |  |  |  |  |  |  |  |  |
| HbA1c, mmol/mol | 391 | 61·0 (17·4) | 391 | 58·5 (16·7) | -2·4 (-4·1, -0·8) | 0·004 | 5,545 | 59·9 (19·0) | 5,545 | 61·1 (19·0) | 1·2 (0·8, 1·6) | <0·001 |
| HbA1c, DCCT % | 391 | 7·7 (1·6) | 391 | 7·5 (1·6) | -0·2 (-0·4, -0·1) | 0·006 | 5,545 | 7·6 (1·8) | 5,545 | 7·7 (1·8) | 0·1 (0·1, 0·2) | <0·001 |
| HbA1c > 53 mmol/mol, % | 234 | 59·85 | 207 | 52·94 |  | <0·001 | 2,945 | 53·1 | 3,178 | 57·31 |  | <0·001 |
| ***Secondary outcomes*** |  |  |  |  |  |  |  |  |  |  |  |  |
| Systolic blood pressure, mmHg | 368 | 133·4 (15·7) | 368 | 130·2 (12·9) | -3·2 (-4·8, -1·6) | 0·001 | 4,752 | 130·7 (15·1) | 4,752 | 131·0 (15·2) | 0·3 (0·2, 0·8) | 0·193 |
| Diastolic blood pressure, mmHg | 368 | 79·7 (10·0) | 368 | 77·6 (8·6) | -2·1 (-3·2, -0·9) | <0·001 | 4,752 | 77·9 (9·7) | 4,752 | 77·7 (9·6) | -0·2 (-0·5, 0·1) | 0·194 |
| Total cholesterol, mmol/L | 346 | 4·5 (1·1) | 346 | 4·3 (1·1) | -0·2 (-0·3, -0·1) | 0·003 | 4,453 | 4·4 (1·2) | 4,453 | 4·3 (1·1) | -0·03 (-0·1, -0·01) | 0·017 |
| Body weight, Kg | 327 | 93·6 (21·3) | 327 | 92·5 (21·0) | -1·1 (-1·9, -0·4) | 0·004 | 3,698 | 89·9 (21·5) | 3,698 | 88·9 (21·5) | -1·0 (-1·3, -0·8) | <0·001 |
| ***Exploratory outcomes*** |  |  |  |  |  |  |  |  |  |  |  |  |
| LDL cholesterol, mmol/L | 284 | 2·3 (0·9) | 284 | 2·2 (0·9) | -0·11 (-0·20, -0·02) | 0·018 | 2,941 | 2·2 (0·9) | 2,941 | 2·2 (0·9) | 0·04 (0·01, 0·1) | 0·006 |
| HDL cholesterol, mmol/L | 339 | 1·2 (0·4) | 339 | 1·2 (0·3) | 0·01 (-0·01, 0·03) | 0·476 | 4,126 | 1·2 (0·3) | 4,126 | 1·2 (0·3) | −0·004 (−0·01, 0·002) | 0·155 |
| Triglycerides, mmol/L | 325 | 2·3 (1·7) | 325 | 2·3 (1·9) | -0·1 ( -0·2, 0·1) | 0·538 | 4,112 | 2·2 (1·5) | 4,112 | 2·2 (1·6) | -0·002 (-0·05, 0·04) | 0·912 |
| Urine albumin creatinine ratio, mg/mmol | 114 | 8·2 (22·0) | 114 | 7·4 (19·6) | -0·8 (-4·0, 2·4) | 0·631 | 455 | 6·9 (20·1) | 455 | 8·4 (24·3) | 1·45 ( -0·1, 2·97) | 0·063 |

Data are N, %, mean (SD) unless stated
Patients were individually matched with up to 20 controls on age, sex, ethnicity and GP postcode

*Negative values in the within-person change column indicate that values were lower post intervention than at baseline*

## Prescription/medication analysis

Descriptive summary and main analyses tables for the medication can be found in Table e2.06 – Table e2.10, broken down by each classification.

Table e2.06 describes the number and percentage of study participants who were observed to have had an increase, decrease, or no change in the number of prescriptions obtained in the two years pre and post the intervention date. This is repeated for each definition of prescriptions outlined in e1·3. For example, with respect to any prescription containing Metformin, for control subjects 29·2% were observed to have experienced no change in their prescription count, 43·3% increase, and 27·6% a decrease. Whereas for case subjects 23·7% remained the same, 37·5% increased, and 38·9% decreased.

Table e2.07 describes the average count for each definition of prescriptions (mean (SD), and median (IQR)) reported for the two years pre and post intervention, and within person difference, by intervention groups. The mean (SD) within person difference in anti-diabetic prescription count was 5·6 (28·7) and 1·6 (21·1) for control and case groups respectively.

Table e2.08 describes the Average Dose (mg per prescription) for each definition of prescriptions (mean (SD), and median (IQR)) for the two years pre and post intervention, and within person difference, by intervention groups. The mean (SD) within person difference in anti-diabetic prescription dose was 8·9 (118·4) and 10·3 (144·7) mgs for control and case groups respectively.

Table e2.09 describes the Total Costs (pence) of prescriptions for each definition of prescriptions (mean (SD), and median (IQR)) for the two years pre and post intervention, and within person difference, by intervention groups. The mean (SD) within person difference in anti-diabetic prescription costs was 8·041 (62,416) and 265 (144·7) pence for control and case groups respectively.

Table e2.10 Generalised linear model (Gamma) of prescription count, average dose, and total costs for the two years pre and post intervention. Results are reported in terms of a Rate Ratio (RR) with a 95% Confidence Interval 95%C.I.) comparing the rate within the intervention group against the controls. The rate of any anti-diabetic drug prescriptions post intervention were observed to be reduced by 17% (RR (95% C.I) = 0·83 (0·76,0·91)) in the intervention group compared to control. This corresponded to a 32% reduction in the costs (0·68 (0·57,0·81)), despite limited evidence of a slight increase (1·10 (0·90,1·37) in dose post intervention.

Table e2.06: Comparison of the change in number of prescriptions during 2 years pre- and post-intervention, by intervention group

|  | Change in No. Prescriptions (-/+2yrs) | | | Total |
| --- | --- | --- | --- | --- |
|  | Decrease | Same | Increase |  |
| Metformin-any |  |  |  |  |
| Control | 2794 (27·6%) | 2957 (29·2%) | 4389 (43·3%) | 10,140 |
| Intervention | 197 (38·9%) | 120 (23·7%) | 190 (37·5%) | 507 |
| Total | 2991 (28·1%) | 3077 (28·9%) | 4579 (43·0%) | 10,647 |
| Metformin – combination |  |  |  |  |
| Control | 2297 (22·7%) | 4207 (41·5%) | 3636 (35·9%) | 10,140 |
| Intervention | 168 (33·1%) | 168 (33·1%) | 171 (33·7%) | 507 |
| Total | 2465 (23·2%) | 4375 (41·1%) | 3807 (35·8%) | 10,647 |
| Metformin-only |  |  |  |  |
| Control | 852 (8·4%) | 7998 (78·9%) | 1290 (12·7%) | 10,140 |
| Intervention | 61 (12·0%) | 390 (76·9%) | 56 (11·0%) | 507 |
| Total | 913 (8·6%) | 8388 (78·8%) | 1346 (12·6%) | 10,647 |
| Sulphonylurea-any |  |  |  |  |
| Control | 872 (8·6%) | 8057 (79·5%) | 1211 (11·9%) | 10,140 |
| Intervention | 35 (6·9%) | 422 (83·2%) | 50 (9·9%) | 507 |
| Total | 907 (8·5%) | 8479 (79·6%) | 1261 (11·8%) | 10,647 |
| Sulphonylurea-combination |  |  |  |  |
| Control |  | 10140 (100·0%) |  |  |
| Intervention |  | 507 (100·0%) |  |  |
| Total |  | 10646 (100·0%) |  |  |
| Sulphonylurea -only |  |  |  |  |
| Control | 872 (8·6%) | 8057 (79·5%) | 1211 (11·9%) | 10,140 |
| Intervention | 35 (6·9%) | 422 (83·2%) | 50 (9·9%) | 507 |
| Total | 907 (8·5%) | 8479 (79·6%) | 1261 (11·8%) | 10,647 |
| Pioglitazone |  |  |  |  |
| Control | >122 (1·2%) | <9795 (96·6%) | >223 (2·2%) | 10,140 |
| Intervention | <10 (2·0%) | >487 (96·1%) | <10 (2·0%) | 507 |
| Total | 132 (1·2%) | 10282 (96·6%) | 233 (2·2%) | 10,647 |
| DPP4i - any |  |  |  |  |
| Control | 948 (9·3%) | 7781 (76·7%) | 1411 (13·9%) | 10,140 |
| Intervention | 46 (9·1%) | 402 (79·3%) | 59 (11·6%) | 507 |
| Total | 994 (9·3%) | 8183 (76·9%) | 1470 (13·8%) | 10,647 |
| DPP4i - combination |  |  |  |  |
| Control | >10 (0·1%) | >10094 (99·5%) | <36 (0·4%) | 10,140 |
| Intervention | <10 (2·0%) | >487 (96·1%) | >10 (2·0%) | 507 |
| Total | 20 (0·2%) | 10581 (99·4%) | 46 (0·4%) | 10,647 |
| DPP4i - only |  |  |  |  |
| Control | 930 (9·2%) | 7836 (77·3%) | 1374 (13·6%) | 10,140 |
| Intervention | 45 (8·9%) | 407 (80·3%) | 55 (10·8%) | 507 |
| Total | 975 (9·2%) | 8243 (77·4%) | 1429 (13·4%) | 10,647 |
| SGLT2i-any |  |  |  |  |
| Control | 1288 (12·7%) | 7417 (73·1%) | 1435 (14·2%) | 10,140 |
| Intervention | 112 (22·1%) | 330 (65·1%) | 65 (12·8%) | 507 |
| Total | 1400 (13·1%) | 7747 (72·8%) | 1500 (14·1%) | 10,647 |
| SGLT2i-combination |  |  |  |  |
| Control | 467 (4·6%) | 9118 (89·9%) | 555 (5·5%) | 10,140 |
| Intervention | 46 (9·1%) | 423 (83·4%) | 38 (7·5%) | 507 |
| Total | 513 (4·8%) | 9541 (89·6%) | 593 (5·6%) | 10,647 |
| SGLT2i-only |  |  |  |  |
| Control | 864 (8·5%) | 8346 (82·3%) | 930 (9·2%) | 10,140 |
| Intervention | 69 (13·6%) | 408 (80·5%) | 30 (5·9%) | 507 |
| Total | 933 (8·8%) | 8754 (82·2%) | 960 (9·0%) | 10,647 |
| GLP1RA-any |  |  |  |  |
| Control | 314 (3·1%) | 9402 (92·7%) | 424 (4·2%) | 10,140 |
| Intervention | 33 (6·5%) | 443 (87·4%) | 31 (6·1%) | 507 |
| Total | 347 (3·3%) | 9845 (92·5%) | 455 (4·3%) | 10,647 |
| GLP1RA-combination |  |  |  |  |
| Control | >15 (0·1%) | <10068 (99·3%) | >57 (0·6%) | 10,140 |
| Intervention | <10 (2·0%) | >487 (96·1%) | <10 (2·0%) | 507 |
| Total | 25 (0·2%) | 10555 (99·1%) | 67 (0·6%) | 10,647 |
| GLP1RA-only |  |  |  |  |
| Control | 296 (2·9%) | 9469 (93·4%) | 375 (3·7%) | 10,140 |
| Intervention | 33 (6·5%) | 446 (88·0%) | 28 (5·5%) | 507 |
| Total | 329 (3·1%) | 9915 (93·1%) | 403 (3·8%) | 10,647 |
| Insulin-any |  |  |  |  |
| Control | >15 (0·1%) | <10068 (99·3%) | >57 (0·6%) | 10,140 |
| Intervention | <10 (2·0%) | >487 (96·1%) | <10 (2·0%) | 507 |
| Total | 25 (0·2%) | 10555 (99·1%) | 67 (0·6%) | 10,647 |
| Statin-any |  |  |  |  |
| Control | 2894 (28·5%) | 3165 (31·2%) | 4081 (40·2%) | 10,140 |
| Intervention | 200 (39·4%) | 132 (26·0%) | 175 (34·5%) | 507 |
| Total | 3094 (29·1%) | 3297 (31·0%) | 4256 (40·0%) | 10,647 |
| Statin-combination |  |  |  |  |
| Control | 2895 (28·6%) | 3169 (31·3%) | 4076 (40·2%) | 10,140 |
| Intervention | 199 (39·3%) | 132 (26·0%) | 176 (34·7%) | 507 |
| Total | 3094 (29·1%) | 3301 (31·0%) | 4252 (39·9%) | 10,647 |
| Statin-only |  |  |  |  |
| Control | <10 (0·1%) | >10068 (99·3%) | <10 (0·1%) | 10,140 |
| Intervention | <10 (2·0%) | >487 (96·1%) | <10 (2·0%) | 507 |
| Total | 20 (0·2%) | 10555 (99·1%) | 20 (0·2%) | 10,647 |
| Fibrate-any |  |  |  |  |
| Control | 360 (3·6%) | 9382 (92·5%) | 398 (3·9%) | 10,140 |
| Intervention | 17 (3·4%) | 477 (94·1%) | 13 (2·6%) | 507 |
| Total | 377 (3·5%) | 9859 (92·6%) | 411 (3·9%) | 10,647 |
| Fibrate=combination |  |  |  |  |
| Control | >63 (0·6%) | <10068 (99·3%) | >67 (0·7%) | 10,140 |
| Intervention | <10 (2·0%) | >487 (96·1%) | <10 (2·0%) | 507 |
| Total | 73 (0·7%) | 10555 (99·1%) | 77 (0·7%) | 10,647 |
| Fibrate-only |  |  |  |  |
| Control | >304 (3·0%) | <9503 (93·7%) | >333 (3·3%) | 10,140 |
| Intervention | <10 (2·0%) | >487 (96·1%) | <10 (2·0%) | 507 |
| Total | 314 (2·9%) | 9990 (93·8%) | >343 (3·2%) | 10,647 |
| Ezetimibe-only |  |  |  |  |
| Control | 647 (6·4%) | 8358 (82·4%) | 1135 (11·2%) | 10,140 |
| Intervention | 33 (6·5%) | 447 (88·2%) | 27 (5·3%) | 507 |
| Total | 680 (6·4%) | 8805 (82·7%) | 1162 (10·9%) | 10,647 |
| Ezetimibe-only |  |  |  |  |
| Control | 647 (6·4%) | 8359 (82·4%) | 1134 (11·2%) | 10,140 |
| Intervention | 33 (6·5%) | 447 (88·2%) | 27 (5·3%) | 507 |
| Total | 680 (6·4%) | 8806 (82·7%) | 1161 (10·9%) | 10,647 |
| Ezetimibe-only |  |  |  |  |
| Control | - | 10140 (100%) | - | 10,140 |
| Intervention | - | 507 (100%) | - | 507 |
| Total | - | 10647 (100%) | - | 10,647 |
| Other Lipid Lowering |  |  |  |  |
| Control | >16 (0·2%) | <10100 (99·6%) | >24 (0·2%) | 10,140 |
| Intervention | <10 (2·0%) | >487 (96·1%) | <10 (2·0%) | 507 |
| Total | 26 (0·2%) | 10587 (99·4%) | 34 (0·3%) | 10,647 |
| Antihypertensive |  |  |  |  |
| Control | 2671 (26·3%) | 3650 (36·0%) | 3819 (37·7%) | 10,140 |
| Intervention | 158 (31·2%) | 174 (34·3%) | 175 (34·5%) | 507 |
| Total | 2829 (26·6%) | 3824 (35·9%) | 3994 (37·5%) | 10,647 |
| Anit-platelet |  |  |  |  |
| Control | 1119 (11·0%) | 7473 (73·7%) | 1548 (15·3%) | 10,140 |
| Intervention | 54 (10·7%) | 398 (78·5%) | 55 (10·8%) | 507 |
| Total | 1173 (11·0%) | 7871 (73·9%) | 1603 (15·1%) | 10,647 |
| Lipid-Lowering |  |  |  |  |
| Control | 2953 (29·1%) | 3055 (30·1%) | 4132 (40·7%) | 10,140 |
| Intervention | 201 (39·6%) | 131 (25·8%) | 175 (34·5%) | 507 |
| Total | 3154 (29·6%) | 3186 (29·9%) | 4307 (40·5%) | 10,647 |
| Anti-Diabetic |  |  |  |  |
| Control | 3188 (31·4%) | 2245 (22·1%) | 4707 (46·4%) | 10,140 |
| Intervention | 223 (44·0%) | 79 (15·6%) | 205 (40·4%) | 507 |
| Total | 3411 (32·0%) | 2324 (21·8%) | 4912 (46·1%) | 10,647 |

Table e2.07: Average count of prescriptions for the two years pre- and post-intervention, and within-person difference, by intervention groups.

|  | N | Two Years Pre-Intervention | | Two Years Post-Intervention | | Within-Person Difference | |
| --- | --- | --- | --- | --- | --- | --- | --- |
|  |  | Mean (SD) | Med (IQR) | Mean (SD) | Med (IQR) | Mean (SD) | Med (IQR) |
| **Anti-diabetic medicines** |  |  |  |  |  |  |  |
| Metformin-any |  |  |  |  |  |  |  |
| Control | 10,140 | 8·6 (11·9) | 5·0 (0·0; 13·0) | 11·6 (16·4) | 9·0 (0·0; 17·0) | 3·0 (15·4) | 0·0 (−1·0; 8·0) |
| Intervention | 507 | 10·6 (11·5) | 9·0 (1·0; 16·0) | 11·2 (13·8) | 9·0 (1·0; 17·0) | 0·6 (11·9) | 0·0 (−3·0; 5·0) |
| Metformin-comb |  |  |  |  |  |  |  |
| Control | 10,140 | 6·5 (10·7) | 2·0 (0·0; 10·0) | 8·8 (13·5) | 4·0 (0·0; 15·0) | 2·3 (12·0) | 0·0 (0·0; 5·0) |
| Intervention | 507 | 8·1 (10·8) | 4·0 (0·0; 14·0) | 8·7 (11·3) | 4·0 (0·0; 15·0) | 0·6 (9·7) | 0·0 (−3·0; 3·0) |
| Metformin-only |  |  |  |  |  |  |  |
| Control | 10,140 | 2·1 (6·3) | 0·0 (0·0; 0·0) | 2·8 (8·8) | 0·0 (0·0; 0·0) | 0·7 (7·3) | 0·0 (0·0; 0·0) |
| Intervention | 507 | 2·5 (6·6) | 0·0 (0·0; 0·0) | 2·5 (7·8) | 0·0 (0·0; 0·0) | 0·0 (5·9) | 0·0 (0·0; 0·0) |
| Sulphonylurea-any |  |  |  |  |  |  |  |
| Control | 10,140 | 2·1 (6·6) | 0·0 (0·0; 0·0) | 2·8 (8·2) | 0·0 (0·0; 0·0) | 0·7 (6·6) | 0·0 (0·0; 0·0) |
| Intervention | 507 | 1·6 (5·2) | 0·0 (0·0; 0·0) | 2·0 (6·5) | 0·0 (0·0; 0·0) | 0·4 (5·3) | 0·0 (0·0; 0·0) |
| Sulphonylurea-comb |  |  |  |  |  |  |  |
| Control | .. | .. | .. | .. | .. | .. | .. |
| Intervention | .. | .. | .. | .. | .. | .. | .. |
| Sulphonylurea-only |  |  |  |  |  |  |  |
| Control | 10,140 | 2·1 (6·6) | 0·0 (0·0; 0·0) | 2·8 (8·2) | 0·0 (0·0; 0·0) | 0·7 (6·6) | 0·0 (0·0; 0·0) |
| Intervention | 507 | 1·6 (5·2) | 0·0 (0·0; 0·0) | 2·0 (6·5) | 0·0 (0·0; 0·0) | 0·4 (5·3) | 0·0 (0·0; 0·0) |
| Pioglitazone |  |  |  |  |  |  |  |
| Control | 10,140 | 0·3 (2·3) | 0·0 (0·0; 0·0) | 0·5 (3·4) | 0·0 (0·0; 0·0) | 0·2 (2·7) | 0·0 (0·0; 0·0) |
| Intervention | 507 | 0·2 (1·4) | 0·0 (0·0; 0·0) | 0·2 (1·7) | 0·0 (0·0; 0·0) | 0·0 (1·6) | 0·0 (0·0; 0·0) |
| DPP4i-any |  |  |  |  |  |  |  |
| Control | 10,140 | 2·2 (6·2) | 0·0 (0·0; 0·0) | 3·0 (9·1) | 0·0 (0·0; 0·0) | 0·8 (8·0) | 0·0 (0·0; 0·0) |
| Intervention | 507 | 1·8 (5·0) | 0·0 (0·0; 0·0) | 2·3 (6·6) | 0·0 (0·0; 0·0) | 0·5 (4·9) | 0·0 (0·0; 0·0) |
| DPP4i-comb |  |  |  |  |  |  |  |
| Control | 10,140 | 0·1 (0·8) | 0·0 (0·0; 0·0) | 0·1 (1·1) | 0·0 (0·0; 0·0) | 0·0 (0·8) | 0·0 (0·0; 0·0) |
| Intervention | 507 | 0·1 (1·1) | 0·0 (0·0; 0·0) | 0·1 (1·3) | 0·0 (0·0; 0·0) | 0·0 (0·5) | 0·0 (0·0; 0·0) |
| DPP4i-only |  |  |  |  |  |  |  |
| Control | 10,140 | 2·1 (6·1) | 0·0 (0·0; 0·0) | 2·9 (9·0) | 0·0 (0·0; 0·0) | 0·8 (8·0) | 0·0 (0·0; 0·0) |
| Intervention | 507 | 1·7 (4·9) | 0·0 (0·0; 0·0) | 2·2 (6·5) | 0·0 (0·0; 0·0) | 0·4 (4·9) | 0·0 (0·0; 0·0) |
| SGLT2i-any |  |  |  |  |  |  |  |
| Control | 10,140 | 2·4 (6·1) | 0·0 (0·0; 0·0) | 3·0 (9·0) | 0·0 (0·0; 0·0) | 0·6 (7·9) | 0·0 (0·0; 0·0) |
| Intervention | 507 | 3·8 (7·4) | 0·0 (0·0; 5·0) | 3·3 (8·0) | 0·0 (0·0; 1·0) | −0·5 (6·5) | 0·0 (0·0; 0·0) |
| SGLT2i-comb |  |  |  |  |  |  |  |
| Control | 10,140 | 0·9 (4·1) | 0·0 (0·0; 0·0) | 1·1 (6·5) | 0·0 (0·0; 0·0) | 0·2 (5·9) | 0·0 (0·0; 0·0) |
| Intervention | 507 | 1·6 (4·7) | 0·0 (0·0; 0·0) | 1·6 (6·1) | 0·0 (0·0; 0·0) | 0·0 (5·0) | 0·0 (0·0; 0·0) |
| SGLT2i-only |  |  |  |  |  |  |  |
| Control | 10,140 | 1·5 (4·8) | 0·0 (0·0; 0·0) | 1·9 (6·5) | 0·0 (0·0; 0·0) | 0·3 (5·4) | 0·0 (0·0; 0·0) |
| Intervention | 507 | 2·3 (6·3) | 0·0 (0·0; 0·0) | 1·7 (5·7) | 0·0 (0·0; 0·0) | −0·5 (4·2) | 0·0 (0·0; 0·0) |
| GLP1RA-any |  |  |  |  |  |  |  |
| Control | 10,140 | 0·6 (2·8) | 0·0 (0·0; 0·0) | 0·7 (3·5) | 0·0 (0·0; 0·0) | 0·2 (2·9) | 0·0 (0·0; 0·0) |
| Intervention | 507 | 1·0 (3·7) | 0·0 (0·0; 0·0) | 1·0 (4·1) | 0·0 (0·0; 0·0) | 0·0 (3·6) | 0·0 (0·0; 0·0) |
| GLP1RA-comb |  |  |  |  |  |  |  |
| Control | 10,140 | 0·1 (0·8) | 0·0 (0·0; 0·0) | 0·1 (1·2) | 0·0 (0·0; 0·0) | 0·0 (0·9) | 0·0 (0·0; 0·0) |
| Intervention | 507 |  | 0·0 (0·0; 0·0) |  | 0·0 (0·0; 0·0) |  | 0·0 (0·0; 0·0) |
| GLP1RA-only |  |  |  |  |  |  |  |
| Control | 10,140 | 0·5 (2·7) | 0·0 (0·0; 0·0) | 0·7 (3·3) | 0·0 (0·0; 0·0) | 0·1 (2·7) | 0·0 (0·0; 0·0) |
| Intervention | 507 | 1·0 (3·7) | 0·0 (0·0; 0·0) | 0·9 (3·7) | 0·0 (0·0; 0·0) | −0·1 (3·3) | 0·0 (0·0; 0·0) |
| Insulin |  |  |  |  |  |  |  |
| Control | 10,140 | 0·1 (0·8) | 0·0 (0·0; 0·0) | 0·1 (1·2) | 0·0 (0·0; 0·0) | 0·0 (0·9) | 0·0 (0·0; 0·0) |
| Intervention | 507 | 0·0 (0·6) | 0·0 (0·0; 0·0) | 0·1 (1·3) | 0·0 (0·0; 0·0) | 0·1 (0·9) | 0·0 (0·0; 0·0) |
| *Total anti-diabetic* |  |  |  |  |  |  |  |
| *Control* | *10,140* | *16·7 (22·3)* | *10·0 (1·0; 23·0)* | *22·4 (30·8)* | *15·0 (2·0; 31·0)* | *5·6 (28·7)* | *0·0 (−3·0; 13·0)* |
| *Intervention* | *507* | *19·1 (20·8)* | *15·0 (4·0; 27·0)* | *20·7 (26·0)* | *15·0 (2·0; 30·0)* | *1·6 (21·1)* | *0·0 (−7·0; 8·0)* |
| **Lipid-lowering medicines** |  |  |  |  |  |  |  |
| Statin-any |  |  |  |  |  |  |  |
| Control | 10,140 | 8·0 (10·8) | 5·0 (0·0; 12·0) | 10·3 (13·3) | 8·0 (0·0; 16·0) | 2·3 (12·2) | 0·0 (−1·0; 6·0) |
| Intervention | 507 | 9·1 (10·4) | 7·0 (0·0; 15·0) | 9·5 (11·0) | 7·0 (0·0; 16·0) | 0·4 (9·4) | 0·0 (−3·0; 4·0) |
| Statin-comb |  |  |  |  |  |  |  |
| Control | 10,140 | 8·0 (10·8) | 5·0 (0·0; 12·0) | 10·3 (13·3) | 8·0 (0·0; 16·0) | 2·3 (12·2) | 0·0 (−1·0; 6·0) |
| Intervention | 507 | 9·1 (10·4) | 7·0 (0·0; 15·0) | 9·5 (11·0) | 7·0 (0·0; 16·0) | 0·4 (9·4) | 0·0 (−3·0; 4·0) |
| Statin-only |  |  |  |  |  |  |  |
| Control | 10,140 | 0·0 (0·2) | 0·0 (0·0; 0·0) | 0·0 (0·4) | 0·0 (0·0; 0·0) | 0·0 (0·2) | 0·0 (0·0; 0·0) |
| Intervention | 507 | 0·0 (0·5) | 0·0 (0·0; 0·0) | 0·0 (0·0) | 0·0 (0·0; 0·0) | 0·0 (0·5) | 0·0 (0·0; 0·0) |
| Fibrate-any |  |  |  |  |  |  |  |
| Control | 10,140 | 0·9 (4·8) | 0·0 (0·0; 0·0) | 1·0 (5·0) | 0·0 (0·0; 0·0) | 0·1 (4·1) | 0·0 (0·0; 0·0) |
| Intervention | 507 | 0·8 (4·7) | 0·0 (0·0; 0·0) | 0·8 (4·4) | 0·0 (0·0; 0·0) | 0·0 (3·2) | 0·0 (0·0; 0·0) |
| Fibrate-comb |  |  |  |  |  |  |  |
| Control | 10,140 | 0·2 (2·1) | 0·0 (0·0; 0·0) | 0·2 (1·9) | 0·0 (0·0; 0·0) | 0·0 (1·6) | 0·0 (0·0; 0·0) |
| Intervention | 507 | 0·2 (1·8) | 0·0 (0·0; 0·0) | 0·2 (1·9) | 0·0 (0·0; 0·0) | 0·0 (1·3) | 0·0 (0·0; 0·0) |
| Fibrate-only |  |  |  |  |  |  |  |
| Control | 10,140 | 0·7 (4·1) | 0·0 (0·0; 0·0) | 0·8 (4·6) | 0·0 (0·0; 0·0) | 0·1 (3·6) | 0·0 (0·0; 0·0) |
| Intervention | 507 | 0·6 (4·2) | 0·0 (0·0; 0·0) | 0·5 (4·0) | 0·0 (0·0; 0·0) | −0·1 (2·6) | 0·0 (0·0; 0·0) |
| Ezetimibe-only |  |  |  |  |  |  |  |
| Control | 10,140 | 1·9 (6·0) | 0·0 (0·0; 0·0) | 2·6 (7·9) | 0·0 (0·0; 0·0) | 0·7 (5·8) | 0·0 (0·0; 0·0) |
| Intervention | 507 | 1·5 (5·9) | 0·0 (0·0; 0·0) | 1·4 (5·1) | 0·0 (0·0; 0·0) | 0·0 (4·0) | 0·0 (0·0; 0·0) |
| Ezetimibe-only |  |  |  |  |  |  |  |
| Control | 10,140 | 1·9 (6·0) | 0·0 (0·0; 0·0) | 2·6 (7·9) | 0·0 (0·0; 0·0) | 0·7 (5·8) | 0·0 (0·0; 0·0) |
| Intervention | 507 | 1·5 (5·9) | 0·0 (0·0; 0·0) | 1·4 (5·1) | 0·0 (0·0; 0·0) | 0·0 (4·0) | 0·0 (0·0; 0·0) |
| Ezetimibe-only |  |  |  |  |  |  |  |
| Control | 10,140 | 0·0 (0·3) | 0·0 (0·0; 0·0) | 0·0 (1·0) | 0·0 (0·0; 0·0) | 0·0 (0·7) | 0·0 (0·0; 0·0) |
| Intervention | 507 |  | 0·0 (0·0; 0·0) |  | 0·0 (0·0; 0·0) |  | 0·0 (0·0; 0·0) |
| Other lipid-lowering |  |  |  |  |  |  |  |
| Control | 10,140 | 9·0 (13·2) | 6·0 (0·0; 13·0) | 11·5 (15·5) | 8·0 (0·0; 17·0) | 2·5 (13·9) | 0·0 (−1·0; 7·0) |
| Intervention | 507 | 10·0 (12·4) | 7·0 (1·0; 15·0) | 10·3 (12·7) | 8·0 (0·0; 16·0) | 0·3 (10·6) | 0·0 (−4·0; 4·0) |
| *Total lipid-lowering* |  |  |  |  |  |  |  |
| *Control* | *10,140* | *9·0 (13·2)* | *6·0 (0·0; 13·0)* | *11·5 (15·5)* | *8·0 (0·0; 17·0)* | *2·5 (13·9)* | *0·0 (−1·0; 7·0)* |
| *Intervention* | *507* | *10·0 (12·4)* | *7·0 (1·0; 15·0)* | *10·3 (12·7)* | *8·0 (0·0; 16·0)* | *0·3 (10·6)* | *0·0 (−4·0; 4·0)* |
| Antihypertensive medicines |  |  |  |  |  |  |  |
| Control | 10,140 | 15·7 (28·5) | 6·0 (0·0; 20·0) | 20·2 (33·9) | 10·0 (0·0; 28·0) | 4·5 (28·7) | 0·0 (−1·0; 9·0) |
| Intervention | 507 | 18·4 (33·2) | 9·0 (0·0; 26·0) | 20·3 (31·7) | 12·0 (0·0; 27·0) | 1·9 (20·9) | 0·0 (−3·0; 7·0) |
| Antiplatelet medicines |  |  |  |  |  |  |  |
| Control | 10,140 | 4·3 (11·9) | 0·0 (0·0; 0·0) | 5·6 (16·8) | 0·0 (0·0; 0·0) | 1·3 (14·0) | 0·0 (0·0; 0·0) |
| Intervention | 507 | 4·0 (16·2) | 0·0 (0·0; 0·0) | 4·5 (16·3) | 0·0 (0·0; 0·0) | 0·5 (9·6) | 0·0 (0·0; 0·0) |

Table e2.08: Average dose (mg per prescription) of prescriptions for the two years pre and post intervention, and within person difference, by intervention groups

|  | Two Years Pre-Intervention | | | Two Years Post-Intervention | | | Within-Person Difference | | |
| --- | --- | --- | --- | --- | --- | --- | --- | --- | --- |
|  | N | Mean (SD) | Med (IQR) | N | Mean (SD) | Med (IQR) | N | Mean (SD) | Med (IQR) |
| **Antidiabetic medicines** |  |  |  |  |  |  |  |  |  |
| Metformin-any |  |  |  |  |  |  |  |  |  |
| Control | 6,946 | 548·5 (230·3) | 500·0 (500·0; 500·0) | 7,125 | 548·6 (221·8) | 500·0 (500·0; 500·0) | 6,583 | −2·0 (101·4) | 0·0 (0·0; 0·0) |
| Intervention | 382 | 515·7 (248·8) | 500·0 (500·0; 500·0) | 383 | 504·0 (221·2) | 500·0 (500·0; 500·0) | 358 | −7·7 (160·4) | 0·0 (0·0; 0·0) |
| Metformin-combination |  |  |  |  |  |  |  |  |  |
| Control | 5,476 | 473·9 (217·8) | 500·0 (500·0; 500·0) | 5,758 | 479·8 (208·7) | 500·0 (500·0; 500·0) | 5,060 | 1·6 (92·5) | 0·0 (0·0; 0·0) |
| Intervention | 313 | 450·6 (250·9) | 500·0 (313·0; 500·0) | 332 | 454·3 (217·4) | 500·0 (500·0; 500·0) | 288 | 0·1 (157·2) | 0·0 (0·0; 0·0) |
| Metformin-only |  |  |  |  |  |  |  |  |  |
| Control | 1,941 | 747·5 (245·3) | 750·0 (500·0; 1000·0) | 2,023 | 744·1 (243·8) | 714·0 (500·0; 1000·0) | 1,734 | −3·6 (74·6) | 0·0 (0·0; 0·0) |
| Intervention | 106 | 725·9 (247·1) | 500·0 (500·0; 1000·0) | 103 | 718·4 (244·6) | 500·0 (500·0; 1000·0) | 86 | 8·7 (95·8) | 0·0 (0·0; 0·0) |
| Sulphonylurea-any |  |  |  |  |  |  |  |  |  |
| Control | 1,822 | 57·9 (28·3) | 80·0 (40·0; 80·0) | 1,907 | 56·8 (29·5) | 80·0 (40·0; 80·0) | 1,575 | −0·7 (9·0) | 0·0 (0·0; 0·0) |
| Intervention | 75 | 64·1 (27·8) | 80·0 (40·0; 80·0) | 81 | 59·9 (26·6) | 80·0 (40·0; 80·0) | 65 | −0·7 (12·4) | 0·0 (0·0; 0·0) |
| Pioglitazone |  |  |  |  |  |  |  |  |  |
| Control | 304 | 31·0 (11·9) | 30·0 (15·0; 45·0) | >328 | 31·1 (11·7) | 30·0 (15·0; 45·0) | >263 | −0·8 (4·3) | 0·0 (0·0; 0·0) |
| Intervention | 10 | 25·5 (10·1) | 30·0 (15·0; 30·0) | <10 | 27·5 (10·7) | 30·0 (15·0; 30·0) | <10 | −3·1 (5·8) | 0·0 (−7·0; 0·0) |
| DPP4i-any |  |  |  |  |  |  |  |  |  |
| Control | 2,113 | 39·8 (56·7) | 25·0 (12·0; 50·0) | 2,113 | 39·8 (56·7) | 25·0 (12·0; 50·0) | 1,715 | 0·9 (28·1) | 0·0 (0·0; 0·0) |
| Intervention | 92 | 36·7 (91·5) | 14·0 (5·0; 25·0) | 92 | 36·7 (91·5) | 14·0 (5·0; 25·0) | 71 | −0·4 (14·6) | 0·0 (0·0; 0·0) |
| DPP4i-combination |  |  |  |  |  |  |  |  |  |
| Control | >54 | 4·4 (1·2) | 5·0 (5·0; 5·0) | >54 | 4·4 (1·2) | 5·0 (5·0; 5·0) | >40 | 0·0 (0·2) | 0·0 (0·0; 0·0) |
| Intervention | <10 | 4·4 (1·3) | 5·0 (5·0; 5·0) | <10 | 4·4 (1·3) | 5·0 (5·0; 5·0) | <10 | 0·0 (0·0) | 0·0 (0·0; 0·0) |
| DPP4i-only |  |  |  |  |  |  |  |  |  |
| Control | 2,060 | 40·7 (57·1) | 25·0 (12·0; 50·0) | 2,060 | 40·7 (57·1) | 25·0 (12·0; 50·0) | 1,669 | 0·9 (28·4) | 0·0 (0·0; 0·0) |
| Intervention | 87 | 38·6 (93·8) | 25·0 (5·0; 25·0) | 87 | 38·6 (93·8) | 25·0 (5·0; 25·0) | 67 | −0·4 (15·0) | 0·0 (0·0; 0·0) |
| SGLT2i-any |  |  |  |  |  |  |  |  |  |
| Control | 2,441 | 81·8 (107·1) | 25·0 (10·0; 100·0) | 2,238 | 75·8 (100·6) | 21·0 (10·0; 100·0) | 1,882 | −10·8 (42·1) | 0·0 (0·0; 0·0) |
| Intervention | 170 | 77·1 (99·6) | 25·0 (10·0; 100·0) | 137 | 69·9 (85·8) | 25·0 (10·0; 100·0) | 122 | −16·3 (46·3) | 0·0 (−8·0; 0·0) |
| SGLT2i-combination |  |  |  |  |  |  |  |  |  |
| Control | 879 | 15·9 (7·6) | 11·0 (10·0; 25·0) | 836 | 15·2 (7·3) | 10·0 (10·0; 25·0) | 672 | −1·6 (3·8) | 0·0 (−1·0; 0·0) |
| Intervention | 76 | 16·3 (7·6) | 12·0 (10·0; 25·0) | 63 | 14·6 (7·4) | 10·0 (10·0; 25·0) | 54 | −2·2 (4·6) | 0·0 (−2·0; 0·0) |
| SGLT2i-only |  |  |  |  |  |  |  |  |  |
| Control | 1,601 | 116·6 (118·4) | 100·0 (10·0; 275·0) | 1,447 | 110·5 (112·6) | 100·0 (10·0; 204·0) | 1,202 | −15·6 (47·1) | 0·0 (0·0; 0·0) |
| Intervention | 98 | 122·9 (113·0) | 100·0 (10·0; 240·0) | 78 | 116·1 (97·0) | 100·0 (10·0; 171·0) | 70 | −27·7 (58·0) | 0·0 (−40·0; 0·0) |
| GLP1RA-any |  |  |  |  |  |  |  |  |  |
| Control | 552 | 3·9 (8·8) | 2·0 (1·0; 6·0) | 552 | 4·6 (10·4) | 6·0 (1·0; 6·0) | 413 | 0·0 (1·1) | 0·0 (0·0; 0·0) |
| Intervention | 53 | 2·5 (2·4) | 1·0 (1·0; 6·0) | 46 | 3·3 (2·7) | 3·0 (1·0; 6·0) | 36 | 0·0 (1·1) | 0·0 (0·0; 0·0) |
| GLP1RA-combination |  |  |  |  |  |  |  |  |  |
| Control | .. | .. | .. | .. | .. | .. | .. | .. | .. |
| Intervention | <10 | .. | .. | <10 | .. | .. | <10 | .. | .. |
| GLP1RA-only |  |  |  |  |  |  |  |  |  |
| Control | 547 | 3·1 (2·5) | 2·0 (1·0; 6·0) | 546 | 3·5 (2·6) | 6·0 (1·0; 6·0) | 409 | 0·0 (0·9) | 0·0 (0·0; 0·0) |
| Intervention | 53 | 2·5 (2·4) | 1·0 (1·0; 6·0) | 46 | 3·3 (2·7) | 3·0 (1·0; 6·0) | 36 | 0·0 (1·1) | 0·0 (0·0; 0·0) |
| Insulin |  |  |  |  |  |  |  |  |  |
| Control | .. | .. | .. | .. | .. | .. | .. | .. | .. |
| Intervention | <10 | .. | .. | <10 | .. | .. | <10 | .. | .. |
| *Total antidiabetic* |  |  |  |  |  |  |  |  |  |
| *Control* | *7,421* | *386·0 (254·2)* | *400·0 (239·0; 500·0)* | *7,530* | *393·4 (236·2)* | *437·0 (251·0; 500·0)* | *7,064* | *8·9 (118·4)* | *0·0 (0·0; 11·0)* |
| *Intervention* | *401* | *384·4 (216·3)* | *423·0 (229·0; 500·0)* | *400* | *389·6 (206·7)* | *500·0 (253·0; 500·0)* | *379* | *10·3 (144·7)* | *0·0 (−5·0; 38·0)* |
| **Lipid-lowering medicines** |  |  |  |  |  |  |  |  |  |
| Statin-any |  |  |  |  |  |  |  |  |  |
| Control | 6,804 | 33·7 (23·8) | 20·0 (20·0; 40·0) | 6,866 | 33·4 (23·3) | 20·0 (20·0; 40·0) | 6,371 | −0·7 (7·9) | 0·0 (0·0; 0·0) |
| Intervention | 379 | 32·7 (22·6) | 20·0 (20·0; 40·0) | 354 | 33·0 (23·7) | 20·0 (20·0; 40·0) | 339 | −1·3 (8·5) | 0·0 (0·0; 0·0) |
| Statin-combination |  |  |  |  |  |  |  |  |  |
| Control | 6,800 | 33·7 (23·8) | 20·0 (20·0; 40·0) | 6,862 | 33·4 (23·3) | 20·0 (20·0; 40·0) | 6,367 | −0·7 (7·9) | 0·0 (0·0; 0·0) |
| Intervention | 379 | 32·7 (22·6) | 20·0 (20·0; 40·0) | 354 | 33·0 (23·7) | 20·0 (20·0; 40·0) | 339 | −1·3 (8·5) | 0·0 (0·0; 0·0) |
| Statin-only |  |  |  |  |  |  |  |  |  |
| Control | .. | .. | .. | .. | .. | .. | .. | .. | .. |
| Intervention | <10 | .. | .. | <10 | .. | .. | <10 | .. | .. |
| Fibrate-any |  |  |  |  |  |  |  |  |  |
| Control | 696 | 103·2 (68·7) | 75·0 (75·0; 75·0) | 654 | 104·5 (69·5) | 75·0 (75·0; 75·0) | 570 | 0·1 (16·7) | 0·0 (0·0; 0·0) |
| Intervention | 29 | 115·1 (69·5) | 75·0 (75·0; 160·0) | 25 | 125·1 (86·1) | 75·0 (75·0; 160·0) | 23 | 5·6 (26·8) | 0·0 (0·0; 0·0) |
| Fibrate-combination |  |  |  |  |  |  |  |  |  |
| Control | >132 | 220·1 (78·9) | 200·0 (160·0; 267·0) | >124 | 226·2 (80·3) | 200·0 (160·0; 267·0) | >110 | 0·9 (10·2) | 0·0 (0·0; 0·0) |
| Intervention | <10 | 236·7 (75·4) | 200·0 (200·0; 267·0) | <10 | 231·7 (79·4) | 200·0 (180·0; 267·0) | <10 | 0·0 (0·0) | 0·0 (0·0; 0·0) |
| Fibrate-only |  |  |  |  |  |  |  |  |  |
| Control | 577 | 77·6 (29·9) | 75·0 (75·0; 75·0) | 539 | 77·3 (22·4) | 75·0 (75·0; 75·0) | 463 | 0·0 (0·0) | 0·0 (0·0; 0·0) |
| Intervention | 22 | 75·0 (0·0) | 75·0 (75·0; 75·0) | 17 | 75·0 (0·0) | 75·0 (75·0; 75·0) | 16 | 0·0 (0·0) | 0·0 (0·0; 0·0) |
| Ezetimibe-only |  |  |  |  |  |  |  |  |  |
| Control | 1,582 | 30·8 (14·7) | 40·0 (20·0; 40·0) | 1,787 | 31·2 (14·3) | 40·0 (20·0; 40·0) | 1,506 | −0·1 (2·8) | 0·0 (0·0; 0·0) |
| Intervention | 59 | 31·1 (13·7) | 40·0 (20·0; 40·0) | 60 | 31·7 (12·3) | 40·0 (20·0; 40·0) | 54 | −0·5 (3·8) | 0·0 (0·0; 0·0) |
| Ezetimibe-only |  |  |  |  |  |  |  |  |  |
| Control | 1,581 | 30·7 (14·7) | 40·0 (20·0; 40·0) | 1,786 | 31·2 (14·3) | 40·0 (20·0; 40·0) | 1,505 | −0·1 (2·8) | 0·0 (0·0; 0·0) |
| Intervention | 59 | 31·0 (13·7) | 40·0 (20·0; 40·0) | 60 | 31·7 (12·3) | 40·0 (20·0; 40·0) | 54 | −0·5 (3·8) | 0·0 (0·0; 0·0) |
| Ezetimibe-only |  |  |  |  |  |  |  |  |  |
| Control | .. | .. | .. |  | .. | .. | .. | .. | .. |
| Intervention | <10 | .. | .. | <10 | .. | .. | <10 | .. | .. |
| Other lipid-lowering |  |  |  |  |  |  |  |  |  |
| Control | 6,905 | 39·3 (99·5) | 25·0 (20·0; 40·0) | 6,957 | 39·2 (92·6) | 26·0 (20·0; 40·0) | 6,463 | −0·1 (49·7) | 0·0 (0·0; 0·0) |
| Intervention | 385 | 37·1 (54·5) | 20·0 (20·0; 40·0) | 361 | 47·0 (213·4) | 20·0 (20·0; 40·0) | 350 | 9·0 (215·9) | 0·0 (0·0; 0·0) |
| *Total lipid-lowering* |  |  |  |  |  |  |  |  |  |
| *Control* | *6,905* | *39·3 (99·5)* | *25·0 (20·0; 40·0)* | *6,957* | *39·1 (92·6)* | *26·0 (20·0; 40·0)* | *6,463* | *−0·1 (49·7)* | *0·0 (0·0; 0·0)* |
| *Intervention* | *385* | *37·1 (54·5)* | *20·0 (20·0; 40·0)* | *361* | *46·9 (213·4)* | *20·0 (20·0; 40·0)* | *350* | *9·0 (215·9)* | *0·0 (0·0; 0·0)* |
| Antihypertensive |  |  |  |  |  |  |  |  |  |
| Control | 6,241 | 19·7 (37·4) | 8·0 (4·0; 19·0) | 6,319 | 20·4 (38·5) | 8·0 (4·0; 20·0) | 5,883 | 0·2 (11·2) | 0·0 (0·0; 0·0) |
| Intervention | 330 | 20·7 (33·6) | 10·0 (5·0; 20·0) | 323 | 21·2 (37·4) | 9·0 (5·0; 19·0) | 309 | 0·6 (16·6) | 0·0 (0·0; 0·0) |
| Antiplatelet |  |  |  |  |  |  |  |  |  |
| Control | 2,473 | 53·8 (50·7) | 52·0 (36·0; 75·0) | 2,526 | 56·2 (57·4) | 57·0 (38·0; 75·0) | 2,235 | 0·6 (13·4) | 0·0 (0·0; 0·0) |
| Intervention | 104 | 55·6 (53·0) | 75·0 (37·0; 75·0) | 96 | 55·9 (53·6) | 75·0 (38·0; 75·0) | 87 | 1·7 (6·6) | 0·0 (0·0; 0·0) |

Table e2.09: Total Costs (GBP) of antidiabetic prescriptions for the two years pre- and post-intervention, and within-person difference, by intervention group

|  | N | Two Years Pre-Intervention | | Two Years Post-Intervention | | Within-Person Difference | |
| --- | --- | --- | --- | --- | --- | --- | --- |
|  |  | Mean (SD) | Med (IQR) | Mean (SD) | Med (IQR) | Mean (SD) | Med (IQR) |
| Metformin – any |  |  |  |  |  |  |  |
| Control | 10140 | 71·21 (195·99) | 17·36 (0·00; 45·92) | 91·96 (288·40) | 28·08 (0·00; 59·04) | 20·75 (259·30) | 0·00 (−4·92; 26·04) |
| Intervention | 507 | 107·33 (209·32) | 26·24 (1·56; 58·22) | 106·13 (258·60) | 21·70 (1·56; 59·04) | −1·20 (225·24) | 0·00 (−16·40; 12·30) |
| Metformin – comb |  |  |  |  |  |  |  |
| Control | 10140 | 65·45 (193·96) | 4·92 (0·00; 39·36) | 84·18 (281·17) | 12·30 (0·00; 52·48) | 18·73 (251·30) | 0·00 (0·00; 15·26) |
| Intervention | 507 | 101·69 (209·13) | 12·30 (0·00; 54·94) | 100·43 (253·59) | 13·12 (0·00; 55·76) | −1·25 (220·91) | 0·00 (−11·48; 8·20) |
| Metformin – only |  |  |  |  |  |  |  |
| Control | 10140 | 5·76 (15·94) | 0·00 (0·00; 0·00) | 7·78 (22·33) | 0·00 (0·00; 0·00) | 2·02 (18·66) | 0·00 (0·00; 0·00) |
| Intervention | 507 | 5·65 (14·17) | 0·00 (0·00; 0·00) | 5·70 (16·78) | 0·00 (0·00; 0·00) | 0·05 (13·53) | 0·00 (0·00; 0·00) |
| Sulphonylurea – any |  |  |  |  |  |  |  |
| Control | 10140 | 5·33 (16·90) | 0·00 (0·00; 0·00) | 7·14 (20·83) | 0·00 (0·00; 0·00) | 1·81 (16·38) | 0·00 (0·00; 0·00) |
| Intervention | 507 | 4·01 (13·99) | 0·00 (0·00; 0·00) | 5·02 (16·35) | 0·00 (0·00; 0·00) | 1·02 (12·66) | 0·00 (0·00; 0·00) |
| Pioglitazone |  |  |  |  |  |  |  |
| Control | 10140 | 1·19 (18·28) | 0·00 (0·00; 0·00) | 2·07 (31·71) | 0·00 (0·00; 0·00) | 0·88 (23·09) | 0·00 (0·00; 0·00) |
| Intervention | 507 | 0·32 (2·76) | 0·00 (0·00; 0·00) | 0·41 (3·81) | 0·00 (0·00; 0·00) | 0·09 (3·64) | 0·00 (0·00; 0·00) |
| DPP4i – any |  |  |  |  |  |  |  |
| Control | 10140 | 76·60 (206·38) | 0·00 (0·00; 0·00) | 105·96 (304·82) | 0·00 (0·00; 0·00) | 29·36 (266·87) | 0·00 (0·00; 0·00) |
| Intervention | 507 | 59·37 (157·92) | 0·00 (0·00; 0·00) | 80·58 (225·28) | 0·00 (0·00; 0·00) | 21·20 (170·83) | 0·00 (0·00; 0·00) |
| DPP4i – comb |  |  |  |  |  |  |  |
| Control | 10140 | 2·27 (36·76) | 0·00 (0·00; 0·00) | 3·20 (48·16) | 0·00 (0·00; 0·00) | 0·93 (33·97) | 0·00 (0·00; 0·00) |
| Intervention | 507 | 3·61 (42·13) | 0·00 (0·00; 0·00) | 5·92 (60·83) | 0·00 (0·00; 0·00) | 2·31 (33·43) | 0·00 (0·00; 0·00) |
| DPP4i – only |  |  |  |  |  |  |  |
| Control | 10140 | 74·33 (203·85) | 0·00 (0·00; 0·00) | 102·76 (301·97) | 0·00 (0·00; 0·00) | 28·43 (264·72) | 0·00 (0·00; 0·00) |
| Intervention | 507 | 55·76 (153·52) | 0·00 (0·00; 0·00) | 74·66 (218·94) | 0·00 (0·00; 0·00) | 18·90 (167·79) | 0·00 (0·00; 0·00) |
| SGLT2i – any |  |  |  |  |  |  |  |
| Control | 10140 | 107·33 (257·74) | 0·00 (0·00; 0·00) | 132·43 (363·37) | 0·00 (0·00; 0·00) | 25·11 (321·64) | 0·00 (0·00; 0·00) |
| Intervention | 507 | 159·18 (295·58) | 0·00 (0·00; 219·54) | 142·64 (330·04) | 0·00 (0·00; 39·20) | −16·54 (273·92) | 0·00 (0·00; 0·00) |
| SGLT2i – comb |  |  |  |  |  |  |  |
| Control | 10140 | 39·11 (171·34) | 0·00 (0·00; 0·00) | 48·51 (255·66) | 0·00 (0·00; 0·00) | 9·41 (230·18) | 0·00 (0·00; 0·00) |
| Intervention | 507 | 64·38 (183·44) | 0·00 (0·00; 0·00) | 66·18 (238·19) | 0·00 (0·00; 0·00) | 1·80 (198·86) | 0·00 (0·00; 0·00) |
| SGLT2i – only |  |  |  |  |  |  |  |
| Control | 10140 | 68·22 (203·64) | 0·00 (0·00; 0·00) | 83·92 (271·19) | 0·00 (0·00; 0·00) | 15·70 (227·09) | 0·00 (0·00; 0·00) |
| Intervention | 507 | 94·80 (254·90) | 0·00 (0·00; 0·00) | 76·46 (241·44) | 0·00 (0·00; 0·00) | −18·34 (184·74) | 0·00 (0·00; 0·00) |
| GLP1RA – any |  |  |  |  |  |  |  |
| Control | 10140 | 51·61 (280·31) | 0·00 (0·00; 0·00) | 68·13 (359·96) | 0·00 (0·00; 0·00) | 16·52 (286·86) | 0·00 (0·00; 0·00) |
| Intervention | 507 | 101·70 (409·58) | 0·00 (0·00; 0·00) | 98·89 (424·20) | 0·00 (0·00; 0·00) | −2·81 (364·29) | 0·00 (0·00; 0·00) |
| **Total** |  |  |  |  |  |  |  |
| **Control** | **10140** | **264·11 (491·88)** | **36·08 (0·00; 352·30)** | **344·52 (705·43)** | **46·80 (0·00; 513·78)** | **80·41 (624·16)** | **0·00 (−14·88; 56·21)** |
| **Intervention** | **507** | **345·14 (597·93)** | **46·96 (5·74; 535·64)** | **347·79 (674·90)** | **34·44 (2·46; 465·64)** | **2·65 (562·50)** | **0·00 (−59·44; 17·22)** |

Table e2.10: Generalised linear model (gamma) of prescription count, average dose, and total costs for the two years pre- and post-intervention

|  | Prescription Count | | | Ave Dose of Prescription | | | Total costs of Prescriptions | | |
| --- | --- | --- | --- | --- | --- | --- | --- | --- | --- |
|  | N | exp(B)(95%CI) | AIC | N | exp(B)(95%CI) | AIC | N | exp(B)(95%CI) | AIC |
| **Antidiabetic medicines** |  |  |  |  |  |  |  |  |  |
| Metformin – any |  |  |  |  |  |  |  |  |  |
| Unadjusted | 10,647 | **0·83 (0·76, 0·91)** | 70,754 | 10,647 | 1·02 (0·68, 1·51) | 142,272 | 10,647 | 0·84 (0·67, 1·04) | 206,211 |
| Adjusted | 10,642 | **0·84 (0·75, 0·93)** | 70,544 | 10,642 | 1·03 (0·70, 1·52) | 142,153 | 10,647 | 0·84 (0·68, 1·04) | 205,834 |
| Sulphonylurea – any |  |  |  |  |  |  |  |  |  |
| Unadjusted | 10,647 | 0·68 (0·38, 1·22) | 25,305 | 10,647 | 0·91 (0·76, 1·07) | 48,321 | 10,647 | **0·60 (0·39, 0·93)** | 141,347 |
| Adjusted | 10,642 | 0·67 (0·44, 1·02) | 24,361 | 10,642 | **0·82 (0·75, 0·89)** | 47,922 | 10,209 | **0·56 (0·37, 0·85)** | 134,921 |
| Pioglitazone |  |  |  |  |  |  |  |  |  |
| Unadjusted | 10,647 | **0·61 (0·42, 0·87)** | −31,152 | 10,647 | 0·81 (0·03, 19·97) | −10,251 | 10,647 | 0·71 (0·16, 3·06) | 93,646 |
| Adjusted | 10,642 | **0·27 (0·09, 0·79)** | −32,630 | 10,642 | **0·41 (0·23, 0·74)** | −13,301 | 10,642 | **0·14 (0·03, 0·60)** | 85,889 |
| DPP4i – any |  |  |  |  |  |  |  |  |  |
| Unadjusted | 10,647 | 0·66 (0·39, 1·13) | 28,977 | 10,647 | 0·91 (0·74, 1·13) | 48,460 | 10,647 | 0·71 (0·49, 1·03) | 202,672 |
| Adjusted | 10,642 | 0·61 (0·35, 1·06) | 28,170 | 10,642 | 0·97 (0·48, 1·96) | 47,414 | 10,647 | 0·73 (0·51, 1·04) | 201,663 |
| SGLT2i – any |  |  |  |  |  |  |  |  |  |
| Unadjusted | 10,647 | 0·68 (0·40, 1·15) | 29,499 | 10,647 | 1·03 (0·93, 1·13) | 57,211 | 10,647 | **0·70 (0·49, 0·99)** | 208,266 |
| Adjusted | 10,642 | 0·69 (0·42, 1·15) | 28,999 | 10,642 | 1·01 (0·54, 1·90) | 56,690 | 10,647 | 0·73 (0·52, 1·01) | 207,723 |
| GLP1RA – any |  |  |  |  |  |  |  |  |  |
| Unadjusted | 10,647 | 1·23 (0·82, 1·86) | −10,868 | 10,573 | 1·09 (0·76, 1·56) | −29,789 | 10,647 | 1·30 (0·66, 2·57) | 182,966 |
| Adjusted | 10,642 | 1·21 (0·78, 1·87) | −13,713 | 10,568 | **1·18 (1·03, 1·35)** | −34,550 | 10,642 | 1·21 (0·57, 2·55) | 178,656 |
| *Total antidiabetic* |  |  |  |  |  |  |  |  |  |
| *Unadjusted* | *10,647* | *0·82 (0·17, 4·03)* | *84,404* | *10,289* | *1·10 (0·61, 1·97)* | *132,932* | *10,647* | ***0·69 (0·58, 0·83)*** | *235,638* |
| *Adjusted* | *10,642* | ***0·83 (0·76, 0·91)*** | *84,222* | *10,284* | *1·11 (0·90, 1·37)* | *132,833* | *10,647* | ***0·68 (0·57, 0·81)*** | *235,362* |
| **Lipid-lowering medicines** |  |  |  |  |  |  |  |  |  |
| Statin – any |  |  |  |  |  |  |  |  |  |
| Unadjusted | 10,647 | **0·83 (0·76, 0·91)** | 67,689 | 10,647 | 0·96 (0·84, 1·09) | 79,882 | .. | .. | .. |
| Adjusted | 10,642 | **0·87 (0·79, 0·97)** | 66,516 | 10,647 | 0·97 (0·88, 1·08) | 78,782 | .. | .. | .. |
| Fibrate – any |  |  |  |  |  |  |  |  |  |
| Unadjusted | 10,647 | 0·35 (0·09, 1·37) | −12,340 | 10,647 | **0·56 (0·39, 0·81)** | 26,690 | .. | .. | .. |
| Adjusted | 10,642 | 0·27 (0·06, 1·35) | −18,659 | 10,642 | 0·76 (0·33, 1·75) | 20,771 | .. | .. | .. |
| Ezetimibe – any |  |  |  |  |  |  |  |  |  |
| Unadjusted | 10,647 | 0·61 (0·35, 1·06) | 18,288 | 10,647 | **0·42 (0·22, 0·78)** | 33,078 | .. | .. | .. |
| Adjusted | 10,642 | 0·72 (0·39, 1·32) | 15,569 | 10,642 | 0·57 (0·29, 1·15) | 30,579 | .. | .. | .. |
| Other lipid-lowering |  |  |  |  |  |  |  |  |  |
| Unadjusted | 10,647 | **0·25 (0·07, 0·90)** | −81,681 | 10,647 | 2·62 (0·33, 20·55) | 46,821 | .. | .. | .. |
| Adjusted | 10,209 | 0·81 (0·51, 1·29) | −86,750 | 10,209 | 2·15 (0·28, 16·47) | 38,902 | .. | .. | .. |
| *Total lipid-lowering* |  |  |  |  |  |  |  |  |  |
| *Unadjusted* | *10,647* | ***0·84 (0·76, 0·92)*** | *69,709* | *10,647* | *1·59 (0·22, 11·58)* | *85,279* | .. | .. | .. |
| *Adjusted* | *10,642* | *0·90 (0·58, 1·39)* | *68,464* | *10,642* | *1·45 (0·70, 3·02)* | *84,361* | .. | .. | .. |
| **Antihypertensive medicines** |  |  |  |  |  |  |  |  |  |
| Unadjusted | 10,647 | **0·84 (0·74, 0·95)** | 79,527 | 10,642 | 0·91 (0·59, 1·40) | 61,499 | .. | .. | .. |
| Adjusted | 10,642 | **0·82 (0·75, 0·91)** | 78,288 | 10,637 | 0·91 (0·77, 1·09) | 60,551 | .. | .. | .. |
| **Antiplatelet medicines** |  |  |  |  |  |  |  |  |  |
| Unadjusted | 10,647 | 0·57 (0·29, 1·15) | 39,456 | 10,645 | 0·78 (0·20, 3·12) | 58,360 | .. | .. | .. |
| Adjusted | 10,642 | 0·56 (0·15, 2·11) | 36,510 | 10,640 | **0·68 (0·48, 0·96)** | 57,137 | .. | .. | .. |

* Adjusted model is for age, gender, ethnicity IMD, and smoking status. Missing data in IMD and smoking status in some cases were dropped due to their effect on model fit (hence N=<10647).

# Heath Economics Modelling Methods

## Overview

A prospective economic evaluation was conducted alongside the Greater Manchester (GM) Diabetes My Way (DMW) clinical evaluation. (1) The economic evaluation was fully integrated into the research design with patient characteristics, medication use, and risk factor data collected during the study incorporated in the analyses. The comprehensive details of the economic evaluation are reported below.

Our analyses were guided with respect to the National Institute for Health and Care Excellence (NICE) reference case for economic evaluation. (2) The base-case analysis was conducted from the perspective of the UK National Health Service (NHS) over a 40-year (life)time horizon to capture important differences in costs and outcomes between the GM DMW and usual care. Costs and outcomes were appropriately discounted at 3·5% beyond the initial time period with costs reported in Pound Sterling (£) for the costing year 2021/22·

The Consolidated Health Economic Evaluation Reporting Standards (CHEERS) 2022 guidance was used in the development and reporting of this study. (3) All relevant items on the checklist were appropriately reported in eAppendix 5:.

## DMW usual Pricing Structure

The way MWDH is priced is to charge a base cost (50% of ‘full population’ license fee), and then an incremental cost based on the % of registrants using the service, with an additional 1/8 of the full population license cost charge for 5%, then 10%. then 15%, then 20% of population registrants. Once the region reaches 20% population registrant the license fee is capped- so in the case of Greater Manchester, they only paid £313,557 when they reached 20% population registrations. This licensing model aims to deliver cost-effectiveness earlier in the contract. However, we used the full population licence fee in the economic modelling in line with NICE guidance.’

## Intervention and Comparator

The intervention was the DMW digital self-management and educational platform. People with type 2 diabetes who registered to use DMW were participants in the intervention group. The inclusion and exclusion criteria for the study are listed in the protocol. (1)

The comparator were people with type 2 diabetes who did not register to use DMW but had access to usual care provided through the NHS. Comparators were found in the Manchester Care Records and matched to the intervention group (up to 10:1) based on age (±2 years), gender, ethnicity, and general practice (1). More information regarding the matching methods can be found in the main text.

## Microsimulation Model and Simulated Cohort

Lifetime costs and effects associated with short-term measures (e.g. reductions in HbA1c levels) observed in the study were estimated using the United Kingdom Prospective Diabetes Study (UKPDS) Outcomes Model version 2·1 (UKPDS-OM2.1) (4). The UKPDS-OM2.1 is a validated, patient-level microsimulation model that predicts incidence of events (e.g. myocardial infarction) and long-term outcomes (life expectancy, quality-adjusted life-years [QALYs], and costs) for groups with different baseline characteristics and risk profiles. (5) Full descriptions of model input and output definitions can be found in the source paper. (5)

We obtained access to the UKPDS-OM2.1 implementation in R used to generate the health economics evidence for the NICE Type 2 diabetes in adults: management guideline (NG28). This enabled us to simulate the same cohort as used for national policymaking purposes (6). In brief, the NICE Type 2 diabetes cohort was derived from The Health Improvement Network^®^ (THIN^®^) data (7) due to its large sample size, coverage of risk factors, and correlations between risk factors. We used a weighted average of individual patient risk profiles across the three intensification levels defined as median years since diagnosis used in NG28 (initial therapy: 1·5 years; first intensification: 4·5 years; and second intensification 8·5 years). (6)

Table e3.01 presents the mean baseline patient characteristics. Clinical risk factors missing from the THIN^®^ dataset (heart rate, haemoglobin level, and albuminuria) were sourced from the IQVIA CORE model (8) default setup and the literature (9), respectively. Table e3.02 presents the proportion of pre-existing medical events within the baseline population.

Table e3.01: Baseline Patient Characteristics

| Characteristics | Mean (SD) | Source |
| --- | --- | --- |
| Ethnic Characteristics | | |
| White (including others) | 94·58% | NG28 THIN |
| Asian | 2·73% | NG28 THIN |
| Black | 2·68% | NG28 THIN |
| Sex proportion | | |
| Female | 43·03% | NG28 THIN |
| Other demographic characteristics | | |
| Age at baseline | 60·68 (12·60) | NG28 THIN |
| Duration of diabetes (days) | 1469·93 | NG28 assumption |
| Weight (kg) | 89·85 (19·05) | NG28 THIN |
| Height (m) | 1·68 (0·10) | NG28 THIN |
| (UKPDS) Risk Factor values | | |
| Atrial fibrillation | 1·77% | NG28 THIN |
| Peripheral vascular disease | 0·57% | NG28 THIN |
| Current Smoker | 16·02% | NG28 THIN |
| Albuminuria | 15·00% | Adler et al (2003)[18] |
| High Density Lipids (mmol/mol) | 1·23 (0·32) | NG28 THIN |
| Low Density Lipids (mmol/mol) | 2·41 (0·89) | NG28 THIN |
| Systolic Blood Pressure (mmHg) | 134·05 (14·50) | NG28 THIN |
| HbA1c (%) | 7·69 (1·21) | NG28 |
| Heart rate (beats per minute) | 72·00 | IQVIA CORE Default |
| White Blood Cell Count | 7·58 (1·94) | NG28 THIN |
| Haemoglobin (g/dL) | 14·50 | IQVIA CORE Default |
| Estimated glomerular filtration rate (ml/min/1·73m2) | 73·14 (17·64) | NG28 THIN |
| Abbreviations: g/dL; grams per decilitre, HbA1c; Glycated haemoglobin, kg; kilogrammes, m; meters, mmHg; millimetres of mercury, mmol/mol; millimoles per mole, SD; standard deviation | | |

Table e3.02: Baseline Patient Preexisting Medical Events

| Characteristics | Mean (SD) | Source |
| --- | --- | --- |
| Medical History | | |
| Ischemic heart disease | 3·07% | THIN |
| Stroke | 0·65% | THIN |
| Blindness | 0·73% | THIN |
| Coronary heart disease | 1·01% | THIN |
| Myocardial infarction | 1·06% | THIN |
| Amputation | 0·12% | THIN |
| Renal failure | 0·20% | THIN |
| Ulcer | 0·40% | THIN |
| Time since event (days) | | |
| Ischemic heart disease | 1275·66 (936·07) | THIN |
| Stroke | 1073·83 (943·00) | THIN |
| Blindness | 1061·69 (790·80) | THIN |
| Coronary heart disease | 999·49 (861·85) | THIN |
| Myocardial infarction | 1151·67 (1001·42) | THIN |
| Amputation | 928·87 (852·95) | THIN |
| Renal failure | 1064·65 (901·63) | THIN |
| Ulcer | 1079·41 (902·10) | THIN |
| Abbreviations: SD; standard deviation | | |

## Treatment Effects

Statistically significant treatment effects, derived from the 24-month pre-and-post statistical analysis (see Table 2 in main text), were applied to participants in the intervention group, with no treatment effects applied to the usual care group. This allowed for a comparison between the participants that did and did not receive the GM DMW Intervention.

In the clinical evaluation, the GM DMW intervention demonstrated a reduction in HbA1c (−0·281% DCCT), a reduction in systolic blood pressure (−1·438 mmHg), a reduction in low density lipids (−0·100 mmol/mol), and no changes in high density lipids and weight.

In the base-case analysis, we assume the treatment effects and costs lasted 2-years with natural disease progression occurred thereafter, as predicted by the underlying UKPDS risk factor time path equations (10). Sensitivity analysis we explored the assumption of the treatment effect duration.

## Cost Inputs

### Intervention Costs

MWDH advised that the full annual licence fee for Greater Manchester (182,075 people with T2DM in February 2024) is £313,557· This fee is currently subject to a negotiated discount; however, following NICE’s stipulation that analyses should only reflect commercial arrangements when prices are nationally guaranteed for a technology’s lifetime, we use the full fee in our base case. We apportioned the fee among users (n=16,194 in December 2023) to derive a cost-per-user of £38 over the 2-year intervention period. We only applied this cost to people in the model who remained alive – equivalent to assuming that uptake of new users, over time, will be similar to attrition of existing users.

### Medication Costs

Medication use between treatment groups are an important source of resource use and cost for the NHS. The UKPDS-OM2.1 does not explicitly incorporate medication costs. Some additional analysis was undertaken using medication use data collected in the study to estimate total medication costs of antidiabetic drugs for each group (see e2.2).

Recorded medicinal product descriptions were mapped onto the dictionary of medicines and devices (dm+d) product descriptions and corresponding actual medicinal product packages (AMPP) and virtual medicinal product packages (VMPP). For some product descriptions, clinical guidance was sought to ascertain the correct product. VMPPs were mapped onto costs from the NHS Prescription Services Drug Tariff (June 2023). (11) We estimated mean total medication costs for each group using a generalised linear model (log-link and gamma family) adjusting for age, sex, ethnicity, deprivation, smoking status, and medication costs in the 2 years before intervention.

### Complication costs

Table e3.03 and Table e3.04 present the complication cost for male and female age ranges. Diabetes complication costs were sourced from Alva et al (2015) for myocardial infarction; ischemic heart disease; stroke; heart failure; amputation; and blindness. (12) The cost in the absence of complications was also calculated by the source study. (12) Costs for renal failure and ulcers were derived from non-UKPDS sources discussed below. Alva et al (2015) reported costs by sex and age (i.e., 50, 55, … 80 years) for annual hospital inpatient and non-inpatient resource use. The sum of both inpatient and non-inpatient costs were calculated for complication events and inflated to 2021/22 for ages 50; 60; 70; and 80· Costs were inflated using the HCHS Pay and Price Index (13) (2011/12 to 2014/15) and the NHSCII Pay and Price Index (14) thereafter. For the purpose of replication, this equates to an inflator value of 1·17625 (2011/12 to 2021/22).

For renal failure costs, a micro-costing approach used in a previous NICE guideline was adapted. For ulceration costs, a methodology used in the health economics evidence for the NICE Type 2 diabetes in adults: management guideline (NG28) was followed.

The cost of renal failure was calculated as a weighted average between (a) the cost of dialysis (both dialysis delivery and ongoing hospital service use) and (b) the cost of kidney transplantation (including the transplant procedure, immunosuppressive medication for life, and ongoing hospital service use):

1. The same micro-costing approach used in NICE NG148 for resource use associated with dialysis was applied. The cost of receiving dialysis was comprised of dialysis sessions, nephrology appointments, eGFR testing, medications (epoetin for a proportion of people), first cycle access procedure costs, as well as supplementary costs for travel and access maintenance. Uprating the NG148 costs to 2021/22 values (14) gave dialysis delivery cost estimates of £34,722 for year 1 and £32,538 thereafter. Costs associated with inpatient and outpatient hospital services of dialysis were sourced from models to predict expected costs as a function of patient-level characteristics including sex, age (50−64 years, 65−75 years, 75+ years), type and duration of dialysis, and comorbidities. (15) In fitting these models, the categorical value for the history of diabetes was set to 1 to best reflect a population of people with Type 2 diabetes. Costs were reported in the model by dialysis type (haemodialysis and peritoneal dialysis). A weighted average cost between the types of dialysis was calculated using the proportion of haemodialysis (87%) and peritoneal dialysis (13%) reported in the UK Renal Registry’s 22^nd^ annual report. (16) The costs of inpatient and outpatient hospital services were inflated to 2021/22 prices (14) and added to the cost of dialysis delivery to derive the total cost.
2. The estimated kidney transplant procedure costs were sourced from the National Cost Collection 2021/22 (17). Specifically, the sum between the average “work-up per transplant” and the weighted average of all kidney transplants was £24,133· Immunosuppressant medication costs in the transplant year and thereafter were sourced from Scotland et al. (2018) (18) and inflated to the 2021/22 price year (14) (£12,043 in year 1, and £10,265 thereafter). Costs associated with inpatient and outpatient hospital services of kidney transplantation were sourced from Li et al (2016). (15) As above, the categorical value for the history of diabetes was set to 1· Costs were reported in the model by transplantation type (deceased donor and living donor). A weighted average cost between the transplantation type was calculated using the proportion of deceased donors (75·3%) and live donors (24·7%) recorded in the National Cost Collection 2021/22 (codes: LA01A; LA02A; and LA03A). (17)

A weighted sum between the total costs of parts (a) and (b) was calculated based on the estimated proportion of dialysis (90·1%) to kidney transplantation (9·9%). (6)

The cost of diabetic ulcerations was calculated as a weighted average between inpatient hospital costs and community, outpatient, and primary care costs. Kerr et al., (2019) highlight the total costs and admissions for inpatients and community, outpatient, and primary care costs for ulcerations in England, 2014−15. (19) Costs were inflated to the 2021/22 price year. (14) It was assumed that all diabetic ulcer patients require community, outpatient, and/or primary care costs and half of all patients will require inpatient admissions. (20) Therefore, the total cost of diabetic ulceration is estimated to be £11,615·21 in year 1 only.

Table e3.03: Male Complication Costs and Absence of Complication Costs Inputs (GBP, 2021/22)

| Age ranges | Absence of complications | Complications | IHD | MI | HF | Stroke | Amputation | Blindness | Renal Failure | Ulcer |
| --- | --- | --- | --- | --- | --- | --- | --- | --- | --- | --- |
| Male aged < 50y | 907·36 | Fatal cost | 3,916·01 | 1,340·61 | 0 | 463·32 | 0 | - | 0 | - |
|  |  | Non-fatal cost (event year) | 11,271·53 | 7,975·64 | 4,175·13 | 7,851·18 | 13,503·36 | 3,134·27 | 44,387·77 | 11,615·21 |
|  |  | Non-fatal cost (subsequent year) | 1,674·53 | 1,643·89 | 2,275·85 | 1,719·00 | 3,325·22 | 1,136·94 | 37,235·02 | 0 |
| Male  aged ≥ 50y and < 60y | 1,165·51 | Fatal cost | 4,429·70 | 1,789·55 | 0 | 732·33 | 0 | - | 0 | - |
|  |  | Non-fatal cost (event year) | 12,504·07 | 8,636·08 | 4,904·83 | 9,287·78 | 14,402·96 | 3,699·02 | 44,387·77 | 11,615·21 |
|  |  | Non-fatal cost (subsequent year) | 2,197·61 | 2,146·63 | 2,877·26 | 2,212·91 | 4,003·22 | 1,400·72 | 37,235·02 | 0 |
| Male aged ≥ 60y and < 70y | 1,524·88 | Fatal cost | 4,927·86 | 2,240·75 | 0 | 1,121·38 | 0 | - | 0 | - |
|  |  | Non-fatal cost (event year) | 13,577·31 | 9,252·37 | 5,618·60 | 10,650·65 | 15,196·65 | 4,414·55 | 44,089·30 | 11,615·21 |
|  |  | Non-fatal cost (subsequent year) | 2,879·97 | 2,797·13 | 3,636·00 | 2,868·32 | 4,811·37 | 1,765·85 | 36,932·40 | 0 |
| Male aged ≥ 70y and < 80y | 2,009·14 | Fatal cost | 5,412·52 | 2,691·87 | 0 | 1,655·10 | 0 | - | 0 | - |
|  |  | Non-fatal cost (event year) | 14,506·19 | 9,833·89 | 6,300·55 | 11,884·82 | 15,905·96 | 5,265·29 | 43,639·60 | 11,615·21 |
|  |  | Non-fatal cost (subsequent year) | 3,720·77 | 3,589·93 | 4,533·98 | 3,694·29 | 5,710·56 | 2,250·27 | 36,513·75 | 0 |
| Male aged ≥ 80y | 2,009·14 | Fatal cost | 5,412·52 | 2,691·87 | 0 | 1,655·10 | 0 | - | 0 | - |
|  |  | Non-fatal cost (event year) | 14,506·19 | 9,833·89 | 6,300·55 | 11,884·82 | 15,905·96 | 5,265·29 | 43,639·60 | 11,615·21 |
|  |  | Non-fatal cost (subsequent year) | 3,720·77 | 3,589·93 | 4,533·98 | 3,694·29 | 5,710·56 | 2,250·27 | 36,513·75 | 0 |
| Sources | (12) | - | (12) | (12) | (12) | (12) | (12) | (12) | Author calculations  (see e3.5.3) | Author calculations  (see e3.5.3) |

Abbreviations: HF; Heart Failure, MI; Myocardial Infarction, IHD; Ischemic Heart Disease, ‘y’; years

Table e3.04: Female Complication Costs and Absence of Complication Costs Inputs (GBP, 2021/22)

| Age ranges | Absence of complications | Complications | IHD | MI | HF | Stroke | Amputation | Blindness | Renal Failure | Ulcer |
| --- | --- | --- | --- | --- | --- | --- | --- | --- | --- | --- |
| Female aged < 50y | 907·36 | Fatal cost | 4,186·97 | 1,593·66 | 0 | 545·41 | 0 | - | 0 | - |
|  |  | Non-fatal cost (event year) | 11,921·59 | 8,467·69 | 4,656·63 | 8,520·61 | 14,065·60 | 3,488·97 | 44,792·16 | 11,615·21 |
|  |  | Non-fatal cost (subsequent year) | 2,011·21 | 1,978·39 | 2,640·23 | 2,044·04 | 3,724·18 | 1,400·75 | 37,609·15 | 0 |
| Female aged ≥ 50y and < 60y | 1,165·51 | Fatal cost | 4,698·71 | 2,045·37 | 0 | 852·4 | 0 | - | 0 | - |
|  |  | Non-fatal cost (event year) | 13,115·87 | 9,117·70 | 5,393·70 | 9,956·05 | 14,937·47 | 4,107·56 | 44,792·16 | 11,615·21 |
|  |  | Non-fatal cost (subsequent year) | 2,587·82 | 2,532·52 | 3,297·50 | 2,590·35 | 4,454·49 | 1,699·12 | 37,609·15 | 0 |
| Female aged ≥ 60y and < 70y | 1,524·88 | Fatal cost | 5,194·57 | 2,497·93 | 0 | 1,289·44 | 0 | - | 0 | - |
|  |  | Non-fatal cost (event year) | 14,151·38 | 9,725·29 | 6,106·94 | 11,296·56 | 15,708·11 | 4,876·99 | 44,496·45 | 11,615·21 |
|  |  | Non-fatal cost (subsequent year) | 3,328·13 | 3,237·33 | 4,111·62 | 3,305·21 | 5,306·91 | 2,107·08 | 37,305·90 | 0 |
| Female aged ≥ 70y and < 80y | 2,009·14 | Fatal cost | 5,677·05 | 2,949·60 | 0 | 1,877·86 | 0 | - | 0 | - |
|  |  | Non-fatal cost (event year) | 15,047·65 | 10,299·98 | 6,784·04 | 12,497·32 | 16,399·49 | 5,772·98 | 44,043·13 | 11,615·21 |
|  |  | Non-fatal cost (subsequent year) | 4,222·82 | 4,079·11 | 5,055·33 | 4,189·99 | 6,235·58 | 2,638·91 | 36,880·48 | 0 |
| Female aged ≥ 80y | 2,009·14 | Fatal cost | 6,658·00 | 2,280·00 | 0 | 7,156·00 | 0 | - | 0 | - |
|  |  | Non-fatal cost (event year) | 17,518·00 | 11,706·00 | 5,222·00 | 11,126·00 | 17,642·00 | 1,830·00 | 44,043·13 | 11,615·21 |
|  |  | Non-fatal cost (subsequent year) | 4,448·00 | 4,266·00 | 5,165·00 | 4,434·00 | 6,108·00 | 2,798·00 | 36,880·48 | 0 |
| Sources | (12) | - | (12) | (12) | (12) | (12) | (12) | (12) | Author calculations  (see e3.5.3) | Author calculations  (see e3.5.3) |

Abbreviations: HF; Heart Failure, MI; Myocardial Infarction, IHD; Ischemic Heart Disease, ‘y’; years

## Health Utility Inputs

All utility values are reported in Table e3.05. Initial health utility was assumed to be 0·815 (6) for all simulated patients. Statistically significant complication utility decrements (myocardial infarction; stroke; heart failure; and amputation) were applied irrespective of age or sex from Alva et al (2014). (21) In subsequent years, the same magnitude decrements were applied for statistically significant complications including stroke; heart failure; and amputation. A meta-analysis of quality-of-life studies provided estimates of ulceration utility decrements. (22) This source allows for inpatients and outpatient costs to be included for treating ulcers.

The UKPDS default utility decrement is taken from Lung et al (2011). (22) The utility decrement estimated in this study is for End Stage Kidney Disease (ESKD). However, the definition of Renal Failure (>250μm/l) used in the UKPDS-OM will include stages of chronic kidney disease (CKD) before ESKD and may overestimate the health utility decrement within the event year. Therefore, we calculated a weighted average between utility decrements of dialysis and kidney transplantation across stages of CKD. Schlackow et al (2017) (23) performed multivariable regression analysis using the Study of Heart and Renal Protection (SHARP) study to estimate that dialysis status was associated with a reduction in utility of 0·06 (0·04–0·07). In the absence of dialysis, the mean utility of stage 3b−5 chronic kidney disease is 0·766. Therefore, the absolute utility value for people on dialysis is (0·766−0·06) 0·706 which compared to baseline utility derives a utility multiplier of 0·866· Li et al (2017) (24) estimate an independent effect of a kidney transplantation (adjusted for age and sex) of a gain in utility of 0·053. Therefore, the absolute utility value for people on dialysis is (0·766+0·053) 0·759 which compared to baseline utility derives a utility multiplier of 0·931. The absolute utility decrement of dialysis was (0·706−0·815) −0·109 and of kidney transplantation was (0·759−0·815) −0·056. A weighted average of both utility decrements was calculated based on the estimated proportion of dialysis (90·1%) to kidney transplantation (9·9%) (6) to derive a utility decrement from renal failure of −0·104 in the event year. In the subsequent year, we assume a utility decrement of −0·330 (22) because ESKD is more likely.

Table e3.05: Health Utility Decrement Inputs

| Complication | Utility decrement  (event year) | Source | Utility decrement  (subsequent year) | Source |
| --- | --- | --- | --- | --- |
| Ischemic heart disease | 0 | (21) | 0 | (21) |
| Stroke | −0·065 | (21) | 0 | (21) |
| Blindness | −0·101 | (21) | −0·101 | (21) |
| Heart Failure | −0·165 | (21) | −0·165 | (21) |
| Myocardial Infarction | −0·172 | (21) | −0·172 | (21) |
| Amputation | 0 | (21) | 0 | (21) |
| Renal Failure | −0·104 | Author calculations (see e3.6) | −0·330 | (21) |
| Ulcer | −0·210 | (22) | −0·210 | (22) |

## Presenting Results

We reported health effects (life years and QALYs gained), disaggregated complication costs, intervention costs, and medication costs for both treatment and reported the relative difference in effects and costs. The cost per QALY gained was reported for those using DMW compared with usual care as an estimate of cost-effectiveness. This was calculated as the relative difference in both costs and QALYs gained between the treatment groups. In England and Wales, NICE considers health technologies demonstrating a lifetime below £20−30,000 per QALY gained as cost-effective. (2)

Risk factor progression equations are presented for HbA1c, systolic blood pressure, low density lipids, high density lipids, and weight. The equations are reported in graphs to show the predicted trajectory of the clinical risk factor over time.

It is important to mention that when scaling the patient-level cost-effectiveness results to the population-level, the current estimate of people with type 2 diabetes in GM is used (n=182,075, as of February 2024). In doing so, the presented results represent the difference between the usual care group and intervention group assuming full uptake of the intervention within the population.

The number of expected complication events over a 10-year period were estimated. This will provide insight into the difference in the predicted number of events between the intervention and usual care groups. Additionally, it will enable the calculation of the number needed to treat (NNT) to avoid each event.

## Planned Sensitivity Analysis

To examine the impact of some of the key assumptions made, sensitivity analyses will be performed.

### Cost and Effect Durations

Those using the GM DMW intervention may or may not experience health benefits beyond the 24-month analysis period. Equally, costs for providing the intervention may persist into the future. In the base case analysis, we examine the impact of 2-year costs and effects with natural disease progression occurring thereafter. To address the potential impact on the decision rule, we explore 3 other scenarios: 1A. permanent costs and effects over the 40-year period (i.e. constant costs and effects are maintained beyond the analysis period between intervention and usual care groups); 1B. 5-year costs and effects (i.e. costs and effects are maintained for 5-years, then natural disease progression occurs thereafter); 1C. immediate reversion to disease trajectory after the 2-year costs and effects (i.e. costs and effects are maintained for 2-years, then disease trajectory immediately reverts to the trajectory of usual care thereafter). Note that, costs are only incurred for individuals within the cohort who are alive.

### Treatment Effect Magnitude

The mean treatment effects may be under or overestimating the true health effects. To address the potential impact on the decision rule, we explore best case and worse case scenarios using the upper and lower confidence interventions. The best-case clinical risk factor change is examined for HbA1c (−0·423% DCCT), a reduction in systolic blood pressure (−2·688 mmHg), a reduction in low density lipids (−0·194 mmol/mol), and no changes in high density lipids and weight. The worse case clinical risk factor change is examined for HbA1c (−0·138% DCCT), a reduction in systolic blood pressure (−0·188 mmHg), a reduction in low density lipids (−0·006 mmol/mol), and no changes in high density lipids and weight.

### Different Time Horizons

The 40-year time horizon may not reflect the information requirements for short-term planning decisions with the GM area. Therefore, the analysis was conducted over both 5- and 10-year time horizons to determine the cost-effectiveness of the GM DMW intervention.

### Different Uptake Levels and Intervention Costs

The decision rule may be influenced by changes in the level of uptake in the population and changes in the cost charged to the healthcare system to operate the intervention. Two-way visualisation graphs are reported to show the degree of cost-effectiveness (represented by colour) given the level of uptake and intervention cost per person.

### Different Parameter Inputs

There may be uncertainty surrounding the choice in parameter inputs (i.e. costs and utility values) used in the analyses. Therefore, the analysis was conducted using the UKPDS-OM default setup that was received when obtaining the model license. Notably, this default setup does not include outpatient costs for event complications. Costs were inflated and reported in Table e3.06 and Table e3.07 for males and females, respectively. Utility values are reported in Table e3.08.

Table e3.06: UKPDS-OM Default Setup, Male Complication Costs and Absence of Complication Costs Inputs (GBP, 2021/22)

| Age ranges | Absence of complications | Complications | IHD | MI | HF | Stroke | Amputation | Blindness | Renal Failure | Ulcer |
| --- | --- | --- | --- | --- | --- | --- | --- | --- | --- | --- |
| Male aged < 50y | 907·36 | Fatal cost | 3,916·01 | 1,340·61 | 0 | 463·32 | 0 | - | 0 | - |
|  |  | Non-fatal cost (event year) | 10,302·65 | 6,890·59 | 3,070·66 | 6,615·05 | 10,376·19 | 1,076·11 | 20,198·82 | 6,945·91 |
|  |  | Non-fatal cost (subsequent year) | 952·97 | 901·47 | 1,178·38 | 876·79 | 1,476·93 | 316·43 | 20,198·82 | 0 |
| Male  aged ≥ 50y and < 60y | 1,165·51 | Fatal cost | 4,429·70 | 1,789·55 | 0 | 732·33 | 0 | - | 0 | - |
|  |  | Non-fatal cost (event year) | 11,487·97 | 7,503·80 | 3,753·13 | 8,004·42 | 11,228·56 | 1,593·64 | 20,198·82 | 6,945·91 |
|  |  | Non-fatal cost (subsequent year) | 1,428·82 | 1,356·98 | 1,732·55 | 1,323·47 | 2,107·70 | 532·98 | 20,198·82 | 0 |
| Male aged ≥ 60y and < 70y | 1,524·88 | Fatal cost | 4,927·86 | 2,240·75 | 0 | 1,121·38 | 0 | - | 0 | - |
|  |  | Non-fatal cost (event year) | 12,513·97 | 8,072·85 | 4,419·67 | 9,320·06 | 11,975·01 | 2,261·94 | 20,198·82 | 6,945·91 |
|  |  | Non-fatal cost (subsequent year) | 2,063·95 | 1,960·25 | 2,444·07 | 1,931·65 | 2,868·62 | 850·88 | 20,198·82 | 0 |
| Male aged ≥ 70y and < 80y | 2,009·14 | Fatal cost | 5,412·52 | 2,691·87 | 0 | 1,655·10 | 0 | - | 0 | - |
|  |  | Non-fatal cost (event year) | 13,395·62 | 8,607·14 | 5,054·39 | 10,506·99 | 12,637·10 | 3,065·45 | 20,198·82 | 6,945·91 |
|  |  | Non-fatal cost (subsequent year) | 2,857·52 | 2,705·81 | 3,294·81 | 2,710·39 | 3,720·58 | 1,288·08 | 20,198·82 | 0 |
| Male aged ≥ 80y | 2,009·14 | Fatal cost | 5,412·52 | 2,691·87 | 0 | 1,655·10 | 0 | - | 0 | - |
|  |  | Non-fatal cost (event year) | 13,395·62 | 8,607·14 | 5,054·39 | 10,506·99 | 12,637·10 | 3,065·45 | 20,198·82 | 6,945·91 |
|  |  | Non-fatal cost (subsequent year) | 2,857·52 | 2,705·81 | 3,294·81 | 2,710·39 | 3,720·58 | 1,288·08 | 20,198·82 | 0 |
| Sources | (12) | - | (12) | (12) | (12) | (12) | (12) | (12) | (25) | (20) |

Abbreviations: HF; Heart Failure, MI; Myocardial Infarction, IHD; Ischemic Heart Disease, ‘y’; years

Table e3.07: UKPDS-OM Default Setup, Female Complication Costs and Absence of Complication Costs Inputs (GBP, 2021/22)

| Age ranges | Absence of complications | Complications | IHD | MI | HF | Stroke | Amputation | Blindness | Renal Failure | Ulcer |
| --- | --- | --- | --- | --- | --- | --- | --- | --- | --- | --- |
| Female aged < 50y | 907·36 | Fatal cost | 4,186·97 | 1,593·66 | 0 | 545·41 | 0 | - | 0 | - |
|  |  | Non-fatal cost (event year) | 10,761·75 | 7,191·67 | 3,361·20 | 7,093·52 | 10,747·46 | 1,239·86 | 20,198·82 | 6,945·91 |
|  |  | Non-fatal cost (subsequent year) | 1,098·69 | 1,045·01 | 1,351·80 | 1,010·87 | 1,684·93 | 389·29 | 20,198·82 | 0 |
| Female aged ≥ 50y and < 60y | 1,165·51 | Fatal cost | 4,698·71 | 2,045·37 | 0 | 852·40 | 0 | - | 0 | - |
|  |  | Non-fatal cost (event year) | 11,908·80 | 7,794·45 | 4,051·04 | 8,481·73 | 11,572·11 | 1,811·21 | 20,198·82 | 6,945·91 |
|  |  | Non-fatal cost (subsequent year) | 1,628·07 | 1,551·90 | 1,961·84 | 1,509·94 | 2,368·00 | 640·42 | 20,198·82 | 0 |
| Female aged ≥ 60y and < 70y | 1,524·88 | Fatal cost | 5,194·57 | 2,497·93 | 0 | 1,289·44 | 0 | - | 0 | - |
|  |  | Non-fatal cost (event year) | 12,897·08 | 8,354·82 | 4,717·04 | 9,775·00 | 12,295·52 | 2,533·42 | 20,198·82 | 6,945·91 |
|  |  | Non-fatal cost (subsequent year) | 2,321·15 | 2,209·48 | 2,728·72 | 2,177·58 | 3,173·20 | 1,001·15 | 20,198·82 | 0 |
| Female aged ≥ 70y and < 80y | 2,009·14 | Fatal cost | 5,677·05 | 2,949·60 | 0 | 1,877·86 | 0 | - | 0 | - |
|  |  | Non-fatal cost (event year) | 13,746·12 | 8,882·27 | 5,346·92 | 10,928·54 | 12,939·67 | 3,382·17 | 20,198·82 | 6,945·91 |
|  |  | Non-fatal cost (subsequent year) | 3,168·61 | 3,004·03 | 3,625·20 | 3,015·13 | 4,054·63 | 1,485·75 | 20,198·82 | 0 |
| Female aged ≥ 80y | 2,009·14 | Fatal cost | 5,412·52 | 2,691·87 | 0 | 1,655·10 | 0 | - | 0 | - |
|  |  | Non-fatal cost (event year) | 13,746·12 | 8,882·27 | 5,346·92 | 10,928·54 | 12,939·67 | 3,382·17 | 20,198·82 | 6,945·91 |
|  |  | Non-fatal cost (subsequent year) | 3,168·61 | 3,004·03 | 3,625·20 | 3,015·13 | 4,054·63 | 1,485·75 | 20,198·82 | 0 |
| Sources | (12) | - | (12) | (12) | (12) | (12) | (12) | (12) | (25) | (20) |

Abbreviations: HF; Heart Failure, MI; Myocardial Infarction, IHD; Ischemic Heart Disease, ‘y’; years

Table e3.08: UKPDS-OM Default Setup, Health Utility Decrement Inputs

| Complication | Utility decrement  (event year) | Source | Utility decrement  (subsequent year) | Source |
| --- | --- | --- | --- | --- |
| Ischemic heart disease | 0 | (21) | 0 | (21) |
| Stroke | −0·065 | (21) | 0 | (21) |
| Blindness | −0·101 | (21) | −0·101 | (21) |
| Heart Failure | −0·165 | (21) | −0·165 | (21) |
| Myocardial Infarction | −0·172 | (21) | −0·172 | (21) |
| Amputation | 0 | (21) | 0 | (21) |
| Renal Failure | −0·330 | (21) | −0·330 | (21) |
| Ulcer | −0·210 | (22) | −0·210 | (22) |

## Planned Scenario Analysis

The national diabetes patient cohort (averaged across three intensification levels) used in the base case analyses may not be fully representative of the patient population in GM. To examine the impact of different populations, two separate scenario analyses were be performed:

(1) the patient characteristics observed in the THIN^®^ data for individuals on initial therapy;

(2) the patient characteristics observed in the GM population using the available demographic, risk factor and correlation data collected in the GM DMW study. A patient population representative of the patients in the study was generated using multivariate normal sampling. We derived a correlation matrix for all patients (i.e. with missing data) using the make.positive.definite function from the {corpcor} package in R to ensure a positive definite covariance matrix for Cholesky decomposition.

Baseline patient characteristics and pre-existing medical events for both populations are reported in the Table e3.09 and Table e3.10, and Table e3.11 and Table e3.12, respectively.

Table e3.09: Baseline Patient Characteristics of initial therapy population from NG28

| Characteristics | Mean (SD) | Source |
| --- | --- | --- |
| Ethnic Characteristics | | |
| White (including others) | 94·58% | THIN |
| Asian | 2·74% | THIN |
| Black | 2·69% | THIN |
| Sex proportion | | |
| Female | 42·94% | THIN |
| Other demographic characteristics | | |
| Age at baseline | 59·82 (12·61) | THIN |
| Duration of diabetes (days) | 548 | NG28 |
| Weight (kg) | 90·49 (19·25) | THIN |
| Height (m) | 1·68 (0·10) | THIN |
| (UKPDS) Risk Factor values | | |
| Atrial fibrillation | 1·00% | THIN |
| Peripheral vascular disease | 0·00% | THIN |
| Current Smoker | 17·00% | THIN |
| Albuminuria | 15·00% | Adler et al (2003)[18] |
| High Density Lipids (mmol/mol) |  |  |
| Low Density Lipids (mmol/mol) | 1·22 (0·31) | THIN |
| Systolic Blood Pressure (mmHg) | 2·55 (0·91) | THIN |
| HbA1c (%) | 134·64 (14·69) | THIN |
| Heart rate (beats per minute) | 8·18 (1·23) | NG28 |
| White Blood Cell Count | 72·00 | IQVIA CORE Default |
| Haemoglobin (g/dL) | 7·58 (1·95) | THIN |
| Estimated glomerular filtration rate (ml/min/1·73m2) | 14·50 | IQVIA CORE Default |
| Abbreviations: g/dL; grams per decilitre, HbA1c; Glycated haemoglobin, kg; kilogrammes, m; meters, mmHg; millimetres of mercury, mmol/mol; millimoles per mole, SD; standard deviation | | |

Table e3.10: Baseline Patient Preexisting Medical Events of initial therapy population from NG28

| Characteristics | Mean (SD) | Source |
| --- | --- | --- |
| Medical History | | |
| Ischemic heart disease | 1·40% | THIN |
| Stroke | 0·30% | THIN |
| Blindness | 0·30% | THIN |
| Coronary heart disease | 0·50% | THIN |
| Myocardial infarction | 0·50% | THIN |
| Amputation | 0·10% | THIN |
| Renal failure | 0·10% | THIN |
| Ulcer | 0·20% | THIN |
| Time since event (days) | | |
| Ischemic heart disease | 1275·66 (936·07) | THIN |
| Stroke | 1073·83 (943·00) | THIN |
| Blindness | 1061·69 (790·80) | THIN |
| Coronary heart disease | 999·49 (861·85) | THIN |
| Myocardial infarction | 1151·67 (1001·42) | THIN |
| Amputation | 928·87 (852·95) | THIN |
| Renal failure | 1064·65 (901·63) | THIN |
| Ulcer | 1079·41 (902·10) | THIN |
| Abbreviations: SD; standard deviation | | |

Table e3.11: Baseline Patient Characteristics of the GM population

| Characteristics | Mean (SD) | Source |
| --- | --- | --- |
| Ethnic Characteristics | | |
| White (including others) | 69·14% | GM Care Records |
| Asian | 5·17% | GM Care Records |
| Black | 2·27% | GM Care Records |
| Sex proportion | | |
| Female | 52·78% | GM Care Records |
| Other demographic characteristics | | |
| Age at baseline | 53·62 (12·21) | GM Care Records |
| Duration of diabetes (days) | 3380·55 (2310·38) | GM Care Records |
| Weight (kg) | 90·36 (22·16) | GM Care Records |
| Height (m) | 1·67 (0·11) | GM Care Records |
| (UKPDS) Risk Factor values | | |
| Atrial fibrillation | 2·71% | GM Care Records |
| Peripheral vascular disease | 2·10% | GM Care Records |
| Current Smoker | 18·21% | GM Care Records |
| Albuminuria | 15·00% | Adler et al (2003)[18] |
| High Density Lipids (mmol/mol) |  |  |
| Low Density Lipids (mmol/mol) | 1·19 (0·32) | GM Care Records |
| Systolic Blood Pressure (mmHg) | 2·27 (0·95) | GM Care Records |
| HbA1c (%) | 130·37 (14·99) | GM Care Records |
| Heart rate (beats per minute) | 7·66 (1·80) | GM Care Records |
| White Blood Cell Count | 72·00 | IQVIA CORE Default |
| Haemoglobin (g/dL) | 7·58 (1·95) | THIN |
| Estimated glomerular filtration rate (ml/min/1·73m2) | 14·50 | IQVIA CORE Default |
| Abbreviations: g/dL; grams per decilitre, HbA1c; Glycated haemoglobin, kg; kilogrammes, m; meters, mmHg; millimetres of mercury, mmol/mol; millimoles per mole, SD; standard deviation | | |

Table e3.12: Baseline Patient Preexisting Medical Events of the GM population

| Characteristics | Mean (SD) | Source |
| --- | --- | --- |
| Medical History | | |
| Ischemic heart disease | 9·57% | GM Care Records |
| Stroke | 5·02% | GM Care Records |
| Blindness | 2·99% | GM Care Records |
| Coronary heart disease | 1·01% | THIN (average) |
| Myocardial infarction | 1·06% | THIN (average) |
| Amputation | 0·12% | THIN (average) |
| Renal failure | 0·20% | THIN (average) |
| Ulcer | 0·40% | THIN (average) |
| Time since event (days) | | |
| Ischemic heart disease | 1931·12 (934·77) | GM Care Records |
| Stroke | 1547·63 (972·74) | GM Care Records |
| Blindness | 1755·05 (935·02) | GM Care Records |
| Coronary heart disease | 1411·25 (951·11) | GM Care Records |
| Myocardial infarction | 1664·84 (1000·96) | GM Care Records |
| Amputation | 1399·21 (1029·34) | GM Care Records |
| Renal failure | 1399·38 (898·65) | GM Care Records |
| Ulcer | 1586·93 (960·37) | GM Care Records |
| Abbreviations: SD; standard deviation | | |

# Health Economics Modelling Results

## Time path trajectories

Figure e4.01: Glycated haemoglobin progression

| 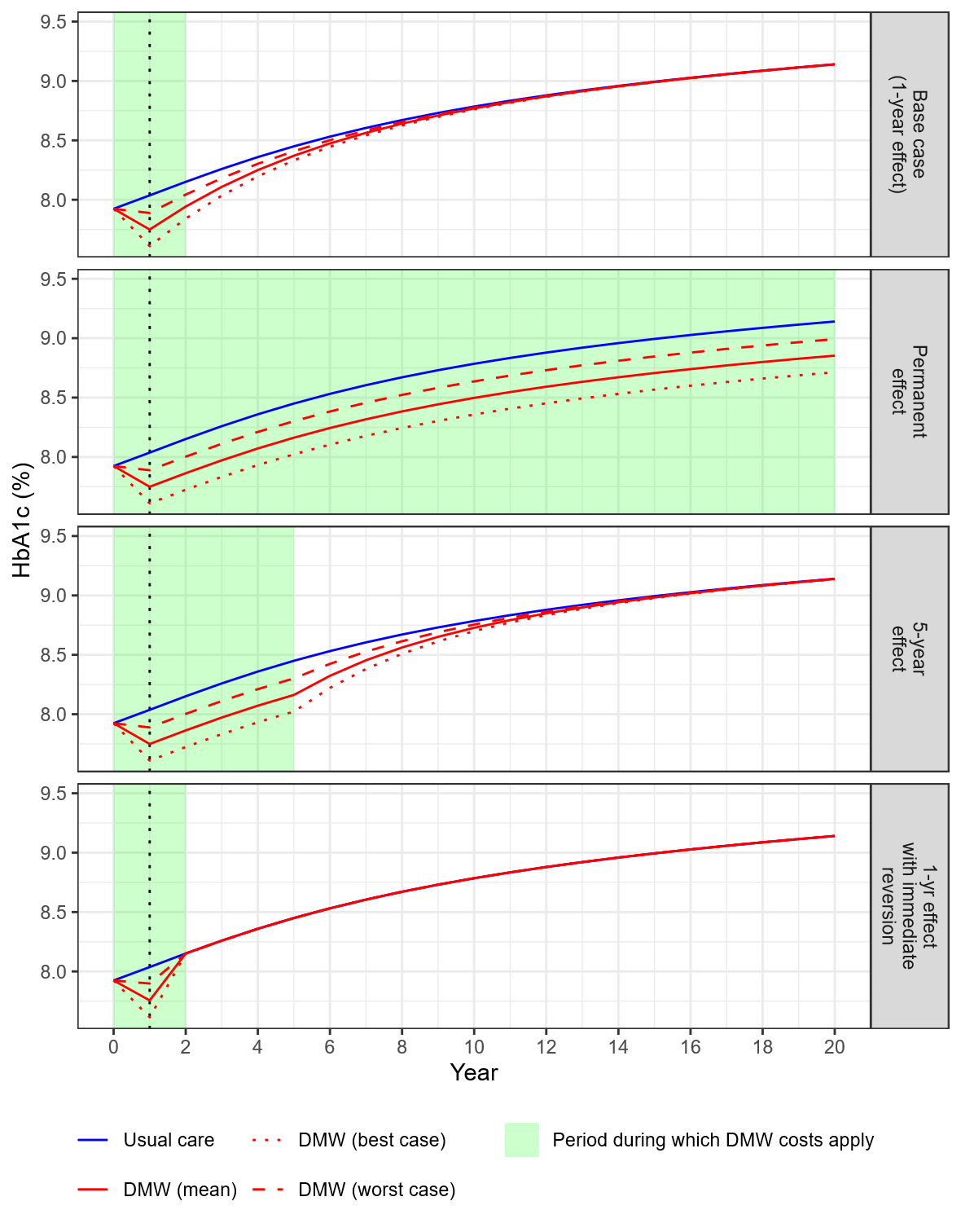 |
| --- |

Figure e4.02: Systolic blood pressure progression

| 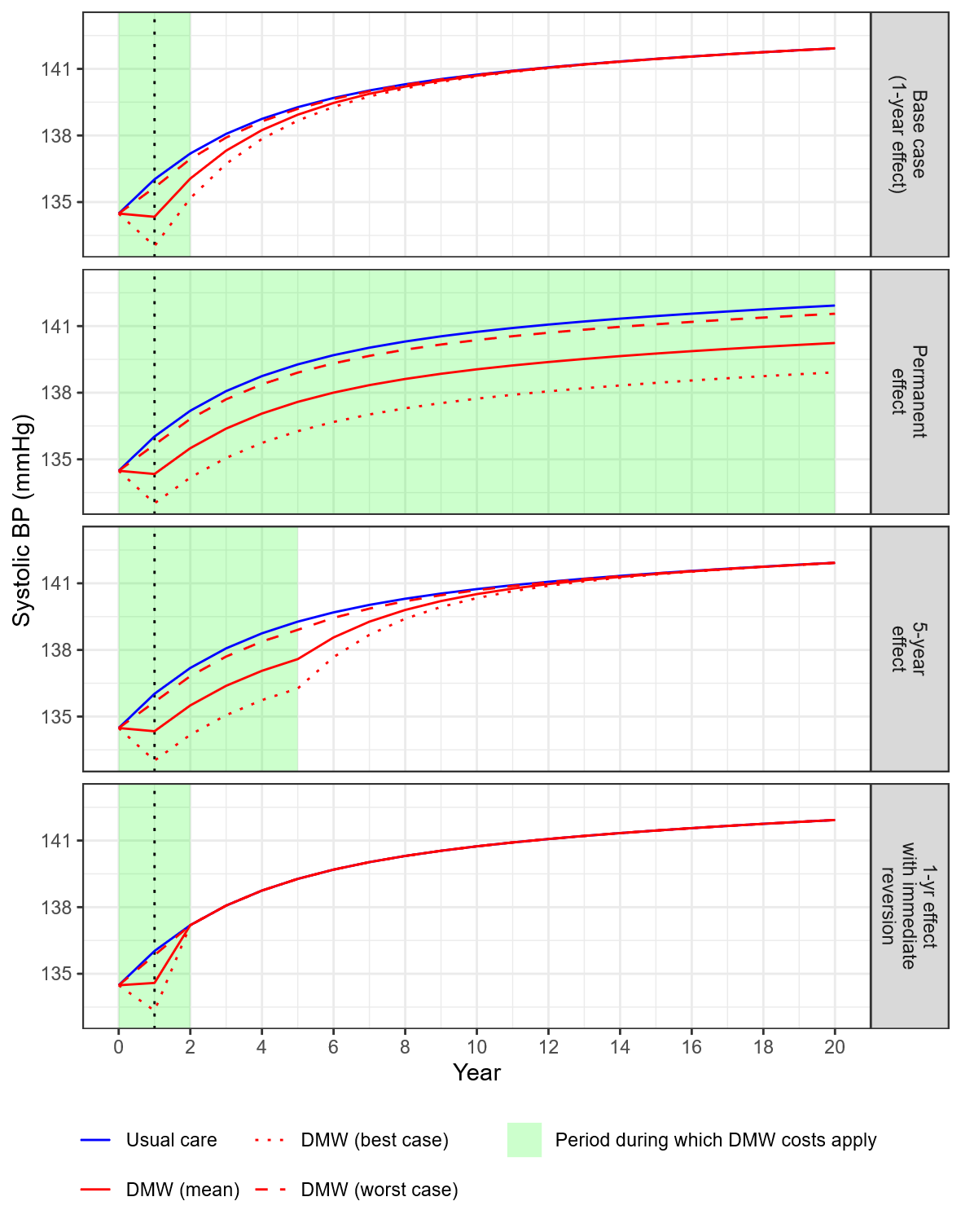 |
| --- |

Figure e4.03: Low density lipid progression

| 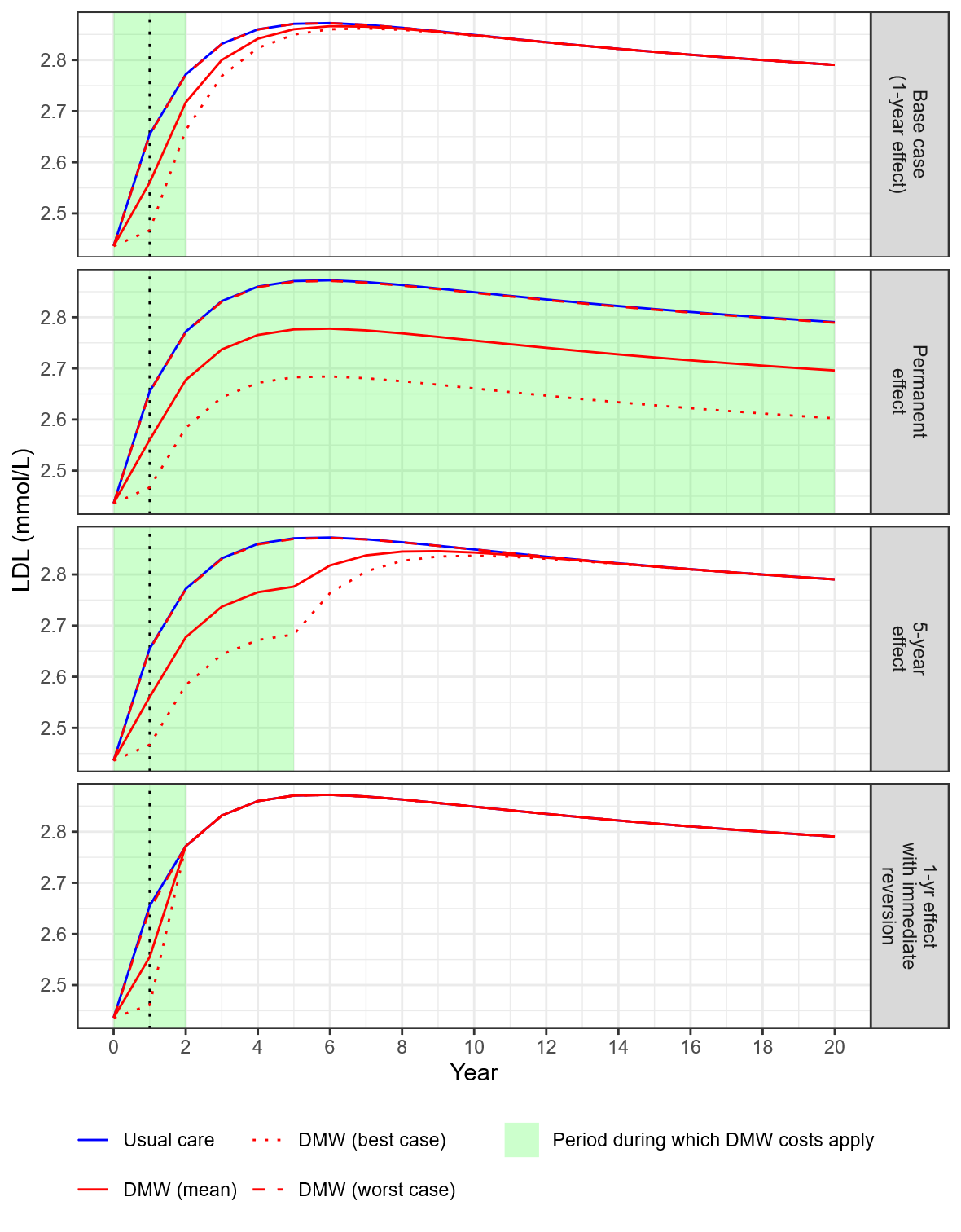 |
| --- |

Figure e4.04: High density lipid progression

| 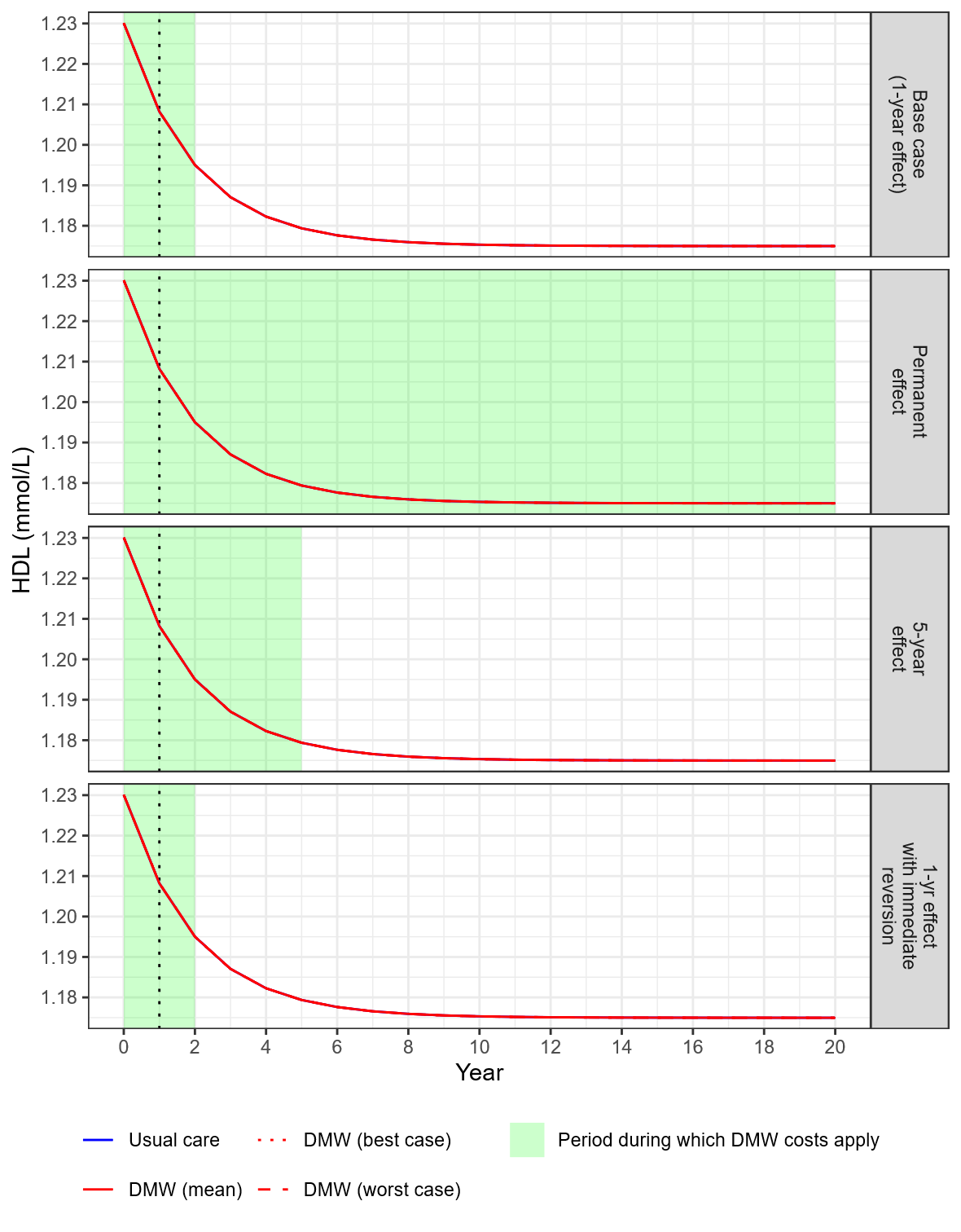 |
| --- |

Figure e4.05: Weight progression

| 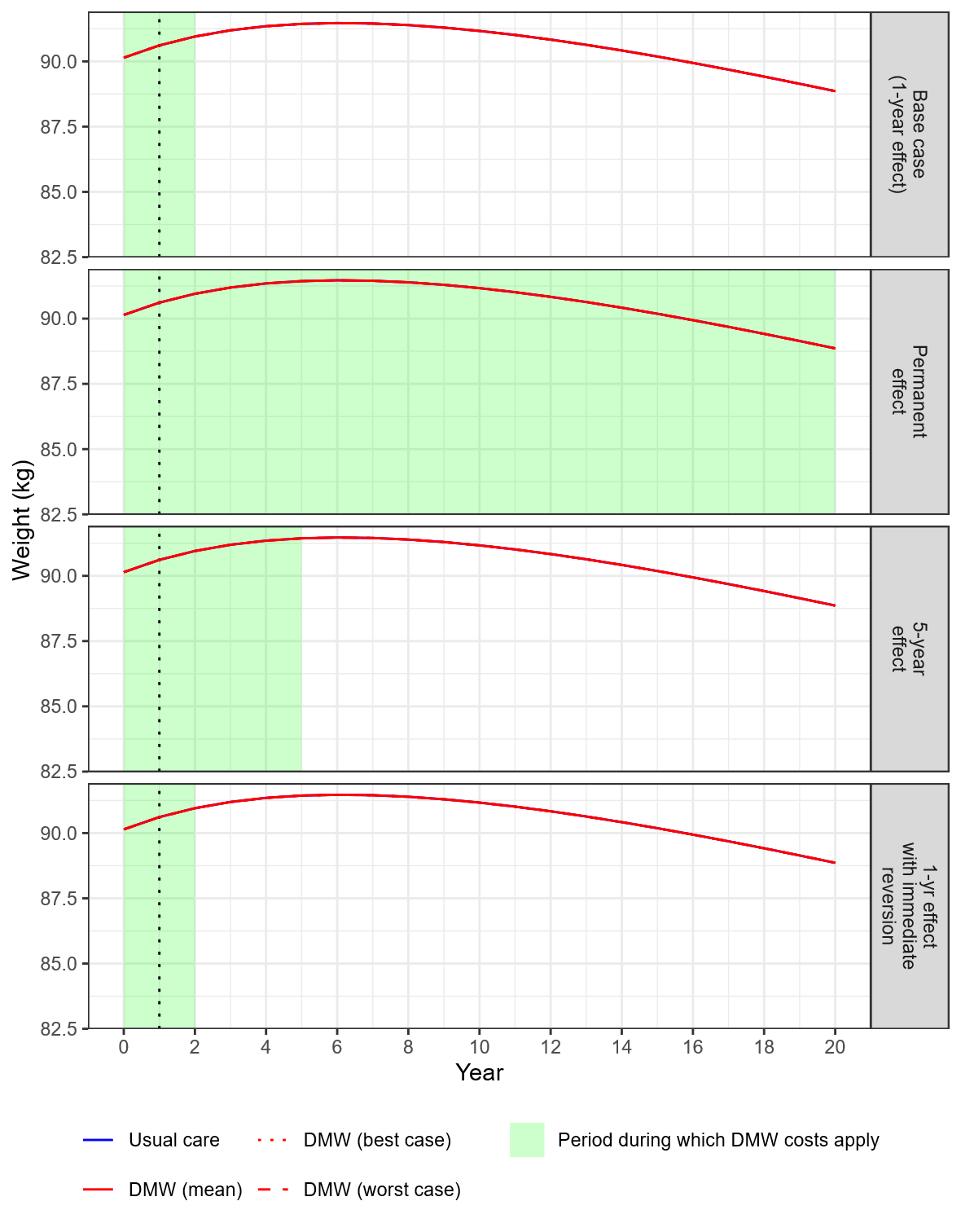 |
| --- |

## Base-case population (weighted average of NICE cohorts)

### Expected events

Table e4.01: Expected events per 100,000 people with type 2 diabetes over 10 years (base-case)

| Event | Usual care | DMW (WCS / BCS) | Difference (WCS / BCS) | NNT (WCS / BCS) |
| --- | --- | --- | --- | --- |
| All death | 28,815 | 28,802 (28,793 / 28,810) | −13 (−22 / −5) | 660 (405 / 1,768) |
| Amputation | 1,783 | 1,779 (1,777 / 1,781) | −4 (−6 / −2) | 2,295 (1,491 / 4,404) |
| Blindness | 4,049 | 4,042 (4,039 / 4,046) | −7 (−10 / −3) | 1,360 (898 / 2,887) |
| Heart failure | 6,546 | 6,543 (6,541 / 6,545) | −3 (−5 / −1) | 3,019 (1,792 / 7,402) |
| IHD | 6,684 | 6,679 (6,676 / 6,683) | −5 (−8 / −1) | 2,029 (1,082 / 16,728) |
| MI | 12,307 | 12,290 (12,279 / 12,301) | −17 (−28 / −6) | 520 (322 / 1,414) |
| Renal failure | 579 | 579 (578 / 579) | 0 (−1 / 0) | 11,086 (7,079 / 43,139) |
| Stroke | 7,513 | 7,501 (7,493 / 7,509) | −12 (−20 / −4) | 754 (452 / 2,264) |
| Ulcer | 2,360 | 2,357 (2,355 / 2,359) | −3 (−5 / −1) | 2,678 (1,768 / 4,842) |

**Abbreviations**: BCS Best-Case Scenario; DMW Diabetes MyWay; IHD Ischaemic Heart Disease; MI myocardial infarction; WCS Worst-Case Scenario. Notes: WCS and BCS do not constitute confidence intervals. They represent the worst case and best case magnitude of the treatment effects for the examined clinical risk factors.

Table e4.02: Expected events per 100,000 people with type 2 diabetes over 10 years (scenarios)

| Scenario (see e4.1) | Event | Usual care | DMW (WCS / BCS) | Difference (WCS / BCS) | NNT (WCS / BCS) |
| --- | --- | --- | --- | --- | --- |
| Permanent costs & effects | All death | 28,815 | 28,764 (28,730 / 28,799) | −51 (−85 / −16) | 175 (105 / 570) |
|  | Amputation | 1,783 | 1,768 (1,761 / 1,776) | −15 (−22 / −7) | 603 (400 / 1,311) |
|  | Blindness | 4,049 | 4,032 (4,023 / 4,041) | −17 (−26 / −8) | 523 (342 / 1,116) |
|  | Heart failure | 6,546 | 6,532 (6,521 / 6,542) | −14 (−25 / −4) | 619 (358 / 2,288) |
|  | IHD | 6,684 | 6,666 (6,650 / 6,682) | −18 (−34 / −2) | 492 (264 / 5,394) |
|  | MI | 12,307 | 12,254 (12,220 / 12,290) | −53 (−87 / −17) | 169 (103 / 520) |
|  | Renal failure | 579 | 576 (574 / 579) | −3 (−5 / 0) | 2,733 (1,584 / 10,844) |
|  | Stroke | 7,513 | 7,472 (7,446 / 7,500) | −41 (−67 / −13) | 219 (132 / 705) |
|  | Ulcer | 2,360 | 2,351 (2,346 / 2,356) | −9 (−14 / −4) | 945 (637 / 1,937) |
| 5-year costs & effects | All death | 28,815 | 28,782 (28,761 / 28,804) | −33 (−54 / −11) | 268 (164 / 847) |
|  | Amputation | 1,783 | 1,773 (1,768 / 1,779) | −10 (−15 / −4) | 901 (611 / 2,016) |
|  | Blindness | 4,049 | 4,036 (4,029 / 4,043) | −13 (−20 / −6) | 672 (445 / 1,444) |
|  | Heart failure | 6,546 | 6,537 (6,531 / 6,543) | −9 (−15 / −3) | 1,019 (599 / 3,494) |
|  | IHD | 6,684 | 6,672 (6,662 / 6,683) | −12 (−22 / −1) | 746 (400 / 8,840) |
|  | MI | 12,307 | 12,269 (12,245 / 12,294) | −38 (−62 / −13) | 234 (144 / 700) |
|  | Renal failure | 579 | 577 (576 / 579) | −2 (−3 / 0) | 4,113 (2,508 / 15,819) |
|  | Stroke | 7,513 | 7,485 (7,466 / 7,504) | −28 (−47 / −9) | 313 (189 / 981) |
|  | Ulcer | 2,360 | 2,353 (2,350 / 2,357) | −7 (−10 / −3) | 1,278 (868 / 2,537) |
| 1-year effects  with immediate reversion; 2-year costs | All death | 28,815 | 28,811 (28,809 / 28,814) | −4 (−6 / −1) | 2,430 (1,523 / 8,463) |
|  | Amputation | 1,783 | 1,782 (1,782 / 1,783) | −1 (−1 / 0) | 12,904 (10,198 / 41,041) |
|  | Blindness | 4,049 | 4,047 (4,046 / 4,048) | −2 (−3 / −1) | 4,798 (3,217 / 11,690) |
|  | Heart failure | 6,546 | 6,545 (6,545 / 6,546) | −1 (−1 / 0) | 19,372 (7,062 / −158,378) |
|  | IHD | 6,684 | 6,682 (6,681 / 6,684) | −2 (−3 / 0) | 6,070 (3,291 / 68,041) |
|  | MI | 12,307 | 12,302 (12,298 / 12,305) | −5 (−9 / −2) | 1,706 (1,038 / 5,176) |
|  | Renal failure | 579 | 579 (579 / 580) | 0 (0 / 1) | 1,801,802 (35,746 / −79,580) |
|  | Stroke | 7,513 | 7,510 (7,508 / 7,512) | −3 (−5 / −1) | 3,314 (1,759 / 15,682) |
|  | Ulcer | 2,360 | 2,360 (2,359 / 2,360) | 0 (−1 / 0) | 9,956 (6,698 / 19,879) |

**Abbreviations**: BCS Best-Case Scenario; DMW Diabetes MyWay; IHD Ischaemic Heart Disease; MI myocardial infarction; WCS Worst-Case Scenario. Notes: WCS and BCS do not constitute confidence intervals. They represent the worst case and best case magnitude of the treatment effects for the examined clinical risk factors.

### Cost per QALY (patient level)

Table e4.03: Cost per QALY (patient level; discounted; 40-yr time horizon; base case)

| Outcome | Usual care | DMW (WCS / BCS) | Difference (WCS / BCS) |
| --- | --- | --- | --- |
| LYs | 12·392 | 12·409 (12·399 / 12·421) | 0·017 (0·006 / 0·028) |
| QALYs | 9·875 | 9·890 (9·881 / 9·901) | 0·015 (0·006 / 0·026) |
| Costs |  |  |  |
| DMW intervention | - | 38 (38 / 38) | 38 (38 / 38) |
| Diabetes medicines | 1,548 | 1,475 (1,474 / 1,476) | −73 (−74 / −72) |
| Long-term complications | 34,527 | 34,495 (34,519 / 34,475) | −32 (−8 / −52) |
| Total cost | 36,075 | 36,007 (36,030 / 35,988) | −67 (−45 / −86) |
| Cost per QALY | - | - | Dominant (Dominant / Dominant) |
| Cost per QALY (excluding medicines) | - | - | 389 (4,898 / Dominant) |

**Abbreviations**: BCS Best-Case Scenario; DMW Diabetes MyWay; LY Life-Year; QALY Quality-Adjusted Life-Year; WCS Worst-Case Scenario. Notes: WCS and BCS do not constitute confidence intervals. They represent the worst case and best case magnitude of the treatment effects for the examined clinical risk factors.

Table e4.04: Cost per QALY (patient level; discounted; 40-yr time horizon; scenarios)

| Scenario | Outcome | Usual care | DMW (WCS / BCS) | Difference (WCS / BCS) |
| --- | --- | --- | --- | --- |
| Permanent costs & effects | LYs | 12·517 | 12·634 (12·551 / 12·715) | 0·118 (0·034 / 0·198) |
|  | QALYs | 9·978 | 10·082 (10·009 / 10·152) | 0·104 (0·031 / 0·174) |
|  | Costs |  |  |  |
|  | DMW intervention | - | 250 (249 / 252) | 250 (249 / 252) |
|  | Diabetes medicines | 1,560 | 1,076 (1,069 / 1,082) | −484 (−491 / −478) |
|  | Long-term complications | 34,686 | 34,579 (34,655 / 34,495) | −107 (−31 / −191) |
|  | Total cost | 36,246 | 35,905 (35,972 / 35,829) | −341 (−274 / −417) |
|  | Cost per QALY | - | - | Dominant (Dominant / Dominant) |
|  | Cost per QALY (exc. meds) | - | - | 1,381 (6,979 / 349) |
| 5-year costs & effects | LYs | 12·517 | 12·557 (12·530 / 12·582) | 0·040 (0·013 / 0·066) |
|  | QALYs | 9·978 | 10·015 (9·990 / 10·038) | 0·037 (0·012 / 0·059) |
|  | Costs |  |  |  |
|  | DMW intervention | - | 86 (86 / 86) | 86 (86 / 86) |
|  | Diabetes medicines | 1,560 | 1,393 (1,390 / 1,396) | −167 (−171 / −164) |
|  | Long-term complications | 34,686 | 34,628 (34,669 / 34,579) | −58 (−17 / −107) |
|  | Total cost | 36,246 | 36,107 (36,145 / 36,061) | −139 (−101 / −185) |
|  | Cost per QALY | - | - | Dominant (Dominant / Dominant) |
|  | Cost per QALY (exc. meds) | - | - | 781 (5,768 / Dominant) |
| 1-year effects  with immediate reversion; 2-year costs | LYs | 12·517 | 12·528 (12·521 / 12·532) | 0·011 (0·004 / 0·015) |
|  | QALYs | 9·978 | 9·988 (9·982 / 9·992) | 0·010 (0·004 / 0·014) |
|  | Costs |  |  |  |
|  | DMW intervention | - | 38 (38 / 38) | 38 (38 / 38) |
|  | Diabetes medicines | 1,560 | 1,486 (1,486 / 1,487) | −74 (−75 / −73) |
|  | Long-term complications | 34,686 | 34,666 (34,678 / 34,656) | −21 (−8 / −30) |
|  | Total cost | 36,246 | 36,190 (36,201 / 36,180) | −57 (−45 / −66) |
|  | Cost per QALY | - | - | Dominant (Dominant / Dominant) |
|  | Cost per QALY (exc. meds) | - | - | 1,763 (7,556 / 531) |

**Abbreviations**: BCS Best-Case Scenario; DMW Diabetes MyWay; LY Life-Year; QALY Quality-Adjusted Life-Year; WCS Worst-Case Scenario. Notes: WCS and BCS do not constitute confidence intervals. They represent the worst case and best case magnitude of the treatment effects for the examined clinical risk factors.

Table e4.05: Cost per QALY (patient level; discounted; 10-yr time horizon)

| Scenario | Outcome | Usual care | DMW (WCS / BCS) | Difference (WCS / BCS) |
| --- | --- | --- | --- | --- |
| 1-year effects & 2-year costs (base case) | LYs | 7·532 | 7·540 (7·535 / 7·546) | 0·009 (0·003 / 0·014) |
|  | QALYs | 6·063 | 6·071 (6·066 / 6·076) | 0·008 (0·003 / 0·014) |
|  | Costs |  |  |  |
|  | DMW intervention | - | 38 (38 / 38) | 38 (38 / 38) |
|  | Diabetes medicines | 1,548 | 1,475 (1,474 / 1,476) | −73 (−74 / −72) |
|  | Long-term complications | 17,858 | 17,818 (17,846 / 17,791) | −40 (−12 / −67) |
|  | Total cost | 19,406 | 19,330 (19,358 / 19,305) | −76 (−49 / −101) |
|  | Cost per QALY | - | - | Dominant (Dominant / Dominant) |
|  | Cost per QALY (exc. meds) | - | - | Dominant (8,782 / Dominant) |
| Permanent costs & effects | LYs | 7·532 | 7·551 (7·538 / 7·564) | 0·019 (0·006 / 0·032) |
|  | QALYs | 6·063 | 6·081 (6·069 / 6·093) | 0·019 (0·006 / 0·031) |
|  | Costs |  |  |  |
|  | DMW intervention | - | 149 (148 / 149) | 149 (148 / 149) |
|  | Diabetes medicines | 1,548 | 1,266 (1,256 / 1,276) | −282 (−292 / −272) |
|  | Long-term complications | 17,858 | 17,736 (17,822 / 17,653) | −122 (−36 / −205) |
|  | Total cost | 19,406 | 19,150 (19,227 / 19,078) | −256 (−180 / −329) |
|  | Cost per QALY | - | - | Dominant (Dominant / Dominant) |
|  | Cost per QALY (exc. meds) | - | - | 1,407 (19,124 / Dominant) |
| 5-year costs & effects | LYs | 7·532 | 7·548 (7·537 / 7·559) | 0·017 (0·005 / 0·027) |
|  | QALYs | 6·063 | 6·079 (6·068 / 6·089) | 0·016 (0·005 / 0·026) |
|  | Costs |  |  |  |
|  | DMW intervention | - | 86 (86 / 86) | 86 (86 / 86) |
|  | Diabetes medicines | 1,548 | 1,381 (1,377 / 1,384) | −167 (−171 / −164) |
|  | Long-term complications | 17,858 | 17,766 (17,831 / 17,705) | −93 (−28 / −154) |
|  | Total cost | 19,406 | 19,233 (19,294 / 19,175) | −174 (−112 / −232) |
|  | Cost per QALY | - | - | Dominant (Dominant / Dominant) |
|  | Cost per QALY (exc. meds) | - | - | Dominant (11,476 / Dominant) |
| 1-year effects  with immediate reversion; 2-year costs | LYs | 7·532 | 7·535 (7·533 / 7·537) | 0·003 (0·001 / 0·005) |
|  | QALYs | 6·063 | 6·066 (6·064 / 6·068) | 0·003 (0·001 / 0·005) |
|  | Costs |  |  |  |
|  | DMW intervention | - | 38 (38 / 38) | 38 (38 / 38) |
|  | Diabetes medicines | 1,548 | 1,473 (1,473 / 1,474) | −75 (−75 / −74) |
|  | Long-term complications | 17,858 | 17,850 (17,859 / 17,839) | −8 (0 / −19) |
|  | Total cost | 19,406 | 19,361 (19,369 / 19,350) | −45 (−37 / −56) |
|  | Cost per QALY | - | - | Dominant (Dominant / Dominant) |
|  | Cost per QALY (exc. meds) | - | - | 9,803 (40,600 / 3,774) |

**Abbreviations**: BCS Best-Case Scenario; DMW Diabetes MyWay; LY Life-Year; QALY Quality-Adjusted Life-Year; WCS Worst-Case Scenario. Notes: WCS and BCS do not constitute confidence intervals. They represent the worst case and best case magnitude of the treatment effects for the examined clinical risk factors.

Table e4.06: Cost per QALY (patient level; discounted; 5-yr time horizon)

| Scenario | Outcome | Usual care | DMW (WCS / BCS) | Difference (WCS / BCS) |
| --- | --- | --- | --- | --- |
| 1-year effects & 2-year costs (base case) | LYs | 4·399 | 4·401 (4·399 / 4·403) | 0·003 (0·001 / 0·004) |
|  | QALYs | 3·558 | 3·560 (3·559 / 3·562) | 0·003 (0·001 / 0·005) |
|  | Costs |  |  |  |
|  | DMW intervention | - | 38 (38 / 38) | 38 (38 / 38) |
|  | Diabetes medicines | 1,548 | 1,475 (1,474 / 1,476) | −73 (−74 / −72) |
|  | Long-term complications | 9,661 | 9,628 (9,651 / 9,606) | −33 (−10 / −54) |
|  | Total cost | 11,209 | 11,141 (11,162 / 11,120) | −68 (−47 / −88) |
|  | Cost per QALY | - | - | Dominant (Dominant / Dominant) |
|  | Cost per QALY (exc. meds) | - | - | 1,791 (29,305 / Dominant) |
| Permanent costs & effects | LYs | 4·399 | 4·402 (4·400 / 4·405) | 0·004 (0·001 / 0·006) |
|  | QALYs | 3·558 | 3·562 (3·559 / 3·564) | 0·004 (0·001 / 0·006) |
|  | Costs |  |  |  |
|  | DMW intervention | - | 86 (86 / 86) | 86 (86 / 86) |
|  | Diabetes medicines | 1,548 | 1,390 (1,380 / 1,400) | −158 (−168 / −148) |
|  | Long-term complications | 9,661 | 9,607 (9,645 / 9,571) | −54 (−16 / −89) |
|  | Total cost | 11,209 | 11,084 (11,111 / 11,058) | −125 (−97 / −151) |
|  | Cost per QALY | - | - | Dominant (Dominant / Dominant) |
|  | Cost per QALY (exc. meds) | - | - | 8,509 (56,802 / Dominant) |
| 5-year costs & effects | LYs | 4·399 | 4·402 (4·400 / 4·405) | 0·004 (0·001 / 0·006) |
|  | QALYs | 3·558 | 3·562 (3·559 / 3·564) | 0·004 (0·001 / 0·006) |
|  | Costs |  |  |  |
|  | DMW intervention | - | 86 (86 / 86) | 86 (86 / 86) |
|  | Diabetes medicines | 1,548 | 1,381 (1,377 / 1,384) | −167 (−171 / −164) |
|  | Long-term complications | 9,661 | 9,607 (9,645 / 9,572) | −54 (−16 / −89) |
|  | Total cost | 11,209 | 11,074 (11,108 / 11,041) | −135 (−100 / −167) |
|  | Cost per QALY | - | - | Dominant (Dominant / Dominant) |
|  | Cost per QALY (exc. meds) | - | - | 8,482 (57,449 / Dominant) |
| 1-year effects  with immediate reversion; 2-year costs | LYs | 4·399 | 4·400 (4·399 / 4·401) | 0·001 (0·000 / 0·002) |
|  | QALYs | 3·558 | 3·559 (3·558 / 3·560) | 0·001 (0·000 / 0·002) |
|  | Costs |  |  |  |
|  | DMW intervention | - | 38 (38 / 38) | 38 (38 / 38) |
|  | Diabetes medicines | 1,548 | 1,473 (1,473 / 1,474) | −75 (−75 / −74) |
|  | Long-term complications | 9,661 | 9,649 (9,658 / 9,640) | −12 (−2 / −21) |
|  | Total cost | 11,209 | 11,160 (11,169 / 11,151) | −49 (−40 / −57) |
|  | Cost per QALY | - | - | Dominant (Dominant / Dominant) |
|  | Cost per QALY (exc. meds) | - | - | 18,135 (74,353 / 7,163) |

**Abbreviations**: BCS Best-Case Scenario; DMW Diabetes MyWay; LY Life-Year; QALY Quality-Adjusted Life-Year; WCS Worst-Case Scenario. Notes: WCS and BCS do not constitute confidence intervals. They represent the worst case and best case magnitude of the treatment effects for the examined clinical risk factors.

### Cost per QALY (Greater Manchester population level)

Table e4.07: Cost per QALY (population level; discounted; 40-yr time horizon; base case)

| Outcome | Usual care | DMW (WCS / BCS) | Difference (WCS / BCS) |
| --- | --- | --- | --- |
| LYs | 2,255,740 | 2,256,010 (2,255,845 / 2,256,200) | 270 (105 / 460) |
| QALYs | 1,797,514 | 1,797,763 (1,797,612 / 1,797,937) | 249 (98 / 423) |
| Costs |  |  |  |
| DMW intervention | - | 609,637 (609,639 / 609,639) | 609,637 (609,639 / 609,639) |
| Diabetes medicines | 281,781,720 | 280,596,980 (280,577,173 / 280,619,747) | −1,184,741 (−1,204,548 / −1,161,973) |
| Long-term complications | 6,284,692,238 | 6,284,179,431 (6,284,562,153 / 6,283,850,129) | −512,807 (−130,085 / −842,109) |
| Total cost | 6,566,473,959 | 6,565,386,048 (6,565,748,965 / 6,565,079,515) | −1,087,911 (−724,994 / −1,394,444) |
| Cost per QALY | - | - | Dominant (Dominant / Dominant) |
| Cost per QALY (exc. meds) | - | - | 389 (4,898 / Dominant) |

**Abbreviations**: BCS Best-Case Scenario; DMW Diabetes MyWay; LY Life-Year; QALY Quality-Adjusted Life-Year; WCS Worst-Case Scenario. Notes: WCS and BCS do not constitute confidence intervals. They represent the worst case and best case magnitude of the treatment effects for the examined clinical risk factors.

Table e4.08: Cost per QALY (population level; discounted; 40-yr time horizon; scenarios)

| Scenario | Outcome | Usual care | DMW (WCS / BCS) | Difference (WCS / BCS) |
| --- | --- | --- | --- | --- |
| Permanent costs & effects | LYs | 2,255,740 | 2,257,693 (2,256,352 / 2,259,035) | 1,953 (612 / 3,295) |
|  | QALYs | 1,797,514 | 1,799,236 (1,798,065 / 1,800,409) | 1,723 (551 / 2,895) |
|  | Costs |  |  |  |
|  | DMW intervention | - | 4,013,268 (3,987,841 / 4,038,714) | 4,013,268 (3,987,841 / 4,038,714) |
|  | Diabetes medicines | 281,781,720 | 274,004,113 (273,893,576 / 274,114,756) | −7,777,607 (−7,888,145 / −7,666,965) |
|  | Long-term complications | 6,284,692,238 | 6,282,739,216 (6,284,196,047 / 6,281,221,197) | −1,953,023 (−496,191 / −3,471,042) |
|  | Total cost | 6,566,473,959 | 6,560,756,597 (6,562,077,464 / 6,559,374,667) | −5,717,362 (−4,396,494 / −7,099,292) |
|  | Cost per QALY | - | - | Dominant (Dominant / Dominant) |
|  | Cost per QALY (excluding medicines) | - | - | 1,196 (6,339 / 196) |
| 5-year costs & effects | LYs | 2,255,740 | 2,256,408 (2,255,962 / 2,256,822) | 668 (222 / 1,082) |
|  | QALYs | 1,797,514 | 1,798,123 (1,797,720 / 1,798,500) | 610 (206 / 986) |
|  | Costs |  |  |  |
|  | DMW intervention | - | 1,398,578 (1,398,009 / 1,399,127) | 1,398,578 (1,398,009 / 1,399,127) |
|  | Diabetes medicines | 281,781,720 | 279,069,532 (279,017,209 / 279,118,126) | −2,712,188 (−2,764,512 / −2,663,595) |
|  | Long-term complications | 6,284,692,238 | 6,283,468,789 (6,284,373,710 / 6,282,641,390) | −1,223,450 (−318,529 / −2,050,848) |
|  | Total cost | 6,566,473,959 | 6,563,936,899 (6,564,788,928 / 6,563,158,643) | −2,537,060 (−1,685,031 / −3,315,316) |
|  | Cost per QALY | - | - | Dominant (Dominant / Dominant) |
|  | Cost per QALY (excluding medicines) | - | - | 287 (5,231 / Dominant) |
| 1-year effects  with immediate reversion; 2-year costs | LYs | 2,255,740 | 2,255,811 (2,255,762 / 2,255,873) | 71 (22 / 133) |
|  | QALYs | 1,797,514 | 1,797,578 (1,797,533 / 1,797,635) | 64 (19 / 122) |
|  | Costs |  |  |  |
|  | DMW intervention | - | 609,639 (609,638 / 609,636) | 609,639 (609,638 / 609,636) |
|  | Diabetes medicines | 281,781,720 | 280,573,051 (280,567,245 / 280,580,484) | −1,208,670 (−1,214,476 / −1,201,237) |
|  | Long-term complications | 6,284,692,238 | 6,284,652,783 (6,284,794,175 / 6,284,438,670) | −39,456 (101,936 / −253,569) |
|  | Total cost | 6,566,473,959 | 6,565,835,473 (6,565,971,058 / 6,565,628,790) | −638,486 (−502,901 / −845,169) |
|  | Cost per QALY | - | - | Dominant (Dominant / Dominant) |
|  | Cost per QALY (excluding medicines) | - | - | 8,899 (36,516 / 2,930) |

**Abbreviations**: BCS Best-Case Scenario; DMW Diabetes MyWay; LY Life-Year; QALY Quality-Adjusted Life-Year; WCS Worst-Case Scenario. Notes: WCS and BCS do not constitute confidence intervals. They represent the worst case and best case magnitude of the treatment effects for the examined clinical risk factors.

Table e4.09: Cost per QALY (population level; discounted; 10-yr time horizon)

| Scenario | Outcome | Usual care | DMW (WCS / BCS) | Difference (WCS / BCS) |
| --- | --- | --- | --- | --- |
| 1-year effects & 2-year costs (base case) | LYs | 1,370,998 | 1,371,136 (1,371,046 / 1,371,225) | 138 (47 / 226) |
|  | QALYs | 1,103,572 | 1,103,705 (1,103,619 / 1,103,791) | 134 (47 / 219) |
|  | Costs |  |  |  |
|  | DMW intervention | - | 609,637 (609,639 / 609,639) | 609,637 (609,639 / 609,639) |
|  | Diabetes medicines | 281,781,720 | 280,596,980 (280,577,173 / 280,619,747) | −1,184,741 (−1,204,548 / −1,161,973) |
|  | Long-term complications | 3,250,671,345 | 3,250,016,618 (3,250,477,121 / 3,249,584,923) | −654,727 (−194,225 / −1,086,422) |
|  | Total cost | 3,532,453,066 | 3,531,223,235 (3,531,663,932 / 3,530,814,309) | −1,229,831 (−789,133 / −1,638,757) |
|  | Cost per QALY | - | - | Dominant (Dominant / Dominant) |
|  | Cost per QALY (excluding medicines) | - | - | Dominant (8,782 / Dominant) |
| Permanent costs & effects | LYs | 1,370,998 | 1,371,306 (1,371,092 / 1,371,511) | 308 (94 / 512) |
|  | QALYs | 1,103,572 | 1,103,872 (1,103,666 / 1,104,068) | 300 (95 / 496) |
|  | Costs |  |  |  |
|  | DMW intervention | - | 2,405,285 (2,401,656 / 2,408,741) | 2,405,285 (2,401,656 / 2,408,741) |
|  | Diabetes medicines | 281,781,720 | 277,214,455 (277,060,398 / 277,369,001) | −4,567,265 (−4,721,322 / −4,412,719) |
|  | Long-term complications | 3,250,671,345 | 3,248,688,570 (3,250,082,654 / 3,247,348,864) | −1,982,775 (−588,691 / −3,322,482) |
|  | Total cost | 3,532,453,066 | 3,528,308,310 (3,529,544,708 / 3,527,126,605) | −4,144,755 (−2,908,357 / −5,326,461) |
|  | Cost per QALY | - | - | Dominant (Dominant / Dominant) |
|  | Cost per QALY (excluding medicines) | - | - | 1,407 (19,124 / Dominant) |
| 5-year costs & effects | LYs | 1,370,998 | 1,371,266 (1,371,080 / 1,371,441) | 267 (82 / 443) |
|  | QALYs | 1,103,572 | 1,103,832 (1,103,654 / 1,104,000) | 260 (83 / 428) |
|  | Costs |  |  |  |
|  | DMW intervention | - | 1,398,578 (1,398,009 / 1,399,127) | 1,398,578 (1,398,009 / 1,399,127) |
|  | Diabetes medicines | 281,781,720 | 279,069,532 (279,017,209 / 279,118,126) | −2,712,188 (−2,764,512 / −2,663,595) |
|  | Long-term complications | 3,250,671,345 | 3,249,168,737 (3,250,222,067 / 3,248,184,116) | −1,502,608 (−449,279 / −2,487,229) |
|  | Total cost | 3,532,453,066 | 3,529,636,847 (3,530,637,285 / 3,528,701,369) | −2,816,218 (−1,815,781 / −3,751,697) |
|  | Cost per QALY | - | - | Dominant (Dominant / Dominant) |
|  | Cost per QALY (excluding medicines) | - | - | Dominant (11,476 / Dominant) |
| 1-year effects  with immediate reversion; 2-year costs | LYs | 1,370,998 | 1,371,050 (1,371,014 / 1,371,080) | 51 (16 / 82) |
|  | QALYs | 1,103,572 | 1,103,620 (1,103,587 / 1,103,650) | 48 (15 / 78) |
|  | Costs |  |  |  |
|  | DMW intervention | - | 609,639 (609,638 / 609,636) | 609,639 (609,638 / 609,636) |
|  | Diabetes medicines | 281,781,720 | 280,573,051 (280,567,245 / 280,580,484) | −1,208,670 (−1,214,476 / −1,201,237) |
|  | Long-term complications | 3,250,671,345 | 3,250,536,040 (3,250,677,584 / 3,250,357,010) | −135,305 (6,238 / −314,335) |
|  | Total cost | 3,532,453,066 | 3,531,718,730 (3,531,854,467 / 3,531,547,130) | −734,336 (−598,599 / −905,936) |
|  | Cost per QALY | - | - | Dominant (Dominant / Dominant) |
|  | Cost per QALY (excluding medicines) | - | - | 9,803 (40,600 / 3,774) |

**Abbreviations**: BCS Best-Case Scenario; DMW Diabetes MyWay; LY Life-Year; QALY Quality-Adjusted Life-Year; WCS Worst-Case Scenario. Notes: WCS and BCS do not constitute confidence intervals. They represent the worst case and best case magnitude of the treatment effects for the examined clinical risk factors.

Table e4.10: Cost per QALY (population level; discounted; 5-yr time horizon)

| Scenario | Outcome | Usual care | DMW (WCS / BCS) | Difference (WCS / BCS) |
| --- | --- | --- | --- | --- |
| 1-year effects & 2-year costs (base case) | LYs | 800,642 | 800,686 (800,657 / 800,714) | 43 (14 / 72) |
|  | QALYs | 647,587 | 647,632 (647,602 / 647,662) | 45 (15 / 75) |
|  | Costs |  |  |  |
|  | DMW intervention | - | 609,637 (609,639 / 609,639) | 609,637 (609,639 / 609,639) |
|  | Diabetes medicines | 281,781,720 | 280,596,980 (280,577,173 / 280,619,747) | −1,184,741 (−1,204,548 / −1,161,973) |
|  | Long-term complications | 1,758,480,271 | 1,757,951,677 (1,758,320,411 / 1,757,600,499) | −528,594 (−159,860 / −879,773) |
|  | Total cost | 2,040,261,992 | 2,039,158,294 (2,039,507,223 / 2,038,829,885) | −1,103,698 (−754,769 / −1,432,107) |
|  | Cost per QALY | - | - | Dominant (Dominant / Dominant) |
|  | Cost per QALY (excluding medicines) | - | - | 1,791 (29,305 / Dominant) |
| Permanent costs & effects | LYs | 800,642 | 800,701 (800,661 / 800,740) | 59 (18 / 98) |
|  | QALYs | 647,587 | 647,649 (647,607 / 647,690) | 62 (20 / 103) |
|  | Costs |  |  |  |
|  | DMW intervention | - | 1,398,583 (1,398,014 / 1,399,127) | 1,398,583 (1,398,014 / 1,399,127) |
|  | Diabetes medicines | 281,781,720 | 279,224,337 (279,064,172 / 279,384,696) | −2,557,384 (−2,717,549 / −2,397,024) |
|  | Long-term complications | 1,758,480,271 | 1,757,612,919 (1,758,223,085 / 1,757,034,681) | −867,352 (−257,187 / −1,445,590) |
|  | Total cost | 2,040,261,992 | 2,038,235,839 (2,038,685,270 / 2,037,818,505) | −2,026,152 (−1,576,722 / −2,443,487) |
|  | Cost per QALY | - | - | Dominant (Dominant / Dominant) |
|  | Cost per QALY (excluding medicines) | - | - | 8,509 (56,802 / Dominant) |
| 5-year costs & effects | LYs | 800,642 | 800,701 (800,660 / 800,740) | 59 (18 / 98) |
|  | QALYs | 647,587 | 647,649 (647,607 / 647,690) | 62 (20 / 103) |
|  | Costs |  |  |  |
|  | DMW intervention | - | 1,398,578 (1,398,009 / 1,399,127) | 1,398,578 (1,398,009 / 1,399,127) |
|  | Diabetes medicines | 281,781,720 | 279,069,532 (279,017,209 / 279,118,126) | −2,712,188 (−2,764,512 / −2,663,595) |
|  | Long-term complications | 1,758,480,271 | 1,757,608,764 (1,758,223,245 / 1,757,036,755) | −871,507 (−257,026 / −1,443,517) |
|  | Total cost | 2,040,261,992 | 2,038,076,875 (2,038,638,463 / 2,037,554,007) | −2,185,117 (−1,623,529 / −2,707,985) |
|  | Cost per QALY | - | - | Dominant (Dominant / Dominant) |
|  | Cost per QALY (excluding medicines) | - | - | 8,482 (57,449 / Dominant) |
| 1-year effects  with immediate reversion; 2-year costs | LYs | 800,642 | 800,665 (800,650 / 800,680) | 23 (8 / 37) |
|  | QALYs | 647,587 | 647,610 (647,595 / 647,625) | 23 (8 / 38) |
|  | Costs |  |  |  |
|  | DMW intervention | - | 609,639 (609,638 / 609,636) | 609,639 (609,638 / 609,636) |
|  | Diabetes medicines | 281,781,720 | 280,573,051 (280,567,245 / 280,580,484) | −1,208,670 (−1,214,476 / −1,201,237) |
|  | Long-term complications | 1,758,480,271 | 1,758,290,083 (1,758,440,680 / 1,758,142,581) | −190,188 (−39,592 / −337,690) |
|  | Total cost | 2,040,261,992 | 2,039,472,773 (2,039,617,563 / 2,039,332,701) | −789,219 (−644,429 / −929,291) |
|  | Cost per QALY | - | - | Dominant (Dominant / Dominant) |
|  | Cost per QALY (excluding medicines) | - | - | 18,135 (74,353 / 7,163) |

**Abbreviations**: BCS Best-Case Scenario; DMW Diabetes MyWay; LY Life-Year; QALY Quality-Adjusted Life-Year; WCS Worst-Case Scenario. Notes: WCS and BCS do not constitute confidence intervals. They represent the worst case and best case magnitude of the treatment effects for the examined clinical risk factors.

## Greater Manchester Diabetes My Way population

### Expected events

Table e4.11: Expected events per 100,000 people with type 2 diabetes over 10 years (base-case)

| Event | Usual care | DMW (WCS / BCS) | Difference (WCS / BCS) | NNT (WCS / BCS) |
| --- | --- | --- | --- | --- |
| All death | 17,604 | 17,594 (17,588 / 17,600) | −10 (−16 / −4) | 906 (578 / 2,284) |
| Amputation | 2,407 | 2,400 (2,397 / 2,404) | −7 (−10 / −3) | 1,275 (899 / 2,604) |
| Blindness | 1,986 | 1,983 (1,981 / 1,985) | −3 (−5 / −1) | 2,697 (1,780 / 5,696) |
| Heart failure | 4,093 | 4,090 (4,089 / 4,092) | −3 (−4 / −1) | 2,880 (1,872 / 5,753) |
| IHD | 5,811 | 5,807 (5,804 / 5,810) | −4 (−7 / −1) | 2,186 (1,171 / 13,355) |
| MI | 8,067 | 8,054 (8,047 / 8,062) | −13 (−20 / −5) | 697 (439 / 1,832) |
| Renal failure | 455 | 454 (454 / 455) | −1 (−1 / 0) | 12,090 (7,456 / 32,845) |
| Stroke | 4,615 | 4,608 (4,604 / 4,613) | −7 (−11 / −2) | 1,313 (810 / 3,673) |
| Ulcer | 1,620 | 1,617 (1,616 / 1,618) | −3 (−4 / −2) | 3,583 (2,491 / 6,375) |

**Abbreviations**: BCS Best-Case Scenario; DMW Diabetes MyWay; IHD Ischaemic Heart Disease; MI myocardial infarction; WCS Worst-Case Scenario. Notes: WCS and BCS do not constitute confidence intervals. They represent the worst case and best case magnitude of the treatment effects for the examined clinical risk factors.

Table e4.12: Expected events per 100,000 people with type 2 diabetes over 10 years (scenarios)

| Scenario (see e4.1) | Event | Usual care | DMW (WCS / BCS) | Difference (WCS / BCS) | NNT (WCS / BCS) |
| --- | --- | --- | --- | --- | --- |
| Permanent costs & effects | All death | 17,604 | 17,566 (17,543 / 17,592) | −38 (−61 / −12) | 237 (145 / 723) |
|  | Amputation | 2,407 | 2,384 (2,373 / 2,397) | −23 (−34 / −10) | 394 (265 / 855) |
|  | Blindness | 1,986 | 1,977 (1,972 / 1,982) | −9 (−14 / −4) | 973 (639 / 2,066) |
|  | Heart failure | 4,093 | 4,082 (4,074 / 4,090) | −11 (−19 / −3) | 759 (459 / 2,480) |
|  | IHD | 5,811 | 5,794 (5,780 / 5,809) | −17 (−31 / −2) | 529 (283 / 4,921) |
|  | MI | 8,067 | 8,027 (8,002 / 8,054) | −40 (−65 / −13) | 221 (136 / 675) |
|  | Renal failure | 455 | 452 (450 / 454) | −3 (−5 / −1) | 2,716 (1,613 / 9,623) |
|  | Stroke | 4,615 | 4,592 (4,577 / 4,607) | −23 (−38 / −8) | 380 (233 / 1,176) |
|  | Ulcer | 1,620 | 1,612 (1,609 / 1,616) | −8 (−11 / −4) | 1,230 (829 / 2,524) |
| 5-year costs & effects | All death | 17,604 | 17,579 (17,564 / 17,596) | −25 (−40 / −8) | 362 (224 / 1,086) |
|  | Amputation | 2,407 | 2,392 (2,384 / 2,400) | −15 (−23 / −7) | 574 (384 / 1,198) |
|  | Blindness | 1,986 | 1,979 (1,976 / 1,983) | −7 (−10 / −3) | 1,293 (847 / 2,740) |
|  | Heart failure | 4,093 | 4,086 (4,081 / 4,091) | −7 (−12 / −2) | 1,178 (725 / 3,490) |
|  | IHD | 5,811 | 5,800 (5,790 / 5,810) | −11 (−21 / −1) | 807 (422 / 6,484) |
|  | MI | 8,067 | 8,038 (8,020 / 8,057) | −29 (−47 / −10) | 309 (190 / 900) |
|  | Renal failure | 455 | 453 (452 / 454) | −2 (−3 / −1) | 4,652 (2,697 / 14,702) |
|  | Stroke | 4,615 | 4,599 (4,589 / 4,610) | −16 (−26 / −5) | 549 (336 / 1,668) |
|  | Ulcer | 1,620 | 1,614 (1,612 / 1,617) | −6 (−8 / −3) | 1,697 (1,126 / 3,258) |
| 1-year effects  with immediate reversion; 2-year costs | All death | 17,604 | 17,601 (17,601 / 17,603) | −3 (−3 / −1) | 2,921 (3,283 / 6,790) |
|  | Amputation | 2,407 | 2,405 (2,406 / 2,406) | −2 (−1 / −1) | 5,133 (9,575 / 9,302) |
|  | Blindness | 1,986 | 1,985 (1,985 / 1,986) | −1 (−1 / 0) | 10,095 (6,640 / 22,228) |
|  | Heart failure | 4,093 | 4,092 (4,093 / 4,093) | −1 (0 / 0) | 8,990 (22,924 / 15,322) |
|  | IHD | 5,811 | 5,810 (5,809 / 5,811) | −1 (−2 / 0) | 7,364 (4,237 / 50,883) |
|  | MI | 8,067 | 8,063 (8,061 / 8,065) | −4 (−6 / −2) | 2,113 (1,568 / 4,927) |
|  | Renal failure | 455 | 455 (455 / 455) | 0 (0 / 0) | −56,180 (−20,837 / −67,060) |
|  | Stroke | 4,615 | 4,613 (4,612 / 4,614) | −2 (−3 / −1) | 4,513 (3,412 / 14,674) |
|  | Ulcer | 1,620 | 1,619 (1,619 / 1,619) | −1 (−1 / −1) | 11,125 (15,127 / 19,201) |

**Abbreviations**: BCS Best-Case Scenario; DMW Diabetes MyWay; IHD Ischaemic Heart Disease; MI myocardial infarction; WCS Worst-Case Scenario. Notes: WCS and BCS do not constitute confidence intervals. They represent the worst case and best case magnitude of the treatment effects for the examined clinical risk factors.

### Cost per QALY (patient level)

Table e4.13: Cost per QALY (patient level; discounted; 40-yr time horizon; base case)

| Outcome | Usual care | DMW (WCS / BCS) | Difference (WCS / BCS) |
| --- | --- | --- | --- |
| LYs | 14·914 | 14·929 (14·921 / 14·937) | 0·015 (0·007 / 0·023) |
| QALYs | 11·905 | 11·920 (11·912 / 11·928) | 0·015 (0·007 / 0·023) |
| Costs |  |  |  |
| DMW intervention | - | 38 (38 / 38) | 38 (38 / 38) |
| Diabetes medicines | 1,871 | 1,797 (1,796 / 1,798) | −74 (−76 / −74) |
| Long-term complications | 38,434 | 38,380 (38,418 / 38,350) | −55 (−16 / −85) |
| Total cost | 40,306 | 40,214 (40,252 / 40,185) | −91 (−54 / −120) |
| Cost per QALY | - | - | Dominant (Dominant / Dominant) |
| Cost per QALY (excluding medicines) | - | - | Dominant (3,372 / Dominant) |

**Abbreviations**: BCS Best-Case Scenario; DMW Diabetes MyWay; LY Life-Year; QALY Quality-Adjusted Life-Year; WCS Worst-Case Scenario. Notes: WCS and BCS do not constitute confidence intervals. They represent the worst case and best case magnitude of the treatment effects for the examined clinical risk factors.

Table e4.14: Cost per QALY (patient level; discounted; 40-yr time horizon; scenarios)

| Scenario | Outcome | Usual care | DMW (WCS / BCS) | Difference (WCS / BCS) |
| --- | --- | --- | --- | --- |
| Permanent costs & effects | LYs | 14·914 | 15·033 (14·954 / 15·110) | 0·119 (0·040 / 0·196) |
|  | QALYs | 11·905 | 12·014 (11·942 / 12·083) | 0·109 (0·037 / 0·178) |
|  | Costs |  |  |  |
|  | DMW intervention | - | 296 (294 / 297) | 296 (294 / 297) |
|  | Diabetes medicines | 1,871 | 1,289 (1,283 / 1,296) | −582 (−589 / −575) |
|  | Long-term complications | 38,434 | 38,172 (38,353 / 38,000) | −262 (−81 / −435) |
|  | Total cost | 40,306 | 39,757 (39,930 / 39,593) | −548 (−376 / −713) |
|  | Cost per QALY | - | - | Dominant (Dominant / Dominant) |
|  | Cost per QALY (exc. meds) | - | - | 306 (5,693 / Dominant) |
| 5-year costs & effects | LYs | 14·914 | 14·949 (14·927 / 14·971) | 0·035 (0·013 / 0·057) |
|  | QALYs | 11·905 | 11·938 (11·918 / 11·959) | 0·033 (0·013 / 0·054) |
|  | Costs |  |  |  |
|  | DMW intervention | - | 88 (88 / 88) | 88 (88 / 88) |
|  | Diabetes medicines | 1,871 | 1,698 (1,695 / 1,700) | −174 (−176 / −171) |
|  | Long-term complications | 38,434 | 38,330 (38,399 / 38,253) | −104 (−35 / −181) |
|  | Total cost | 40,306 | 40,116 (40,182 / 40,042) | −189 (−123 / −264) |
|  | Cost per QALY | - | - | Dominant (Dominant / Dominant) |
|  | Cost per QALY (exc. meds) | - | - | Dominant (4,037 / Dominant) |
| 1-year effects  with immediate reversion; 2-year costs | LYs | 14·914 | 14·918 (14·916 / 14·917) | 0·004 (0·002 / 0·003) |
|  | QALYs | 11·905 | 11·909 (11·907 / 11·908) | 0·004 (0·002 / 0·003) |
|  | Costs |  |  |  |
|  | DMW intervention | - | 38 (38 / 38) | 38 (38 / 38) |
|  | Diabetes medicines | 1,871 | 1,795 (1,795 / 1,795) | −76 (−76 / −76) |
|  | Long-term complications | 38,434 | 38,425 (38,439 / 38,421) | −9 (5 / −14) |
|  | Total cost | 40,306 | 40,259 (40,272 / 40,254) | −47 (−33 / −52) |
|  | Cost per QALY | - | - | Dominant (Dominant / Dominant) |
|  | Cost per QALY (exc. meds) | - | - | 6,977 (24,091 / 8,962) |

**Abbreviations**: BCS Best-Case Scenario; DMW Diabetes MyWay; LY Life-Year; QALY Quality-Adjusted Life-Year; WCS Worst-Case Scenario. Notes: WCS and BCS do not constitute confidence intervals. They represent the worst case and best case magnitude of the treatment effects for the examined clinical risk factors.

Table e4.15: Cost per QALY (patient level; discounted; 10-yr time horizon)

| Scenario | Outcome | Usual care | DMW (WCS / BCS) | Difference (WCS / BCS) |
| --- | --- | --- | --- | --- |
| 1-year effects & 2-year costs (base case) | LYs | 7·976 | 7·982 (7·978 / 7·985) | 0·006 (0·002 / 0·009) |
|  | QALYs | 6·442 | 6·448 (6·444 / 6·451) | 0·006 (0·002 / 0·010) |
|  | Costs |  |  |  |
|  | DMW intervention | - | 38 (38 / 38) | 38 (38 / 38) |
|  | Diabetes medicines | 1,871 | 1,797 (1,796 / 1,798) | −74 (−76 / −74) |
|  | Long-term complications | 16,444 | 16,400 (16,428 / 16,373) | −44 (−16 / −71) |
|  | Total cost | 18,315 | 18,234 (18,261 / 18,208) | −81 (−54 / −107) |
|  | Cost per QALY | - | - | Dominant (Dominant / Dominant) |
|  | Cost per QALY (exc. meds) | - | - | Dominant (9,663 / Dominant) |
| Permanent costs & effects | LYs | 7·976 | 7·990 (7·980 / 7·998) | 0·014 (0·004 / 0·022) |
|  | QALYs | 6·442 | 6·456 (6·446 / 6·464) | 0·014 (0·005 / 0·023) |
|  | Costs |  |  |  |
|  | DMW intervention | - | 156 (156 / 156) | 156 (156 / 156) |
|  | Diabetes medicines | 1,871 | 1,571 (1,561 / 1,580) | −300 (−310 / −291) |
|  | Long-term complications | 16,444 | 16,320 (16,404 / 16,239) | −124 (−40 / −205) |
|  | Total cost | 18,315 | 18,047 (18,121 / 17,975) | −269 (−194 / −340) |
|  | Cost per QALY | - | - | Dominant (Dominant / Dominant) |
|  | Cost per QALY (exc. meds) | - | - | 2,285 (24,370 / Dominant) |
| 5-year costs & effects | LYs | 7·976 | 7·988 (7·980 / 7·995) | 0·012 (0·004 / 0·019) |
|  | QALYs | 6·442 | 6·454 (6·446 / 6·461) | 0·012 (0·004 / 0·019) |
|  | Costs |  |  |  |
|  | DMW intervention | - | 88 (88 / 88) | 88 (88 / 88) |
|  | Diabetes medicines | 1,871 | 1,698 (1,695 / 1,700) | −174 (−176 / −171) |
|  | Long-term complications | 16,444 | 16,350 (16,412 / 16,287) | −93 (−32 / −156) |
|  | Total cost | 18,315 | 18,136 (18,195 / 18,076) | −179 (−120 / −239) |
|  | Cost per QALY | - | - | Dominant (Dominant / Dominant) |
|  | Cost per QALY (exc. meds) | - | - | Dominant (13,909 / Dominant) |
| 1-year effects  with immediate reversion; 2-year costs | LYs | 7·976 | 7·978 (7·977 / 7·979) | 0·002 (0·001 / 0·003) |
|  | QALYs | 6·442 | 6·444 (6·442 / 6·445) | 0·002 (0·001 / 0·003) |
|  | Costs |  |  |  |
|  | DMW intervention | - | 38 (38 / 38) | 38 (38 / 38) |
|  | Diabetes medicines | 1,871 | 1,795 (1,795 / 1,795) | −76 (−76 / −76) |
|  | Long-term complications | 16,444 | 16,433 (16,441 / 16,430) | −11 (−3 / −14) |
|  | Total cost | 18,315 | 18,266 (18,274 / 18,263) | −49 (−41 / −52) |
|  | Cost per QALY | - | - | Dominant (Dominant / Dominant) |
|  | Cost per QALY (exc. meds) | - | - | 11,580 (43,020 / 8,154) |

**Abbreviations**: BCS Best-Case Scenario; DMW Diabetes MyWay; LY Life-Year; QALY Quality-Adjusted Life-Year; WCS Worst-Case Scenario. Notes: WCS and BCS do not constitute confidence intervals. They represent the worst case and best case magnitude of the treatment effects for the examined clinical risk factors.

Table e4.16: Cost per QALY (patient level; discounted; 5-yr time horizon)

| Scenario | Outcome | Usual care | DMW (WCS / BCS) | Difference (WCS / BCS) |
| --- | --- | --- | --- | --- |
| 1-year effects & 2-year costs (base case) | LYs | 4·519 | 4·520 (4·519 / 4·521) | 0·002 (0·001 / 0·003) |
|  | QALYs | 3·664 | 3·666 (3·665 / 3·667) | 0·002 (0·001 / 0·003) |
|  | Costs |  |  |  |
|  | DMW intervention | - | 38 (38 / 38) | 38 (38 / 38) |
|  | Diabetes medicines | 1,871 | 1,797 (1,796 / 1,798) | −74 (−76 / −74) |
|  | Long-term complications | 8,551 | 8,523 (8,541 / 8,505) | −28 (−10 / −46) |
|  | Total cost | 10,422 | 10,357 (10,375 / 10,340) | −65 (−47 / −82) |
|  | Cost per QALY | - | - | Dominant (Dominant / Dominant) |
|  | Cost per QALY (exc. meds) | - | - | 4,879 (40,345 / Dominant) |
| Permanent costs & effects | LYs | 4·519 | 4·521 (4·519 / 4·523) | 0·003 (0·001 / 0·004) |
|  | QALYs | 3·664 | 3·667 (3·665 / 3·669) | 0·003 (0·001 / 0·005) |
|  | Costs |  |  |  |
|  | DMW intervention | - | 88 (88 / 88) | 88 (88 / 88) |
|  | Diabetes medicines | 1,871 | 1,708 (1,698 / 1,717) | −163 (−173 / −154) |
|  | Long-term complications | 8,551 | 8,505 (8,536 / 8,475) | −46 (−14 / −76) |
|  | Total cost | 10,422 | 10,301 (10,323 / 10,280) | −121 (−99 / −142) |
|  | Cost per QALY | - | - | Dominant (Dominant / Dominant) |
|  | Cost per QALY (exc. meds) | - | - | 14,838 (74,321 / 2,734) |
| 5-year costs & effects | LYs | 4·519 | 4·521 (4·519 / 4·523) | 0·003 (0·001 / 0·004) |
|  | QALYs | 3·664 | 3·667 (3·665 / 3·669) | 0·003 (0·001 / 0·005) |
|  | Costs |  |  |  |
|  | DMW intervention | - | 88 (88 / 88) | 88 (88 / 88) |
|  | Diabetes medicines | 1,871 | 1,698 (1,695 / 1,700) | −174 (−176 / −171) |
|  | Long-term complications | 8,551 | 8,505 (8,536 / 8,475) | −46 (−15 / −76) |
|  | Total cost | 10,422 | 10,290 (10,319 / 10,263) | −132 (−103 / −159) |
|  | Cost per QALY | - | - | Dominant (Dominant / Dominant) |
|  | Cost per QALY (exc. meds) | - | - | 14,886 (78,968 / 2,721) |
| 1-year effects  with immediate reversion; 2-year costs | LYs | 4·519 | 4·520 (4·519 / 4·520) | 0·001 (0·000 / 0·002) |
|  | QALYs | 3·664 | 3·665 (3·664 / 3·666) | 0·001 (0·000 / 0·002) |
|  | Costs |  |  |  |
|  | DMW intervention | - | 38 (38 / 38) | 38 (38 / 38) |
|  | Diabetes medicines | 1,871 | 1,795 (1,795 / 1,795) | −76 (−76 / −76) |
|  | Long-term complications | 8,551 | 8,540 (8,548 / 8,535) | −11 (−3 / −16) |
|  | Total cost | 10,422 | 10,373 (10,381 / 10,368) | −49 (−42 / −54) |
|  | Cost per QALY | - | - | Dominant (Dominant / Dominant) |
|  | Cost per QALY (exc. meds) | - | - | 25,039 (99,449 / 13,365) |

**Abbreviations**: BCS Best-Case Scenario; DMW Diabetes MyWay; LY Life-Year; QALY Quality-Adjusted Life-Year; WCS Worst-Case Scenario. Notes: WCS and BCS do not constitute confidence intervals. They represent the worst case and best case magnitude of the treatment effects for the examined clinical risk factors.

### Cost per QALY (Greater Manchester population level)

Table e4.17: Cost per QALY (population level; discounted; 40-yr time horizon; base case)

| Outcome | Usual care | DMW (WCS / BCS) | Difference (WCS / BCS) |
| --- | --- | --- | --- |
| LYs | 2,714,723 | 2,714,973 (2,714,832 / 2,715,103) | 250 (108 / 380) |
| QALYs | 2,167,019 | 2,167,262 (2,167,125 / 2,167,387) | 243 (106 / 368) |
| Costs |  |  |  |
| DMW intervention | - | 612,816 (612,814 / 612,813) | 612,816 (612,814 / 612,813) |
| Diabetes medicines | 340,627,490 | 339,421,267 (339,404,118 / 339,437,011) | −1,206,223 (−1,223,373 / −1,190,479) |
| Long-term complications | 6,996,003,048 | 6,995,119,540 (6,995,746,506 / 6,994,630,711) | −883,508 (−256,542 / −1,372,337) |
| Total cost | 7,336,630,538 | 7,335,153,623 (7,335,763,437 / 7,334,680,535) | −1,476,915 (−867,101 / −1,950,003) |
| Cost per QALY | - | - | Dominant (Dominant / Dominant) |
| Cost per QALY (exc. meds) | - | - | Dominant (3,372 / Dominant) |

**Abbreviations**: BCS Best-Case Scenario; DMW Diabetes MyWay; LY Life-Year; QALY Quality-Adjusted Life-Year; WCS Worst-Case Scenario. Notes: WCS and BCS do not constitute confidence intervals. They represent the worst case and best case magnitude of the treatment effects for the examined clinical risk factors.

Table e4.18: Cost per QALY (population level; discounted; 40-yr time horizon; scenarios)

| Scenario | Outcome | Usual care | DMW (WCS / BCS) | Difference (WCS / BCS) |
| --- | --- | --- | --- | --- |
| Permanent costs & effects | LYs | 2,714,723 | 2,716,652 (2,715,364 / 2,717,893) | 1,929 (641 / 3,170) |
|  | QALYs | 2,167,019 | 2,168,786 (2,167,625 / 2,169,907) | 1,767 (606 / 2,888) |
|  | Costs |  |  |  |
|  | DMW intervention | - | 4,786,192 (4,761,859 / 4,809,650) | 4,786,192 (4,761,859 / 4,809,650) |
|  | Diabetes medicines | 340,627,490 | 331,205,065 (331,097,255 / 331,309,103) | −9,422,426 (−9,530,236 / −9,318,388) |
|  | Long-term complications | 6,996,003,048 | 6,991,757,000 (6,994,689,891 / 6,988,963,641) | −4,246,048 (−1,313,157 / −7,039,407) |
|  | Total cost | 7,336,630,538 | 7,327,748,256 (7,330,549,005 / 7,325,082,394) | −8,882,282 (−6,081,533 / −11,548,144) |
|  | Cost per QALY | - | - | Dominant (Dominant / Dominant) |
|  | Cost per QALY (excluding medicines) | - | - | 306 (5,693 / Dominant) |
| 5-year costs & effects | LYs | 2,714,723 | 2,715,283 (2,714,941 / 2,715,640) | 560 (218 / 917) |
|  | QALYs | 2,167,019 | 2,167,555 (2,167,232 / 2,167,893) | 535 (213 / 874) |
|  | Costs |  |  |  |
|  | DMW intervention | - | 1,428,081 (1,427,679 / 1,428,430) | 1,428,081 (1,427,679 / 1,428,430) |
|  | Diabetes medicines | 340,627,490 | 337,813,773 (337,773,239 / 337,856,444) | −2,813,717 (−2,854,252 / −2,771,046) |
|  | Long-term complications | 6,996,003,048 | 6,994,321,733 (6,995,435,368 / 6,993,071,266) | −1,681,315 (−567,680 / −2,931,782) |
|  | Total cost | 7,336,630,538 | 7,333,563,587 (7,334,636,286 / 7,332,356,140) | −3,066,951 (−1,994,252 / −4,274,398) |
|  | Cost per QALY | - | - | Dominant (Dominant / Dominant) |
|  | Cost per QALY (excluding medicines) | - | - | Dominant (4,037 / Dominant) |
| 1-year effects  with immediate reversion; 2-year costs | LYs | 2,714,723 | 2,714,792 (2,714,755 / 2,714,764) | 69 (32 / 41) |
|  | QALYs | 2,167,019 | 2,167,086 (2,167,048 / 2,167,063) | 67 (29 / 44) |
|  | Costs |  |  |  |
|  | DMW intervention | - | 612,814 (612,815 / 612,816) | 612,814 (612,815 / 612,816) |
|  | Diabetes medicines | 340,627,490 | 339,399,300 (339,394,766 / 339,395,831) | −1,228,190 (−1,232,724 / −1,231,660) |
|  | Long-term complications | 6,996,003,048 | 6,995,855,447 (6,996,085,783 / 6,995,784,168) | −147,600 (82,735 / −218,880) |
|  | Total cost | 7,336,630,538 | 7,335,867,562 (7,336,093,364 / 7,335,792,814) | −762,976 (−537,174 / −837,724) |
|  | Cost per QALY | - | - | Dominant (Dominant / Dominant) |
|  | Cost per QALY (excluding medicines) | - | - | 6,977 (24,091 / 8,962) |

**Abbreviations**: BCS Best-Case Scenario; DMW Diabetes MyWay; LY Life-Year; QALY Quality-Adjusted Life-Year; WCS Worst-Case Scenario. Notes: WCS and BCS do not constitute confidence intervals. They represent the worst case and best case magnitude of the treatment effects for the examined clinical risk factors.

Table e4.19: Cost per QALY (population level; discounted; 10-yr time horizon)

| Scenario | Outcome | Usual care | DMW (WCS / BCS) | Difference (WCS / BCS) |
| --- | --- | --- | --- | --- |
| 1-year effects & 2-year costs (base case) | LYs | 1,451,836 | 1,451,929 (1,451,869 / 1,451,985) | 93 (33 / 150) |
|  | QALYs | 1,172,523 | 1,172,620 (1,172,560 / 1,172,678) | 97 (37 / 155) |
|  | Costs |  |  |  |
|  | DMW intervention | - | 612,816 (612,814 / 612,813) | 612,816 (612,814 / 612,813) |
|  | Diabetes medicines | 340,627,490 | 339,421,267 (339,404,118 / 339,437,011) | −1,206,223 (−1,223,373 / −1,190,479) |
|  | Long-term complications | 2,993,187,883 | 2,992,472,471 (2,992,929,559 / 2,992,038,059) | −715,412 (−258,324 / −1,149,825) |
|  | Total cost | 3,333,815,374 | 3,332,506,554 (3,332,946,491 / 3,332,087,883) | −1,308,820 (−868,883 / −1,727,491) |
|  | Cost per QALY | - | - | Dominant (Dominant / Dominant) |
|  | Cost per QALY (excluding medicines) | - | - | Dominant (9,663 / Dominant) |
| Permanent costs & effects | LYs | 1,451,836 | 1,452,055 (1,451,907 / 1,452,192) | 219 (71 / 357) |
|  | QALYs | 1,172,523 | 1,172,749 (1,172,600 / 1,172,888) | 226 (77 / 365) |
|  | Costs |  |  |  |
|  | DMW intervention | - | 2,527,940 (2,525,447 / 2,530,255) | 2,527,940 (2,525,447 / 2,530,255) |
|  | Diabetes medicines | 340,627,490 | 335,761,754 (335,609,876 / 335,908,454) | −4,865,736 (−5,017,614 / −4,719,036) |
|  | Long-term complications | 2,993,187,883 | 2,991,177,189 (2,992,541,314 / 2,989,874,033) | −2,010,694 (−646,569 / −3,313,850) |
|  | Total cost | 3,333,815,374 | 3,329,466,883 (3,330,676,637 / 3,328,312,742) | −4,348,491 (−3,138,737 / −5,502,631) |
|  | Cost per QALY | - | - | Dominant (Dominant / Dominant) |
|  | Cost per QALY (excluding medicines) | - | - | 2,285 (24,370 / Dominant) |
| 5-year costs & effects | LYs | 1,451,836 | 1,452,027 (1,451,896 / 1,452,144) | 192 (60 / 309) |
|  | QALYs | 1,172,523 | 1,172,720 (1,172,589 / 1,172,838) | 197 (66 / 316) |
|  | Costs |  |  |  |
|  | DMW intervention | - | 1,428,081 (1,427,679 / 1,428,430) | 1,428,081 (1,427,679 / 1,428,430) |
|  | Diabetes medicines | 340,627,490 | 337,813,773 (337,773,239 / 337,856,444) | −2,813,717 (−2,854,252 / −2,771,046) |
|  | Long-term complications | 2,993,187,883 | 2,991,675,448 (2,992,675,729 / 2,990,654,054) | −1,512,436 (−512,155 / −2,533,829) |
|  | Total cost | 3,333,815,374 | 3,330,917,301 (3,331,876,646 / 3,329,938,929) | −2,898,072 (−1,938,727 / −3,876,445) |
|  | Cost per QALY | - | - | Dominant (Dominant / Dominant) |
|  | Cost per QALY (excluding medicines) | - | - | Dominant (13,909 / Dominant) |
| 1-year effects  with immediate reversion; 2-year costs | LYs | 1,451,836 | 1,451,873 (1,451,848 / 1,451,884) | 37 (13 / 48) |
|  | QALYs | 1,172,523 | 1,172,561 (1,172,536 / 1,172,571) | 38 (13 / 48) |
|  | Costs |  |  |  |
|  | DMW intervention | - | 612,814 (612,815 / 612,816) | 612,814 (612,815 / 612,816) |
|  | Diabetes medicines | 340,627,490 | 339,399,300 (339,394,766 / 339,395,831) | −1,228,190 (−1,232,724 / −1,231,660) |
|  | Long-term complications | 2,993,187,883 | 2,993,012,958 (2,993,146,535 / 2,992,968,547) | −174,926 (−41,349 / −219,336) |
|  | Total cost | 3,333,815,374 | 3,333,025,072 (3,333,154,116 / 3,332,977,194) | −790,301 (−661,258 / −838,180) |
|  | Cost per QALY | - | - | Dominant (Dominant / Dominant) |
|  | Cost per QALY (excluding medicines) | - | - | 11,580 (43,020 / 8,154) |

**Abbreviations**: BCS Best-Case Scenario; DMW Diabetes MyWay; LY Life-Year; QALY Quality-Adjusted Life-Year; WCS Worst-Case Scenario. Notes: WCS and BCS do not constitute confidence intervals. They represent the worst case and best case magnitude of the treatment effects for the examined clinical risk factors.

Table e4.20: Cost per QALY (population level; discounted; 5-yr time horizon)

| Scenario | Outcome | Usual care | DMW (WCS / BCS) | Difference (WCS / BCS) |
| --- | --- | --- | --- | --- |
| 1-year effects & 2-year costs (base case) | LYs | 822,491 | 822,520 (822,501 / 822,538) | 29 (10 / 47) |
|  | QALYs | 666,954 | 666,986 (666,966 / 667,006) | 32 (11 / 52) |
|  | Costs |  |  |  |
|  | DMW intervention | - | 612,816 (612,814 / 612,813) | 612,816 (612,814 / 612,813) |
|  | Diabetes medicines | 340,627,490 | 339,421,267 (339,404,118 / 339,437,011) | −1,206,223 (−1,223,373 / −1,190,479) |
|  | Long-term complications | 1,556,453,182 | 1,555,996,410 (1,556,299,145 / 1,555,707,057) | −456,772 (−154,037 / −746,125) |
|  | Total cost | 1,897,080,672 | 1,896,030,493 (1,896,316,076 / 1,895,756,882) | −1,050,180 (−764,596 / −1,323,791) |
|  | Cost per QALY | - | - | Dominant (Dominant / Dominant) |
|  | Cost per QALY (excluding medicines) | - | - | 4,879 (40,345 / Dominant) |
| Permanent costs & effects | LYs | 822,491 | 822,532 (822,505 / 822,558) | 41 (14 / 66) |
|  | QALYs | 666,954 | 667,000 (666,970 / 667,027) | 46 (16 / 73) |
|  | Costs |  |  |  |
|  | DMW intervention | - | 1,428,078 (1,427,700 / 1,428,434) | 1,428,078 (1,427,700 / 1,428,434) |
|  | Diabetes medicines | 340,627,490 | 337,981,050 (337,824,903 / 338,131,704) | −2,646,441 (−2,802,587 / −2,495,786) |
|  | Long-term complications | 1,556,453,182 | 1,555,704,324 (1,556,219,074 / 1,555,225,050) | −748,859 (−234,108 / −1,228,132) |
|  | Total cost | 1,897,080,672 | 1,895,113,451 (1,895,471,678 / 1,894,785,188) | −1,967,221 (−1,608,995 / −2,295,485) |
|  | Cost per QALY | - | - | Dominant (Dominant / Dominant) |
|  | Cost per QALY (excluding medicines) | - | - | 14,838 (74,321 / 2,734) |
| 5-year costs & effects | LYs | 822,491 | 822,533 (822,504 / 822,558) | 41 (13 / 66) |
|  | QALYs | 666,954 | 667,000 (666,969 / 667,027) | 46 (15 / 73) |
|  | Costs |  |  |  |
|  | DMW intervention | - | 1,428,081 (1,427,679 / 1,428,430) | 1,428,081 (1,427,679 / 1,428,430) |
|  | Diabetes medicines | 340,627,490 | 337,813,773 (337,773,239 / 337,856,444) | −2,813,717 (−2,854,252 / −2,771,046) |
|  | Long-term complications | 1,556,453,182 | 1,555,707,424 (1,556,212,820 / 1,555,223,587) | −745,758 (−240,362 / −1,229,596) |
|  | Total cost | 1,897,080,672 | 1,894,949,278 (1,895,413,738 / 1,894,508,461) | −2,131,395 (−1,666,935 / −2,572,212) |
|  | Cost per QALY | - | - | Dominant (Dominant / Dominant) |
|  | Cost per QALY (excluding medicines) | - | - | 14,886 (78,968 / 2,721) |
| 1-year effects  with immediate reversion; 2-year costs | LYs | 822,491 | 822,507 (822,496 / 822,516) | 16 (5 / 25) |
|  | QALYs | 666,954 | 666,972 (666,960 / 666,980) | 17 (6 / 26) |
|  | Costs |  |  |  |
|  | DMW intervention | - | 612,814 (612,815 / 612,816) | 612,814 (612,815 / 612,816) |
|  | Diabetes medicines | 340,627,490 | 339,399,300 (339,394,766 / 339,395,831) | −1,228,190 (−1,232,724 / −1,231,660) |
|  | Long-term complications | 1,556,453,182 | 1,556,274,499 (1,556,400,879 / 1,556,190,335) | −178,683 (−52,303 / −262,847) |
|  | Total cost | 1,897,080,672 | 1,896,286,613 (1,896,408,460 / 1,896,198,981) | −794,059 (−672,213 / −881,691) |
|  | Cost per QALY | - | - | Dominant (Dominant / Dominant) |
|  | Cost per QALY (excluding medicines) | - | - | 25,039 (99,449 / 13,365) |

**Abbreviations**: BCS Best-Case Scenario; DMW Diabetes MyWay; LY Life-Year; QALY Quality-Adjusted Life-Year; WCS Worst-Case Scenario. Notes: WCS and BCS do not constitute confidence intervals. They represent the worst case and best case magnitude of the treatment effects for the examined clinical risk factors.

Figure e4.06: Cost-effectiveness of Diabetes My Way as a function of cost and uptake

| 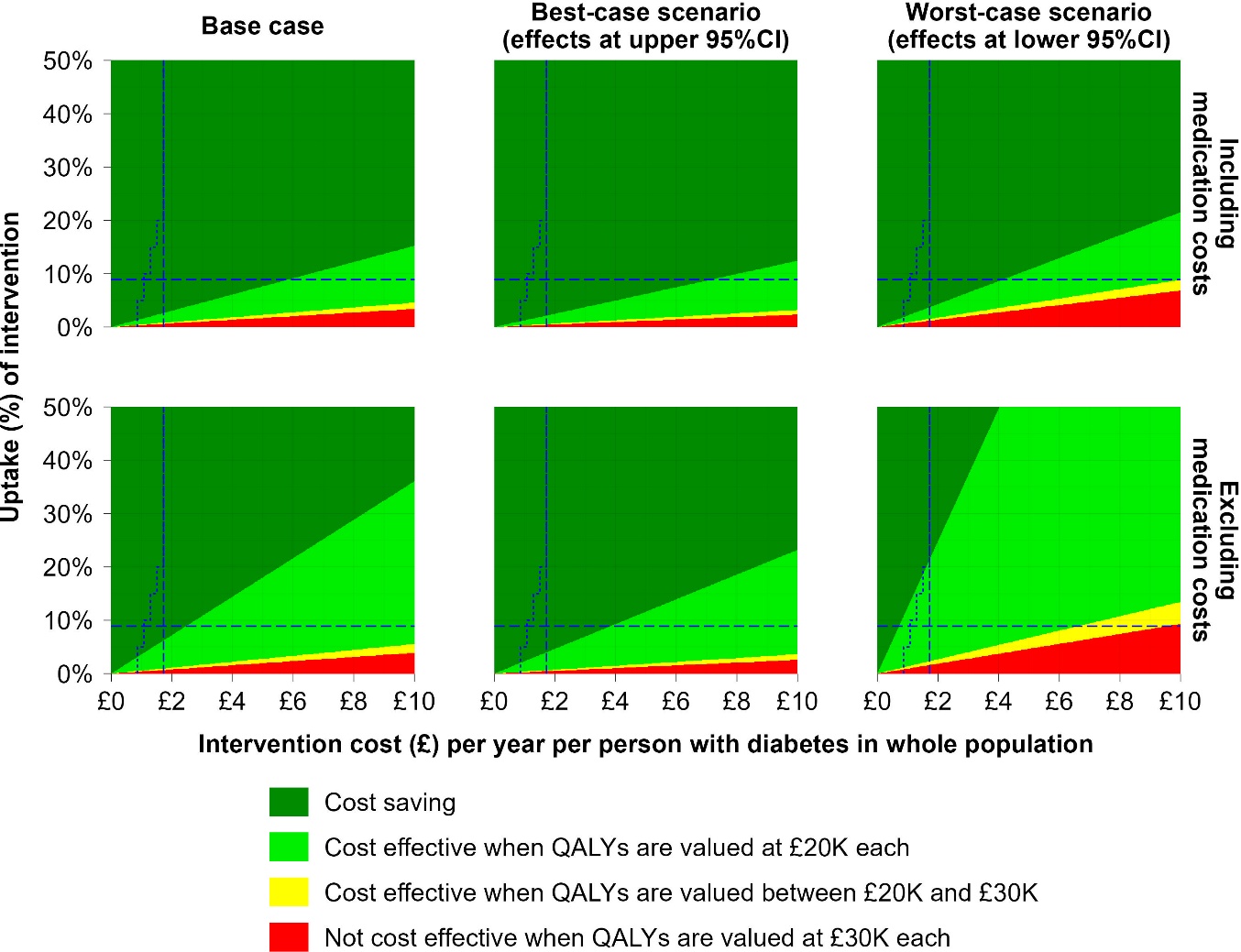 |
| --- |
| Horizontal dashed: current uptake in Greater Manchester (16,194 of 182,025 people with type 2 diabetes = 8·9%)  Vertical dashed: full GMDMW cost (licence fee = £313,557 per year; eligible population in Greater Manchester = 182,025; £1·72 per person per year)  Vertical dotted (staggered): full GMDMW cost given the current commercial arrangement (initial 50% discount; rising to full price once uptake reaches 20%; equal steps for every 5 percentage-points of uptake in between) |

## NICE initial population

### Expected events

Table e4.21: Expected events per 100,000 people with type 2 diabetes over 10 years (base-case)

| Event | Usual care | DMW (WCS / BCS) | Difference (WCS / BCS) | NNT (WCS / BCS) |
| --- | --- | --- | --- | --- |
| All death | 25,122 | 25,111 (25,104 / 25,118) | −11 (−18 / −4) | 795 (475 / 2,292) |
| Amputation | 1,364 | 1,361 (1,360 / 1,363) | −3 (−4 / −1) | 3,263 (2,127 / 7,434) |
| Blindness | 4,341 | 4,334 (4,330 / 4,337) | −7 (−11 / −4) | 1,241 (829 / 2,641) |
| Heart failure | 6,061 | 6,058 (6,056 / 6,060) | −3 (−5 / −1) | 3,557 (2,048 / 14,875) |
| IHD | 6,523 | 6,520 (6,516 / 6,523) | −3 (−7 / 0) | 2,389 (1,233 / 29,275) |
| MI | 12,096 | 12,080 (12,070 / 12,090) | −16 (−26 / −6) | 550 (335 / 1,564) |
| Renal failure | 474 | 473 (473 / 474) | −1 (−1 / 0) | 15,188 (9,122 / 69,803) |
| Stroke | 6,844 | 6,833 (6,827 / 6,840) | −11 (−17 / −4) | 859 (519 / 2,634) |
| Ulcer | 2,557 | 2,554 (2,552 / 2,555) | −3 (−5 / −2) | 2,605 (1,756 / 5,017) |

**Abbreviations**: BCS Best-Case Scenario; DMW Diabetes MyWay; IHD Ischaemic Heart Disease; MI myocardial infarction; WCS Worst-Case Scenario. Notes: WCS and BCS do not constitute confidence intervals. They represent the worst case and best case magnitude of the treatment effects for the examined clinical risk factors.

Table e4.22: Expected events per 100,000 people with type 2 diabetes over 10 years (scenarios)

| Scenario (see e4.1) | Event | Usual care | DMW (WCS / BCS) | Difference (WCS / BCS) | NNT (WCS / BCS) |
| --- | --- | --- | --- | --- | --- |
| Permanent costs & effects | All death | 25,122 | 25,077 (25,046 / 25,109) | −45 (−76 / −13) | 195 (117 / 662) |
|  | Amputation | 1,364 | 1,353 (1,347 / 1,359) | −11 (−17 / −5) | 803 (534 / 1,828) |
|  | Blindness | 4,341 | 4,322 (4,313 / 4,332) | −19 (−28 / −9) | 485 (320 / 1,031) |
|  | Heart failure | 6,061 | 6,047 (6,038 / 6,057) | −14 (−23 / −4) | 669 (390 / 2,769) |
|  | IHD | 6,523 | 6,506 (6,490 / 6,522) | −17 (−33 / −1) | 513 (270 / 6,064) |
|  | MI | 12,096 | 12,043 (12,009 / 12,080) | −53 (−87 / −16) | 168 (102 / 539) |
|  | Renal failure | 474 | 471 (469 / 473) | −3 (−5 / −1) | 3,501 (2,062 / 16,219) |
|  | Stroke | 6,844 | 6,806 (6,782 / 6,833) | −38 (−62 / −11) | 238 (144 / 789) |
|  | Ulcer | 2,557 | 2,547 (2,542 / 2,552) | −10 (−15 / −5) | 871 (585 / 1,807) |
| 5-year costs & effects | All death | 25,122 | 25,092 (25,073 / 25,113) | −30 (−49 / −9) | 297 (180 / 964) |
|  | Amputation | 1,364 | 1,357 (1,353 / 1,361) | −7 (−11 / −3) | 1,228 (825 / 2,776) |
|  | Blindness | 4,341 | 4,327 (4,319 / 4,334) | −14 (−22 / −7) | 625 (414 / 1,324) |
|  | Heart failure | 6,061 | 6,053 (6,047 / 6,059) | −8 (−14 / −2) | 1,125 (661 / 4,516) |
|  | IHD | 6,523 | 6,512 (6,502 / 6,522) | −11 (−21 / −1) | 792 (416 / 8,951) |
|  | MI | 12,096 | 12,058 (12,034 / 12,084) | −38 (−62 / −12) | 234 (142 / 720) |
|  | Renal failure | 474 | 472 (471 / 473) | −2 (−3 / −1) | 5,716 (3,335 / 23,882) |
|  | Stroke | 6,844 | 6,818 (6,802 / 6,836) | −26 (−42 / −8) | 351 (211 / 1,121) |
|  | Ulcer | 2,557 | 2,550 (2,546 / 2,554) | −7 (−11 / −3) | 1,155 (788 / 2,438) |
| 1-year effects  with immediate reversion; 2-year costs | All death | 25,122 | 25,119 (25,117 / 25,121) | −3 (−5 / −1) | 3,023 (1,711 / 8,195) |
|  | Amputation | 1,364 | 1,364 (1,363 / 1,364) | 0 (−1 / 0) | 19,246 (11,854 / 68,083) |
|  | Blindness | 4,341 | 4,339 (4,338 / 4,340) | −2 (−3 / −1) | 3,958 (2,823 / 8,449) |
|  | Heart failure | 6,061 | 6,060 (6,060 / 6,061) | −1 (−1 / 0) | 16,120 (8,284 / 89,206) |
|  | IHD | 6,523 | 6,522 (6,521 / 6,523) | −1 (−2 / 0) | 8,844 (3,775 / 266,880) |
|  | MI | 12,096 | 12,091 (12,088 / 12,094) | −5 (−8 / −2) | 1,879 (1,089 / 5,152) |
|  | Renal failure | 474 | 473 (473 / 474) | −1 (−1 / 0) | 30,045 (22,049 / 93,703) |
|  | Stroke | 6,844 | 6,841 (6,839 / 6,843) | −3 (−5 / −1) | 3,063 (2,005 / 11,338) |
|  | Ulcer | 2,557 | 2,556 (2,556 / 2,557) | −1 (−1 / 0) | 10,882 (6,603 / 21,214) |

**Abbreviations**: BCS Best-Case Scenario; DMW Diabetes MyWay; IHD Ischaemic Heart Disease; MI myocardial infarction; WCS Worst-Case Scenario. Notes: WCS and BCS do not constitute confidence intervals. They represent the worst case and best case magnitude of the treatment effects for the examined clinical risk factors.

### Cost per QALY (patient level)

Table e4.23: Cost per QALY (patient level; discounted; 40-yr time horizon; base case)

| Outcome | Usual care | DMW (WCS / BCS) | Difference (WCS / BCS) |
| --- | --- | --- | --- |
| LYs | 13·005 | 13·021 (13·011 / 13·031) | 0·015 (0·005 / 0·026) |
| QALYs | 10·361 | 10·376 (10·366 / 10·385) | 0·014 (0·005 / 0·024) |
| Costs |  |  |  |
| DMW intervention | - | 38 (38 / 38) | 38 (38 / 38) |
| Diabetes medicines | 1,611 | 1,538 (1,536 / 1,539) | −73 (−74 / −72) |
| Long-term complications | 34,565 | 34,531 (34,557 / 34,509) | −33 (−8 / −56) |
| Total cost | 36,175 | 36,107 (36,131 / 36,085) | −69 (−44 / −90) |
| Cost per QALY | - | - | Dominant (Dominant / Dominant) |
| Cost per QALY (excluding medicines) | - | - | 311 (5,926 / Dominant) |

**Abbreviations**: BCS Best-Case Scenario; DMW Diabetes MyWay; LY Life-Year; QALY Quality-Adjusted Life-Year; WCS Worst-Case Scenario. Notes: WCS and BCS do not constitute confidence intervals. They represent the worst case and best case magnitude of the treatment effects for the examined clinical risk factors.

Table e4.24: Cost per QALY (patient level; discounted; 40-yr time horizon; scenarios)

| Scenario | Outcome | Usual care | DMW (WCS / BCS) | Difference (WCS / BCS) |
| --- | --- | --- | --- | --- |
| Permanent costs & effects | LYs | 13·005 | 13·127 (13·042 / 13·210) | 0·122 (0·037 / 0·205) |
|  | QALYs | 10·361 | 10·469 (10·395 / 10·541) | 0·107 (0·033 / 0·180) |
|  | Costs |  |  |  |
|  | DMW intervention | - | 259 (258 / 261) | 259 (258 / 261) |
|  | Diabetes medicines | 1,611 | 1,111 (1,104 / 1,117) | −500 (−507 / −493) |
|  | Long-term complications | 34,565 | 34,428 (34,529 / 34,326) | −136 (−36 / −239) |
|  | Total cost | 36,175 | 35,798 (35,890 / 35,704) | −377 (−285 / −471) |
|  | Cost per QALY | - | - | Dominant (Dominant / Dominant) |
|  | Cost per QALY (exc. meds) | - | - | 1,147 (6,714 / 123) |
| 5-year costs & effects | LYs | 13·005 | 13·044 (13·018 / 13·067) | 0·038 (0·013 / 0·062) |
|  | QALYs | 10·361 | 10·397 (10·373 / 10·418) | 0·035 (0·012 / 0·057) |
|  | Costs |  |  |  |
|  | DMW intervention | - | 87 (87 / 87) | 87 (87 / 87) |
|  | Diabetes medicines | 1,611 | 1,442 (1,439 / 1,445) | −168 (−171 / −165) |
|  | Long-term complications | 34,565 | 34,492 (34,548 / 34,438) | −72 (−17 / −126) |
|  | Total cost | 36,175 | 36,022 (36,075 / 35,971) | −153 (−101 / −204) |
|  | Cost per QALY | - | - | Dominant (Dominant / Dominant) |
|  | Cost per QALY (exc. meds) | - | - | 420 (6,004 / Dominant) |
| 1-year effects  with immediate reversion; 2-year costs | LYs | 13·005 | 13·010 (13·007 / 13·013) | 0·005 (0·002 / 0·008) |
|  | QALYs | 10·361 | 10·366 (10·363 / 10·369) | 0·004 (0·002 / 0·007) |
|  | Costs |  |  |  |
|  | DMW intervention | - | 38 (38 / 38) | 38 (38 / 38) |
|  | Diabetes medicines | 1,611 | 1,536 (1,536 / 1,537) | −74 (−75 / −74) |
|  | Long-term complications | 34,565 | 34,547 (34,561 / 34,542) | −17 (−4 / −23) |
|  | Total cost | 36,175 | 36,122 (36,134 / 36,117) | −54 (−41 / −59) |
|  | Cost per QALY | - | - | Dominant (Dominant / Dominant) |
|  | Cost per QALY (exc. meds) | - | - | 4,614 (18,485 / 2,029) |

**Abbreviations**: BCS Best-Case Scenario; DMW Diabetes MyWay; LY Life-Year; QALY Quality-Adjusted Life-Year; WCS Worst-Case Scenario. Notes: WCS and BCS do not constitute confidence intervals. They represent the worst case and best case magnitude of the treatment effects for the examined clinical risk factors.

Table e4.25: Cost per QALY (patient level; discounted; 10-yr time horizon)

| Scenario | Outcome | Usual care | DMW (WCS / BCS) | Difference (WCS / BCS) |
| --- | --- | --- | --- | --- |
| 1-year effects & 2-year costs (base case) | LYs | 7·699 | 7·705 (7·701 / 7·710) | 0·007 (0·002 / 0·011) |
|  | QALYs | 6·201 | 6·208 (6·204 / 6·212) | 0·007 (0·002 / 0·011) |
|  | Costs |  |  |  |
|  | DMW intervention | - | 38 (38 / 38) | 38 (38 / 38) |
|  | Diabetes medicines | 1,611 | 1,538 (1,536 / 1,539) | −73 (−74 / −72) |
|  | Long-term complications | 16,907 | 16,866 (16,895 / 16,839) | −42 (−12 / −68) |
|  | Total cost | 18,518 | 18,441 (18,469 / 18,416) | −77 (−49 / −102) |
|  | Cost per QALY | - | - | Dominant (Dominant / Dominant) |
|  | Cost per QALY (exc. meds) | - | - | Dominant (11,681 / Dominant) |
| Permanent costs & effects | LYs | 7·699 | 7·715 (7·704 / 7·726) | 0·016 (0·005 / 0·027) |
|  | QALYs | 6·201 | 6·217 (6·206 / 6·228) | 0·016 (0·005 / 0·026) |
|  | Costs |  |  |  |
|  | DMW intervention | - | 151 (151 / 152) | 151 (151 / 152) |
|  | Diabetes medicines | 1,611 | 1,325 (1,315 / 1,335) | −286 (−295 / −276) |
|  | Long-term complications | 16,907 | 16,785 (16,873 / 16,703) | −122 (−35 / −204) |
|  | Total cost | 18,518 | 18,262 (18,339 / 18,189) | −256 (−179 / −329) |
|  | Cost per QALY | - | - | Dominant (Dominant / Dominant) |
|  | Cost per QALY (exc. meds) | - | - | 1,845 (24,039 / Dominant) |
| 5-year costs & effects | LYs | 7·699 | 7·713 (7·703 / 7·722) | 0·014 (0·004 / 0·023) |
|  | QALYs | 6·201 | 6·215 (6·206 / 6·224) | 0·014 (0·004 / 0·023) |
|  | Costs |  |  |  |
|  | DMW intervention | - | 87 (87 / 87) | 87 (87 / 87) |
|  | Diabetes medicines | 1,611 | 1,442 (1,439 / 1,445) | −168 (−171 / −165) |
|  | Long-term complications | 16,907 | 16,816 (16,880 / 16,755) | −91 (−27 / −153) |
|  | Total cost | 18,518 | 18,346 (18,407 / 18,287) | −172 (−111 / −231) |
|  | Cost per QALY | - | - | Dominant (Dominant / Dominant) |
|  | Cost per QALY (exc. meds) | - | - | Dominant (14,170 / Dominant) |
| 1-year effects  with immediate reversion; 2-year costs | LYs | 7·699 | 7·701 (7·700 / 7·703) | 0·002 (0·001 / 0·004) |
|  | QALYs | 6·201 | 6·204 (6·202 / 6·205) | 0·002 (0·001 / 0·004) |
|  | Costs |  |  |  |
|  | DMW intervention | - | 38 (38 / 38) | 38 (38 / 38) |
|  | Diabetes medicines | 1,611 | 1,536 (1,536 / 1,537) | −74 (−75 / −74) |
|  | Long-term complications | 16,907 | 16,892 (16,903 / 16,883) | −15 (−4 / −24) |
|  | Total cost | 18,518 | 18,467 (18,477 / 18,458) | −51 (−41 / −60) |
|  | Cost per QALY | - | - | Dominant (Dominant / Dominant) |
|  | Cost per QALY (exc. meds) | - | - | 9,611 (46,245 / 3,416) |

**Abbreviations**: BCS Best-Case Scenario; DMW Diabetes MyWay; LY Life-Year; QALY Quality-Adjusted Life-Year; WCS Worst-Case Scenario. Notes: WCS and BCS do not constitute confidence intervals. They represent the worst case and best case magnitude of the treatment effects for the examined clinical risk factors.

Table e4.26: Cost per QALY (patient level; discounted; 5-yr time horizon)

| Scenario | Outcome | Usual care | DMW (WCS / BCS) | Difference (WCS / BCS) |
| --- | --- | --- | --- | --- |
| 1-year effects & 2-year costs (base case) | LYs | 4·448 | 4·450 (4·449 / 4·452) | 0·002 (0·001 / 0·003) |
|  | QALYs | 3·600 | 3·603 (3·601 / 3·604) | 0·002 (0·001 / 0·004) |
|  | Costs |  |  |  |
|  | DMW intervention | - | 38 (38 / 38) | 38 (38 / 38) |
|  | Diabetes medicines | 1,611 | 1,538 (1,536 / 1,539) | −73 (−74 / −72) |
|  | Long-term complications | 8,971 | 8,940 (8,962 / 8,920) | −31 (−9 / −51) |
|  | Total cost | 10,582 | 10,516 (10,536 / 10,497) | −66 (−46 / −85) |
|  | Cost per QALY | - | - | Dominant (Dominant / Dominant) |
|  | Cost per QALY (exc. meds) | - | - | 3,302 (43,160 / Dominant) |
| Permanent costs & effects | LYs | 4·448 | 4·451 (4·449 / 4·453) | 0·003 (0·001 / 0·005) |
|  | QALYs | 3·600 | 3·603 (3·601 / 3·605) | 0·003 (0·001 / 0·005) |
|  | Costs |  |  |  |
|  | DMW intervention | - | 87 (87 / 87) | 87 (87 / 87) |
|  | Diabetes medicines | 1,611 | 1,452 (1,442 / 1,462) | −158 (−168 / −148) |
|  | Long-term complications | 8,971 | 8,921 (8,957 / 8,887) | −50 (−14 / −84) |
|  | Total cost | 10,582 | 10,460 (10,486 / 10,436) | −122 (−96 / −145) |
|  | Cost per QALY | - | - | Dominant (Dominant / Dominant) |
|  | Cost per QALY (exc. meds) | - | - | 12,097 (78,218 / 588) |
| 5-year costs & effects | LYs | 4·448 | 4·451 (4·449 / 4·453) | 0·003 (0·001 / 0·005) |
|  | QALYs | 3·600 | 3·603 (3·601 / 3·605) | 0·003 (0·001 / 0·005) |
|  | Costs |  |  |  |
|  | DMW intervention | - | 87 (87 / 87) | 87 (87 / 87) |
|  | Diabetes medicines | 1,611 | 1,442 (1,439 / 1,445) | −168 (−171 / −165) |
|  | Long-term complications | 8,971 | 8,921 (8,956 / 8,887) | −50 (−15 / −84) |
|  | Total cost | 10,582 | 10,451 (10,483 / 10,420) | −131 (−99 / −162) |
|  | Cost per QALY | - | - | Dominant (Dominant / Dominant) |
|  | Cost per QALY (exc. meds) | - | - | 11,926 (78,370 / 618) |
| 1-year effects  with immediate reversion; 2-year costs | LYs | 4·448 | 4·449 (4·449 / 4·450) | 0·001 (0·000 / 0·002) |
|  | QALYs | 3·600 | 3·601 (3·601 / 3·602) | 0·001 (0·000 / 0·002) |
|  | Costs |  |  |  |
|  | DMW intervention | - | 38 (38 / 38) | 38 (38 / 38) |
|  | Diabetes medicines | 1,611 | 1,536 (1,536 / 1,537) | −74 (−75 / −74) |
|  | Long-term complications | 8,971 | 8,959 (8,968 / 8,950) | −12 (−4 / −21) |
|  | Total cost | 10,582 | 10,533 (10,541 / 10,524) | −49 (−40 / −57) |
|  | Cost per QALY | - | - | Dominant (Dominant / Dominant) |
|  | Cost per QALY (exc. meds) | - | - | 22,675 (105,121 / 8,796) |

**Abbreviations**: BCS Best-Case Scenario; DMW Diabetes MyWay; LY Life-Year; QALY Quality-Adjusted Life-Year; WCS Worst-Case Scenario. Notes: WCS and BCS do not constitute confidence intervals. They represent the worst case and best case magnitude of the treatment effects for the examined clinical risk factors.

### Cost per QALY (Greater Manchester population level)

Table e4.27: Cost per QALY (population level; discounted; 40-yr time horizon; base case)

| Outcome | Usual care | DMW (WCS / BCS) | Difference (WCS / BCS) |
| --- | --- | --- | --- |
| LYs | 2,367,271 | 2,367,522 (2,367,358 / 2,367,688) | 251 (87 / 417) |
| QALYs | 1,886,035 | 1,886,267 (1,886,116 / 1,886,420) | 232 (82 / 385) |
| Costs |  |  |  |
| DMW intervention | - | 611,028 (611,028 / 611,027) | 611,028 (611,028 / 611,027) |
| Diabetes medicines | 293,184,366 | 292,002,522 (291,983,005 / 292,022,333) | −1,181,845 (−1,201,361 / −1,162,034) |
| Long-term complications | 6,291,610,216 | 6,291,071,462 (6,291,483,725 / 6,290,703,622) | −538,754 (−126,491 / −906,594) |
| Total cost | 6,584,794,582 | 6,583,685,012 (6,584,077,758 / 6,583,336,982) | −1,109,571 (−716,824 / −1,457,601) |
| Cost per QALY | - | - | Dominant (Dominant / Dominant) |
| Cost per QALY (exc. meds) | - | - | 311 (5,926 / Dominant) |

**Abbreviations**: BCS Best-Case Scenario; DMW Diabetes MyWay; LY Life-Year; QALY Quality-Adjusted Life-Year; WCS Worst-Case Scenario. Notes: WCS and BCS do not constitute confidence intervals. They represent the worst case and best case magnitude of the treatment effects for the examined clinical risk factors.

Table e4.28: Cost per QALY (population level; discounted; 40-yr time horizon; scenarios)

| Scenario | Outcome | Usual care | DMW (WCS / BCS) | Difference (WCS / BCS) |
| --- | --- | --- | --- | --- |
| Permanent costs & effects | LYs | 2,367,271 | 2,369,241 (2,367,863 / 2,370,584) | 1,969 (592 / 3,313) |
|  | QALYs | 1,886,035 | 1,887,775 (1,886,571 / 1,888,950) | 1,740 (536 / 2,916) |
|  | Costs |  |  |  |
|  | DMW intervention | - | 4,201,923 (4,175,822 / 4,227,382) | 4,201,923 (4,175,822 / 4,227,382) |
|  | Diabetes medicines | 293,184,366 | 285,087,311 (284,974,443 / 285,197,496) | −8,097,055 (−8,209,923 / −7,986,870) |
|  | Long-term complications | 6,291,610,216 | 6,289,403,940 (6,291,032,788 / 6,287,741,480) | −2,206,276 (−577,428 / −3,868,736) |
|  | Total cost | 6,584,794,582 | 6,578,693,174 (6,580,183,053 / 6,577,166,358) | −6,101,408 (−4,611,529 / −7,628,224) |
|  | Cost per QALY | - | - | Dominant (Dominant / Dominant) |
|  | Cost per QALY (excluding medicines) | - | - | 1,147 (6,714 / 123) |
| 5-year costs & effects | LYs | 2,367,271 | 2,367,893 (2,367,475 / 2,368,279) | 622 (204 / 1,008) |
|  | QALYs | 1,886,035 | 1,886,604 (1,886,225 / 1,886,955) | 569 (190 / 920) |
|  | Costs |  |  |  |
|  | DMW intervention | - | 1,410,889 (1,410,429 / 1,411,302) | 1,410,889 (1,410,429 / 1,411,302) |
|  | Diabetes medicines | 293,184,366 | 290,460,629 (290,411,805 / 290,505,736) | −2,723,737 (−2,772,561 / −2,678,630) |
|  | Long-term complications | 6,291,610,216 | 6,290,438,758 (6,291,341,388 / 6,289,566,721) | −1,171,457 (−268,828 / −2,043,495) |
|  | Total cost | 6,584,794,582 | 6,582,310,276 (6,583,163,622 / 6,581,483,759) | −2,484,306 (−1,630,960 / −3,310,823) |
|  | Cost per QALY | - | - | Dominant (Dominant / Dominant) |
|  | Cost per QALY (excluding medicines) | - | - | 420 (6,004 / Dominant) |
| 1-year effects  with immediate reversion; 2-year costs | LYs | 2,367,271 | 2,367,348 (2,367,303 / 2,367,403) | 77 (32 / 132) |
|  | QALYs | 1,886,035 | 1,886,107 (1,886,064 / 1,886,156) | 72 (30 / 121) |
|  | Costs |  |  |  |
|  | DMW intervention | - | 611,028 (611,028 / 611,028) | 611,028 (611,028 / 611,028) |
|  | Diabetes medicines | 293,184,366 | 291,981,873 (291,976,524 / 291,988,399) | −1,202,493 (−1,207,842 / −1,195,967) |
|  | Long-term complications | 6,291,610,216 | 6,291,332,351 (6,291,547,576 / 6,291,245,415) | −277,865 (−62,640 / −364,801) |
|  | Total cost | 6,584,794,582 | 6,583,925,252 (6,584,135,128 / 6,583,844,841) | −869,330 (−659,454 / −949,741) |
|  | Cost per QALY | - | - | Dominant (Dominant / Dominant) |
|  | Cost per QALY (excluding medicines) | - | - | 4,614 (18,485 / 2,029) |

**Abbreviations**: BCS Best-Case Scenario; DMW Diabetes MyWay; LY Life-Year; QALY Quality-Adjusted Life-Year; WCS Worst-Case Scenario. Notes: WCS and BCS do not constitute confidence intervals. They represent the worst case and best case magnitude of the treatment effects for the examined clinical risk factors.

Table e4.29: Cost per QALY (population level; discounted; 10-yr time horizon)

| Scenario | Outcome | Usual care | DMW (WCS / BCS) | Difference (WCS / BCS) |
| --- | --- | --- | --- | --- |
| 1-year effects & 2-year costs (base case) | LYs | 1,401,394 | 1,401,500 (1,401,427 / 1,401,573) | 106 (34 / 179) |
|  | QALYs | 1,128,810 | 1,128,917 (1,128,845 / 1,128,988) | 106 (35 / 178) |
|  | Costs |  |  |  |
|  | DMW intervention | - | 611,028 (611,028 / 611,027) | 611,028 (611,028 / 611,027) |
|  | Diabetes medicines | 293,184,366 | 292,002,522 (291,983,005 / 292,022,333) | −1,181,845 (−1,201,361 / −1,162,034) |
|  | Long-term complications | 3,077,549,248 | 3,076,876,499 (3,077,347,053 / 3,076,444,725) | −672,749 (−202,195 / −1,104,523) |
|  | Total cost | 3,370,733,614 | 3,369,490,049 (3,369,941,086 / 3,369,078,084) | −1,243,566 (−792,528 / −1,655,530) |
|  | Cost per QALY | - | - | Dominant (Dominant / Dominant) |
|  | Cost per QALY (excluding medicines) | - | - | Dominant (11,681 / Dominant) |
| Permanent costs & effects | LYs | 1,401,394 | 1,401,652 (1,401,470 / 1,401,827) | 258 (76 / 433) |
|  | QALYs | 1,128,810 | 1,129,067 (1,128,889 / 1,129,238) | 257 (79 / 428) |
|  | Costs |  |  |  |
|  | DMW intervention | - | 2,451,685 (2,448,621 / 2,454,629) | 2,451,685 (2,448,621 / 2,454,629) |
|  | Diabetes medicines | 293,184,366 | 288,558,042 (288,399,492 / 288,712,875) | −4,626,325 (−4,784,874 / −4,471,491) |
|  | Long-term complications | 3,077,549,248 | 3,075,571,942 (3,076,987,990 / 3,074,243,915) | −1,977,306 (−561,258 / −3,305,333) |
|  | Total cost | 3,370,733,614 | 3,366,581,669 (3,367,836,104 / 3,365,411,419) | −4,151,945 (−2,897,511 / −5,322,195) |
|  | Cost per QALY | - | - | Dominant (Dominant / Dominant) |
|  | Cost per QALY (excluding medicines) | - | - | 1,845 (24,039 / Dominant) |
| 5-year costs & effects | LYs | 1,401,394 | 1,401,620 (1,401,461 / 1,401,767) | 226 (67 / 373) |
|  | QALYs | 1,128,810 | 1,129,034 (1,128,879 / 1,129,178) | 223 (69 / 367) |
|  | Costs |  |  |  |
|  | DMW intervention | - | 1,410,889 (1,410,429 / 1,411,302) | 1,410,889 (1,410,429 / 1,411,302) |
|  | Diabetes medicines | 293,184,366 | 290,460,629 (290,411,805 / 290,505,736) | −2,723,737 (−2,772,561 / −2,678,630) |
|  | Long-term complications | 3,077,549,248 | 3,076,073,561 (3,077,113,873 / 3,075,075,134) | −1,475,687 (−435,375 / −2,474,114) |
|  | Total cost | 3,370,733,614 | 3,367,945,079 (3,368,936,106 / 3,366,992,173) | −2,788,536 (−1,797,508 / −3,741,441) |
|  | Cost per QALY | - | - | Dominant (Dominant / Dominant) |
|  | Cost per QALY (excluding medicines) | - | - | Dominant (14,170 / Dominant) |
| 1-year effects  with immediate reversion; 2-year costs | LYs | 1,401,394 | 1,401,432 (1,401,405 / 1,401,460) | 38 (12 / 67) |
|  | QALYs | 1,128,810 | 1,128,849 (1,128,822 / 1,128,876) | 38 (12 / 66) |
|  | Costs |  |  |  |
|  | DMW intervention | - | 611,028 (611,028 / 611,028) | 611,028 (611,028 / 611,028) |
|  | Diabetes medicines | 293,184,366 | 291,981,873 (291,976,524 / 291,988,399) | −1,202,493 (−1,207,842 / −1,195,967) |
|  | Long-term complications | 3,077,549,248 | 3,077,307,392 (3,077,487,715 / 3,077,162,350) | −241,856 (−61,533 / −386,898) |
|  | Total cost | 3,370,733,614 | 3,369,900,293 (3,370,075,267 / 3,369,761,777) | −833,321 (−658,347 / −971,837) |
|  | Cost per QALY | - | - | Dominant (Dominant / Dominant) |
|  | Cost per QALY (excluding medicines) | - | - | 9,611 (46,245 / 3,416) |

**Abbreviations**: BCS Best-Case Scenario; DMW Diabetes MyWay; LY Life-Year; QALY Quality-Adjusted Life-Year; WCS Worst-Case Scenario. Notes: WCS and BCS do not constitute confidence intervals. They represent the worst case and best case magnitude of the treatment effects for the examined clinical risk factors.

Table e4.30: Cost per QALY (population level; discounted; 5-yr time horizon)

| Scenario | Outcome | Usual care | DMW (WCS / BCS) | Difference (WCS / BCS) |
| --- | --- | --- | --- | --- |
| 1-year effects & 2-year costs (base case) | LYs | 809,729 | 809,760 (809,738 / 809,782) | 32 (9 / 53) |
|  | QALYs | 655,359 | 655,394 (655,370 / 655,417) | 35 (11 / 58) |
|  | Costs |  |  |  |
|  | DMW intervention | - | 611,028 (611,028 / 611,027) | 611,028 (611,028 / 611,027) |
|  | Diabetes medicines | 293,184,366 | 292,002,522 (291,983,005 / 292,022,333) | −1,181,845 (−1,201,361 / −1,162,034) |
|  | Long-term complications | 1,632,965,017 | 1,632,468,400 (1,632,812,862 / 1,632,139,980) | −496,617 (−152,155 / −825,037) |
|  | Total cost | 1,926,149,383 | 1,925,081,950 (1,925,406,895 / 1,924,773,339) | −1,067,433 (−742,488 / −1,376,044) |
|  | Cost per QALY | - | - | Dominant (Dominant / Dominant) |
|  | Cost per QALY (excluding medicines) | - | - | 3,302 (43,160 / Dominant) |
| Permanent costs & effects | LYs | 809,729 | 809,773 (809,741 / 809,804) | 44 (13 / 75) |
|  | QALYs | 655,359 | 655,408 (655,374 / 655,441) | 49 (15 / 82) |
|  | Costs |  |  |  |
|  | DMW intervention | - | 1,410,866 (1,410,429 / 1,411,289) | 1,410,866 (1,410,429 / 1,411,289) |
|  | Diabetes medicines | 293,184,366 | 290,621,993 (290,458,232 / 290,781,824) | −2,562,373 (−2,726,134 / −2,402,542) |
|  | Long-term complications | 1,632,965,017 | 1,632,148,574 (1,632,730,317 / 1,631,601,984) | −816,442 (−234,700 / −1,363,032) |
|  | Total cost | 1,926,149,383 | 1,924,181,433 (1,924,598,978 / 1,923,795,098) | −1,967,950 (−1,550,405 / −2,354,285) |
|  | Cost per QALY | - | - | Dominant (Dominant / Dominant) |
|  | Cost per QALY (excluding medicines) | - | - | 12,097 (78,218 / 588) |
| 5-year costs & effects | LYs | 809,729 | 809,774 (809,741 / 809,805) | 46 (13 / 76) |
|  | QALYs | 655,359 | 655,409 (655,374 / 655,442) | 50 (15 / 83) |
|  | Costs |  |  |  |
|  | DMW intervention | - | 1,410,889 (1,410,429 / 1,411,302) | 1,410,889 (1,410,429 / 1,411,302) |
|  | Diabetes medicines | 293,184,366 | 290,460,629 (290,411,805 / 290,505,736) | −2,723,737 (−2,772,561 / −2,678,630) |
|  | Long-term complications | 1,632,965,017 | 1,632,153,911 (1,632,727,933 / 1,631,604,741) | −811,106 (−237,084 / −1,360,276) |
|  | Total cost | 1,926,149,383 | 1,924,025,428 (1,924,550,167 / 1,923,521,780) | −2,123,955 (−1,599,216 / −2,627,603) |
|  | Cost per QALY | - | - | Dominant (Dominant / Dominant) |
|  | Cost per QALY (excluding medicines) | - | - | 11,926 (78,370 / 618) |
| 1-year effects  with immediate reversion; 2-year costs | LYs | 809,729 | 809,745 (809,733 / 809,757) | 17 (5 / 29) |
|  | QALYs | 655,359 | 655,377 (655,364 / 655,390) | 18 (5 / 30) |
|  | Costs |  |  |  |
|  | DMW intervention | - | 611,028 (611,028 / 611,028) | 611,028 (611,028 / 611,028) |
|  | Diabetes medicines | 293,184,366 | 291,981,873 (291,976,524 / 291,988,399) | −1,202,493 (−1,207,842 / −1,195,967) |
|  | Long-term complications | 1,632,965,017 | 1,632,762,922 (1,632,907,651 / 1,632,620,719) | −202,095 (−57,366 / −344,298) |
|  | Total cost | 1,926,149,383 | 1,925,355,823 (1,925,495,203 / 1,925,220,146) | −793,560 (−654,180 / −929,237) |
|  | Cost per QALY | - | - | Dominant (Dominant / Dominant) |
|  | Cost per QALY (excluding medicines) | - | - | 22,675 (105,121 / 8,796) |

**Abbreviations**: BCS Best-Case Scenario; DMW Diabetes MyWay; LY Life-Year; QALY Quality-Adjusted Life-Year; WCS Worst-Case Scenario. Notes: WCS and BCS do not constitute confidence intervals. They represent the worst case and best case magnitude of the treatment effects for the examined clinical risk factors.

Figure e4.07: Cost-effectiveness of Diabetes My Way as a function of cost and uptake

| 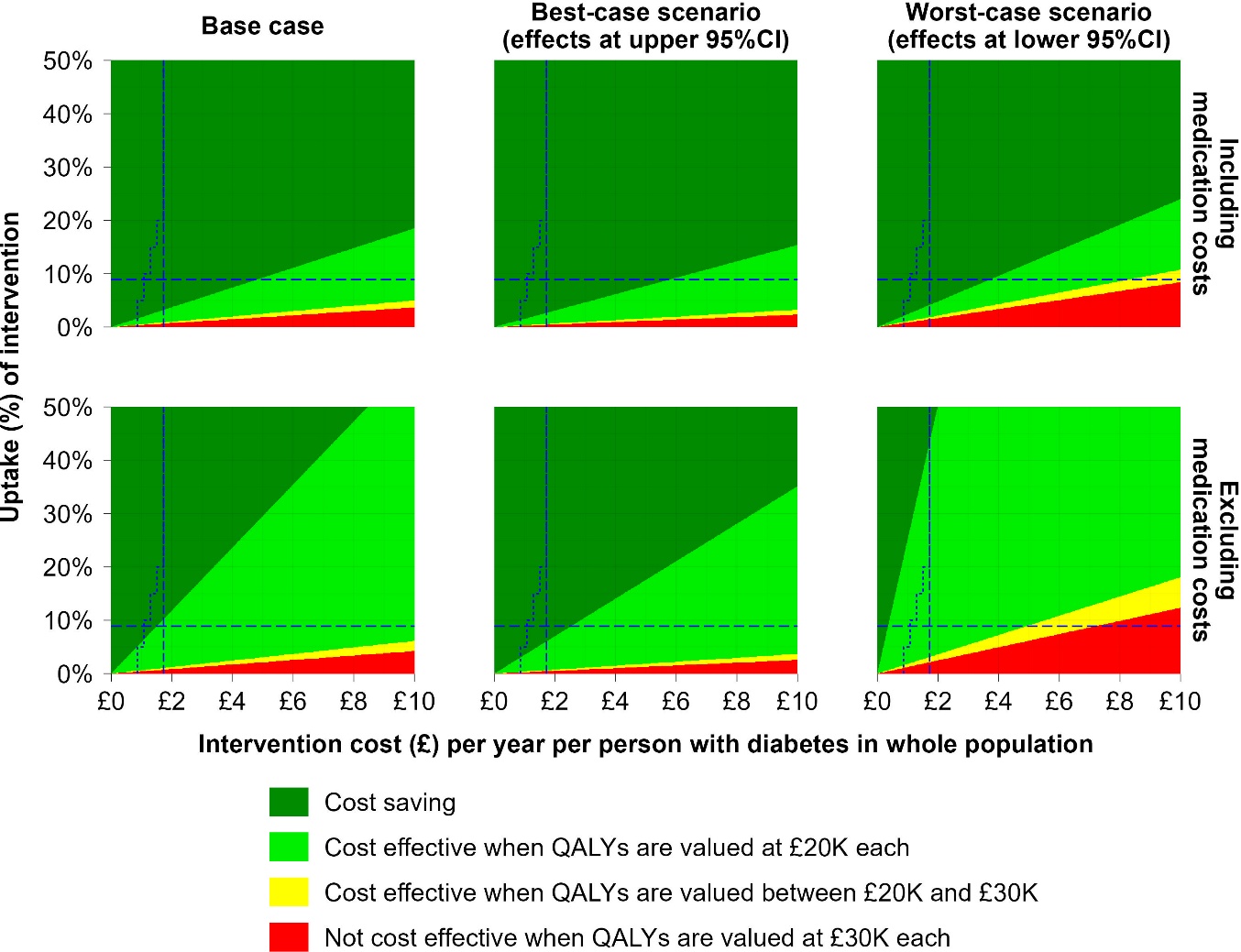 |
| --- |
| Horizontal dashed: current uptake in Greater Manchester (16,194 of 182,025 people with type 2 diabetes = 8·9%)  Vertical dashed: full GMDMW cost (licence fee = £313,557 per year; eligible population in Greater Manchester = 182,025; £1·72 per person per year)  Vertical dotted (staggered): full GMDMW cost given the current commercial arrangement (initial 50% discount; rising to full price once uptake reaches 20%; equal steps for every 5 percentage-points of uptake in between) |

## UKPDS-OM default inputs

### Cost per QALY (patient level)

Table e4.31: Cost per QALY (patient level; discounted; 40-yr time horizon; base case)

| Outcome | Usual care | DMW (WCS / BCS) | Difference (WCS / BCS) |
| --- | --- | --- | --- |
| LYs | 12·392 | 12·409 (12·399 / 12·421) | 0·017 (0·006 / 0·028) |
| QALYs | 9·776 | 9·791 (9·782 / 9·802) | 0·015 (0·006 / 0·026) |
| Costs |  |  |  |
| DMW intervention | - | 38 (38 / 38) | 38 (38 / 38) |
| Diabetes medicines | 1,548 | 1,475 (1,474 / 1,476) | −73 (−74 / −72) |
| Long-term complications | 29,226 | 29,222 (29,228 / 29,220) | −3 (2 / −6) |
| Total cost | 30,774 | 30,735 (30,739 / 30,734) | −39 (−35 / −40) |
| Cost per QALY | - | - | Dominant (Dominant / Dominant) |
| Cost per QALY (excluding medicines) | - | - | 2,237 (6,566 / 1,217) |

**Abbreviations**: BCS Best-Case Scenario; DMW Diabetes MyWay; LY Life-Year; QALY Quality-Adjusted Life-Year; WCS Worst-Case Scenario. **Notes**: WCS and BCS do not constitute confidence intervals. They represent the worst case and best case magnitude of the treatment effects for the examined clinical risk factors.

Table e4.32: Cost per QALY (patient level; discounted; 40-yr time horizon; scenarios)

| Scenario | Outcome | Usual care | DMW (WCS / BCS) | Difference (WCS / BCS) |
| --- | --- | --- | --- | --- |
| Permanent costs & effects | LYs | 12·392 | 12·513 (12·430 / 12·596) | 0·121 (0·038 / 0·203) |
|  | QALYs | 9·776 | 9·881 (9·809 / 9·953) | 0·106 (0·034 / 0·177) |
|  | Costs |  |  |  |
|  | DMW intervention | - | 248 (246 / 249) | 248 (246 / 249) |
|  | Diabetes medicines | 1,548 | 1,068 (1,061 / 1,075) | −480 (−487 / −473) |
|  | Long-term complications | 29,226 | 29,231 (29,235 / 29,227) | 6 (9 / 1) |
|  | Total cost | 30,774 | 30,547 (30,542 / 30,551) | −227 (−232 / −223) |
|  | Cost per QALY | - | - | Dominant (Dominant / Dominant) |
|  | Cost per QALY (exc. meds) | - | - | 2,401 (7,559 / 1,412) |
| 5-year costs & effects | LYs | 12·392 | 12·434 (12·406 / 12·459) | 0·041 (0·014 / 0·067) |
|  | QALYs | 9·776 | 9·813 (9·788 / 9·836) | 0·037 (0·013 / 0·060) |
|  | Costs |  |  |  |
|  | DMW intervention | - | 86 (86 / 86) | 86 (86 / 86) |
|  | Diabetes medicines | 1,548 | 1,381 (1,377 / 1,384) | −167 (−171 / −164) |
|  | Long-term complications | 29,226 | 29,215 (29,227 / 29,204) | −11 (1 / −22) |
|  | Total cost | 30,774 | 30,682 (30,691 / 30,674) | −92 (−83 / −100) |
|  | Cost per QALY | - | - | Dominant (Dominant / Dominant) |
|  | Cost per QALY (exc. meds) | - | - | 2,024 (6,940 / 1,066) |
| 1-year effects  with immediate reversion; 2-year costs | LYs | 12·392 | 12·398 (12·394 / 12·401) | 0·005 (0·002 / 0·008) |
|  | QALYs | 9·776 | 9·780 (9·777 / 9·783) | 0·005 (0·002 / 0·008) |
|  | Costs |  |  |  |
|  | DMW intervention | - | 38 (38 / 38) | 38 (38 / 38) |
|  | Diabetes medicines | 1,548 | 1,473 (1,473 / 1,474) | −75 (−75 / −74) |
|  | Long-term complications | 29,226 | 29,228 (29,230 / 29,225) | 2 (4 / −1) |
|  | Total cost | 30,774 | 30,739 (30,741 / 30,736) | −35 (−33 / −38) |
|  | Cost per QALY | - | - | Dominant (Dominant / Dominant) |
|  | Cost per QALY (exc. meds) | - | - | 8,842 (23,845 / 4,872) |

**Abbreviations**: BCS Best-Case Scenario; DMW Diabetes MyWay; LY Life-Year; QALY Quality-Adjusted Life-Year; WCS Worst-Case Scenario. Notes: WCS and BCS do not constitute confidence intervals. They represent the worst case and best case magnitude of the treatment effects for the examined clinical risk factors.

Table e4.33: Cost per QALY (patient level; discounted; 10-yr time horizon)

| Scenario | Outcome | Usual care | DMW (WCS / BCS) | Difference (WCS / BCS) |
| --- | --- | --- | --- | --- |
| 1-year effects & 2-year costs (base case) | LYs | 7·532 | 7·540 (7·535 / 7·546) | 0·009 (0·003 / 0·014) |
|  | QALYs | 6·003 | 6·011 (6·006 / 6·017) | 0·008 (0·003 / 0·013) |
|  | Costs |  |  |  |
|  | DMW intervention | - | 38 (38 / 38) | 38 (38 / 38) |
|  | Diabetes medicines | 1,548 | 1,475 (1,474 / 1,476) | −73 (−74 / −72) |
|  | Long-term complications | 15,769 | 15,750 (15,764 / 15,737) | −19 (−5 / −33) |
|  | Total cost | 17,317 | 17,263 (17,276 / 17,251) | −55 (−42 / −67) |
|  | Cost per QALY | - | - | Dominant (Dominant / Dominant) |
|  | Cost per QALY (exc. meds) | - | - | 2,253 (11,232 / 374) |
| Permanent costs & effects | LYs | 7·532 | 7·551 (7·538 / 7·564) | 0·019 (0·006 / 0·032) |
|  | QALYs | 6·003 | 6·022 (6·009 / 6·034) | 0·018 (0·006 / 0·030) |
|  | Costs |  |  |  |
|  | DMW intervention | - | 149 (148 / 149) | 149 (148 / 149) |
|  | Diabetes medicines | 1,548 | 1,266 (1,256 / 1,276) | −282 (−292 / −272) |
|  | Long-term complications | 15,769 | 15,699 (15,750 / 15,649) | −71 (−20 / −120) |
|  | Total cost | 17,317 | 17,113 (17,155 / 17,074) | −204 (−163 / −244) |
|  | Cost per QALY | - | - | Dominant (Dominant / Dominant) |
|  | Cost per QALY (exc. meds) | - | - | 4,218 (22,101 / 947) |
| 5-year costs & effects | LYs | 7·532 | 7·548 (7·537 / 7·559) | 0·017 (0·005 / 0·027) |
|  | QALYs | 6·003 | 6·019 (6·008 / 6·030) | 0·016 (0·005 / 0·026) |
|  | Costs |  |  |  |
|  | DMW intervention | - | 86 (86 / 86) | 86 (86 / 86) |
|  | Diabetes medicines | 1,548 | 1,381 (1,377 / 1,384) | −167 (−171 / −164) |
|  | Long-term complications | 15,769 | 15,720 (15,756 / 15,686) | −49 (−14 / −83) |
|  | Total cost | 17,317 | 17,187 (17,219 / 17,156) | −130 (−98 / −161) |
|  | Cost per QALY | - | - | Dominant (Dominant / Dominant) |
|  | Cost per QALY (exc. meds) | - | - | 2,317 (14,319 / 131) |
| 1-year effects  with immediate reversion; 2-year costs | LYs | 7·532 | 7·535 (7·533 / 7·537) | 0·003 (0·001 / 0·005) |
|  | QALYs | 6·003 | 6·006 (6·004 / 6·008) | 0·003 (0·001 / 0·005) |
|  | Costs |  |  |  |
|  | DMW intervention | - | 38 (38 / 38) | 38 (38 / 38) |
|  | Diabetes medicines | 1,548 | 1,473 (1,473 / 1,474) | −75 (−75 / −74) |
|  | Long-term complications | 15,769 | 15,766 (15,769 / 15,762) | −3 (0 / −8) |
|  | Total cost | 17,317 | 17,277 (17,280 / 17,273) | −40 (−37 / −44) |
|  | Cost per QALY | - | - | Dominant (Dominant / Dominant) |
|  | Cost per QALY (exc. meds) | - | - | 11,097 (36,566 / 6,134) |

**Abbreviations**: BCS Best-Case Scenario; DMW Diabetes MyWay; LY Life-Year; QALY Quality-Adjusted Life-Year; WCS Worst-Case Scenario. Notes: WCS and BCS do not constitute confidence intervals. They represent the worst case and best case magnitude of the treatment effects for the examined clinical risk factors.

Table e4.34: Cost per QALY (patient level; discounted; 5-yr time horizon)

| Scenario | Outcome | Usual care | DMW (WCS / BCS) | Difference (WCS / BCS) |
| --- | --- | --- | --- | --- |
| 1-year effects & 2-year costs (base case) | LYs | 4·399 | 4·401 (4·399 / 4·403) | 0·003 (0·001 / 0·004) |
|  | QALYs | 3·523 | 3·526 (3·524 / 3·528) | 0·003 (0·001 / 0·005) |
|  | Costs |  |  |  |
|  | DMW intervention | - | 38 (38 / 38) | 38 (38 / 38) |
|  | Diabetes medicines | 1,548 | 1,475 (1,474 / 1,476) | −73 (−74 / −72) |
|  | Long-term complications | 8,722 | 8,700 (8,715 / 8,686) | −21 (−6 / −36) |
|  | Total cost | 10,270 | 10,213 (10,227 / 10,199) | −57 (−43 / −70) |
|  | Cost per QALY | - | - | Dominant (Dominant / Dominant) |
|  | Cost per QALY (exc. meds) | - | - | 5,805 (33,296 / 322) |
| Permanent costs & effects | LYs | 4·399 | 4·402 (4·400 / 4·405) | 0·004 (0·001 / 0·006) |
|  | QALYs | 3·523 | 3·527 (3·524 / 3·530) | 0·004 (0·001 / 0·006) |
|  | Costs |  |  |  |
|  | DMW intervention | - | 86 (86 / 86) | 86 (86 / 86) |
|  | Diabetes medicines | 1,548 | 1,390 (1,380 / 1,400) | −158 (−168 / −148) |
|  | Long-term complications | 8,722 | 8,685 (8,711 / 8,660) | −37 (−10 / −62) |
|  | Total cost | 10,270 | 10,161 (10,178 / 10,146) | −109 (−92 / −124) |
|  | Cost per QALY | - | - | Dominant (Dominant / Dominant) |
|  | Cost per QALY (exc. meds) | - | - | 12,837 (61,397 / 3,836) |
| 5-year costs & effects | LYs | 4·399 | 4·402 (4·400 / 4·405) | 0·004 (0·001 / 0·006) |
|  | QALYs | 3·523 | 3·527 (3·524 / 3·530) | 0·004 (0·001 / 0·006) |
|  | Costs |  |  |  |
|  | DMW intervention | - | 86 (86 / 86) | 86 (86 / 86) |
|  | Diabetes medicines | 1,548 | 1,381 (1,377 / 1,384) | −167 (−171 / −164) |
|  | Long-term complications | 8,722 | 8,685 (8,711 / 8,660) | −37 (−10 / −62) |
|  | Total cost | 10,270 | 10,151 (10,175 / 10,130) | −118 (−95 / −140) |
|  | Cost per QALY | - | - | Dominant (Dominant / Dominant) |
|  | Cost per QALY (exc. meds) | - | - | 12,858 (62,058 / 3,837) |
| 1-year effects  with immediate reversion; 2-year costs | LYs | 4·399 | 4·400 (4·399 / 4·401) | 0·001 (0·000 / 0·002) |
|  | QALYs | 3·523 | 3·525 (3·524 / 3·526) | 0·001 (0·000 / 0·002) |
|  | Costs |  |  |  |
|  | DMW intervention | - | 38 (38 / 38) | 38 (38 / 38) |
|  | Diabetes medicines | 1,548 | 1,473 (1,473 / 1,474) | −75 (−75 / −74) |
|  | Long-term complications | 8,722 | 8,714 (8,720 / 8,709) | −7 (−2 / −13) |
|  | Total cost | 10,270 | 10,225 (10,231 / 10,220) | −44 (−39 / −49) |
|  | Cost per QALY | - | - | Dominant (Dominant / Dominant) |
|  | Cost per QALY (exc. meds) | - | - | 20,672 (73,199 / 10,398) |

**Abbreviations**: BCS Best-Case Scenario; DMW Diabetes MyWay; LY Life-Year; QALY Quality-Adjusted Life-Year; WCS Worst-Case Scenario. Notes: WCS and BCS do not constitute confidence intervals. They represent the worst case and best case magnitude of the treatment effects for the examined clinical risk factors.

### Cost per QALY (Greater Manchester population level)

Table e4.35: Cost per QALY (population level; discounted; 40-yr time horizon; base case)

| Outcome | Usual care | DMW (WCS / BCS) | Difference (WCS / BCS) |
| --- | --- | --- | --- |
| LYs | 2,255,740 | 2,256,010 (2,255,845 / 2,256,200) | 270 (105 / 460) |
| QALYs | 1,779,428 | 1,779,675 (1,779,525 / 1,779,847) | 247 (97 / 419) |
| Costs |  |  |  |
| DMW intervention | - | 609,637 (609,639 / 609,639) | 609,637 (609,639 / 609,639) |
| Diabetes medicines | 281,781,720 | 280,596,980 (280,577,173 / 280,619,747) | −1,184,741 (−1,204,548 / −1,161,973) |
| Long-term complications | 5,319,847,861 | 5,319,791,402 (5,319,875,957 / 5,319,748,509) | −56,459 (28,096 / −99,352) |
| Total cost | 5,601,629,581 | 5,600,998,019 (5,601,062,769 / 5,600,977,895) | −631,562 (−566,812 / −651,686) |
| Cost per QALY | - | - | Dominant (Dominant / Dominant) |
| Cost per QALY (exc. meds) | - | - | 2,237 (6,566 / 1,217) |

**Abbreviations**: BCS Best-Case Scenario; DMW Diabetes MyWay; LY Life-Year; QALY Quality-Adjusted Life-Year; WCS Worst-Case Scenario. Notes: WCS and BCS do not constitute confidence intervals. They represent the worst case and best case magnitude of the treatment effects for the examined clinical risk factors.

Table e4.36: Cost per QALY (population level; discounted; 40-yr time horizon; scenarios)

| Scenario | Outcome | Usual care | DMW (WCS / BCS) | Difference (WCS / BCS) |
| --- | --- | --- | --- | --- |
| Permanent costs & effects | LYs | 2,255,740 | 2,257,693 (2,256,352 / 2,259,035) | 1,953 (612 / 3,295) |
|  | QALYs | 1,779,428 | 1,781,137 (1,779,974 / 1,782,300) | 1,709 (546 / 2,872) |
|  | Costs |  |  |  |
|  | DMW intervention | - | 4,013,268 (3,987,841 / 4,038,714) | 4,013,268 (3,987,841 / 4,038,714) |
|  | Diabetes medicines | 281,781,720 | 274,004,113 (273,893,576 / 274,114,756) | −7,777,607 (−7,888,145 / −7,666,965) |
|  | Long-term complications | 5,319,847,861 | 5,319,937,355 (5,319,990,555 / 5,319,865,192) | 89,495 (142,695 / 17,331) |
|  | Total cost | 5,601,629,581 | 5,597,954,736 (5,597,871,973 / 5,598,018,662) | −3,674,845 (−3,757,608 / −3,610,919) |
|  | Cost per QALY | - | - | Dominant (Dominant / Dominant) |
|  | Cost per QALY (excluding medicines) | - | - | 2,401 (7,559 / 1,412) |
| 5-year costs & effects | LYs | 2,255,740 | 2,256,408 (2,255,962 / 2,256,822) | 668 (222 / 1,082) |
|  | QALYs | 1,779,428 | 1,780,033 (1,779,633 / 1,780,406) | 605 (205 / 978) |
|  | Costs |  |  |  |
|  | DMW intervention | - | 1,398,578 (1,398,009 / 1,399,127) | 1,398,578 (1,398,009 / 1,399,127) |
|  | Diabetes medicines | 281,781,720 | 279,069,532 (279,017,209 / 279,118,126) | −2,712,188 (−2,764,512 / −2,663,595) |
|  | Long-term complications | 5,319,847,861 | 5,319,673,536 (5,319,870,761 / 5,319,491,558) | −174,325 (22,901 / −356,302) |
|  | Total cost | 5,601,629,581 | 5,600,141,646 (5,600,285,979 / 5,600,008,811) | −1,487,935 (−1,343,602 / −1,620,770) |
|  | Cost per QALY | - | - | Dominant (Dominant / Dominant) |
|  | Cost per QALY (excluding medicines) | - | - | 2,024 (6,940 / 1,066) |
| 1-year effects  with immediate reversion; 2-year costs | LYs | 2,255,740 | 2,255,821 (2,255,772 / 2,255,874) | 81 (33 / 134) |
|  | QALYs | 1,779,428 | 1,779,501 (1,779,457 / 1,779,550) | 73 (29 / 122) |
|  | Costs |  |  |  |
|  | DMW intervention | - | 609,638 (609,637 / 609,636) | 609,638 (609,637 / 609,636) |
|  | Diabetes medicines | 281,781,720 | 280,574,340 (280,568,482 / 280,580,653) | −1,207,381 (−1,213,238 / −1,201,068) |
|  | Long-term complications | 5,319,847,861 | 5,319,884,028 (5,319,919,565 / 5,319,831,559) | 36,167 (71,704 / −16,302) |
|  | Total cost | 5,601,629,581 | 5,601,068,005 (5,601,097,684 / 5,601,021,848) | −561,576 (−531,897 / −607,733) |
|  | Cost per QALY | - | - | Dominant (Dominant / Dominant) |
|  | Cost per QALY (excluding medicines) | - | - | 8,842 (23,845 / 4,872) |

**Abbreviations**: BCS Best-Case Scenario; DMW Diabetes MyWay; LY Life-Year; QALY Quality-Adjusted Life-Year; WCS Worst-Case Scenario. Notes: WCS and BCS do not constitute confidence intervals. They represent the worst case and best case magnitude of the treatment effects for the examined clinical risk factors.

Table e4.37: Cost per QALY (population level; discounted; 10-yr time horizon)

| Scenario | Outcome | Usual care | DMW (WCS / BCS) | Difference (WCS / BCS) |
| --- | --- | --- | --- | --- |
| 1-year effects & 2-year costs (base case) | LYs | 1,370,998 | 1,371,136 (1,371,046 / 1,371,225) | 138 (47 / 226) |
|  | QALYs | 1,092,749 | 1,092,881 (1,092,796 / 1,092,967) | 133 (47 / 218) |
|  | Costs |  |  |  |
|  | DMW intervention | - | 609,637 (609,639 / 609,639) | 609,637 (609,639 / 609,639) |
|  | Diabetes medicines | 281,781,720 | 280,596,980 (280,577,173 / 280,619,747) | −1,184,741 (−1,204,548 / −1,161,973) |
|  | Long-term complications | 2,870,421,697 | 2,870,111,107 (2,870,339,894 / 2,869,893,577) | −310,590 (−81,804 / −528,120) |
|  | Total cost | 3,152,203,418 | 3,151,317,724 (3,151,526,705 / 3,151,122,963) | −885,694 (−676,713 / −1,080,455) |
|  | Cost per QALY | - | - | Dominant (Dominant / Dominant) |
|  | Cost per QALY (excluding medicines) | - | - | 2,253 (11,232 / 374) |
| Permanent costs & effects | LYs | 1,370,998 | 1,371,306 (1,371,092 / 1,371,511) | 308 (94 / 512) |
|  | QALYs | 1,092,749 | 1,093,047 (1,092,843 / 1,093,242) | 299 (94 / 494) |
|  | Costs |  |  |  |
|  | DMW intervention | - | 2,405,285 (2,401,656 / 2,408,741) | 2,405,285 (2,401,656 / 2,408,741) |
|  | Diabetes medicines | 281,781,720 | 277,214,455 (277,060,398 / 277,369,001) | −4,567,265 (−4,721,322 / −4,412,719) |
|  | Long-term complications | 2,870,421,697 | 2,869,276,154 (2,870,103,380 / 2,868,480,194) | −1,145,543 (−318,318 / −1,941,503) |
|  | Total cost | 3,152,203,418 | 3,148,895,894 (3,149,565,434 / 3,148,257,936) | −3,307,524 (−2,637,984 / −3,945,482) |
|  | Cost per QALY | - | - | Dominant (Dominant / Dominant) |
|  | Cost per QALY (excluding medicines) | - | - | 4,218 (22,101 / 947) |
| 5-year costs & effects | LYs | 1,370,998 | 1,371,266 (1,371,080 / 1,371,441) | 267 (82 / 443) |
|  | QALYs | 1,092,749 | 1,093,007 (1,092,831 / 1,093,174) | 259 (82 / 426) |
|  | Costs |  |  |  |
|  | DMW intervention | - | 1,398,578 (1,398,009 / 1,399,127) | 1,398,578 (1,398,009 / 1,399,127) |
|  | Diabetes medicines | 281,781,720 | 279,069,532 (279,017,209 / 279,118,126) | −2,712,188 (−2,764,512 / −2,663,595) |
|  | Long-term complications | 2,870,421,697 | 2,869,622,332 (2,870,200,260 / 2,869,078,162) | −799,366 (−221,437 / −1,343,535) |
|  | Total cost | 3,152,203,418 | 3,150,090,442 (3,150,615,478 / 3,149,595,415) | −2,112,976 (−1,587,940 / −2,608,003) |
|  | Cost per QALY | - | - | Dominant (Dominant / Dominant) |
|  | Cost per QALY (excluding medicines) | - | - | 2,317 (14,319 / 131) |
| 1-year effects  with immediate reversion; 2-year costs | LYs | 1,370,998 | 1,371,052 (1,371,016 / 1,371,082) | 53 (17 / 83) |
|  | QALYs | 1,092,749 | 1,092,799 (1,092,765 / 1,092,828) | 50 (17 / 79) |
|  | Costs |  |  |  |
|  | DMW intervention | - | 609,638 (609,637 / 609,636) | 609,638 (609,637 / 609,636) |
|  | Diabetes medicines | 281,781,720 | 280,574,340 (280,568,482 / 280,580,653) | −1,207,381 (−1,213,238 / −1,201,068) |
|  | Long-term complications | 2,870,421,697 | 2,870,368,990 (2,870,421,386 / 2,870,297,257) | −52,708 (−312 / −124,441) |
|  | Total cost | 3,152,203,418 | 3,151,552,967 (3,151,599,505 / 3,151,487,546) | −650,451 (−603,913 / −715,872) |
|  | Cost per QALY | - | - | Dominant (Dominant / Dominant) |
|  | Cost per QALY (excluding medicines) | - | - | 11,097 (36,566 / 6,134) |

**Abbreviations**: BCS Best-Case Scenario; DMW Diabetes MyWay; LY Life-Year; QALY Quality-Adjusted Life-Year; WCS Worst-Case Scenario. Notes: WCS and BCS do not constitute confidence intervals. They represent the worst case and best case magnitude of the treatment effects for the examined clinical risk factors.

Table e4.38: Cost per QALY (population level; discounted; 5-yr time horizon)

| Scenario | Outcome | Usual care | DMW (WCS / BCS) | Difference (WCS / BCS) |
| --- | --- | --- | --- | --- |
| 1-year effects & 2-year costs (base case) | LYs | 800,642 | 800,686 (800,657 / 800,714) | 43 (14 / 72) |
|  | QALYs | 641,316 | 641,361 (641,331 / 641,390) | 45 (15 / 75) |
|  | Costs |  |  |  |
|  | DMW intervention | - | 609,637 (609,639 / 609,639) | 609,637 (609,639 / 609,639) |
|  | Diabetes medicines | 281,781,720 | 280,596,980 (280,577,173 / 280,619,747) | −1,184,741 (−1,204,548 / −1,161,973) |
|  | Long-term complications | 1,587,562,482 | 1,587,214,621 (1,587,461,425 / 1,586,976,823) | −347,862 (−101,057 / −585,659) |
|  | Total cost | 1,869,344,203 | 1,868,421,237 (1,868,648,237 / 1,868,206,209) | −922,965 (−695,966 / −1,137,993) |
|  | Cost per QALY | - | - | Dominant (Dominant / Dominant) |
|  | Cost per QALY (excluding medicines) | - | - | 5,805 (33,296 / 322) |
| Permanent costs & effects | LYs | 800,642 | 800,701 (800,661 / 800,740) | 59 (18 / 98) |
|  | QALYs | 641,316 | 641,378 (641,336 / 641,418) | 62 (20 / 102) |
|  | Costs |  |  |  |
|  | DMW intervention | - | 1,398,583 (1,398,014 / 1,399,127) | 1,398,583 (1,398,014 / 1,399,127) |
|  | Diabetes medicines | 281,781,720 | 279,224,337 (279,064,172 / 279,384,696) | −2,557,384 (−2,717,549 / −2,397,024) |
|  | Long-term complications | 1,587,562,482 | 1,586,963,340 (1,587,393,150 / 1,586,556,391) | −599,142 (−169,332 / −1,006,092) |
|  | Total cost | 1,869,344,203 | 1,867,586,260 (1,867,855,335 / 1,867,340,214) | −1,757,942 (−1,488,867 / −2,003,989) |
|  | Cost per QALY | - | - | Dominant (Dominant / Dominant) |
|  | Cost per QALY (excluding medicines) | - | - | 12,837 (61,397 / 3,836) |
| 5-year costs & effects | LYs | 800,642 | 800,701 (800,660 / 800,740) | 59 (18 / 98) |
|  | QALYs | 641,316 | 641,378 (641,336 / 641,418) | 62 (20 / 103) |
|  | Costs |  |  |  |
|  | DMW intervention | - | 1,398,578 (1,398,009 / 1,399,127) | 1,398,578 (1,398,009 / 1,399,127) |
|  | Diabetes medicines | 281,781,720 | 279,069,532 (279,017,209 / 279,118,126) | −2,712,188 (−2,764,512 / −2,663,595) |
|  | Long-term complications | 1,587,562,482 | 1,586,961,013 (1,587,392,750 / 1,586,556,703) | −601,470 (−169,732 / −1,005,780) |
|  | Total cost | 1,869,344,203 | 1,867,429,123 (1,867,807,968 / 1,867,073,955) | −1,915,080 (−1,536,235 / −2,270,247) |
|  | Cost per QALY | - | - | Dominant (Dominant / Dominant) |
|  | Cost per QALY (excluding medicines) | - | - | 12,858 (62,058 / 3,837) |
| 1-year effects  with immediate reversion; 2-year costs | LYs | 800,642 | 800,666 (800,650 / 800,681) | 23 (8 / 38) |
|  | QALYs | 641,316 | 641,340 (641,324 / 641,355) | 24 (8 / 39) |
|  | Costs |  |  |  |
|  | DMW intervention | - | 609,638 (609,637 / 609,636) | 609,638 (609,637 / 609,636) |
|  | Diabetes medicines | 281,781,720 | 280,574,340 (280,568,482 / 280,580,653) | −1,207,381 (−1,213,238 / −1,201,068) |
|  | Long-term complications | 1,587,562,482 | 1,587,443,632 (1,587,532,856 / 1,587,356,893) | −118,850 (−29,627 / −205,590) |
|  | Total cost | 1,869,344,203 | 1,868,627,609 (1,868,710,975 / 1,868,547,182) | −716,593 (−633,228 / −797,021) |
|  | Cost per QALY | - | - | Dominant (Dominant / Dominant) |
|  | Cost per QALY (excluding medicines) | - | - | 20,672 (73,199 / 10,398) |

**Abbreviations**: BCS Best-Case Scenario; DMW Diabetes MyWay; LY Life-Year; QALY Quality-Adjusted Life-Year; WCS Worst-Case Scenario. Notes: WCS and BCS do not constitute confidence intervals. They represent the worst case and best case magnitude of the treatment effects for the examined clinical risk factors.

Figure e4.08: Cost-effectiveness of Diabetes My Way as a function of cost and uptake

| 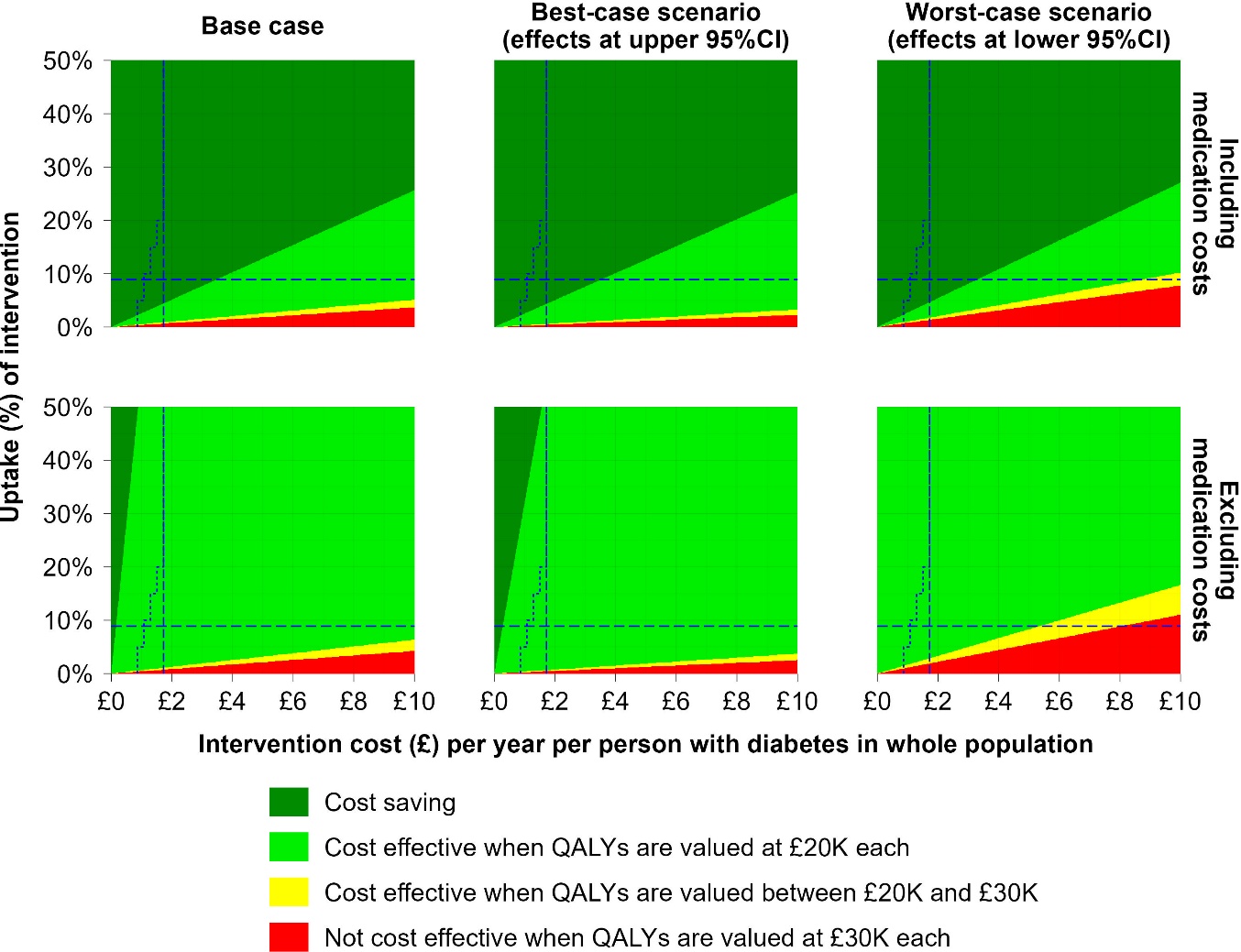 |
| --- |
| Horizontal dashed: current uptake in Greater Manchester (16,194 of 182,025 people with type 2 diabetes = 8·9%)  Vertical dashed: full GMDMW cost (licence fee = £313,557 per year; eligible population in Greater Manchester = 182,025; £1·72 per person per year)  Vertical dotted (staggered): full GMDMW cost given the current commercial arrangement (initial 50% discount; rising to full price once uptake reaches 20%; equal steps for every 5 percentage-points of uptake in between) |

# Consolidated Health Economic Evaluation Reporting Standards 2022 (CHEERS 2022) Checklist

Table e5.01: CHEERS 2022 Checklist

| **Section/topic** | **Item No** | **Guidance for reporting** | **Reported in section** |
| --- | --- | --- | --- |
| **Title** | | | |
| Title | 1 | Identify the study as an economic evaluation and specify the interventions being compared. | Title and Page 3 |
| **Abstract** | | | |
| Abstract | 2 | Provide a structured summary that highlights context, key methods, results, and alternative analyses. | Abstract |
| **Introduction** | | | |
| Background and objectives | 3 | Give the context for the study, the study question, and its practical relevance for decision making in policy or practice. | Page 3 |
| **Methods** | | | |
| Health economic analysis plan | 4 | Indicate whether a health economic analysis plan was developed and where available. | N/A |
| Study population | 5 | Describe characteristics of the study population (such as age range, demographics, socioeconomic, or clinical characteristics). | Pages 3-4 |
| Setting and location | 6 | Provide relevant contextual information that may influence findings. | Page 3 |
| Comparators | 7 | Describe the interventions or strategies being compared and why chosen. | Page 3-3 |
| Perspective | 8 | State the perspective(s) adopted by the study and why chosen. | Page 3 |
| Time horizon | 9 | State the time horizon for the study and why appropriate. | Page 3 |
| Discount rate | 10 | Report the discount rate(s) and reason chosen. | Page 3 |
| Selection of outcomes | 11 | Describe what outcomes were used as the measure(s) of benefit(s) and harm(s). | Page 3 and supplementary material |
| Measurement of outcomes | 12 | Describe how outcomes used to capture benefit(s) and harm(s) were measured. | Page 3 and supplementary material |
| Valuation of outcomes | 13 | Describe the population and methods used to measure and value outcomes. | Pages 3-4 and supplementary material |
| Measurement and valuation of resources and costs | 14 | Describe how costs were valued. | Pages 3-4 and supplementary material |
| Currency, price date, and conversion | 15 | Report the dates of the estimated resource quantities and unit costs, plus the currency and year of conversion. | Page 3 |
| Rationale and description of model | 16 | If modelling is used, describe in detail and why used. Report if the model is publicly available and where it can be accessed. | Page 3 |
| Analytics and assumptions | 17 | Describe any methods for analysing or statistically transforming data, any extrapolation methods, and approaches for validating any model used. | Pages 3-4 and supplementary material |
| Characterising heterogeneity | 18 | Describe any methods used for estimating how the results of the study vary for subgroups. | Page 4 |
| Characterising distributional effects | 19 | Describe how impacts are distributed across different individuals or adjustments made to reflect priority populations. | N/A |
| Characterising uncertainty | 20 | Describe methods to characterise any sources of uncertainty in the analysis. | Pages 3-4 |
| Approach to engagement with patients and others affected by the study | 21 | Describe any approaches to engage patients or service recipients, the general public, communities, or stakeholders (such as clinicians or payers) in the design of the study. | N/A |
| **Results** | | | |
| Study parameters | 22 | Report all analytic inputs (such as values, ranges, references) including uncertainty or distributional assumptions. | Page 5 and supplementary material |
| Summary of main results | 23 | Report the mean values for the main categories of costs and outcomes of interest and summarise them in the most appropriate overall measure. | Page 5 and supplementary material |
| Effect of uncertainty | 24 | Describe how uncertainty about analytic judgments, inputs, or projections affect findings. Report the effect of choice of discount rate and time horizon, if applicable. | Page 5 and supplementary material |
| Effect of engagement with patients and others affected by the study | 25 | Report on any difference patient/service recipient, general public, community, or stakeholder involvement made to the approach or findings of the study | N/A |
| **Discussion** | | | |
| Study findings, limitations, generalisability, and current knowledge | 26 | Report key findings, limitations, ethical or equity considerations not captured, and how these could affect patients, policy, or practice. | Pages 6-8 |
| **Other relevant information** | | | |
| Source of funding | 27 | Describe how the study was funded and any role of the funder in the identification, design, conduct, and reporting of the analysis | Abstract |
| Conflicts of interest | 28 | Report authors conflicts of interest according to journal or International Committee of Medical Journal Editors requirements. | Page 8 |

Husereau D, Drummond M, Augustovski F, de Bekker-Grob E, Briggs AH, Carswell C, Caulley L, Chaiyakunapruk N, Greenberg D, Loder E, Mauskopf J, Mullins CD, Petrou S, Pwu RF, Staniszewska S; CHEERS 2022 ISPOR Good Research Practices Task Force. Consolidated Health Economic Evaluation Reporting Standards 2022 (CHEERS 2022) Statement: Updated Reporting Guidance for Health Economic Evaluations. BMJ. 2022;376:e067975.

The checklist is Open Access distributed in accordance with the terms of the Creative Commons Attribution (CC BY 4·0) license, which permits others to distribute, remix, adapt and build upon this work, for commercial use, provided the original work is properly cited. See: [http://creativecommons.org/licenses/by/4·0/.](http://creativecommons.org/licenses/by/4.0/)

# References

1. Goldthorpe J, Allen T, Brooks J, Kontopantelis E, Holland F, Moss C, et al. Digital Interventions Supporting Self-care in People With Type 2 Diabetes Across Greater Manchester (Greater Manchester Diabetes My Way): Protocol for a Mixed Methods Evaluation. JMIR Res Protoc. 2022;11(8):e26237.

2. National Institute for Health and Care Excellence. NICE health technology evaluations: the manual. London: National Institute for Health and Care Excellence; 2022.

3. Husereau D, Drummond M, Augustovski F, De Bekker-Grob E, Briggs AH, Carswell C, et al. Consolidated Health Economic Evaluation Reporting Standards 2022 (CHEERS 2022) statement: updated reporting guidance for health economic evaluations. BMJ. 2022:e067975.

4. University of Oxford. United Kingdom Prospective Diabetes Study (UKPDS Outcomes Model [Available from: <https://www.dtu.ox.ac.uk/outcomesmodel/>].

5. Hayes AJ, Leal J, Gray AM, Holman RR, Clarke PM. UKPDS outcomes model 2: a new version of a model to simulate lifetime health outcomes of patients with type 2 diabetes mellitus using data from the 30 year United Kingdom Prospective Diabetes Study: UKPDS 82. Diabetologia. 2013;56(9):1925−33.

6. National Institute for Health and Care Excellence. Type 2 diabetes in adults: management (update). Health economic model report (NICE guideline NG28). London: National Institute for Health and Care Excellence; 2022.

7. National Health Service (NHS). The Health Improvement Network (THIN) 2020 [Available from: <https://www.the-health-improvement-network.com/>].

8. Palmer AJ, Roze S, Valentine WJ, Minshall ME, Foos V, Lurati FM, et al. The CORE Diabetes Model: Projecting long-term clinical outcomes, costs and cost-effectiveness of interventions in diabetes mellitus (types 1 and 2) to support clinical and reimbursement decision-making. Curr Med Res Opin. 2004;20 Suppl 1:S5−26.

9. Adler AI, Stevens RJ, Manley SE, Bilous RW, Cull CA, Holman RR. Development and progression of nephropathy in type 2 diabetes: the United Kingdom Prospective Diabetes Study (UKPDS 64). Kidney Int. 2003;63(1):225−32.

10. Leal J, Alva M, Gregory V, Hayes A, Mihaylova B, Gray AM, et al. Estimating risk factor progression equations for the UKPDS Outcomes Model 2 (UKPDS 90). Diabetic Medicine. 2021;38(10):e14656.

11. National Health Service (NHS) Business Service Authority. Electronic Drug Tariff. 2023(June).

12. Alva ML, Gray A, Mihaylova B, Leal J, Holman RR. The impact of diabetes-related complications on healthcare costs: new results from the UKPDS (UKPDS 84). 2015;32:459−66.

13. Curtis LA, Burns A. Unit Costs of Health & Social Care 2020: PSSRU, University of Kent; 2020 2020/12/16.

14. Jones KC, Weatherly H, Birch S, Castelli A, Chalkley M, Dargan A, et al. *Unit Costs of Health and Social Care 2022 Manual*. Canterbury: Personal Social Services Research Unit (University of Kent) & Centre for Health Economics (University of York); 2023.

15. Li B, Cairns J, Fotheringham J, Ravanan R. Predicting hospital costs for patients receiving renal replacement therapy to inform an economic evaluation. Eur J Health Econ. 2016;17(6):659−68.

16. United Kingdom (UK) Renal Association. UK Renal Registry 22nd Annual Report – data to 31/12/2018. Bristol, UK; 2020.

17. National Health Service (NHS) England. National Schedule of NHS Costs 2020/21 [Available from: [https://www.england.nhs.uk/publication/2020−21-national-cost-collection-data-publication/](https://www.england.nhs.uk/publication/2020-21-national-cost-collection-data-publication/)].

18. Scotland G, Cruickshank M, Jacobsen E, Cooper D, Fraser C, Shimonovich M, et al. Multiple-frequency bioimpedance devices for fluid management in people with chronic kidney disease receiving dialysis: a systematic review and economic evaluation. Health Technol Assess. 2018;22(1):1−138.

19. Kerr M, Barron E, Chadwick P, Evans T, Kong WM, Rayman G, et al. The cost of diabetic foot ulcers and amputations to the National Health Service in England. Diabet Med. 2019;36(8):995−1002.

20. Kerr M. Foot Care for People with Diabetes: The Economic Case for Change. 2012.

21. Alva M, Gray A, Mihaylova B, Clarke P. The effect of diabetes complications on health-related quality of life: the importance of longitudinal data to address patient heterogeneity. Health Econ. 2014;23(4):487−500.

22. Lung TW, Hayes AJ, Hayen A, Farmer A, Clarke PM. A meta-analysis of health state valuations for people with diabetes: explaining the variation across methods and implications for economic evaluation. Qual Life Res. 2011;20(10):1669−78.

23. Schlackow I, Kent S, Herrington W, Emberson J, Haynes R, Reith C, et al. A policy model of cardiovascular disease in moderate-to-advanced chronic kidney disease. Heart. 2017;103(23):1880−90.

24. Li B, Cairns JA, Draper H, Dudley C, Forsythe JL, Johnson RJ, et al. Estimating Health-State Utility Values in Kidney Transplant Recipients and Waiting-List Patients Using the EQ−5D−5L. Value Health. 2017;20(7):976−84.

25. National Health Service (NHS) Blood and Transplant. Factsheet 7: Cost-effectiveness of transplantation. London, UK.; 2012.
