# Supplementary material for: Clinical and cost-effectiveness of the digital intervention, MyWay Diabetes, in people with type 2 diabetes living in Greater Manchester during the COVID-19 pandemic
Source: PLoS One. 2026 Jun 25;21(6):e0349232. doi: 10.1371/journal.pone.0349232 (PMC13298753; doi:10.1371/journal.pone.0349232)
Supplement: Attachment — Submitted filename: Response to Reviewers.docx [file pone.0349232.s004.docx]

PONE-D-25-41865

Clinical and cost-effectiveness of the digital intervention, MyWay Diabetes, in people with type 2 diabetes living in Greater Manchester during the COVID-19 pandemic.

PLOS ONE

Dear Dr.  Ravindrarajah,

Thank you for submitting your manuscript to PLOS ONE. After careful consideration, we feel that it has merit but does not fully meet PLOS ONE’s publication criteria as it currently stands. Therefore, we invite you to submit a revised version of the manuscript that addresses the points raised during the review process.

Please submit your revised manuscript by Dec 12 2025 11:59PM. If you will need more time than this to complete your revisions, please reply to this message or contact the journal office at [plosone@plos.org](mailto:plosone@plos.org). When you're ready to submit your revision, log on to [https://www.editorialmanager.com/pone/ [editorialmanager.com]](https://urldefense.com/v3/__https:/www.editorialmanager.com/pone/__;!!PDiH4ENfjr2_Jw!C1K40HAWArCysU31bhiuPj6NtplKVA_yhE9ok2V64BTdZh1vohyVGmZYfoODzJeXFF7AOhEn2glA37Z_NOr9BbherD6CHRA$) and select the 'Submissions Needing Revision' folder to locate your manuscript file.

Please include the following items when submitting your revised manuscript:

- A rebuttal letter that responds to each point raised by the academic editor and reviewer(s). You should upload this letter as a separate file labeled 'Response to Reviewers'.
- A marked-up copy of your manuscript that highlights changes made to the original version. You should upload this as a separate file labeled 'Revised Manuscript with Track Changes'.
- An unmarked version of your revised paper without tracked changes. You should upload this as a separate file labeled 'Manuscript'.

If you would like to make changes to your financial disclosure, please include your updated statement in your cover letter. Guidelines for resubmitting your figure files are available below the reviewer comments at the end of this letter.

If applicable, we recommend that you deposit your laboratory protocols in protocols.io to enhance the reproducibility of your results. Protocols.io assigns your protocol its own identifier (DOI) so that it can be cited independently in the future. For instructions see: [https://journals.plos.org/plosone/s/submission-guidelines#loc-laboratory-protocols [track.editorialmanager.com]](https://urldefense.com/v3/__https:/track.editorialmanager.com/CL0/https:*2F*2Fjournals.plos.org*2Fplosone*2Fs*2Fsubmission-guidelines*23loc-laboratory-protocols/1/010f019a2dff0555-7f7ccaec-b8e1-488a-a889-062d52f9f64a-000000/wDZKpPNoqpQd_RgDc88Hxlnd1HX4fDsS_4liqVUSG28=235__;JSUlJSUl!!PDiH4ENfjr2_Jw!C1K40HAWArCysU31bhiuPj6NtplKVA_yhE9ok2V64BTdZh1vohyVGmZYfoODzJeXFF7AOhEn2glA37Z_NOr9Bbhe2_pb2PQ$). Additionally, PLOS ONE offers an option for publishing peer-reviewed Lab Protocol articles, which describe protocols hosted on protocols.io. Read more information on sharing protocols at [https://plos.org/protocols?utm_medium=editorial-email&utm_source=authorletters&utm_campaign=protocols [track.editorialmanager.com]](https://urldefense.com/v3/__https:/track.editorialmanager.com/CL0/https:*2F*2Fplos.org*2Fprotocols*3Futm_medium=editorial-email*26utm_source=authorletters*26utm_campaign=protocols/1/010f019a2dff0555-7f7ccaec-b8e1-488a-a889-062d52f9f64a-000000/hnsCyoMO_k9p53sD6bAjh72GhbPHEHctLNYHs8jSi8Q=235__;JSUlJSUl!!PDiH4ENfjr2_Jw!C1K40HAWArCysU31bhiuPj6NtplKVA_yhE9ok2V64BTdZh1vohyVGmZYfoODzJeXFF7AOhEn2glA37Z_NOr9Bbhel1WLJiI$).

We look forward to receiving your revised manuscript.

Kind regards,

Ennio Polilli
Academic Editor
PLOS ONE

…………………………………………………………………………………………………………………………………..

**Journal Requirements:**

When submitting your revision, we need you to address these additional requirements.

1. Please ensure that your manuscript meets PLOS ONE's style requirements, including those for file naming. The PLOS ONE style templates can be found at

[https://journals.plos.org/plosone/s/file?id=wjVg/PLOSOne_formatting_sample_main_body.pdf [journals.plos.org]](https://urldefense.com/v3/__https:/journals.plos.org/plosone/s/file?id=wjVg*PLOSOne_formatting_sample_main_body.pdf__;Lw!!PDiH4ENfjr2_Jw!C1K40HAWArCysU31bhiuPj6NtplKVA_yhE9ok2V64BTdZh1vohyVGmZYfoODzJeXFF7AOhEn2glA37Z_NOr9BbheTBQnXsc$) and

[https://journals.plos.org/plosone/s/file?id=ba62/PLOSOne_formatting_sample_title_authors_affiliations.pdf [track.editorialmanager.com]](https://urldefense.com/v3/__https:/track.editorialmanager.com/CL0/https:*2F*2Fjournals.plos.org*2Fplosone*2Fs*2Ffile*3Fid=ba62*2FPLOSOne_formatting_sample_title_authors_affiliations.pdf/1/010f019a2dff0555-7f7ccaec-b8e1-488a-a889-062d52f9f64a-000000/t6pCmzWB6fM02jTvf9FAq8ekdE6uUBuwz4MyByBLggc=235__;JSUlJSUlJQ!!PDiH4ENfjr2_Jw!C1K40HAWArCysU31bhiuPj6NtplKVA_yhE9ok2V64BTdZh1vohyVGmZYfoODzJeXFF7AOhEn2glA37Z_NOr9BbheuBcTiu4$)

2. Please provide additional details regarding participant consent. In the ethics statement in the Methods and online submission information, please ensure that you have specified what type you obtained (for instance, written or verbal, and if verbal, how it was documented and witnessed). If your study included minors, state whether you obtained consent from parents or guardians. If the need for consent was waived by the ethics committee, please include this information.

Once you have amended this/these statement(s) in the Methods section of the manuscript, please add the same text to the “Ethics Statement” field of the submission form (via “Edit Submission”).

For additional information about PLOS ONE ethical requirements for human subjects research, please refer to [http://journals.plos.org/plosone/s/submission-guidelines#loc-human-subjects-research [track.editorialmanager.com]](https://urldefense.com/v3/__http:/track.editorialmanager.com/CL0/http:*2F*2Fjournals.plos.org*2Fplosone*2Fs*2Fsubmission-guidelines*23loc-human-subjects-research/1/010f019a2dff0555-7f7ccaec-b8e1-488a-a889-062d52f9f64a-000000/SCSozfsCPcXHesa2AS55SbhKEFiLeHWyqOl7ASqRUYs=235__;JSUlJSUl!!PDiH4ENfjr2_Jw!C1K40HAWArCysU31bhiuPj6NtplKVA_yhE9ok2V64BTdZh1vohyVGmZYfoODzJeXFF7AOhEn2glA37Z_NOr9Bbhe9jtWVUU$).

3. Thank you for stating the following financial disclosure:

“Innovate UK and NHS England (ref: 104658)”

Please state what role the funders took in the study.  If the funders had no role, please state:

**Author response*: "The funders had no role in study design, data collection and analysis, decision to publish, or preparation of the manuscript."***

Please include this amended Role of Funder statement in your cover letter; we will change the online submission form on your behalf.

4. Please note that funding information should not appear in any section or other areas of your manuscript. We will only publish funding information present in the Funding Statement section of the online submission form. Please remove any funding-related text from the manuscript.

5. Thank you for stating the following in the Competing Interests section:

***“Doogie Brodie is development manager at MyWay Digital Health. Deborah J Wake is a co-founder and shareholder of MyWay Digital Health (MWDH). Martin K Rutter reports consulting fees from Eli Lilly and Brigham and Women’s Hospital and modest stock ownership in GSK, all unrelated to this work. Michael Morgan-Curran is CEO at Asclepius MedTech Limited, unrelated to this work. Scott G Cunningham is co-founder, director and CTO at MyWay Digital Health and reports grants from Scottish Government, outside the submitted work; personal fees, non-financial support and other from MyWay Digital Health. Naresh Kanumilli reports fees for lectures and advisory boards for BI, Lily, AZ, Novartis, Sanofi Manarini, Novo, Abbott, unrelated to this work. George Tilston is funded by the National Institute for Health and Care Research (NIHR) Manchester Biomedical Research Centre (BRC) (NIHR203308)***

***All other authors report no conflict of interests. All authors have completed the conflict-of-interest disclosure forms.”***

***In accordance with Innovate UK’s funding agreement, the University of Manchester team conducted data analysis and manuscript writing independently of MyWay Digital Health. Both teams collaborated on study design and the Innovate UK funding application, and the MyWay Digital Health team provided feedback on the final manuscript draft; however, they did not influence the study’s results or conclusions.”***

We note that one or more of the authors are employed by a commercial company: MyWay Digital Health (MWDH)

1. Please provide an amended Funding Statement declaring this commercial affiliation, as well as a statement regarding the Role of Funders in your study. If the funding organization did not play a role in the study design, data collection and analysis, decision to publish, or preparation of the manuscript and only provided financial support in the form of authors' salaries and/or research materials, please review your statements relating to the author contributions, and ensure you have specifically and accurately indicated the role(s) that these authors had in your study. You can update author roles in the Author Contributions section of the online submission form.

Please also include the following statement within your amended Funding Statement.

“The funder provided support in the form of salaries for authors [insert relevant initials], but did not have any additional role in the study design, data collection and analysis, decision to publish, or preparation of the manuscript. The specific roles of these authors are articulated in the ‘author contributions’ section.”

If your commercial affiliation did play a role in your study, please state and explain this role within your updated Funding Statement.

**Author response**: **Funding statement: Grant funding was provided by Innovate UK and NHS England (ref: 104658). The funders had no role in study design, data collection and analysis, decision to publish, or preparation of the manuscript. The funders provided support in the form of salaries for some co-authors employed by MyWay Digital Health [DJW, DB, SGC]. These individuals did not have a role in the study analysis, developing study conclusions or the decision to publish. The specific roles of these authors are articulated in the ‘author contributions’ section. In summary, all listed co-authors who are employees of MyWay Digital Health justify their authorship through contributions to the conception of the study, study design, obtaining funding, study set up and reviewing the final manuscript.**

2. Please also provide an updated Competing Interests Statement declaring this commercial affiliation along with any other relevant declarations relating to employment, consultancy, patents, products in development, or marketed products, etc.

Within your Competing Interests Statement, please confirm that this commercial affiliation does not alter your adherence to all PLOS ONE policies on sharing data and materials by including the following statement: "This does not alter our adherence to PLOS ONE policies on sharing data and materials.” (as detailed online in our guide for authors [http://journals.plos.org/plosone/s/competing-interests [journals.plos.org]](https://urldefense.com/v3/__http:/journals.plos.org/plosone/s/competing-interests__;!!PDiH4ENfjr2_Jw!C1K40HAWArCysU31bhiuPj6NtplKVA_yhE9ok2V64BTdZh1vohyVGmZYfoODzJeXFF7AOhEn2glA37Z_NOr9BbhedAV9w6U$)) . If this adherence statement is not accurate and there are restrictions on sharing of data and/or materials, please state these. Please note that we cannot proceed with consideration of your article until this information has been declared.

Please include both an updated Funding Statement and Competing Interests Statement in your cover letter. We will change the online submission form on your behalf.

**Author’s Response:**

**We have added the following statement in the manuscript. The commercial affiliations above do not alter our adherence to PLOS ONE policies on sharing data and materials.**

6. We note that you have indicated that there are restrictions to data sharing for this study. PLOS only allows data to be available upon request if there are legal or ethical restrictions on sharing data publicly. For more information on unacceptable data access restrictions, please see [http://journals.plos.org/plosone/s/data-availability#loc-unacceptable-data-access-restrictions [journals.plos.org]](https://urldefense.com/v3/__http:/journals.plos.org/plosone/s/data-availability*loc-unacceptable-data-access-restrictions__;Iw!!PDiH4ENfjr2_Jw!C1K40HAWArCysU31bhiuPj6NtplKVA_yhE9ok2V64BTdZh1vohyVGmZYfoODzJeXFF7AOhEn2glA37Z_NOr9BbhewhdwRT4$).

Before we proceed with your manuscript, please address the following prompts:

a) If there are ethical or legal restrictions on sharing a de-identified data set, please explain them in detail (e.g., data contain potentially identifying or sensitive patient information, data are owned by a third-party organization, etc.) and who has imposed them (e.g., a Research Ethics Committee or Institutional Review Board, etc.). Please also provide contact information for a data access committee, ethics committee, or other institutional body to which data requests may be sent.

**Author response: There are restrictions on the sharing of data used in this research. GP practices and NHS organisations (e.g., NHS Greater Manchester and individual practices) are the data controllers of the primary care records used in our analysis. Patients own their personal health information under GDPR/UK data protection law, with rights to opt-out of secondary uses of their data (e.g., research) via national and local mechanisms. Applications to access to these data for research purposes can be made through Health Innovation Manchester with whom these data are shared under strict governance arrangements:** [**https://healthinnovationmanchester.com/gms-secure-data-environment-sde-for-health-and-care/**](https://healthinnovationmanchester.com/gms-secure-data-environment-sde-for-health-and-care/)

b) If there are no restrictions, please upload the minimal anonymized data set necessary to replicate your study findings to a stable, public repository and provide us with the relevant URLs, DOIs, or accession numbers.

**Author response: These data are restricted for the reasons mentioned above.**

c) For a list of recommended repositories, please see

[https://journals.plos.org/plosone/s/recommended-repositories [journals.plos.org]](https://urldefense.com/v3/__https:/journals.plos.org/plosone/s/recommended-repositories__;!!PDiH4ENfjr2_Jw!C1K40HAWArCysU31bhiuPj6NtplKVA_yhE9ok2V64BTdZh1vohyVGmZYfoODzJeXFF7AOhEn2glA37Z_NOr9BbheLnXH5dQ$). You also have the option of uploading the data as Supporting Information files, but we would recommend depositing data directly to a data repository if possible.

We will update your Data Availability statement on your behalf to reflect the information you provide.

7. If the reviewer comments include a recommendation to cite specific previously published works, please review and evaluate these publications to determine whether they are relevant and should be cited. There is no requirement to cite these works unless the editor has indicated otherwise. 

[Note: HTML markup is below. Please do not edit.]

…………………………………………………………………………………………………………………………………..

**Reviewer's Responses to Questions**

**Comments to the Author**

1. Is the manuscript technically sound, and do the data support the conclusions?

The manuscript must describe a technically sound piece of scientific research with data that supports the conclusions. Experiments must have been conducted rigorously, with appropriate controls, replication, and sample sizes. The conclusions must be drawn appropriately based on the data presented.

Reviewer #1: Yes

Reviewer #2: Yes

Reviewer #3: Yes

Reviewer #4: Yes

2. Has the statistical analysis been performed appropriately and rigorously?

Reviewer #1: Yes

Reviewer #2: No

Reviewer #3: Yes

Reviewer #4: Yes

3. Have the authors made all data underlying the findings in their manuscript fully available?

The [PLOS Data policy [track.editorialmanager.com]](https://urldefense.com/v3/__http:/track.editorialmanager.com/CL0/http:*2F*2Fwww.plosone.org*2Fstatic*2Fpolicies.action*23sharing/1/010f019a2dff0555-7f7ccaec-b8e1-488a-a889-062d52f9f64a-000000/Ch40dgzahZ7oCtO47yJGszzeOyCURSniqQ0eymdUjwI=235__;JSUlJSU!!PDiH4ENfjr2_Jw!C1K40HAWArCysU31bhiuPj6NtplKVA_yhE9ok2V64BTdZh1vohyVGmZYfoODzJeXFF7AOhEn2glA37Z_NOr9Bbhe2CtSwpI$) requires authors to make all data underlying the findings described in their manuscript fully available without restriction, with rare exception (please refer to the Data Availability Statement in the manuscript PDF file). The data should be provided as part of the manuscript or its supporting information, or deposited to a public repository. For example, in addition to summary statistics, the data points behind means, medians and variance measures should be available. If there are restrictions on publicly sharing data—e.g. participant privacy or use of data from a third party—those must be specified.

Reviewer #1: Yes

Reviewer #2: No

Reviewer #3: No

Reviewer #4: No

4. Is the manuscript presented in an intelligible fashion and written in standard English?

PLOS ONE does not copyedit accepted manuscripts, so the language in submitted articles must be clear, correct, and unambiguous. Any typographical or grammatical errors should be corrected at revision, so please note any specific errors here.

Reviewer #1: Yes

Reviewer #2: Yes

Reviewer #3: Yes

Reviewer #4: Yes

Dear Editorial Team,

**PONE-D-25-41865; Clinical and cost-effectiveness of the digital intervention, MyWay Diabetes, in people with type 2 diabetes living in Greater Manchester during the COVID-19 pandemic.**

We are very grateful that we have been provided with another opportunity to refine and improve our manuscript. We have provided point-by-point responses below to each comment from the reviewers.

We trust that the manuscript is now suitable for publication in your journal and we will look forward to hearing from you.

Your sincerely,

**Rathi Ravindrarajah, PhD**

On behalf of the authors

**……………………………..**

5. Review Comments to the Author

Please use the space provided to explain your answers to the questions above. You may also include additional comments for the author, including concerns about dual publication, research ethics, or publication ethics. (Please upload your review as an attachment if it exceeds 20,000 characters)

**Reviewer #1:** The paper by Ravindrarajah and colleagues aimed to explore the use of digital intervention, MyWay Diabetes (MDW) in improving glycemic control and in reducing cardiovascular risk in individuals with type 2 diabetes during the COVID-19 pandemic in Greater Manchester. It also aims to establish the cost-effectiveness of this digital intervention in view of expanding its use in larger number of individuals. The Authors demonstrated that over 2 years follow up there was a reduction of 3.1 mmol/mol of HbA1c in patients using MDW as compared to non-users. Also, there was an improve in blood pressure and cholesterol level. In terms of costs, there was a reduction in patients using MDW as compared to non-users. The study is well conducted with rigorous methodology. The statistical analysis included major cofactors and covariates to ensure the best approach. The novelty is mainly represented by the use of MDW. For the first time also the economic burden of developing type 2 diabetes and its link with the development of complications is considered and analyzed. As stated in the limitations of the study, the length of follow-up of 2 years may have limited the results, it is advisable to prolong it and check whether a higher reduction in HbA1c and in blood pressure may be obtained although this may have a more negative impact on the costs.

Minor points to be addressed and/or commented:

**Comment 1.1:** Number of non-users vs. users: this is quite different. Have the Authors taken any measure to mitigate its impact?

**Response 1.1: Thank you for your comments. We studied a higher number of non-users by design to maximise statistical power to show differences between people using the intervention and people not using it; we matched 1 user to up to 20 non-users. To mitigate any effect of an imbalance in the simple size, we matched users to non-users for age (within one year) and sex, and our analysis adjusted for several covariates that could potentially confound between-group differences in HbA1c, blood pressure and lipids.**

**Comment 1.2:** Do the authors think that the use of certain medications may have impacted the results regardless of the use of MDW? Did they take this into account in their multivariable logistic regression?

**Response 1.2: Overall, MWD users did not increase their diabetes medication prescriptions, while controls did. As we have explained in the discussion, this suggests that the intervention may have helped prevent the escalation of prescribing in MWD users, which was observed in the control group. In other words, MWD users required less medication as their condition naturally deteriorated over time, perhaps because MWD led to improvements in diet or physical activity driven by increased awareness of these key lifestyle factors.**

**However, when we conducted extensive supplementary analyses on specific intervention-related changes in diabetes, blood pressure and lipid lowering medication, and we found no clinically significant changes in the prescribing of specific drug classes, when comparing MWD users and non-users (Supplementary e2.2 and tables e2.06-2.08). Since, there were no specific medication classes that appeared to mediate intervention-related between-group differences in HbA1c, blood pressure, and cholesterol, there was no case for including specific drug classes in the multivariable-adjusted analysis, in a mediation framework.**

**Comment 1.3**: Were patients monitored for glucose levels with sensors? This may have impacted in improving results of the use of MDW. The Authors may add a comment.

**Response 1.3: The purpose of our study was to evaluate the digital intervention MWD, in the setting of usual care and therefore we did not supplement the intervention with continuous glucose monitoring. A small number of participants in both MWD and non-MWD groups may have been using continuous glucose monitoring as part of the clinical care; our study made no attempt to change this routine care.**

Reviewer #2: The authors performed an interesting study. I have the following comments:

**Comment 2.1:** The authors may want to highlight more on the importance of digital intervention – 1. During COVID where face-to-face consultations are not feasible and 2. Even after COVID pandemic, patients esp the older population face mobility issues.

**Response 2.1: We are grateful to the reviewer for this suggestion. In response, we have added the following text in the Introduction (Page 3: lines 94-96), “Digital solutions offer clear benefits over usual care when face-to-face care is limited, such as during the COVID-19 pandemic, and in people with limited mobility*.”***

**Comment 2.2:** What are Changing Health, Oviva and MyCognition interventions?

**Response 2.2: Oviva and Changing Health both provide structured, personalized 12-week behaviour-change programmes delivered via apps and remote coaching designed to improve glucose control and body weight. MyCognition focuses on enhancing cognitive performance and mental resilience through personalised digital training, with studies indicating improvements in cognition that may support better diabetes self-management.**

**As we explained in the text of the introduction, we initially planned to evaluate these digital interventions that were also offered to MWD users, but since uptake was very low, we presented results only for MWD (lines 108-109, page 3). We explained in the results section that among MWD registrants, only 15 (3%) also used MyCognition, 7 (1.4%) also used Changing Health, 0 (0%) also used Oviva (lines 224-226, page 8). Excluding participants who also used Changing Health and MyCognition interventions had no effect on main study outcomes (Supplementary Table e2.03).**

**Comment 2.3:** Why were only age and sex chosen as covariates for matching? The participants should be matched based on A1c, other blood results, ethnicity…etc as well

**Response 2.3: Although we can understand the reviewer’s concerns, our approach is established practice for such analyses i.e., matching on key demographic variables and then controlling for remaining covariates of interest in a regression model. In a simulation study we have shown that this approach works well if cohort size is reasonably high,^1^ as it is in the current study. Matching on many variables can reduce the control pool so much that matching becomes impossible.**

**Comment 2.4:** Further to the comment above, while the authors argue that multivariate adjustment has been done in regression, the authors should still match the participants based on these important covariates then further adjust in the multivariate regression, known as ‘doubly-robust’ adjustment, which has been shown to further eliminate residual confounding. E.g. [https://www.thelancet.com/journals/lanwpc/article/PIIS2666-6065(22)00032-3/fulltext [thelancet.com]](https://urldefense.com/v3/__https:/www.thelancet.com/journals/lanwpc/article/PIIS2666-6065(22)00032-3/fulltext__;!!PDiH4ENfjr2_Jw!C1K40HAWArCysU31bhiuPj6NtplKVA_yhE9ok2V64BTdZh1vohyVGmZYfoODzJeXFF7AOhEn2glA37Z_NOr9BbheFaJtOWU$)

**Response 2.4: We agree with the use of the “doubly robust” statistical method highlighted by the reviewer. In all analyses, we have adjusting for the covariates we matched on; age and sex in this case, in line with best practice. However, as we explained in our response to the point above, the exact matching approach needs to be able to procure enough controls in the first place. In our study, matching on more variables would have reduced the number of controls and reduced the statistical power of our analysis.**

**An alternative methodology is propensity score matching, but, as we explained in our response above, our simulation study^1^ has shown that the approach that we adopted in our analysis is as good, if not better, when sample size is reasonably large as it was in this study. Propensity score matching approaches were developed with small samples in mind.**

**Comment 2.5:** Why did the authors choose to select a ratio as high as 1:20 for matching? For other studies e.g. propensity score matching it is usually as high as 1:5 only. By choosing a lower ratio, imputation may not even need to be done as you can select patients with complete set of data

**Response 2.5: Thank you for your comment. The 1:20 matching ratio was chosen to maximise statistical power and improve the precision of our estimates by making full use of available data in our non-user controls.**

**We agree that exact or propensity score matching studies often use lower ratios (e.g., up to 1:5). This tends to be the case because there may be smaller pools of controls available in many studies and/or because the relationship between control numbers and power is log-linear and therefore benefits are small as numbers increase - especially with more than 10 controls. Nevertheless, because we had a large control pool, we aimed to maximise power by matching up to 1:20.**

**Selecting only patients with complete data would arguably be problematic because of recording bias. In primary care research, patients with poorer health tend to have more complete data because they visit their GP surgery more often. These controls with complete data could be systematically different to the general cohort of people with type 2 diabetes. Our aim was to generate generalisable data that would inform about the potential impact of the MWD intervention when made available to all people with type 2 diabetes in primary care, and so we aimed to be as inclusive as possible for both MWD users and non-user controls.**

**Comment 2.6:** Any information on medication compliance?

**Response 2.6: We had information on medication prescribing, but we did not have access to any information on whether medication was dispensed or taken by the patient. Assessing medication compliance in UK primary care data is difficult because there are no routinely available data to indicate if medicines are actually taken.**

**However, as previously stated above, our results suggest that the positive effects of MWD intervention are not mediated by higher medication use; the opposite seems to be true. My Way Diabetes users tend to experience less of an increase in prescribing (and by inference, medication use) compared to no users during the study.**

**Reviewer #3:** This manuscript presents a well-conducted and clearly written evaluation of the MyWay Diabetes (MDW) digital self-management intervention among people with type 2 diabetes in Greater Manchester during the COVID-19 pandemic. The topic is timely and important, addressing how large-scale digital tools can support diabetes care and long-term cost-effectiveness.

The study design is appropriate, the analytical approach is rigorous, and the conclusions are well supported by the data. The manuscript reads smoothly and maintains a professional, balanced tone. I appreciate the authors’ careful handling of real-world data and their transparent discussion of limitations.

That said, a few revisions would improve clarity, transparency, and presentation.

Major comments

**Comment 3.1:** Participant selection and engagement
The possibility of self-selection bias is acknowledged but not further explored. Including simple engagement metrics—such as login frequency, module completion rate, or duration of use—would help readers understand whether the observed effects reflect platform engagement or user motivation. If such data are unavailable, please note this explicitly and discuss the limitation.

**Response 3.1: We don’t have access to these data, and we referred to this in the discussion but recognise that our text could have been clearer. In response to this helpful comment, we have modified the following text in the limitations section of the discussion which previously read, “data on participant engagement was not collected, limiting our ability to assess sustained use and its potential influence on effectiveness”. It now reads, “data on MWD usage/participant engagement was not collected, limiting our ability to assess the impact of motivation and sustained use on effectiveness”.**

**Comment 3.2:** Matching and baseline comparability.

While age- and sex-matching is reasonable, some differences remain in ethnicity and the proportion of participants with HbA1c >53 mmol/mol. Reporting standardized mean differences or presenting a balance table would improve transparency and reader confidence in group comparability.

**Response**
**Thank you. These data provided in tables e2.03-e2.05 in the supplementary document.**

**Table e2.03 shows multivariable-adjusted mean differences in post intervention primary and secondary outcomes between MWD users and matched controls not using MWD, after excluding participants who also used Changing Health and MyCognition interventions.**

**Table e2.04 shows multivariable-adjusted mean differences in primary and secondary outcomes between MWD users and matched controls not using MWD, using paired available outcome data.**

**Table e2.05 shows within-group changes in outcomes for test bed participants and matched controls who did not take up the intervention (pre and post-intervention levels are shown).**

**We have focused on the outcome variables because they are of greatest importance. Please note that we adjust all analyses for any differences in baseline levels of these outcomes, and other covariates including ethnicity, in the multivariable-adjusted analyses.**

**In a simulation study,^1^ we have shown that controlling for these covariates in a regression modelling framework is as good if not better than propensity score matching approaches when samples are large enough, as they are in our study.**

**Comment 3.3:** Handling of missing data
Multiple imputation is appropriate, but the manuscript would benefit from a short summary of missingness rates and patterns for key variables in the main text, not only in the Supplement. Please also confirm that the assumption of data being “missing at random” is justified.

**Response 3.3: Thank you. We have now added a short summary of missingness and patterns for key variables, lines 241-249, page 8-9.**

***“Across outcomes, the proportion of missing data was consistently higher at the post-intervention timepoint than at baseline for both DMW users and controls. For the primary outcome (HbA1c), missingness increased from 3.9% to 21.9% among DMW users and from 13.1% to 31.2% among controls. Similar patterns were observed for secondary outcomes: systolic and diastolic blood pressure missingness rose from 8.7% to 21.5% in users and from 16.1% to 33.4% in controls; total cholesterol missingness increased from 8.1% to 28.2% in users and from 17.5% to 36.8% in controls; and body weight missingness rose from 10.8% to 30.0% in users and from 20.8% to 41.5% in controls. Exploratory outcomes also showed substantial missingness, with post-intervention missing data ranging from 22.1% to 65.3% among users and 31.7% to 74.9% among controls. “***

**Regarding the MAR assumption, we cannot evaluate whether data are missing at random (MAR) or missing not at random (MNAR). However, it is known that multiple imputation is preferrable to a complete case analysis not only when data are missing completely at random or MCAR (increasing power) or MAR (reducing/minimising bias), but even when data are MNAR, as we have shown in a published simulation study.^2^**

**It is important to note that we performed sensitivity analyses using complete-case data to assess the robustness of the findings using multiple imputation, with agreement in the results across the two approaches.**

**Comment 3.4**: Medication use and costs
The finding of a smaller increase in medication costs among MDW users is interesting but may also reflect prescribing behaviour or medication adherence rather than a direct effect of the intervention. This should be acknowledged more explicitly in the Discussion.

**Response 3.4: In response to this helpful comment, we have added some text in lines 376-379, pages 13-14*, “While the smaller change in medication costs observed among DMW users is encouraging, it may reflect differences in prescribing behaviour or medication adherence rather than a direct effect of the intervention, and this potential influence should be considered when interpreting the findings.”***

**Comment 3.5:** Economic modelling transparency
The economic evaluation using the UKPDS Outcomes Model (v2.1) is methodologically appropriate and consistent with NICE guidance. However, the absence of a probabilistic sensitivity analysis limits uncertainty assessment. Please highlight this limitation more clearly, and consider adding a concise table or appendix summarising key input parameters for reproducibility.

**Response 3.5: Thank you for your comments. In response, we have added text to lines 316-317, page 11, highlighting that the absence of a** **probabilistic sensitivity analysis limits the assessment of parameter uncertainty. The text reads, “*the absence of a probabilistic sensitivity analysis (this was not computationally feasible), which limits assessment of parameter uncertainty*”**

**We have also addressed your suggestion of highlighting the key inputs by adding text to lines 180-182, page 6, which directs the reader to the appropriate appended tables. This reads, “*Supplemental Material e3 provides comprehensive methods, with Tables e3.01–4.05 highlighting key model parameters and patient characteristics.”***

Minor comments

**Comment 3.6:** A simple flow diagram outlining inclusion, exclusion, and matching would enhance clarity.

**Response 3.6: We originally provided flow diagrams in supplementary text (figures e2.01-e2.04; pages 6-7). We will be happy to move these to the main paper, if the reviewer and editorial team feel this would be helpful.**

**Comment 3.7:** Figure 1 (cost-effectiveness analysis): please refine the legend to explain all line types clearly (e.g., horizontal dashed = current uptake; vertical dashed = full GM licence cost; dotted = discounted cost scenarios). Ensure both axes are labelled with units.

**Response 3.7: Thank you for this helpful comment. In response we have modified the legend to this figure so that it now reads as shown below:**

**Horizontal dashed: current uptake in Greater Manchester (16,194 of 182,025 people with type 2 diabetes = 8·9%)**

**Vertical dashed: full GMDMW cost (licence fee = £313,557 per year; eligible population in Greater Manchester = 182,025; £1·72 per person per year)**

**Vertical dotted (staggered): full GMDMW cost given the current commercial arrangement (initial 50% discount; rising to full price once uptake reaches 20%; equal steps for every 5 percentage-points of uptake in between)**

**As suggested, have ensured that both axis are labelled with units.**

**Comment 3.8:** Table 1: consider rounding p-values to two decimal places and aligning numerical values for readability. Add short notes for abbreviations (e.g., “SD = standard deviation; IMD = Index of Multiple Deprivation”).

**Response 3.8: Thank you for these helpful suggestions. We have rounded the p-values to 2 decimal places, aligned numerical values for readability and added short notes for the abbreviations as suggested (lines 569-574, page 24).**

**Comment 3.9:** Table 2: add a brief note in the legend stating that models were adjusted for baseline risk factors and time between measurements, so the table is interpretable independently from the text.

**Response 3.9: In response to this helpful comment, we have added a legend below this table (lines 563-565, page 21). The text reads,**

***“All models were adjusted for age, sex, ethnicity, deprivation, baseline risk factors (HbA1c, systolic blood pressure, diastolic blood pressure, total, HDL and LDL cholesterol, triglycerides, UACR, smoking and body weight) and time from pre-intervention outcome assessment to post intervention outcome assessment.”***

**Comment 3.10:** Supplementary figures (e4.01–e4.05): line styles and colours for best- and worst-case scenarios are difficult to distinguish; use distinct line types or include an in-figure legend. Ensure all axes include units (e.g., “probability,” “cost £”).

**Response 3.10: In response to this helpful comment, we have changed “best case scenario” lines to dotted style in supplementary figures e4.01-e4.05. This makes it easier to distinguish. We have made sure that all axes have appropriate units e.g., weight (kg).**

**Comment 3.11:** Referencing in the Supplement: indicate that citations correspond to those in the main manuscript to avoid confusion during typesetting.

**Response 3.11: Thank you for this comment. We would be grateful if the editorial team could advise us on whether it would be helpful to combine the reference list from the main manuscript and the supplement (currently they are separate). We would also welcome guidance on whether we need to change the single reference provided at the base of supplementary table e5.01 (page 68).**

**Comment 3.12:** Summary of supplement: the supplementary material is comprehensive and strengthens the manuscript; still, briefly summarising key supplementary results (missing data, sensitivity assumptions) in the main text would make the paper more self-contained.

**Response 3.12: A missing data handling summary has now been added; lines 240-248, page 8. This limited word count restricts us in providing a more comprehensive summary of the results in the main manuscript, currently in the supplementary tables. We would welcome guidance from the editorial team about extending the word limit if it was felt that additional information in the manuscript would be sufficiently useful.**

**Comment 3.13:** Maintain tense consistency (prefer past tense in Methods).

**Response 3.13: We have modified the text in response to this helpful suggestion.**

**Comment 3.14:** The paragraph on speculative behavioural mechanisms could be shortened or moved to the supplement for better flow.

**Response 3.14: In response to this helpful comment, we have shortened the paragraph on mechanisms which now reads, “*Although our study was not designed to investigate mechanisms behind MWD’s impact on risk factors, improvements in diet or physical activity, driven by increased awareness of key lifestyle factors, could have played a role. Notably, MWD users did not increase their diabetes medication prescriptions (while controls did), and there were no intervention-related changes in body weight, suggesting that these factors were not mediating factors.”* (362-366, page 13)**

**Comment 3.15:** Highlight early in the Introduction that MDW directly integrates with NHS primary-care data, which distinguishes it from other digital interventions.

**Response 3.15: Thank you. In response, we have added the following text to the introduction (lines112-114): *“Unlike other digital interventions, the MWD platform directly integrates with NHS primary-care data, enabling seamless access to patient records and more personalised data-driven support.”***

**Comment 3.16:** Ethics and data availability
Ethical approval and data governance are clearly described (Northwest GM South REC 261268). The data-availability restriction under the UK Data Protection Act is understandable; however, if feasible, providing access to analytic code or a synthetic dataset would further enhance transparency and reproducibility.

**Response 3.16: Thank you for this helpful suggestion. We do not have access to synthetic data but we will provide links to code in the supplementary material.**

**Comment 3.16:** Language and presentation
The manuscript is written in fluent and professional English. The overall presentation is clear and polished. Minor stylistic edits may improve rhythm and consistency, but no substantive language issues were found. Figures and tables are generally well prepared and consistent with the results; the minor refinements above would make them publication-ready.

This is a high-quality and methodologically sound study that meets PLOS ONE standards for scientific validity. The paper makes a valuable contribution to the evidence base for digital diabetes management. Addressing the suggested clarifications—particularly regarding engagement data, baseline balance, and presentation details—will further strengthen an already excellent manuscript.

**Response 3.17: Thank you very much for these positive supportive comments.**

**Reviewer #4:** This quasi-experimental study evaluated the clinical and economic impact of the MyWay Diabetes (MDW) digital self-management platform among adults with type 2 diabetes (T2D) in Greater Manchester during the COVID-19 pandemic. The study suggested that MyWay Diabetes offers a scalable, low-cost digital tool that enhances diabetes self-management and modestly improves key cardiometabolic risk factors. The integration of MyWay Diabetes into primary care systems was considered cost effective by reducing long-term complication risks.

**Comment 4.1:** The major concern for this study is the non-randomised design, which warrants proper statement as a limitation.

**Response 4.1: Thank you for highlighting this important point. The limitation section of the discussion starts with the statement, “Our study limitations are: a) the observational design, rather than an RCT, limits causal inference…”.**

**When compared to a randomised controlled trial design, the adopted study design was chosen for cost, ethical and logistical reasons. Our analytical approach aimed to minimise confounding through matching and regression modelling.**

**Comment 4.2:** The presentation of this study needs improvement including figures and tables. For example, in Table 1, it is unclear what N available and data mean. For outcomes, it is important to report the changes in each group before the difference between arms.

**Response 4.2: Thank you for this helpful comment. We have reviewed all tables and figure in the manuscript and the extensive supplemental file; and modified the data display when appropriate. A footnote to Table 1 provides additional explanation for the metrics presented there. We will be happy to make further changes as recommended by the reviewer.**

**Comment 4.3:** The cost-effectiveness model is not quite clear and a bubble plot is necessary in the main text.

**Response 4.3: It would be helpful if we could be provided with further clarification of the reviewer’s request here. If the reviewer is referring to a bubble plot describing a probabilistic sensitivity analysis (PSA), then we regret that a PSA was not computationally feasible in our data, as highlighted in the limitations section of the discussion.**

**Comment 4.4:** It is relevant to apply more risk calculators such as Qrisk3 for risk assessment.

**Response 4.4: Thank you for this comment. We would like to highlight that our model included several covariates relevant to T2DM management, most if not all of which are included in many risk prediction models, including QRISK. We do not see how inclusion of a cardiovascular disease risk prediction model would usefully add to the covariates already included in our model. However, if the reviewer could kindly provide justification for this request and we would be very pleased to consider this further.**

6. PLOS authors have the option to publish the peer review history of their article ([what does this mean? [track.editorialmanager.com]](https://urldefense.com/v3/__https:/track.editorialmanager.com/CL0/https:*2F*2Fjournals.plos.org*2Fplosone*2Fs*2Feditorial-and-peer-review-process*23loc-peer-review-history/1/010f019a2dff0555-7f7ccaec-b8e1-488a-a889-062d52f9f64a-000000/eBiEmNBev-pzhRnj4OEtTxoP6RTDLLjopyq501RHBgA=235__;JSUlJSUl!!PDiH4ENfjr2_Jw!C1K40HAWArCysU31bhiuPj6NtplKVA_yhE9ok2V64BTdZh1vohyVGmZYfoODzJeXFF7AOhEn2glA37Z_NOr9BbheQKT8xJk$)). If published, this will include your full peer review and any attached files.

If you choose “no”, your identity will remain anonymous but your review may still be made public.

**Do you want your identity to be public for this peer review?** For information about this choice, including consent withdrawal, please see our [Privacy Policy [track.editorialmanager.com]](https://urldefense.com/v3/__https:/track.editorialmanager.com/CL0/https:*2F*2Fwww.plos.org*2Fprivacy-policy/1/010f019a2dff0555-7f7ccaec-b8e1-488a-a889-062d52f9f64a-000000/fWFjRw3Xy89knMUGOtOhX3MgY4nFVfWFt2pAeSqz_Cs=235__;JSUl!!PDiH4ENfjr2_Jw!C1K40HAWArCysU31bhiuPj6NtplKVA_yhE9ok2V64BTdZh1vohyVGmZYfoODzJeXFF7AOhEn2glA37Z_NOr9Bbhevq_woR4$).

Reviewer #1: No

Reviewer #2: No

Reviewer #3: No

Reviewer #4: No

[NOTE: If reviewer comments were submitted as an attachment file, they will be attached to this email and accessible via the submission site. Please log into your account, locate the manuscript record, and check for the action link "View Attachments". If this link does not appear, there are no attachment files.]

To ensure your figures meet our technical requirements, please review our figure guidelines: [https://journals.plos.org/plosone/s/figures [journals.plos.org]](https://urldefense.com/v3/__https:/journals.plos.org/plosone/s/figures__;!!PDiH4ENfjr2_Jw!C1K40HAWArCysU31bhiuPj6NtplKVA_yhE9ok2V64BTdZh1vohyVGmZYfoODzJeXFF7AOhEn2glA37Z_NOr9Bbhel2I5ZZI$)

You may also use PLOS’s free figure tool, NAAS, to help you prepare publication quality figures: [https://journals.plos.org/plosone/s/figures#loc-tools-for-figure-preparation [journals.plos.org]](https://urldefense.com/v3/__https:/journals.plos.org/plosone/s/figures*loc-tools-for-figure-preparation__;Iw!!PDiH4ENfjr2_Jw!C1K40HAWArCysU31bhiuPj6NtplKVA_yhE9ok2V64BTdZh1vohyVGmZYfoODzJeXFF7AOhEn2glA37Z_NOr9BbhebOEAlsg$).

NAAS will assess whether your figures meet our technical requirements by comparing each figure against our figure specifications.

**References**

1.	Wilkinson JD, Mamas MA, Kontopantelis E. Logistic regression frequently outperformed propensity score methods, especially for large datasets: a simulation study. *J Clin Epidemiol*. 2022;152:176-184. doi: 10.1016/j.jclinepi.2022.09.009

2.	Kontopantelis E, White IR, Sperrin M, Buchan I. Outcome-sensitive multiple imputation: a simulation study. *BMC medical research methodology*. 2017;17:2. doi: 10.1186/s12874-016-0281-5
