# Supplementary material for: Clinical and cost-effectiveness of the digital intervention, MyWay Diabetes, in people with type 2 diabetes living in Greater Manchester during the COVID-19 pandemic
Source: PLoS One. 2026 Jun 25;21(6):e0349232. doi: 10.1371/journal.pone.0349232 (PMC13298753; doi:10.1371/journal.pone.0349232)
Supplement: Attachment — Submitted filename: Response_to_Reviewers_auresp_2.docx [file pone.0349232.s005.docx]

Dear Editorial Team,

**PONE-D-25-41865; Clinical and cost-effectiveness of the digital intervention, MyWay Diabetes, in people with type 2 diabetes living in Greater Manchester during the COVID-19 pandemic.**

We are very grateful that we have been provided with an opportunity to further improve our manuscript. Here we provide a point-by-point response to each comment from the reviewers.

We trust that the manuscript is now suitable for publication in your journal and we look forward to hearing from you.

Your sincerely,

**Rathi Ravindrarajah, PhD**

On behalf of the authors

……………………………………………………………………………………………………………………………………………

Reviewer #2: The authors submitted a revision of the manuscript. I have the following comments:

Comment 1. The authors calculated the sample size with 1:10 ratio. Please present in sensitivity analysis the findings if PS matching 1:10 is used, ideally these patients should be those with complete data.

**Response 1: Thank you for this comment. However, we are not entirely certain that we have interpreted the suggestion correctly as the wording of the comment is somewhat unclear. We understand that the reviewer may be suggesting conducting a sensitivity analysis using PS matching with a 1:10 ratio.**

**We have already conducted numerous sensitivity analyses to assess the robustness of our findings. In our view, the proposed sensitivity analysis would not provide useful additional insights beyond the approaches already implemented, nor is it justified in the context of our study design. We have therefore not added the analysis.**

**Our rationale is explained in more detail below (please see our response to point 3 below), where we discuss the methodological considerations relevant to the suggestion.**

Comment 2. What is meant by "age (within 1 year)"? Age should be fined according to that upon enrolment

**Response 2: We thank the reviewer for this comment. "Age within 1 year" refers to the matching criterion applied during control selection, whereby each case (MWD user) was matched to controls whose age differed by no more than one year. Age was defined at the “index date”, which in such observational studies represents the time point at which follow-up begins. In our study, the index date corresponds to the date of first use the intervention for cases (MWD users); matched controls inherit the index date from their matched MWD user.**

**In the dataset used only birthyear was available to preserve participant anonymity. As a result, a small degree of variation in exact age will occur. In response to this helpful comment, we have revised the manuscript to clarify the definition of age and the matching procedure (lines 146-148, page 5).**

**This text reads, ‘*Matching for birth year (within 1 year) and sex was performed, with each MWD user matched to up to 20 controls not using MWD to increase statistical power. Age and sex were defined at the MWD start date for each user, which served as the index date for the corresponding matched control non-users*.’**

Comment 3. Despite the authors having extensively responded to the comments regarding adjusting for covariates in regression vs PS matching, putting the covariates in the propensity score is a more commonly accepted approach. Please perform this a sensitivity analysis, accepting a lower ratio e.g. 1:5, which is also commonly adopted in the current literature. The authors may also argue that increasing the number of covariates may reduce matching, but (a) it can be done with a lower ratio (b) at the authors quoted in their own previous simulation study, logistic regression only outperformed PS when sample size is large enough. The authors may also consider use of covariate-balancing PS (CBPS) with doubly-robust adjustment - please refer to the paper I cited in my previous comments.

**Response 3: We appreciate the opportunity to clarify our methodological choices. We respectfully maintain that the analytical approach used in our study remains appropriate for the research question and the data structure.**

**In observational studies, using EHR data, the approach we used - a combination of exact matching on key variables and covariate adjustment is a commonly accepted approach, and has become the norm. As noted previously, PS matching methods are primarily intended as a dimension reduction strategy, which is particularly useful when the number of covariates is large relative to the sample size.**

**In our study, however, we have a substantial sample size of close to 11,000 participants, and therefore direct covariant adjustment through regression modelling is both feasible and methodologically appropriate.**

**It is also well recognised that including a large number of covariates in the PS can sometimes lead to poorer matching on key covariates (the reviewer may have misunderstood that point, our argument was this, not that numbers to be matched would be reduced). This is the reason why best practice is to combine matching on the PS with adjustment for the included covariates, rather than relying solely on PS matching. When the sample size is large this effectively makes the matching process pointless.**

**The reviewer also refers to our previously cited simulation study. In that work, regression adjustment performed similarly or better than PS-based approaches when the sample size exceeded 1,000. Given that our sample includes nearly 11,000 individuals, this supports the suitability of the regression-based approach that we used.**

**Regarding the suggestion to use a lower matching ratio (e.g., 1:5), our concern is that this will reduce the statistical power of the study without addressing a clear methodological concern. Lower ratios are often used in other studies where there are not enough controls to match on, which is not the case in our data.**

**For these reasons, and given the multiple sensitivity analyses already performed, we believe that additional analyses proposed would be unlikely to materially alter the key findings in our manuscript.**
