# Supplementary material for: Clinical and cost-effectiveness of the digital intervention, MyWay Diabetes, in people with type 2 diabetes living in Greater Manchester during the COVID-19 pandemic
Source: PLoS One. 2026 Jun 25;21(6):e0349232. doi: 10.1371/journal.pone.0349232 (PMC13298753; doi:10.1371/journal.pone.0349232)
Supplement: Attachment — Submitted filename: Response to Reviewers_200426.docx [file pone.0349232.s006.docx]

Dear Editorial Team,

**PONE-D-25-41865; Clinical and cost-effectiveness of the digital intervention, MyWay Diabetes, in people with type 2 diabetes living in Greater Manchester during the COVID-19 pandemic.**

We are very grateful that we have been provided with an opportunity to further improve our manuscript. Here we provide a point-by-point response to each comment from the reviewers.

We trust that the manuscript is now suitable for publication in your journal and we look forward to hearing from you.

Your sincerely,

**Rathi Ravindrarajah, PhD**

On behalf of the authors

……………………………………………………………………………………………………………………………………………

Reviewer #3: Final version

I thank the authors for their thorough and thoughtful responses to my previous comments. The majority of revisions have been addressed satisfactorily, and the manuscript has improved considerably. However, two points require further clarification before I can recommend acceptance.

Remaining concerns

1. Baseline comparability (Major comment 3.2)

I appreciate the authors' response, but I note that the tables referenced (e2.03–e2.05) present outcome data rather than baseline covariate balance. My original request was for standardized mean differences (SMDs) or a balance table showing comparability between MWD users and controls on key baseline characteristics — particularly ethnicity and the proportion with HbA1c >53 mmol/mol, where differences remain visible in Table 1.

Reporting SMDs for baseline covariates is standard practice in observational studies and would allow readers to assess residual imbalance independently of regression adjustment. I would suggest that the authors either: (a) add a column of SMDs to Table 1, or (b) provide a brief supplementary balance table. This is a reporting addition and does not require further analysis.

**Response 1: In response to this helpful comment, we have now added an extra column in Table 1 where the SMD values have been added. Pages 19-20**

2. Flow diagram (Minor comment 3.6)

The authors have offered to move the flow diagram to the main text if considered helpful. I believe this would improve clarity and would encourage its inclusion in the main manuscript rather than in the supplementary material. A participant flow diagram is standard for studies of this type and aids reader comprehension.

**Response 2: Thank you for your comments. In response we have now moved the flow diagrams from the appendix to the main text. [Pages 26-26]**
