# Supplementary material for: Clinical and cost-effectiveness of the digital intervention, MyWay Diabetes, in people with type 2 diabetes living in Greater Manchester during the COVID-19 pandemic
Source: PLoS One. 2026 Jun 25;21(6):e0349232. doi: 10.1371/journal.pone.0349232 (PMC13298753; doi:10.1371/journal.pone.0349232)
Supplement: Peer review history

# Peer review: Decision Letter 0

**DOI:** 10.1371/journal.pone.0349232.r001

**Authors:** Ennio Polilli

28 Oct 2025

PONE-D-25-41865

Clinical and cost-effectiveness of the digital intervention, MyWay Diabetes, in people with type 2 diabetes living in Greater Manchester during the COVID-19 pandemic.

PLOS ONE

Dear Dr.  Ravindrarajah,

Thank you for submitting your manuscript to PLOS ONE. After careful consideration, we feel that it has merit but does not fully meet PLOS ONE’s publication criteria as it currently stands. Therefore, we invite you to submit a revised version of the manuscript that addresses the points raised during the review process.

Please submit your revised manuscript by Dec 12 2025 11:59PM. If you will need more time than this to complete your revisions, please reply to this message or contact the journal office at plosone@plos.org. When you're ready to submit your revision, log on to https://www.editorialmanager.com/pone/ and select the 'Submissions Needing Revision' folder to locate your manuscript file.

Please include the following items when submitting your revised manuscript:

- A rebuttal letter that responds to each point raised by the academic editor and reviewer(s). You should upload this letter as a separate file labeled 'Response to Reviewers'.
- A marked-up copy of your manuscript that highlights changes made to the original version. You should upload this as a separate file labeled 'Revised Manuscript with Track Changes'.
- An unmarked version of your revised paper without tracked changes. You should upload this as a separate file labeled 'Manuscript'.

If you would like to make changes to your financial disclosure, please include your updated statement in your cover letter. Guidelines for resubmitting your figure files are available below the reviewer comments at the end of this letter.

If applicable, we recommend that you deposit your laboratory protocols in protocols.io to enhance the reproducibility of your results. Protocols.io assigns your protocol its own identifier (DOI) so that it can be cited independently in the future. For instructions see: https://journals.plos.org/plosone/s/submission-guidelines#loc-laboratory-protocols. Additionally, PLOS ONE offers an option for publishing peer-reviewed Lab Protocol articles, which describe protocols hosted on protocols.io. Read more information on sharing protocols at https://plos.org/protocols?utm_medium=editorial-email&utm_source=authorletters&utm_campaign=protocols.

We look forward to receiving your revised manuscript.

Kind regards,

Ennio Polilli

Academic Editor

PLOS ONE

Journal Requirements:

When submitting your revision, we need you to address these additional requirements.

1. Please ensure that your manuscript meets PLOS ONE's style requirements, including those for file naming. The PLOS ONE style templates can be found at

https://journals.plos.org/plosone/s/file?id=wjVg/PLOSOne_formatting_sample_main_body.pdf and

https://journals.plos.org/plosone/s/file?id=ba62/PLOSOne_formatting_sample_title_authors_affiliations.pdf

2. Please provide additional details regarding participant consent. In the ethics statement in the Methods and online submission information, please ensure that you have specified what type you obtained (for instance, written or verbal, and if verbal, how it was documented and witnessed). If your study included minors, state whether you obtained consent from parents or guardians. If the need for consent was waived by the ethics committee, please include this information.

Once you have amended this/these statement(s) in the Methods section of the manuscript, please add the same text to the “Ethics Statement” field of the submission form (via “Edit Submission”).

For additional information about PLOS ONE ethical requirements for human subjects research, please refer to http://journals.plos.org/plosone/s/submission-guidelines#loc-human-subjects-research.

3. Thank you for stating the following financial disclosure:

“Innovate UK and NHS England (ref: 104658)”

Please state what role the funders took in the study.  If the funders had no role, please state: "The funders had no role in study design, data collection and analysis, decision to publish, or preparation of the manuscript."

If this statement is not correct you must amend it as needed.

Please include this amended Role of Funder statement in your cover letter; we will change the online submission form on your behalf.

4. Please note that funding information should not appear in any section or other areas of your manuscript. We will only publish funding information present in the Funding Statement section of the online submission form. Please remove any funding-related text from the manuscript.

5. Thank you for stating the following in the Competing Interests section:

“Doogie Brodie is development manager at MyWay Digital Health. Deborah J Wake is a co-founder and shareholder of MyWay Digital Health (MWDH). Martin K Rutter reports consulting fees from Eli Lilly and Brigham and Women’s Hospital and modest stock ownership in GSK, all unrelated to this work. Michael Morgan-Curran is CEO at Asclepius MedTech Limited, unrelated to this work. Scott G Cunningham is co-founder, director and CTO at MyWay Digital Health and reports grants from Scottish Government, outside the submitted work; personal fees, non-financial support and other from MyWay Digital Health. Naresh Kanumilli reports fees for lectures and advisory boards for BI, Lily, AZ, Novartis, Sanofi Manarini, Novo, Abbott, unrelated to this work. George Tilston is funded by the National Institute for Health and Care Research (NIHR) Manchester Biomedical Research Centre (BRC) (NIHR203308)

All other authors report no conflict of interests. All authors have completed the conflict-of-interest disclosure forms.

In accordance with Innovate UK’s funding agreement, the University of Manchester team conducted data analysis and manuscript writing independently of MyWay Digital Health. Both teams collaborated on study design and the Innovate UK funding application, and the MyWay Digital Health team provided feedback on the final manuscript draft; however, they did not influence the study’s results or conclusions.”

We note that one or more of the authors are employed by a commercial company: MyWay Digital Health (MWDH)

1. Please provide an amended Funding Statement declaring this commercial affiliation, as well as a statement regarding the Role of Funders in your study. If the funding organization did not play a role in the study design, data collection and analysis, decision to publish, or preparation of the manuscript and only provided financial support in the form of authors' salaries and/or research materials, please review your statements relating to the author contributions, and ensure you have specifically and accurately indicated the role(s) that these authors had in your study. You can update author roles in the Author Contributions section of the online submission form.

Please also include the following statement within your amended Funding Statement.

“The funder provided support in the form of salaries for authors [insert relevant initials], but did not have any additional role in the study design, data collection and analysis, decision to publish, or preparation of the manuscript. The specific roles of these authors are articulated in the ‘author contributions’ section.”

If your commercial affiliation did play a role in your study, please state and explain this role within your updated Funding Statement.

2. Please also provide an updated Competing Interests Statement declaring this commercial affiliation along with any other relevant declarations relating to employment, consultancy, patents, products in development, or marketed products, etc.

Within your Competing Interests Statement, please confirm that this commercial affiliation does not alter your adherence to all PLOS ONE policies on sharing data and materials by including the following statement: "This does not alter our adherence to  PLOS ONE policies on sharing data and materials.” (as detailed online in our guide for authors http://journals.plos.org/plosone/s/competing-interests) . If this adherence statement is not accurate and  there are restrictions on sharing of data and/or materials, please state these. Please note that we cannot proceed with consideration of your article until this information has been declared.

Please include both an updated Funding Statement and Competing Interests Statement in your cover letter. We will change the online submission form on your behalf.

6. We note that you have indicated that there are restrictions to data sharing for this study. PLOS only allows data to be available upon request if there are legal or ethical restrictions on sharing data publicly. For more information on unacceptable data access restrictions, please see http://journals.plos.org/plosone/s/data-availability#loc-unacceptable-data-access-restrictions.

Before we proceed with your manuscript, please address the following prompts:

a) If there are ethical or legal restrictions on sharing a de-identified data set, please explain them in detail (e.g., data contain potentially identifying or sensitive patient information, data are owned by a third-party organization, etc.) and who has imposed them (e.g., a Research Ethics Committee or Institutional Review Board, etc.). Please also provide contact information for a data access committee, ethics committee, or other institutional body to which data requests may be sent.

b) If there are no restrictions, please upload the minimal anonymized data set necessary to replicate your study findings to a stable, public repository and provide us with the relevant URLs, DOIs, or accession numbers. For a list of recommended repositories, please see

https://journals.plos.org/plosone/s/recommended-repositories. You also have the option of uploading the data as Supporting Information files, but we would recommend depositing data directly to a data repository if possible.

We will update your Data Availability statement on your behalf to reflect the information you provide.

7. If the reviewer comments include a recommendation to cite specific previously published works, please review and evaluate these publications to determine whether they are relevant and should be cited. There is no requirement to cite these works unless the editor has indicated otherwise.

[Note: HTML markup is below. Please do not edit.]

Reviewer's Responses to Questions

**Comments to the Author**

1. Is the manuscript technically sound, and do the data support the conclusions?

The manuscript must describe a technically sound piece of scientific research with data that supports the conclusions. Experiments must have been conducted rigorously, with appropriate controls, replication, and sample sizes. The conclusions must be drawn appropriately based on the data presented.

Reviewer #1: Yes

Reviewer #2: Yes

Reviewer #3: Yes

Reviewer #4: Yes

**********

2. Has the statistical analysis been performed appropriately and rigorously?

Reviewer #1: Yes

Reviewer #2: No

Reviewer #3: Yes

Reviewer #4: Yes

**********

3. Have the authors made all data underlying the findings in their manuscript fully available?

The PLOS Data policy requires authors to make all data underlying the findings described in their manuscript fully available without restriction, with rare exception (please refer to the Data Availability Statement in the manuscript PDF file). The data should be provided as part of the manuscript or its supporting information, or deposited to a public repository. For example, in addition to summary statistics, the data points behind means, medians and variance measures should be available. If there are restrictions on publicly sharing data—e.g. participant privacy or use of data from a third party—those must be specified.

Reviewer #1: Yes

Reviewer #2: No

Reviewer #3: No

Reviewer #4: No

**********

4. Is the manuscript presented in an intelligible fashion and written in standard English?

PLOS ONE does not copyedit accepted manuscripts, so the language in submitted articles must be clear, correct, and unambiguous. Any typographical or grammatical errors should be corrected at revision, so please note any specific errors here.

Reviewer #1: Yes

Reviewer #2: Yes

Reviewer #3: Yes

Reviewer #4: Yes

**********

5. Review Comments to the Author

Please use the space provided to explain your answers to the questions above. You may also include additional comments for the author, including concerns about dual publication, research ethics, or publication ethics. (Please upload your review as an attachment if it exceeds 20,000 characters)

Reviewer #1: The paper by Ravindrarajah and colleagues aimed to explore the use of digital intervention, MyWay Diabetes (MDW) in improving glycemic control and in reducing cardiovascular risk in individuals with type 2 diabetes during the COVID-19 pandemic in Greater Manchester. It also aims to establish the cost-effectiveness of this digital intervention in view of expanding its use in larger number of individuals. The Authors demonstrated that over 2 years follow up there was a reduction of 3.1 mmol/mol of HbA1c in patients using MDW as compared to non-users. Also, there was an improve in blood pressure and cholesterol level. In terms of costs, there was a reduction in patients using MDW as compared to non-users. The study is well conducted with rigorous methodology. The statistical analysis included major cofactors and covariates to ensure the best approach. The novelty is mainly represented by the use of MDW. For the first time also the economic burden of developing type 2 diabetes and its link with the development of complications is considered and analyzed. As stated in the limitations of the study, the length of follow-up of 2 years may have limited the results, it is advisable to prolong it and check whether a higher reduction in HbA1c and in blood pressure may be obtained although this may have a more negative impact on the costs.

Minr points to be addressed and/or commented:

1) Number of non-users vs. users: this is quite different. Have the Authors taken any measure to mitigate its impact?

2) Do the Authors think that the use of certain medications may have impacted the results regardless of the use of MDW? Did they take this into account in their multivariable logistic regression?

3) Were patients monitored for glucose levels with sensors? This may have impacted in improving results of the use of MDW. The Authors may add a comment.

Reviewer #2: The authors performed an interesting study. I have the following comments:

1. The authors may want to highlight more on the importance of digital intervention – 1. During COVID where face-to-face consultations are not feasible and 2. Even after COVID pandemic, patients esp the older population face mobility issues

2. What are Changing Health, Oviva and MyCognition interventions?

3. Why were only age and sex chosen as covariates for matching? The participants should be matched based on A1c, other blood results, ethnicity…etc as well

4. Further to the comment above, while the authors argue that multivariate adjustment has been done in regression, the authors should still match the participants based on these important covariates then further adjust in the multivariate regression, known as ‘doubly-robust’ adjustment, which has been shown to further eliminate residual confounding. E.g. https://www.thelancet.com/journals/lanwpc/article/PIIS2666-6065(22)00032-3/fulltext

5. Why did the authors choose to select a ratio as high as 1:20 for matching? For other studies e.g. propensity score matching it is usually as high as 1:5 only. By choosing a lower ratio, imputation may not even need to be done as you can select patients with complete set of data

6. Any information on medication compliance?

Reviewer #3: This manuscript presents a well-conducted and clearly written evaluation of the MyWay Diabetes (MDW) digital self-management intervention among people with type 2 diabetes in Greater Manchester during the COVID-19 pandemic. The topic is timely and important, addressing how large-scale digital tools can support diabetes care and long-term cost-effectiveness.

The study design is appropriate, the analytical approach is rigorous, and the conclusions are well supported by the data. The manuscript reads smoothly and maintains a professional, balanced tone. I appreciate the authors’ careful handling of real-world data and their transparent discussion of limitations.

That said, a few revisions would improve clarity, transparency, and presentation.

Major comments

1. Participant selection and engagement

The possibility of self-selection bias is acknowledged but not further explored. Including simple engagement metrics—such as login frequency, module completion rate, or duration of use—would help readers understand whether the observed effects reflect platform engagement or user motivation. If such data are unavailable, please note this explicitly and discuss the limitation.

2. Matching and baseline comparability

While age- and sex-matching is reasonable, some differences remain in ethnicity and the proportion of participants with HbA1c >53 mmol/mol. Reporting standardized mean differences or presenting a balance table would improve transparency and reader confidence in group comparability.

3. Handling of missing data

Multiple imputation is appropriate, but the manuscript would benefit from a short summary of missingness rates and patterns for key variables in the main text, not only in the Supplement. Please also confirm that the assumption of data being “missing at random” is justified.

4. Medication use and costs

The finding of a smaller increase in medication costs among MDW users is interesting but may also reflect prescribing behaviour or medication adherence rather than a direct effect of the intervention. This should be acknowledged more explicitly in the Discussion.

5. Economic modelling transparency

The economic evaluation using the UKPDS Outcomes Model (v2.1) is methodologically appropriate and consistent with NICE guidance. However, the absence of a probabilistic sensitivity analysis limits uncertainty assessment. Please highlight this limitation more clearly, and consider adding a concise table or appendix summarising key input parameters for reproducibility.

Minor comments

- A simple flow diagram outlining inclusion, exclusion, and matching would enhance clarity.

- Figure 1 (cost-effectiveness analysis): please refine the legend to explain all line types clearly (e.g., horizontal dashed = current uptake; vertical dashed = full GM licence cost; dotted = discounted cost scenarios). Ensure both axes are labelled with units.

- Table 1: consider rounding p-values to two decimal places and aligning numerical values for readability. Add short notes for abbreviations (e.g., “SD = standard deviation; IMD = Index of Multiple Deprivation”).

- Table 2: add a brief note in the legend stating that models were adjusted for baseline risk factors and time between measurements, so the table is interpretable independently from the text.

- Supplementary figures (e4.01–e4.05): line styles and colours for best- and worst-case scenarios are difficult to distinguish; use distinct line types or include an in-figure legend. Ensure all axes include units (e.g., “probability,” “cost £”).

- Referencing in the Supplement: indicate that citations correspond to those in the main manuscript to avoid confusion during typesetting.

- Summary of supplement: the supplementary material is comprehensive and strengthens the manuscript; still, briefly summarising key supplementary results (missing data, sensitivity assumptions) in the main text would make the paper more self-contained.

- Maintain tense consistency (prefer past tense in Methods).

- The paragraph on speculative behavioural mechanisms could be shortened or moved to the supplement for better flow.

- Highlight early in the Introduction that MDW directly integrates with NHS primary-care data, which distinguishes it from other digital interventions.

Ethics and data availability

Ethical approval and data governance are clearly described (Northwest GM South REC 261268). The data-availability restriction under the UK Data Protection Act is understandable; however, if feasible, providing access to analytic code or a synthetic dataset would further enhance transparency and reproducibility.

Language and presentation

The manuscript is written in fluent and professional English. The overall presentation is clear and polished. Minor stylistic edits may improve rhythm and consistency, but no substantive language issues were found. Figures and tables are generally well prepared and consistent with the results; the minor refinements above would make them publication-ready.

This is a high-quality and methodologically sound study that meets PLOS ONE standards for scientific validity. The paper makes a valuable contribution to the evidence base for digital diabetes management. Addressing the suggested clarifications—particularly regarding engagement data, baseline balance, and presentation details—will further strengthen an already excellent manuscript.

Reviewer #4: This quasi-experimental study evaluated the clinical and economic impact of the MyWay Diabetes (MDW) digital self-management platform among adults with type 2 diabetes (T2D) in Greater Manchester during the COVID-19 pandemic. The study suggested that MyWay Diabetes offers a scalable, low-cost digital tool that enhances diabetes self-management and modestly improves key cardiometabolic risk factors. The integration of MyWay Diabetes into primary care systems was considered cost effective by reducing long-term complication risks.

1. The major concern for this study is the non-randomised design, which warrants proper statement as a limitation.

2. The presentation of this study needs improvement including figures and tables. For example, in Table 1, it is unclear what N available and data mean. For outcomes, it is important to report the changes in each group before the difference between arms.

3. The cost-effectiveness model is not quite clear and a bubble plot is necessary in the main text.

4. It is relevant to apply more risk calculators such as Qrisk3 for risk assessment.

**********

6. PLOS authors have the option to publish the peer review history of their article (what does this mean?). If published, this will include your full peer review and any attached files.

If you choose “no”, your identity will remain anonymous but your review may still be made public.

**Do you want your identity to be public for this peer review?**  For information about this choice, including consent withdrawal, please see our Privacy Policy.

Reviewer #1: No

Reviewer #2: No

Reviewer #3: No

Reviewer #4: No

**********

[NOTE: If reviewer comments were submitted as an attachment file, they will be attached to this email and accessible via the submission site. Please log into your account, locate the manuscript record, and check for the action link "View Attachments". If this link does not appear, there are no attachment files.]

To ensure your figures meet our technical requirements, please review our figure guidelines: https://journals.plos.org/plosone/s/figures

You may also use PLOS’s free figure tool, NAAS, to help you prepare publication quality figures: https://journals.plos.org/plosone/s/figures#loc-tools-for-figure-preparation.

NAAS will assess whether your figures meet our technical requirements by comparing each figure against our figure specifications.

# Author comment: Author response to Decision Letter 1

**DOI:** 10.1371/journal.pone.0349232.r002

22 Jan 2026

Dear Editorial Team,

PONE-D-25-41865; Clinical and cost-effectiveness of the digital intervention, MyWay Diabetes, in people with type 2 diabetes living in Greater Manchester during the COVID-19 pandemic.

We are very grateful that we have been provided with another opportunity to refine and improve our manuscript. We have provided point-by-point responses below to each comment from the reviewers.

We trust that the manuscript is now suitable for publication in your journal and we will look forward to hearing from you.

Your sincerely,

Rathi Ravindrarajah, PhD

On behalf of the authors

……………………………..

5. Review Comments to the Author

Please use the space provided to explain your answers to the questions above. You may also include additional comments for the author, including concerns about dual publication, research ethics, or publication ethics. (Please upload your review as an attachment if it exceeds 20,000 characters)

Reviewer #1: The paper by Ravindrarajah and colleagues aimed to explore the use of digital intervention, MyWay Diabetes (MDW) in improving glycemic control and in reducing cardiovascular risk in individuals with type 2 diabetes during the COVID-19 pandemic in Greater Manchester. It also aims to establish the cost-effectiveness of this digital intervention in view of expanding its use in larger number of individuals. The Authors demonstrated that over 2 years follow up there was a reduction of 3.1 mmol/mol of HbA1c in patients using MDW as compared to non-users. Also, there was an improve in blood pressure and cholesterol level. In terms of costs, there was a reduction in patients using MDW as compared to non-users. The study is well conducted with rigorous methodology. The statistical analysis included major cofactors and covariates to ensure the best approach. The novelty is mainly represented by the use of MDW. For the first time also the economic burden of developing type 2 diabetes and its link with the development of complications is considered and analyzed. As stated in the limitations of the study, the length of follow-up of 2 years may have limited the results, it is advisable to prolong it and check whether a higher reduction in HbA1c and in blood pressure may be obtained although this may have a more negative impact on the costs.

Minor points to be addressed and/or commented:

Comment 1.1: Number of non-users vs. users: this is quite different. Have the Authors taken any measure to mitigate its impact?

Response 1.1: Thank you for your comments. We studied a higher number of non-users by design to maximise statistical power to show differences between people using the intervention and people not using it; we matched 1 user to up to 20 non-users. To mitigate any effect of an imbalance in the simple size, we matched users to non-users for age (within one year) and sex, and our analysis adjusted for several covariates that could potentially confound between-group differences in HbA1c, blood pressure and lipids.

Comment 1.2: Do the authors think that the use of certain medications may have impacted the results regardless of the use of MDW? Did they take this into account in their multivariable logistic regression?

Response 1.2: Overall, MWD users did not increase their diabetes medication prescriptions, while controls did. As we have explained in the discussion, this suggests that the intervention may have helped prevent the escalation of prescribing in MWD users, which was observed in the control group. In other words, MWD users required less medication as their condition naturally deteriorated over time, perhaps because MWD led to improvements in diet or physical activity driven by increased awareness of these key lifestyle factors.

However, when we conducted extensive supplementary analyses on specific intervention-related changes in diabetes, blood pressure and lipid lowering medication, and we found no clinically significant changes in the prescribing of specific drug classes, when comparing MWD users and non-users (Supplementary e2.2 and tables e2.06-2.08). Since, there were no specific medication classes that appeared to mediate intervention-related between-group differences in HbA1c, blood pressure, and cholesterol, there was no case for including specific drug classes in the multivariable-adjusted analysis, in a mediation framework.

Comment 1.3: Were patients monitored for glucose levels with sensors? This may have impacted in improving results of the use of MDW. The Authors may add a comment.

Response 1.3: The purpose of our study was to evaluate the digital intervention MWD, in the setting of usual care and therefore we did not supplement the intervention with continuous glucose monitoring. A small number of participants in both MWD and non-MWD groups may have been using continuous glucose monitoring as part of the clinical care; our study made no attempt to change this routine care.

Reviewer #2: The authors performed an interesting study. I have the following comments:

Comment 2.1: The authors may want to highlight more on the importance of digital intervention – 1. During COVID where face-to-face consultations are not feasible and 2. Even after COVID pandemic, patients esp the older population face mobility issues.

Response 2.1: We are grateful to the reviewer for this suggestion. In response, we have added the following text in the Introduction (Page 3: lines 94-96), “Digital solutions offer clear benefits over usual care when face-to-face care is limited, such as during the COVID-19 pandemic, and in people with limited mobility.”

Comment 2.2: What are Changing Health, Oviva and MyCognition interventions?

Response 2.2: Oviva and Changing Health both provide structured, personalized 12-week behaviour-change programmes delivered via apps and remote coaching designed to improve glucose control and body weight. MyCognition focuses on enhancing cognitive performance and mental resilience through personalised digital training, with studies indicating improvements in cognition that may support better diabetes self-management.

As we explained in the text of the introduction, we initially planned to evaluate these digital interventions that were also offered to MWD users, but since uptake was very low, we presented results only for MWD (lines 108-109, page 3). We explained in the results section that among MWD registrants, only 15 (3%) also used MyCognition, 7 (1.4%) also used Changing Health, 0 (0%) also used Oviva (lines 224-226, page 8). Excluding participants who also used Changing Health and MyCognition interventions had no effect on main study outcomes (Supplementary Table e2.03).

Comment 2.3: Why were only age and sex chosen as covariates for matching? The participants should be matched based on A1c, other blood results, ethnicity…etc as well

Response 2.3: Although we can understand the reviewer’s concerns, our approach is established practice for such analyses i.e., matching on key demographic variables and then controlling for remaining covariates of interest in a regression model. In a simulation study we have shown that this approach works well if cohort size is reasonably high,1 as it is in the current study. Matching on many variables can reduce the control pool so much that matching becomes impossible.

Comment 2.4: Further to the comment above, while the authors argue that multivariate adjustment has been done in regression, the authors should still match the participants based on these important covariates then further adjust in the multivariate regression, known as ‘doubly-robust’ adjustment, which has been shown to further eliminate residual confounding. E.g. https://www.thelancet.com/journals/lanwpc/article/PIIS2666-6065(22)00032-3/fulltext [thelancet.com]

Response 2.4: We agree with the use of the “doubly robust” statistical method highlighted by the reviewer. In all analyses, we have adjusting for the covariates we matched on; age and sex in this case, in line with best practice. However, as we explained in our response to the point above, the exact matching approach needs to be able to procure enough controls in the first place. In our study, matching on more variables would have reduced the number of controls and reduced the statistical power of our analysis.

An alternative methodology is propensity score matching, but, as we explained in our response above, our simulation study1 has shown that the approach that we adopted in our analysis is as good, if not better, when sample size is reasonably large as it was in this study. Propensity score matching approaches were developed with small samples in mind.

Comment 2.5: Why did the authors choose to select a ratio as high as 1:20 for matching? For other studies e.g. propensity score matching it is usually as high as 1:5 only. By choosing a lower ratio, imputation may not even need to be done as you can select patients with complete set of data

Response 2.5: Thank you for your comment. The 1:20 matching ratio was chosen to maximise statistical power and improve the precision of our estimates by making full use of available data in our non-user controls.

We agree that exact or propensity score matching studies often use lower ratios (e.g., up to 1:5). This tends to be the case because there may be smaller pools of controls available in many studies and/or because the relationship between control numbers and power is log-linear and therefore benefits are small as numbers increase - especially with more than 10 controls. Nevertheless, because we had a large control pool, we aimed to maximise power by matching up to 1:20.

Selecting only patients with complete data would arguably be problematic because of recording bias. In primary care research, patients with poorer health tend to have more complete data because they visit their GP surgery more often. These controls with complete data could be systematically different to the general cohort of people with type 2 diabetes. Our aim was to generate generalisable data that would inform about the potential impact of the MWD intervention when made available to all people with type 2 diabetes in primary care, and so we aimed to be as inclusive as possible for both MWD users and non-user controls.

Comment 2.6: Any information on medication compliance?

Response 2.6: We had information on medication prescribing, but we did not have access to any information on whether medication was dispensed or taken by the patient. Assessing medication compliance in UK primary care data is difficult because there are no routinely available data to indicate if medicines are actually taken.

However, as previously stated above, our results suggest that the positive effects of MWD intervention are not mediated by higher medication use; the opposite seems to be true. My Way Diabetes users tend to experience less of an increase in prescribing (and by inference, medication use) compared to no users during the study.

Reviewer #3: This manuscript presents a well-conducted and clearly written evaluation of the MyWay Diabetes (MDW) digital self-management intervention among people with type 2 diabetes in Greater Manchester during the COVID-19 pandemic. The topic is timely and important, addressing how large-scale digital tools can support diabetes care and long-term cost-effectiveness.

The study design is appropriate, the analytical approach is rigorous, and the conclusions are well supported by the data. The manuscript reads smoothly and maintains a professional, balanced tone. I appreciate the authors’ careful handling of real-world data and their transparent discussion of limitations.

That said, a few revisions would improve clarity, transparency, and presentation.

Major comments

Comment 3.1: Participant selection and engagement

The possibility of self-selection bias is acknowledged but not further explored. Including simple engagement metrics—such as login frequency, module completion rate, or duration of use—would help readers understand whether the observed effects reflect platform engagement or user motivation. If such data are unavailable, please note this explicitly and discuss the limitation.

Response 3.1: We don’t have access to these data, and we referred to this in the discussion but recognise that our text could have been clearer. In response to this helpful comment, we have modified the following text in the limitations section of the discussion which previously read, “data on participant engagement was not collected, limiting our ability to assess sustained use and its potential influence on effectiveness”. It now reads, “data on MWD usage/participant engagement was not collected, limiting our ability to assess the impact of motivation and sustained use on effectiveness”.

Comment 3.2: Matching and baseline comparability.

While age- and sex-matching is reasonable, some differences remain in ethnicity and the proportion of participants with HbA1c >53 mmol/mol. Reporting standardized mean differences or presenting a balance table would improve transparency and reader confidence in group comparability.

Response

Thank you. These data provided in tables e2.03-e2.05 in the supplementary document.

Table e2.03 shows multivariable-adjusted mean differences in post intervention primary and secondary outcomes between MWD users and matched controls not using MWD, after excluding participants who also used Changing Health and MyCognition interventions.

Table e2.04 shows multivariable-adjusted mean differences in primary and secondary outcomes between MWD users and matched controls not using MWD, using paired available outcome data.

Table e2.05 shows within-group changes in outcomes for test bed participants and matched controls who did not take up the intervention (pre and post-intervention levels are shown).

We have focused on the outcome variables because they are of greatest importance. Please note that we adjust all analyses for any differences in baseline levels of these outcomes, and other covariates including ethnicity, in the multivariable-adjusted analyses.

In a simulation study,1 we have shown that controlling for these covariates in a regression modelling framework is as good if not better than propensity score matching approaches when samples are large enough, as they are in our study.

Comment 3.3: Handling of missing data

Multiple imputation is appropriate, but the manuscript would benefit from a short summary of missingness rates and patterns for key variables in the main text, not only in the Supplement. Please also confirm that the assumption of data being “missing at random” is justified.

Response 3.3: Thank you. We have now added a short summary of missingness and patterns for key variables, lines 241-249, page 8-9.

“Across outcomes, the proportion of missing data was consistently higher at the post-intervention timepoint than at baseline for both DMW users and controls. For the primary outcome (HbA1c), missingness increased from 3.9% to 21.9% among DMW users and from 13.1% to 31.2% among controls. Similar patterns were observed for secondary outcomes: systolic and diastolic blood pressure missingness rose from 8.7% to 21.5% in users and from 16.1% to 33.4% in controls; total cholesterol missingness increased from 8.1% to 28.2% in users and from 17.5% to 36.8% in controls; and body weight missingness rose from 10.8% to 30.0% in users and from 20.8% to 41.5% in controls. Exploratory outcomes also showed substantial missingness, with post-intervention missing data ranging from 22.1% to 65.3% among users and 31.7% to 74.9% among controls. “

Regarding the MAR assumption, we cannot evaluate whether data are missing at random (MAR) or missing not at random (MNAR). However, it is known that multiple imputation is preferrable to a complete case analysis not only when data are missing completely at random or MCAR (increasing power) or MAR (reducing/minimising bias), but even when data are MNAR, as we have shown in a published simulation study.2

It is important to note that we performed sensitivity analyses using complete-case data to assess the robustness of the findings using multiple imputation, with agreement in the results across the two approaches.

Comment 3.4: Medication use

Attachment Submitted filename: Response to Reviewers.docx

# Peer review: Decision Letter 1

**DOI:** 10.1371/journal.pone.0349232.r003

**Authors:** Ennio Polilli, Ennio Polilli

27 Feb 2026

PONE-D-25-41865R1

Clinical and cost-effectiveness of the digital intervention, MyWay Diabetes, in people with type 2 diabetes living in Greater Manchester during the COVID-19 pandemic.

PLOS One

Dear Dr.  Ravindrarajah,

Thank you for submitting your manuscript to PLOS ONE. After careful consideration, we feel that it has merit but does not fully meet PLOS ONE’s publication criteria as it currently stands. Therefore, we invite you to submit a revised version of the manuscript that addresses the points raised during the review process.

Please submit your revised manuscript by Apr 13 2026 11:59PM. If you will need more time than this to complete your revisions, please reply to this message or contact the journal office at plosone@plos.org. When you're ready to submit your revision, log on to https://www.editorialmanager.com/pone/ and select the 'Submissions Needing Revision' folder to locate your manuscript file.

Please include the following items when submitting your revised manuscript:

- A letter that responds to each point raised by the academic editor and reviewer(s). You should upload this letter as a separate file labeled 'Response to Reviewers'.
- A marked-up copy of your manuscript that highlights changes made to the original version. You should upload this as a separate file labeled 'Revised Manuscript with Track Changes'.
- An unmarked version of your revised paper without tracked changes. You should upload this as a separate file labeled 'Manuscript'.

-->If you would like to make changes to your financial disclosure, please include your updated statement in your cover letter. Guidelines for resubmitting your figure files are available below the reviewer comments at the end of this letter.

If applicable, we recommend that you deposit your laboratory protocols in protocols.io to enhance the reproducibility of your results. Protocols.io assigns your protocol its own identifier (DOI) so that it can be cited independently in the future. For instructions see: https://journals.plos.org/plosone/s/submission-guidelines#loc-laboratory-protocols. Additionally, PLOS ONE offers an option for publishing peer-reviewed Lab Protocol articles, which describe protocols hosted on protocols.io. Read more information on sharing protocols at https://plos.org/protocols?utm_medium=editorial-email&utm_source=authorletters&utm_campaign=protocols.

We look forward to receiving your revised manuscript.

Kind regards,

Ennio Polilli

Academic Editor

PLOS One

Journal Requirements:

1. If the reviewer comments include a recommendation to cite specific previously published works, please review and evaluate these publications to determine whether they are relevant and should be cited. There is no requirement to cite these works unless the editor has indicated otherwise.

2. Please review your reference list to ensure that it is complete and correct. If you have cited papers that have been retracted, please include the rationale for doing so in the manuscript text, or remove these references and replace them with relevant current references. Any changes to the reference list should be mentioned in the rebuttal letter that accompanies your revised manuscript. If you need to cite a retracted article, indicate the article’s retracted status in the References list and also include a citation and full reference for the retraction notice.

[Note: HTML markup is below. Please do not edit.]

Reviewer's Responses to Questions

**Comments to the Author**

1. If the authors have adequately addressed your comments raised in a previous round of review and you feel that this manuscript is now acceptable for publication, you may indicate that here to bypass the “Comments to the Author” section, enter your conflict of interest statement in the “Confidential to Editor” section, and submit your "Accept" recommendation.

Reviewer #1: All comments have been addressed

Reviewer #2: All comments have been addressed

Reviewer #4: All comments have been addressed

**********

2. Is the manuscript technically sound, and do the data support the conclusions?

The manuscript must describe a technically sound piece of scientific research with data that supports the conclusions. Experiments must have been conducted rigorously, with appropriate controls, replication, and sample sizes. The conclusions must be drawn appropriately based on the data presented.

Reviewer #1: Yes

Reviewer #2: Yes

Reviewer #4: Yes

**********

3. Has the statistical analysis been performed appropriately and rigorously?

Reviewer #1: Yes

Reviewer #2: No

Reviewer #4: Yes

**********

4. Have the authors made all data underlying the findings in their manuscript fully available?

The PLOS Data policy requires authors to make all data underlying the findings described in their manuscript fully available without restriction, with rare exception (please refer to the Data Availability Statement in the manuscript PDF file). The data should be provided as part of the manuscript or its supporting information, or deposited to a public repository. For example, in addition to summary statistics, the data points behind means, medians and variance measures should be available. If there are restrictions on publicly sharing data—e.g. participant privacy or use of data from a third party—those must be specified.

Reviewer #1: Yes

Reviewer #2: No

Reviewer #4: Yes

**********

5. Is the manuscript presented in an intelligible fashion and written in standard English?

PLOS ONE does not copyedit accepted manuscripts, so the language in submitted articles must be clear, correct, and unambiguous. Any typographical or grammatical errors should be corrected at revision, so please note any specific errors here.

Reviewer #1: Yes

Reviewer #2: Yes

Reviewer #4: Yes

**********

6. Review Comments to the Author

Please use the space provided to explain your answers to the questions above. You may also include additional comments for the author, including concerns about dual publication, research ethics, or publication ethics. (Please upload your review as an attachment if it exceeds 20,000 characters)

Reviewer #1: (No Response)

Reviewer #2: The authors submitted a revision of the manuscript. I have the following comments:

1. The authors calculated the sample size with 1:10 ratio. Please present in sensitivity analysis the findings if PS matching 1:10 is used, ideally these patients should be those with complete data.

2. What is meant by "age (within 1 year)"? Age should be fined according to that upon enrollment

3. Despite the authors having extensively responded to the comments regarding adjusting for covariates in regression vs PS matching, putting the covariates in the propensity score is a more commonly accepted approach. Please perform this a sensitivity analysis, accepting a lower ratio e.g. 1:5, which is also commonly adopted in the current literature. The authors may also argue that increasing the number of covariates may reduce matching, but (a) it can be done with a lower ratio (b) at the authors quoted in their own previous simulation study, logistic regression only outperformed PS when sample size is large enough. The authors may also consider use of covariate-balancing PS (CBPS) with doubly-robust adjustment - please refer to the paper I cited in my previous comments.

Reviewer #4: Thanks for the revised manuscript, which improve the paper significantly. I have no more comments or suggestions and am happy to find its publication.

**********

7. PLOS authors have the option to publish the peer review history of their article (what does this mean?). If published, this will include your full peer review and any attached files.

If you choose “no”, your identity will remain anonymous but your review may still be made public.

**Do you want your identity to be public for this peer review?**  For information about this choice, including consent withdrawal, please see our Privacy Policy.

Reviewer #1: No

Reviewer #2: No

Reviewer #4: No

**********

[NOTE: If reviewer comments were submitted as an attachment file, they will be attached to this email and accessible via the submission site. Please log into your account, locate the manuscript record, and check for the action link "View Attachments". If this link does not appear, there are no attachment files.]

To ensure your figures meet our technical requirements, please review our figure guidelines: https://journals.plos.org/plosone/s/figures

You may also use PLOS’s free figure tool, NAAS, to help you prepare publication quality figures: https://journals.plos.org/plosone/s/figures#loc-tools-for-figure-preparation.

NAAS will assess whether your figures meet our technical requirements by comparing each figure against our figure specifications.

# Author comment: Author response to Decision Letter 2

**DOI:** 10.1371/journal.pone.0349232.r004

19 Mar 2026

Dear Editorial Team,

PONE-D-25-41865; Clinical and cost-effectiveness of the digital intervention, MyWay Diabetes, in people with type 2 diabetes living in Greater Manchester during the COVID-19 pandemic.

We are very grateful that we have been provided with an opportunity to further improve our manuscript. Here we provide a point-by-point response to each comment from the reviewers.

We trust that the manuscript is now suitable for publication in your journal and we look forward to hearing from you.

Your sincerely,

Rathi Ravindrarajah, PhD

On behalf of the authors

……………………………………………………………………………………………………………………………………………

Reviewer #2: The authors submitted a revision of the manuscript. I have the following comments:

Comment 1. The authors calculated the sample size with 1:10 ratio. Please present in sensitivity analysis the findings if PS matching 1:10 is used, ideally these patients should be those with complete data.

Response 1: Thank you for this comment. However, we are not entirely certain that we have interpreted the suggestion correctly as the wording of the comment is somewhat unclear. We understand that the reviewer may be suggesting conducting a sensitivity analysis using PS matching with a 1:10 ratio.

We have already conducted numerous sensitivity analyses to assess the robustness of our findings. In our view, the proposed sensitivity analysis would not provide useful additional insights beyond the approaches already implemented, nor is it justified in the context of our study design. We have therefore not added the analysis.

Our rationale is explained in more detail below (please see our response to point 3 below), where we discuss the methodological considerations relevant to the suggestion.

Comment 2. What is meant by "age (within 1 year)"? Age should be fined according to that upon enrolment

Response 2: We thank the reviewer for this comment. "Age within 1 year" refers to the matching criterion applied during control selection, whereby each case (MWD user) was matched to controls whose age differed by no more than one year. Age was defined at the “index date”, which in such observational studies represents the time point at which follow-up begins. In our study, the index date corresponds to the date of first use the intervention for cases (MWD users); matched controls inherit the index date from their matched MWD user.

In the dataset used only birthyear was available to preserve participant anonymity. As a result, a small degree of variation in exact age will occur. In response to this helpful comment, we have revised the manuscript to clarify the definition of age and the matching procedure (lines 146-148, page 5).

This text reads, ‘Matching for birth year (within 1 year) and sex was performed, with each MWD user matched to up to 20 controls not using MWD to increase statistical power. Age and sex were defined at the MWD start date for each user, which served as the index date for the corresponding matched control non-users.’

Comment 3. Despite the authors having extensively responded to the comments regarding adjusting for covariates in regression vs PS matching, putting the covariates in the propensity score is a more commonly accepted approach. Please perform this a sensitivity analysis, accepting a lower ratio e.g. 1:5, which is also commonly adopted in the current literature. The authors may also argue that increasing the number of covariates may reduce matching, but (a) it can be done with a lower ratio (b) at the authors quoted in their own previous simulation study, logistic regression only outperformed PS when sample size is large enough. The authors may also consider use of covariate-balancing PS (CBPS) with doubly-robust adjustment - please refer to the paper I cited in my previous comments.

Response 3: We appreciate the opportunity to clarify our methodological choices. We respectfully maintain that the analytical approach used in our study remains appropriate for the research question and the data structure.

In observational studies, using EHR data, the approach we used - a combination of exact matching on key variables and covariate adjustment is a commonly accepted approach, and has become the norm. As noted previously, PS matching methods are primarily intended as a dimension reduction strategy, which is particularly useful when the number of covariates is large relative to the sample size.

In our study, however, we have a substantial sample size of close to 11,000 participants, and therefore direct covariant adjustment through regression modelling is both feasible and methodologically appropriate.

It is also well recognised that including a large number of covariates in the PS can sometimes lead to poorer matching on key covariates (the reviewer may have misunderstood that point, our argument was this, not that numbers to be matched would be reduced). This is the reason why best practice is to combine matching on the PS with adjustment for the included covariates, rather than relying solely on PS matching. When the sample size is large this effectively makes the matching process pointless.

The reviewer also refers to our previously cited simulation study. In that work, regression adjustment performed similarly or better than PS-based approaches when the sample size exceeded 1,000. Given that our sample includes nearly 11,000 individuals, this supports the suitability of the regression-based approach that we used.

Regarding the suggestion to use a lower matching ratio (e.g., 1:5), our concern is that this will reduce the statistical power of the study without addressing a clear methodological concern. Lower ratios are often used in other studies where there are not enough controls to match on, which is not the case in our data.

For these reasons, and given the multiple sensitivity analyses already performed, we believe that additional analyses proposed would be unlikely to materially alter the key findings in our manuscript.

Attachment Submitted filename: Response_to_Reviewers_auresp_2.docx

# Peer review: Decision Letter 2

**DOI:** 10.1371/journal.pone.0349232.r005

**Authors:** Ennio Polilli, Ennio Polilli, Ennio Polilli

13 Apr 2026

PONE-D-25-41865R2

Clinical and cost-effectiveness of the digital intervention, MyWay Diabetes, in people with type 2 diabetes living in Greater Manchester during the COVID-19 pandemic.

PLOS One

Dear Dr. Ravindrarajah,

Thank you for submitting your manuscript to PLOS ONE. After careful consideration, we feel that it has merit but does not fully meet PLOS ONE’s publication criteria as it currently stands. Therefore, we invite you to submit a revised version of the manuscript that addresses the points raised during the review process.

Please submit your revised manuscript by May 28 2026 11:59PM. If you will need more time than this to complete your revisions, please reply to this message or contact the journal office at plosone@plos.org. When you're ready to submit your revision, log on to https://www.editorialmanager.com/pone/ and select the 'Submissions Needing Revision' folder to locate your manuscript file.

Please include the following items when submitting your revised manuscript:

- A letter that responds to each point raised by the academic editor and reviewer(s). You should upload this letter as a separate file labeled 'Response to Reviewers'.
- A marked-up copy of your manuscript that highlights changes made to the original version. You should upload this as a separate file labeled 'Revised Manuscript with Track Changes'.
- An unmarked version of your revised paper without tracked changes. You should upload this as a separate file labeled 'Manuscript'.

If you would like to make changes to your financial disclosure, please include your updated statement in your cover letter. Guidelines for resubmitting your figure files are available below the reviewer comments at the end of this letter.

If applicable, we recommend that you deposit your laboratory protocols in protocols.io to enhance the reproducibility of your results. Protocols.io assigns your protocol its own identifier (DOI) so that it can be cited independently in the future. For instructions see: https://journals.plos.org/plosone/s/submission-guidelines#loc-laboratory-protocols. Additionally, PLOS ONE offers an option for publishing peer-reviewed Lab Protocol articles, which describe protocols hosted on protocols.io. Read more information on sharing protocols at https://plos.org/protocols?utm_medium=editorial-email&utm_source=authorletters&utm_campaign=protocols.

As the corresponding author, your ORCID iD is verified in the submission system and will appear in the published article. PLOS supports the use of ORCID, and we encourage all coauthors to register for an ORCID iD and use it as well. Please encourage your coauthors to verify their ORCID iD within the submission system before final acceptance, as unverified ORCID iDs will not appear in the published article. *Only*  the individual author can complete the verification step; PLOS staff *cannot*  verify ORCID iDs on behalf of authors.

We look forward to receiving your revised manuscript.

Kind regards,

Ennio Polilli

Academic Editor

PLOS One

Journal Requirements:

If the reviewer comments include a recommendation to cite specific previously published works, please review and evaluate these publications to determine whether they are relevant and should be cited. There is no requirement to cite these works unless the editor has indicated otherwise.

Please review your reference list to ensure that it is complete and correct. If you have cited papers that have been retracted, please include the rationale for doing so in the manuscript text, or remove these references and replace them with relevant current references. Any changes to the reference list should be mentioned in the rebuttal letter that accompanies your revised manuscript. If you need to cite a retracted article, indicate the article’s retracted status in the References list and also include a citation and full reference for the retraction notice.

[Note: HTML markup is below. Please do not edit.]

Reviewers' comments:

Reviewer's Responses to Questions

**Comments to the Author**

1. If the authors have adequately addressed your comments raised in a previous round of review and you feel that this manuscript is now acceptable for publication, you may indicate that here to bypass the “Comments to the Author” section, enter your conflict of interest statement in the “Confidential to Editor” section, and submit your "Accept" recommendation.

Reviewer #2: All comments have been addressed

Reviewer #3: (No Response)

**********

2. Is the manuscript technically sound, and do the data support the conclusions?

The manuscript must describe a technically sound piece of scientific research with data that supports the conclusions. Experiments must have been conducted rigorously, with appropriate controls, replication, and sample sizes. The conclusions must be drawn appropriately based on the data presented.

Reviewer #2: Yes

Reviewer #3: Yes

**********

3. Has the statistical analysis been performed appropriately and rigorously?

Reviewer #2: Yes

Reviewer #3: Yes

**********

4. Have the authors made all data underlying the findings in their manuscript fully available?

The PLOS Data policy requires authors to make all data underlying the findings described in their manuscript fully available without restriction, with rare exception (please refer to the Data Availability Statement in the manuscript PDF file). The data should be provided as part of the manuscript or its supporting information, or deposited to a public repository. For example, in addition to summary statistics, the data points behind means, medians and variance measures should be available. If there are restrictions on publicly sharing data—e.g. participant privacy or use of data from a third party—those must be specified.

Reviewer #2: Yes

Reviewer #3: No

**********

5. Is the manuscript presented in an intelligible fashion and written in standard English?

PLOS ONE does not copyedit accepted manuscripts, so the language in submitted articles must be clear, correct, and unambiguous. Any typographical or grammatical errors should be corrected at revision, so please note any specific errors here.

Reviewer #2: Yes

Reviewer #3: Yes

**********

6. Review Comments to the Author

Please use the space provided to explain your answers to the questions above. You may also include additional comments for the author, including concerns about dual publication, research ethics, or publication ethics. (Please upload your review as an attachment if it exceeds 20,000 characters)

Reviewer #2: The authors have addressed the comments adequately.

Reviewer #3: Final version

I thank the authors for their thorough and thoughtful responses to my previous comments. The majority of revisions have been addressed satisfactorily, and the manuscript has improved considerably. However, two points require further clarification before I can recommend acceptance.

Remaining concerns

1. Baseline comparability (Major comment 3.2)

I appreciate the authors' response, but I note that the tables referenced (e2.03–e2.05) present outcome data rather than baseline covariate balance. My original request was for standardized mean differences (SMDs) or a balance table showing comparability between MWD users and controls on key baseline characteristics — particularly ethnicity and the proportion with HbA1c >53 mmol/mol, where differences remain visible in Table 1.

Reporting SMDs for baseline covariates is standard practice in observational studies and would allow readers to assess residual imbalance independently of regression adjustment. I would suggest that the authors either: (a) add a column of SMDs to Table 1, or (b) provide a brief supplementary balance table. This is a reporting addition and does not require further analysis.

2. Flow diagram (Minor comment 3.6)

The authors have offered to move the flow diagram to the main text if considered helpful. I believe this would improve clarity and would encourage its inclusion in the main manuscript rather than in the supplementary material. A participant flow diagram is standard for studies of this type and aids reader comprehension.

Points addressed satisfactorily

All other major and minor comments have been addressed to my satisfaction, including:

Engagement data limitation is now clearly stated

Missing data summary and MAR assumption are appropriately described

Medication cost interpretation has been clarified

The absence of probabilistic sensitivity analysis is acknowledged

Figure and table legends have been improved

Supplementary figures have been clarified

The behavioural mechanism discussion has been refined

NHS data integration is now clearly highlighted

This remains a high-quality and important study. The remaining points are minor and relate to presentation only. I would be happy to recommend acceptance once these have been addressed.

**********

7. PLOS authors have the option to publish the peer review history of their article (what does this mean?). If published, this will include your full peer review and any attached files.

If you choose “no”, your identity will remain anonymous but your review may still be made public.

**Do you want your identity to be public for this peer review?**  For information about this choice, including consent withdrawal, please see our Privacy Policy.

Reviewer #2: No

Reviewer #3: No

**********

[NOTE: If reviewer comments were submitted as an attachment file, they will be attached to this email and accessible via the submission site. Please log into your account, locate the manuscript record, and check for the action link "View Attachments". If this link does not appear, there are no attachment files.]

To ensure your figures meet our technical requirements, please review our figure guidelines: https://journals.plos.org/plosone/s/figures

You may also use PLOS’s free figure tool, NAAS, to help you prepare publication quality figures: https://journals.plos.org/plosone/s/figures#loc-tools-for-figure-preparation.

NAAS will assess whether your figures meet our technical requirements by comparing each figure against our figure specifications.

# Author comment: Author response to Decision Letter 3

**DOI:** 10.1371/journal.pone.0349232.r006

23 Apr 2026

Dear Editorial Team,

PONE-D-25-41865; Clinical and cost-effectiveness of the digital intervention, MyWay Diabetes, in people with type 2 diabetes living in Greater Manchester during the COVID-19 pandemic.

We are very grateful that we have been provided with an opportunity to further improve our manuscript. Here we provide a point-by-point response to each comment from the reviewers.

We trust that the manuscript is now suitable for publication in your journal and we look forward to hearing from you.

Your sincerely,

Rathi Ravindrarajah, PhD

On behalf of the authors

……………………………………………………………………………………………………………………………………………

Reviewer #3: Final version

Ithank the authors for their thorough and thoughtful responses to my previous comments. The majority of revisions have been addressed satisfactorily, and the manuscript has improved considerably. However, two points require further clarification before I can recommend acceptance.

Remaining concerns

1. Baseline comparability (Major comment 3.2)

I appreciate the authors' response, but I note that the tables referenced (e2.03–e2.05) present outcome data rather than baseline covariate balance. My original request was for standardized mean differences (SMDs) or a balance table showing comparability between MWD users and controls on key baseline characteristics — particularly ethnicity and the proportion with HbA1c >53 mmol/mol, where differences remain visible in Table 1.

Reporting SMDs for baseline covariates is standard practice in observational studies and would allow readers to assess residual imbalance independently of regression adjustment. I would suggest that the authors either: (a) add a column of SMDs to Table 1, or (b) provide a brief supplementary balance table. This is a reporting addition and does not require further analysis.

Response 1: In response to this helpful comment, we have now added an extra column in Table 1 where the SMD values have been added. Pages 19-20

2. Flow diagram (Minor comment 3.6)

The authors have offered to move the flow diagram to the main text if considered helpful. I believe this would improve clarity and would encourage its inclusion in the main manuscript rather than in the supplementary material. A participant flow diagram is standard for studies of this type and aids reader comprehension.

Response 2: Thank you for your comments. In response we have now moved the flow diagrams from the appendix to the main text. [Pages 26-26]

Attachment Submitted filename: Response to Reviewers_200426.docx

# Peer review: Decision Letter 3

**DOI:** 10.1371/journal.pone.0349232.r007

**Authors:** Ennio Polilli, Ennio Polilli, Ennio Polilli, Ennio Polilli

27 Apr 2026

Clinical and cost-effectiveness of the digital intervention, MyWay Diabetes, in people with type 2 diabetes living in Greater Manchester during the COVID-19 pandemic.

PONE-D-25-41865R3

Dear Dr. Rutter,

We’re pleased to inform you that your manuscript has been judged scientifically suitable for publication and will be formally accepted for publication once it meets all outstanding technical requirements.

Within one week, you’ll receive an e-mail detailing the required amendments. When these have been addressed, you’ll receive a formal acceptance letter and your manuscript will be scheduled for publication.

An invoice will be generated when your article is formally accepted. Please note, if your institution has a publishing partnership with PLOS and your article meets the relevant criteria, all or part of your publication costs will be covered. Please make sure your user information is up-to-date by logging into Editorial Manager at Editorial Manager® and clicking the ‘Update My Information' link at the top of the page. For questions related to billing, please contact billing support.

If your institution or institutions have a press office, please notify them about your upcoming paper to help maximize its impact. If they’ll be preparing press materials, please inform our press team as soon as possible -- no later than 48 hours after receiving the formal acceptance. Your manuscript will remain under strict press embargo until 2 pm Eastern Time on the date of publication. For more information, please contact onepress@plos.org.

Kind regards,

Ennio Polilli

Academic Editor

PLOS One

Additional Editor Comments (optional):

Reviewers' comments:

Reviewer's Responses to Questions

**Comments to the Author**

1. If the authors have adequately addressed your comments raised in a previous round of review and you feel that this manuscript is now acceptable for publication, you may indicate that here to bypass the “Comments to the Author” section, enter your conflict of interest statement in the “Confidential to Editor” section, and submit your "Accept" recommendation.

Reviewer #3: All comments have been addressed

**********

2. Is the manuscript technically sound, and do the data support the conclusions?

The manuscript must describe a technically sound piece of scientific research with data that supports the conclusions. Experiments must have been conducted rigorously, with appropriate controls, replication, and sample sizes. The conclusions must be drawn appropriately based on the data presented.

Reviewer #3: Yes

**********

3. Has the statistical analysis been performed appropriately and rigorously?

Reviewer #3: Yes

**********

4. Have the authors made all data underlying the findings in their manuscript fully available?

The PLOS Data policy requires authors to make all data underlying the findings described in their manuscript fully available without restriction, with rare exception (please refer to the Data Availability Statement in the manuscript PDF file). The data should be provided as part of the manuscript or its supporting information, or deposited to a public repository. For example, in addition to summary statistics, the data points behind means, medians and variance measures should be available. If there are restrictions on publicly sharing data—e.g. participant privacy or use of data from a third party—those must be specified.

Reviewer #3: Yes

**********

5. Is the manuscript presented in an intelligible fashion and written in standard English?

PLOS ONE does not copyedit accepted manuscripts, so the language in submitted articles must be clear, correct, and unambiguous. Any typographical or grammatical errors should be corrected at revision, so please note any specific errors here.

Reviewer #3: Yes

**********

6. Review Comments to the Author

Please use the space provided to explain your answers to the questions above. You may also include additional comments for the author, including concerns about dual publication, research ethics, or publication ethics. (Please upload your review as an attachment if it exceeds 20,000 characters)

Reviewer #3: I thank the authors for addressing the remaining concerns from the previous round. The addition of standardized mean differences in Table 1 improves transparency in baseline comparability, and moving the participant flow diagrams into the main text enhances clarity. These revisions are satisfactory, and I have no further comments. I recommend acceptance.

**********

7. PLOS authors have the option to publish the peer review history of their article (what does this mean?). If published, this will include your full peer review and any attached files.

If you choose “no”, your identity will remain anonymous but your review may still be made public.

**Do you want your identity to be public for this peer review?**  For information about this choice, including consent withdrawal, please see our Privacy Policy.

Reviewer #3: No

**********

# Editor report: Acceptance letter

**DOI:** 10.1371/journal.pone.0349232.r008

**Authors:** Ennio Polilli, Ennio Polilli, Ennio Polilli, Ennio Polilli

PONE-D-25-41865R3

PLOS One

Dear Dr. Ravindrarajah,

I'm pleased to inform you that your manuscript has been deemed suitable for publication in PLOS One. Congratulations! Your manuscript is now being handed over to our production team.

At this stage, our production department will prepare your paper for publication. This includes ensuring the following:

* All references, tables, and figures are properly cited

* All relevant supporting information is included in the manuscript submission,

* There are no issues that prevent the paper from being properly typeset

You will receive further instructions from the production team, including instructions on how to review your proof when it is ready. Please keep in mind that we are working through a large volume of accepted articles, so please give us a few days to review your paper and let you know the next and final steps.

Lastly, if your institution or institutions have a press office, please let them know about your upcoming paper now to help maximize its impact. If they'll be preparing press materials, please inform our press team within the next 48 hours. Your manuscript will remain under strict press embargo until 2 pm Eastern Time on the date of publication. For more information, please contact onepress@plos.org.

You will receive an invoice from PLOS for your publication fee after your manuscript has reached the completed accept phase. If you receive an email requesting payment before acceptance or for any other service, this may be a phishing scheme. Learn how to identify phishing emails and protect your accounts at https://explore.plos.org/phishing.

If we can help with anything else, please email us at customercare@plos.org.

Thank you for submitting your work to PLOS ONE and supporting open access.

Kind regards,

PLOS ONE Editorial Office Staff

on behalf of

Dr. Ennio Polilli

Academic Editor

PLOS One
